# The interplay between hydrogen and halogen bonding: substituent effects and their role in the hydrogen bond enhanced halogen bond†

**DOI:** 10.1039/d3sc02348f

**Published:** 2023-07-21

**Authors:** Jiyu Sun, Daniel A. Decato, Vyacheslav S. Bryantsev, Eric A. John, Orion B. Berryman

**Affiliations:** a Department of Chemistry and Biochemistry, University of Montana, 32 Campus Drive Missoula MT 59812 USA orion.berryman@umontana.edu; b Chemical Sciences Division, Oak Ridge National Laboratory Oak Ridge TN 37831 USA

## Abstract

The hydrogen bond enhanced halogen bond (HBeXB) has recently been used to effectively improve anion binding, organocatalysis, and protein structure/function. In this study, we present the first systematic investigation of substituent effects in the HBeXB. NMR analysis confirmed intramolecular HBing between the amine and the electron-rich belt of the XB donor (N–H⋯I). Gas-phase density functional theory studies showed that the influence of HBing on the halogen atom is more sensitive to substitution on the HB donor ring (R_1_). The NMR studies revealed that the intramolecular HBing had a significant impact on receptor performance, resulting in a 50-fold improvement. Additionally, linear free energy relationship (LFER) analysis was employed for the first time to study the substituent effect in the HBeXB. The results showed that substituents on the XB donor ring (R_2_) had a competing effect where electron donating groups strengthened the HB and weakened the XB. Therefore, selecting an appropriate substituent on the adjacent HB donor ring (R_1_) could be an alternative and effective way to enhance an electron-rich XB donor. X-ray crystallographic analysis demonstrated that intramolecular HBing plays an important role in the receptor adopting the bidentate conformation. Taken together, the findings imply that modifying distal substituents that affect neighboring noncovalent interactions can have a similar impact to conventional *para* substitution substituent effects.

## Abstract 2

The hydrogen bond enhanced halogen bond can be modulated by changing substituents on the HB or XB donor ring. Thus, distant substituents can impact nearby noncovalent interactions and this can be used as a viable approach for molecular design.

## Graphical abstract

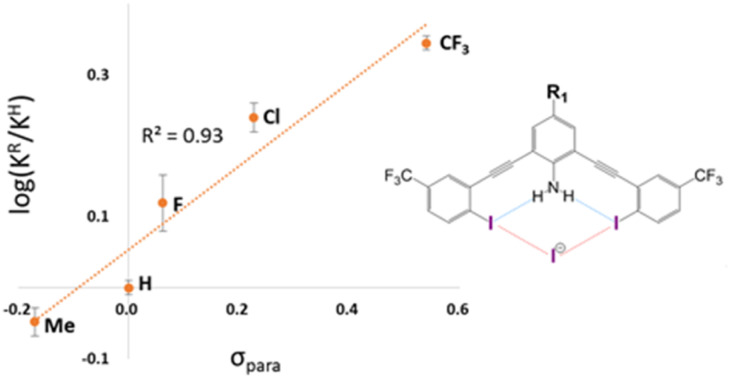

## Introduction

Understanding how to modulate noncovalent interactions that are in close proximity is paramount to engineering functional materials,^1^ supramolecular assemblies,^2^ drugs,^3^ and catalysts.^4^ Foundational work has detailed the importance of substituent effects^5^ to tune the electronics of noncovalent interactions.^6^ Despite this pioneering work, few have looked at substituent effects involving multiple noncovalent interactions that are spatially close. Recently, Cockroft, and coworkers reported rare experimental data quantifying through-space substituent effects on noncovalent interactions and presented the inadequacy of describing substituent effects using classic Hammett parameters when through-space effects dominate.^7^ Additionally, Zonta, and coworkers utilized similar methods to carry out an experimental survey of aromatic stacking interactions in solution.^8^ Clearly, understanding how adjacent noncovalent interactions influence each other by substituent effects is critically lacking.

Among the myriad noncovalent interactions, HBs are privileged for their directionality and tunability. Halogen bonds (XBs) share similarities with HBs, yet contain an electron deficient donor (halogen) that forms an attractive noncovalent interaction with an electron rich species. This interaction can be understood in term of electrostatics and covalency.^9^ An electrostatic description suggests that due to the polarizability of the halogen, its electron density can become anisotropic. In this case, a partial positive potential develops on the halogen, opposite to the C–X σ bond which has been coined the σ-hole. Concurrently, an electron rich belt is generated on the XB donor which is orthogonal to the direction of the σ bond.^10^ The high directionality, tunability and complementarity with “soft” Lewis bases,^11^ makes XBing accordant with HBing. Given these unique properties, exciting applications of XBing supramolecule self-assembly,^12^ molecular recognition,^13^ anion binders,^14^ organocatalysts,^15^ and anion transporters^16^ are appearing at a rapid pace. Understanding how to modulate both the strength of the XB and the structure of these molecules is of broad importance for the continued development of functional halogenated species. Using substituent effects to alter the electronics of the halogen donors remains a leading strategy. However, experimental substituent effects on XBing are limited (despite numerous computational studies^17^). Only Taylor,^18^ Diederich,^19^ Erdelyi,^20^ Stilinović^21^ and Franz^22^ have explicitly studied XBing substituent effects in solution—with different outcomes for each of their systems. Despite the critical importance of these substituent studies, the influence of a neighbouring noncovalent interaction has not been studied.

**Fig. 1:**
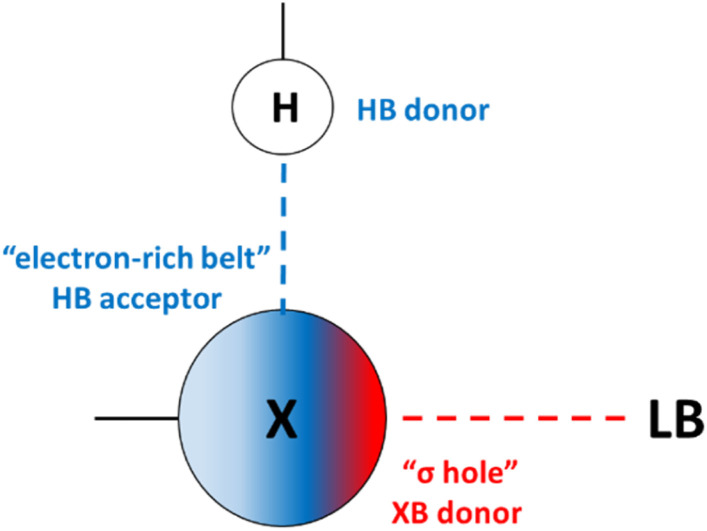
Cooperativity between HB and XB donors and acceptors in the HBeXB interaction. Blue dashed line: HB interaction, red dashed line: XB interaction, LB: Lewis base.

We recently introduced a new strategy—the hydrogen bond enhanced halogen bond (HBeXB)—that directs intramolecular HBs to the electron rich belt of XB donors for preorganization and enhanced XB strength (Fig. 1). Recently, the HBeXB interaction has been used to increase anion binding affinity by nearly an order of magnitude and to improve the function of organocatalysts.^14*b*,15*a*^ Similarly, Ho and his group have noted the HBeXB in biological settings and employed it to stabilize and improve a T4 lysozyme mutant.^23^ These studies have been complemented by fundamental studies as well.^14*b*,24^ Over the course of these seminal works there are implications of reciprocity between the hydrogen and halogen bond, especially when considering circumstances where augmentation and preorganization are simultaneously operating. In fact, using substituent effects to modulate the HBeXB may not be straightforward as there are potentially competing electronic effects. Making the halogen atom more electron rich may decrease the XB donor ability but will increase the strength of the adjacent HB. The subtle interplay between these two interactions naturally leads one to ask important questions. For example, when optimizing binding, is it more efficient to tune the XB strength or the HB strength with electronic effects? Additionally, how does this influence molecular conformations? To address these questions and more, we present the first HBeXB substituent and LFER studies in solution, gas, and solid phases.

## Results and discussion

### Receptor design

**Fig. 2:**
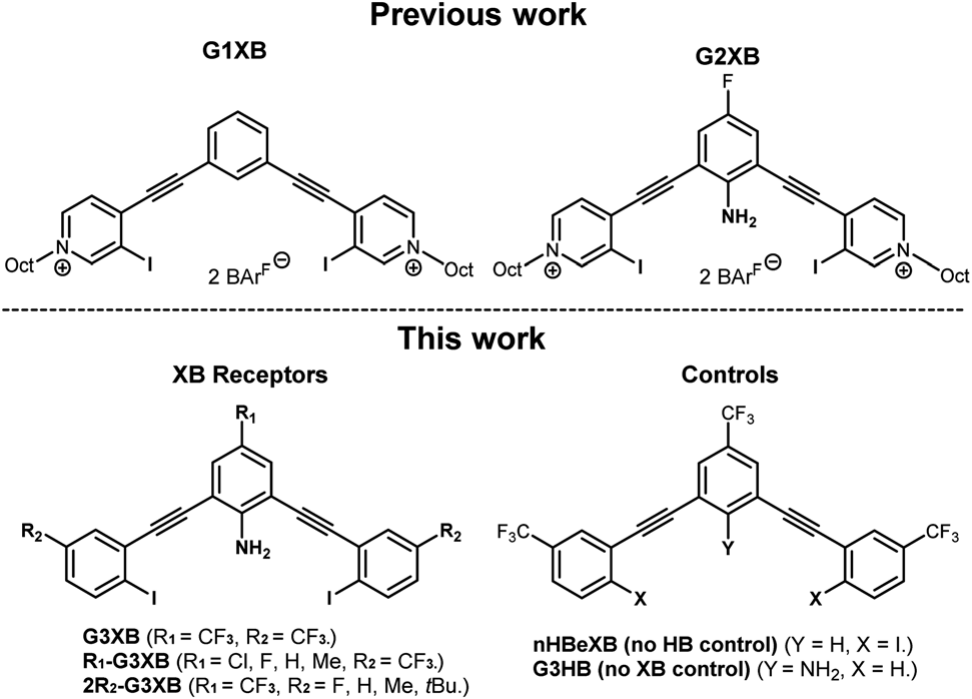
ChemDraw representation of previous XB receptors and G3XB derivatives in this work.

We previously developed two generations of bis-ethynyl XB receptors that presented two charged pyridinium XB donors in a convergent manner (Fig. 2 top). In the 2^nd^-generation receptor we introduced an amine substituent to the core that provided intramolecular N–H⋯I HBs to the XB donors. This innovation improved binding by nearly an order of magnitude over a control lacking the amine.^14*b*^ We coined this effect the HBeXB and showed that the enhancement was due to both preorganization of the receptor into a bidentate binding conformation and strengthening of the XB. The success of the HBeXB and our experience with this scaffold prompted us to evaluate HBeXB substituent effects using a 3^rd^-generation anion receptor presented here (G3XB, Fig. 2 bottom). This latest iteration replaces the flanking iodopyridinium rings with neutral aromatic rings to improve solubility in organic solvents. The redesign also reduced the number of synthetic steps to produce the diverse range of receptors needed to examine substituent effects. Specifically, we prepared a series of compounds that contained substituents of varying electronic properties that were *para* to both the amine (R_1_-G3XB) and the iodine donors (2R_2_-G3XB). This design permitted systematic modulation of the electron density on both the HB and XB donor rings to test substituent effects. Controls with trifluoromethyl substituents (the strongest electron withdrawing groups in this study) were also prepared without the amine (nHBeXB) and without the iodine XB donors (G3HB).

### Synthesis and characterization

**Scheme 1:**
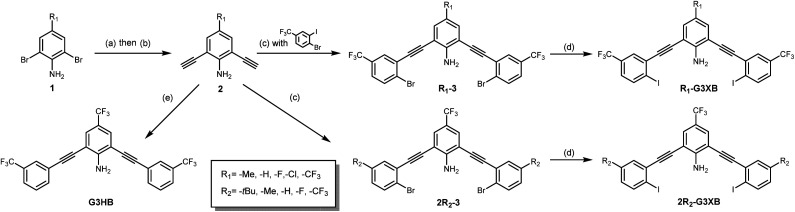
Synthesis of G3XB derivatives and controls used to study HBeXB substituent effects. Reagents and conditions: (a) TMS-acetylene, Pd(PPh_3_)_2_Cl_2_, Cu(i)I, DIPEA, DMF, overnight, N_2_, 80 °C; (b) K_2_CO_3_, MeOH/DCM (1 : 1 v/v), 4 hours, rt, 45–96%. (c) 4-Bromo-3-iodo-R_2_-benzene, Pd(PPh_3_)_2_Cl_2_, Cu(i)I, DIPEA, DMF, overnight, N_2_, rt, 53–87%; (d) NaI, Cu(i)I, *trans-N*,*N*-dimethylcyclohexane-1,2-diamine, 1,4-dioxane, microwave reactor, 12–24 hours, 150 °C, 33–84%; (e) 3-bromotrifluoromethylbenzene, Pd(PPh_3_)_2_Cl_2_, Cu(i)I, DIPEA, DMF, overnight, N_2_, 80 °C, 60%.

The synthesis of G3XB derivatives is outlined in Scheme 1. 2,6-Bis(ethynyl)-4-R_1_-aniline (2) was synthesized by Sonogashira cross-coupling 2,6-dibromo-4-R_1_-aniline (1) with trimethylsilylacetylene followed by removal of the trimethylsilyl protecting groups with potassium carbonate. Precursor scaffolds containing bromine (R_1_-3, 2R_2_-3, and HB receptor G3HB) were synthesized by Sonogashira cross coupling 2 at the iodo-functionality of 4-bromo-3-iodo-R_2_-benzene or 3-bromotrifluoromethylbenzene, respectively. The iodine containing R_1_-G3XB and 2R_2_-G3XB were obtained by microwave assisted halogen exchange of R_1_-3 or 2R_2_-3. The complete experimental procedures can be found in the ESI.†

### Experimental evidence of intramolecular hydrogen bonding

**Fig. 3:**
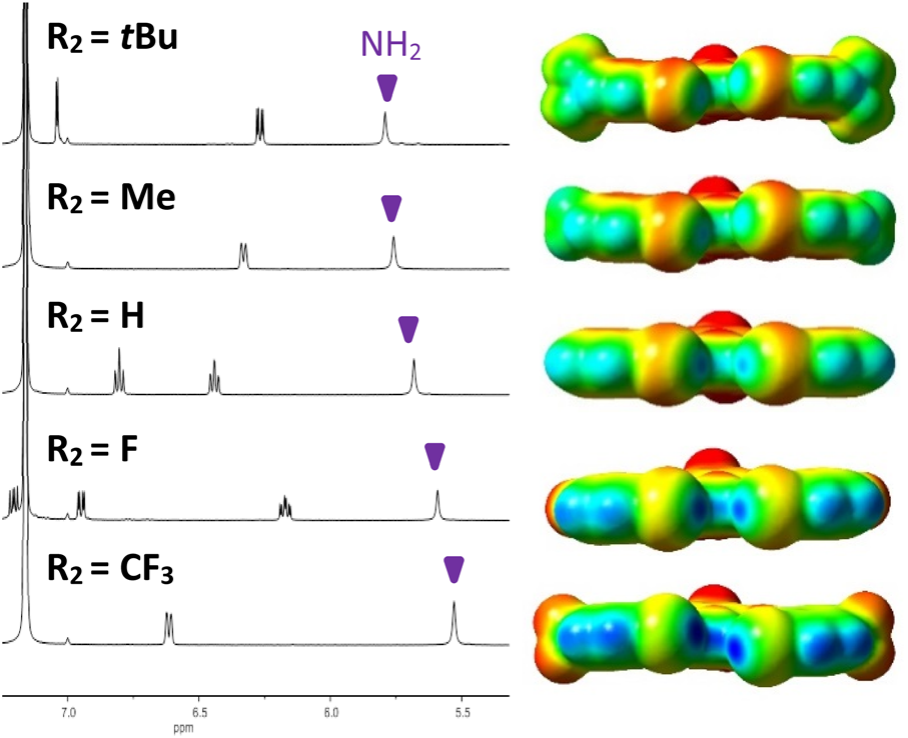
^1^H NMR spectra (C_6_D_6_, 500 MHz, 5 mM) of 2R_2_-G3XB derivatives (left) highlighting the HBing downfield shift of the amine as the halogen becomes more electron rich; ESP maps (isovalue = 0.001 a.u.) for 2R_2_-G3XB derivatives (right) are displayed on the same scale. Electron deficient regions are blue and electron rich regions are red.

Analysis of the amine ^1^H NMR resonances provided initial indication of intramolecular HBing between the amine and the electron rich belt of the XB donor (N–H⋯I). The analysis provided a preliminary evaluation of substituent effects and provided rare experimental evidence of HBing to larger halogens.^24*a*,25^ Control receptor G3HB, lacking XB donors to accept HBs, had an amine ^1^H chemical shift of 4.64 ppm in C_6_D_6_, whereas the chemical shift for G3XB, with iodine acceptors, was 5.53 ppm. This 0.89 ppm downfield shift is indicative of intramolecular HBing. The series of 2R_2_-G3XB derivatives (Fig. 3, left) showed that as the *para* substituent on the XB donor ring became more electron donating, the HBing amine proton shifted downfield from 5.53 ppm to 5.80 ppm. This downfield shift occurs despite the expectation that adding electron donating substituents should shield the nuclei and would produce an upfield shift for the proton. However, the electron donating groups in the 2R_2_-G3XB compounds transfer additional electron density onto the iodine atoms (*vide infra*) making the iodine a better HB acceptor, resulting in the downfield shift.‡‡No clear trend was observed among R_1_-G3XB derivatives. The amine proton peak is 5.53 ppm for R_1_=CF_3_, 5.25 ppm R_1_=Cl, 5.17 ppm R_1_=F, 5.40 ppm R_1_=H and 5.31 ppm R_1_ = Me. The deviation for the fluorine and chlorine substituents might be a result of their π electron donating property affecting the amine. We rationalize the trend observed of 2R_2_-G3XB derivatives based on how the substituents influence the electronics on the HB acceptor (in this case the XB donor) rather than the HB donor. Overall this NMR analysis suggests that the intramolecular HBing is formed and the strength of this HBing correlates with the electron density of the halogens.

## Computational evaluations

To garner preliminary insight into substituent effects of HBeXBs, *in silico* studies were performed. All the receptors were evaluated using Gaussian 16 at the M06-2X/def2TZVPP level of theory (see ESI† for further details). A systematic use of conformational analysis and electrostatic potential (ESP) mapping provided early insight into the synergy between the HB and XB and helped to stimulate deeper LFER analysis.

### σ-Hole calculations

σ-Hole analysis (maximum/minimum electrostatic potentials, denoted by *V*_s,max_/*V*_s,min_) showed several trends regarding the influence of the intramolecular HB on XB donor strength. Consistent with our previous studies, the HB from the amine to the iodine augments the σ-hole (greater *V*_s,max_). For example, G3XB in the bidentate conformation has a *V*_s,max_ that is 4.02 kcal mol^−1^ greater than nHBeXB—a consequence of the HBeXB (Table 1).

**Table 1:** Halogen *V*_s,max_ of G3XB derivatives^a^

	Bidentate	S conformation (HBed iodine/non-HBed iodine)	Δ*V*_s,max_^b^
G3XB	32.05	31.45/24.46	6.99
nHBeXB	28.03	26.64/25.39	1.25
2F-G3XB	28.40	28.52/20.94	7.58
2H-G3XB	24.98	26.05/17.95	8.10
2Me-G3XB	23.03	24.69/16.94	7.75
2*t*Bu-G3XB	22.41	24.37/16.15	8.22
Cl-G3XB	30.84	29.85/24.16	5.69
F-G3XB	30.12	29.33/24.61	4.72
H-G3XB	28.72	28.08/24.07	4.01
Me-G3XB	28.11	27.36/24.04	3.31

a *V*_s,max_ (kcal mol^−1^) were calculated at 0.001 Å isoelectric surface.

b Δ*V*_s,max_ = *V*_s,max_ HBed iodine − *V*_s,max_, non-HBed iodine.

### Substitution on the HB donor ring

Modulation of the R_1_ group helped determine if stronger HBs would correlate to greater XB enhancement. Within the R_1_ series, the *V*_s,max_ values on the XB donors ranged from 32.05 kcal mol^−1^ for the most electron withdrawing G3XB in the bidentate conformation to 28.11 kcal mol^−1^ for Me-G3XB (Table 1). The data verify that stronger HB donors correlate with a more positive *V*_s,max_—confirming a notion alluded to in our previous HBeXB systems.^14*b*,24*a*^ Our results here highlight that tuning the HB donor with a single remote substituent can influence the XB donor *V*_s,max_ by 3.94 kcal mol^−1^.

### Substitution on the XB donor ring

Next, the *V*_s,max_ of the 2R_2_-G3XB derivatives in the bidentate conformation was probed to determine how substituent electronics on the XBing ring influence XB donor strength. As expected, the *V*_s,max_ becomes larger as the substituent *para* to the XB donor increases in electron withdrawing capacity. The G3XB derivative with CF_3_ substituents represents the upper end in this series with a *V*_s,max_ of 32.05 kcal mol^−1^ whereas, 2*t*Bu-G3XB represents the lower end at 22.41 kcal mol^−1^. These studies align with previous reports^18,19,22^ showing that XB donors can be directly influenced by electronic substituent effects. For instance, the *V*_s,max_ of the XB donors in Talyor's 4-R-C_6_F_4_I studies differ by 6.9 kcal mol^−1^ when a *para* fluoro substituent is changed to a piperidyl group.^18^ However, it is notable that in this bisethynyl system the *V*_s,max_ varies by 9.64 kcal mol^−1^ by direct substitution on the XB ring.

In contrast to the above discussion on XB donor strength (*i.e. V*_s,max_), we also considered the *V*_s,min_ of the iodine atoms as a measure of HB acceptor capacity. As the substituent *para* to the XB donor becomes more electron donating, the iodine species becomes more electron-rich (Fig. 3). The *V*_s,min_ of the 2R_2_-G3XB compounds (−1.11 to −11.14 kcal mol^−1^, for details see ESI†) trend with the downfield NMR shifting—suggesting that R_2_ electron donating groups strengthen the intramolecular HBing between the amine and the XB donor.

### Conformational effects on the XB donor

#### R_1_ – minimal through bond effects on the XB donor

**Fig. 4:**
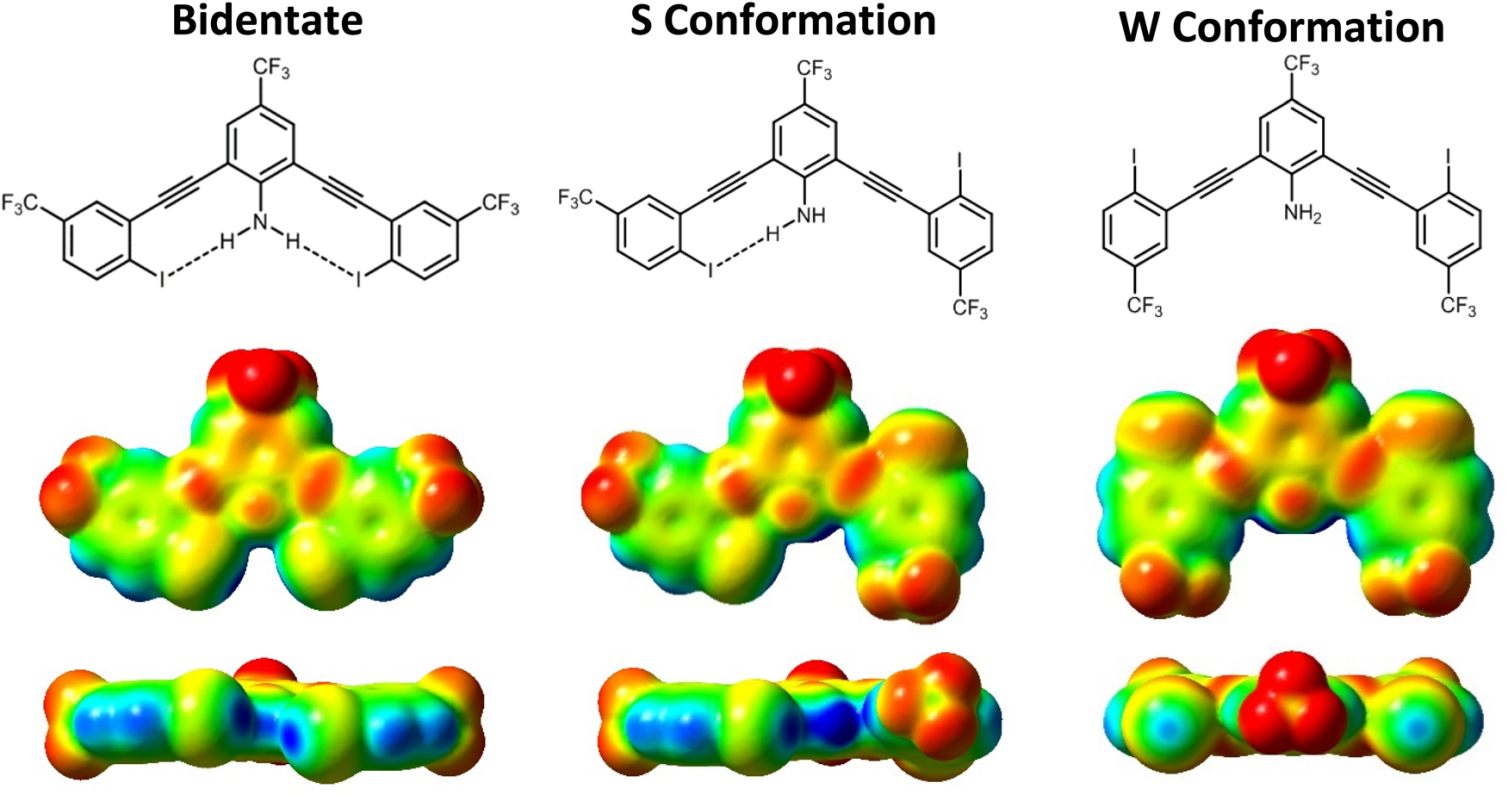
ChemDraws and ESP maps for G3XB in all three planar conformations. The bidentate conformation (left), where both XB donors are convergent; the S conformation (middle), where the XB donors are on opposite sides of the molecule; and the W conformation (right), where both XBs are directed away from the amine. All ESP maps are displayed on the same scale. Electron deficient regions are blue and electron rich regions are red.

ESP maps were calculated for the R_1_-G3XB derivatives in the S conformation (Fig. 4). The S conformation contains one XB donor that accepts a HB and one that does not. This S arrangement was used to determine whether substitution on the HB donor ring has a through bond electronic effect on the XB donor. The S conformation of R_1_-G3XB derivatives all exhibit similar *V*_s,max_ values (≈24 kcal mol^−1^) for the iodine not accepting a HB. This demonstrates that substituents on the central HB donor ring do not directly inductively alter the electronics of the XB donor.§§This finding also correlates with computational data from Scheiner^26^ illustrating that the through bond influence of the electron withdrawing group on the XB properties diminishes with its distance from the halogen atom.

#### R_1_ – through space effects on the XB donor

In contrast, the potency of the HB donor does have an influence on the XB donor strength. The *V*_s,max_ of the halogen accepting a HB has a greater ESP than the non-HB accepting iodine. The *V*_s,max_ values for the iodine in G3XB that accepts a HB was 31.45 kcal mol^−1^ and 27.36 kcal mol^−1^ for Me-G3XB which has the most electron donating substituent R_1_. Thus, strengthening the intramolecular HB donor can modulate the *V*_s,max_ of a single XB donor by 4.09 kcal mol^−1^, a value similar to the bidentate assessment described above.

The through space influence of a HB on the XB donor was analyzed by computing the difference (Δ*V*_s,max_) between the two iodine donors in the S conformation (Δ*V*_s,max_ = *V*_s,max_ HBed iodine − *V*_s,max_ non-HBed iodine). Here the weakest HB donor Me-G3XB derivative had a Δ*V*_s,max_ of 3.31 kcal mol^−1^ while the strongest HB donor G3XB had the largest Δ*V*_s,max_ of 6.99 kcal mol^−1^. Collectively the Δ*V*_s,max_ values of the R_1_-G3XB derivatives adhere to the trend that increasing the electron withdrawing ability of R_1_, strengthens the HB which in turn has a larger influence on the XB donor.

#### R_2_ – through bond effects on the XB donor ring¶¶Comparing the entire 2R_2_-G3XB series, we noted that the *V*_s,max_ of 2Me-G3XB is smaller than 2H-G3XB which deviates from the expected electronic trend. This is likely due to small differences in the planarity of the structures during the calculations that affect the electronic environment of the halogens (see ESI†). For 2R_2_-G3XB derivatives which have more electron donating substituents (R_2_=F, H, Me, *t*Bu), it was observed that the iodine in the S conformation which accepts a HB has a 0.1 to 2.0 kcal mol^−1^ higher *V*_s,max_ than the iodines in the bidentate conformation (which also accept HBs). We hypothesized that the S conformation provides more flexibility for the only N–H⋯I HB thus producing a stronger HB. However, when considering receptor design, this small difference would likely be compensated by allowing two convergent XBs to be available in the bidentate conformation.

*V*_s,max_ values for 2R_2_-G3XB derivatives in the S conformation were used to quantify how changing the electronics of the XB donor ring influences the iodine σ-hole. For example, the *V*_s,max_ of the externally directed iodine (non-HBed iodine) generally followed the trends expected from the electronic contributions of the R_2_ group (*i.e. V*_s,max_ values for 2R_2_-G3XB trended in the order CF_3_ > F > H > Me > *t*Bu) ranging from 24.46 to 16.15 kcal mol^−1^. The internally directed iodine atoms (accepting a HB) all had larger *V*_s,max_ values ranging from 31.45 to 24.37 kcal mol^−1^ and generally followed the same trend.

#### R_2_ – through space effects on the XB donor†

The strength of the HB donor in the 2R_2_-G3XB derivatives is constant (*i.e.* a CF_3_ group *para* to the central amine). Thus, the Δ*V*_s,max_ values here are a measure of how the R_2_ electronics impact the halogen as both a XB donor and a HB acceptor. The Δ*V*_s,max_ values of 6.99, 7.58, 8.10, 7.75 and 8.22 kcal mol^−1^ were obtained for G3XB, 2F-G3XB, 2H-G3XB, 2Me-G3XB and 2*t*Bu-G3XB, respectively. The trend parallels previous evaluations and shows that generally, more electron rich iodines experience a greater augmentation.

Comparing 2R_2_-G3XB and R_1_-G3XB Δ*V*_s,max_ values provides a measure of which substituent position impacts XBing the most by a through space effect. The smaller range of values for the 2R_2_-G3XB series (1.23 kcal mol^−1^), as compared to the R_1_-G3XB derivatives (3.68 kcal mol^−1^), suggests that the influence of HBing on the halogen atom is more sensitive to substitution on the HB donor ring. For example, altering one R_1_ substituent from CF_3_ to Me results in a 3.68 kcal mol^−1^ Δ*V*_s,max_ difference. However, altering two R_2_ substituents from CF_3_ to Me results in a 0.76 kcal mol^−1^ Δ*V*_s,max_ difference.

#### Influence of R_1_ and R_2_ on conformation

To assess the role of preorganization in G3XB derivatives, their relative stabilities were assessed based on electronic energies from DFT. The difference between the S and bidentate conformation energy illustrates that intramolecular HBeXBs stabilize the bidentate conformation of the receptors (Table 2). The bidentate conformation of G3XB contains two intramolecular HBs (N–H⋯I) and is more stable than the S conformation by 1.53 kcal mol^−1^. The W form, lacking intramolecular HBs is 3.11 kcal mol^−1^ higher in energy than the bidentate conformation. For the 2R_2_-G3XB series, as R_2_ becomes more electron donating, the energy differences between the bidentate and S conformation increases from 1.76 kcal mol^−1^ for 2F-G3XB to 2.15 kcal mol^−1^ for 2*t*Bu-G3XB. This suggests a greater stabilization when the iodine HB acceptor is more electron rich. These results track with the amine ^1^H NMR chemical shift analysis for 2R_2_-G3XB. In contrast, the difference between the bidentate and S conformation for the R_1_-G3XB series is comparatively attenuated; however, only a single substituent group is modified. The Δ*E* is 1.42 kcal mol^−1^, 1.33 kcal mol^−1^, 1.43 kcal mol^−1^, and 1.36 kcal mol^−1^ for Cl-G3XB, F-G3XB, H-G3XB and Me-G3XB, respectively. These data indicate that conformational preference is sensitive to the electronics of both the XB and HB donor.

**Table 2:** Electronic energy difference (Δ*E*) between S and bidentate conformations of G3XB derivatives

	Δ*E* (kcal mol^−1^)
G3XB	1.53
nHBeXB	−0.01
2F-G3XB	1.76
2H-G3XB	1.83
2Me-G3XB	1.90
2*t*Bu-G3XB	2.15
Cl-G3XB	1.42
F-G3XB	1.33
H-G3XB	1.43
Me-G3XB	1.36

## Solution studies

### NMR titrations and association constants

^1^H NMR anion binding titrations were performed to quantify HBeXB substituent effects in solution. Titrations were conducted in C_6_D_6_ with tetra-n-hexylammonium iodide (THAI) as the guest to ensure all complexes remained in solution. The addition of THAI resulted in downfield shifts for nearly all of the ^1^H NMR signals on the receptors, (except for a center core singlet of nHBeXB). Bindfit^27^ was used to fit the changes in the ^1^H NMR signals to a 1 : 1 binding model. Iterative and simultaneous refinement of multiple isotherms provided association constants (*K*_a_) for all scaffolds (Table 3).

**Table 3:** Measured association constants and binding energies for G3XB a

Host	*K*_a_ (M^−1^)	Δ*G*_binding_ (kcal mol^−1^)
G3XB	420	−3.6
G3HB	30	−2.1
nHBeXB	10	−1.5
2F-G3XB	170	−3.0
2H-G3XB	70	−2.5
2Me-G3XB	50	−2.3
2*t*Bu-G3XB	50	−2.3
Cl-G3XB	330	−3.4
F-G3XB	250	−3.3
H-G3XB	190	−3.3
Me-G3XB	170	−3.1

a The *K*_a_ values are reported as the average of three titration experiments. All titrations were performed in C_6_D_6_; two significant figures are reported and errors are estimated at 10%. Tetra-n-hexylammonium iodide was used and titrations were performed at 25 °C. Bindfit was used to fit changes in chemical shift to a stepwise 1 : 1 host–guest binding model. The free energy of binding (Δ*G*_binding_) was calculated from the association constant.

### Role of intramolecular HBing on anion binding

G3XB (all R groups –CF_3_) had the strongest binding (420 M^−1^) which was nearly 14 times greater than the isostructural no XB control (G3HB) (30 M^−1^). The substantially lower binding affinity of G3HB suggests that the amine doesn't significantly HB to the iodide guest; the amine of the G3XB derivatives largely forms intramolecular HBs with the iodine XB donors. The considerable influence of the intramolecular N–H⋯I HBs is evident when comparing G3XB to the control lacking an amine (nHBeXB). nHBeXB exhibited very weak binding in solution with a *K*_a_ = 10 M^−1^. The nearly 50-fold difference in *K*_a_ between G3XB and nHBeXB demonstrates the striking impact that a weak intramolecular N–H⋯I HB can have on receptor performance. The HBeXB enhancement is far greater than our original HBeXB study using a dicationic receptor where only a 9-fold increase was observed.^14*b*^ The greater HBeXB influence in this study could be due to the iodine XB donors of G3XB being more electron rich (*i.e.* neutral receptor) than the iodopyridinium donors previously evaluated—allowing for stronger HBeXB and greater preorganization. It could also be attributed to solvent effects as the two studies were conducted in significantly different media (C_6_D_6_*vs.* 60% CD_3_NO_2_/40% CDCl_3_). These binding studies highlight that the central amine interacts minimally with the iodide and largely operates as an intramolecular HB donor to the iodine XB donor atoms.

### 2R_2_-G3XB substituent effects on anion binding

The 2R_2_-G3XB (R_2_=CF_3_, F, H, Me, *t*Bu) series of molecules were used to quantify how substituents *para* to the XB donor influence the HBeXB. Varying these substituents resulted in association constants for THAI ranging from 50 M^−1^ to 420 M^−1^ (Table 3). Having a stronger electron withdrawing group *para* to the XB donor increases the XB strength and for this series of compounds this generally holds true. G3XB (R_2_=CF_3_) maintained the greatest affinity followed by 2F-G3XB with a *K*_a_ = 170 M^−1^, which is 60% less than G3XB. 2H-G3XB binding was further diminished to a *K*_a_ of 70 M^−1^. The most electron rich 2*t*Bu-G3XB exhibited similar iodide binding with the 2Me-G3XB derivative (50 *vs.* 50 M^−1^, respectively). Nevertheless, the general trend in substituent effects matches previous studies for XB derivatives.^18–22^

### R_1_-G3XB substituent effects on anion binding

While studies have evaluated the influence of functional groups on the XB and HB independently none have considered their interplay. Here binding studies of R_1_-G3XB derivatives represent the first experimental consideration of this.

The binding data for R_1_-G3XB derivatives (R_1_=CF_3_, Cl, F, H, Me) highlights that stronger intramolecular HBing enhances the XB receptor binding affinity. By increasing the electron withdrawing capacity of the substituent *para* to the amine (strengthening the HB donor) from a Me group to a CF_3_ group the binding increased 2.5-fold. Me-G3XB, the most electron rich of the R_1_-G3XB series, had the lowest association constant (170 M^−1^), while the most electron deficient G3XB had the highest (420 M^−1^). This result reveals an effective way to increase the overall XB binding ability in a system which includes an intramolecular HB to XB donor—electronically tuning the HB with substituents rather than the XB.

### R_1_ and R_2_ interplay: receptor performance

As noted above, the receptor performance can be modulated by either changing the substituents *para* to the XB donor or *para* to the HB donor. To further quantify the substituent effects on binding Δ*G*_binding_ was calculated for each receptor. The binding energy for 2R_2_-G3XB derivatives can be tuned by 1.3 kcal mol^−1^ simply by changing out the two CF_3_ groups to Me groups on the XBing rings. Intriguingly, altering only one substituent (from CF_3_ to Me) on the center ring can elicit a 0.5 kcal mol^−1^ change. This suggests that binding can be modified by a comparable amount with a smaller structural change to the receptor. These small energetic changes can have large implications, as previously demonstrated in a study of XB catalyst transition state binding.^28^

### R_1_ and R_2_ interplay: linear free energy relationships

**Fig. 5:**
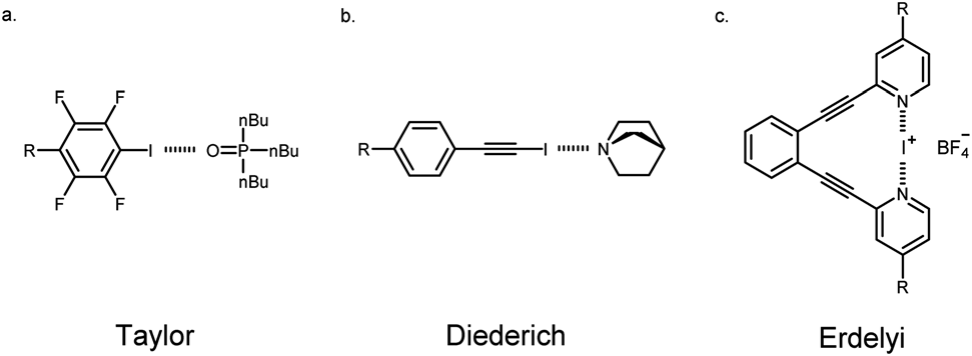
Selected examples of systems that were used to study halogen bonding LFER.

Linear free energy relationships (LFERs) are gaining importance in understanding substituent effects on noncovalent interactions like HBing, XBing, chalcogen bonding, cation–π and π–π.^17,29^ There are surprisingly few studies that have experimentally examined substituent effects by evaluating LFERs on the XB.^18–22^ Taylor and coworkers adeptly used this approach to evaluate the XB between *para*-substituted tetrafluoro-iodobenzene and tributylphosphine oxide (Fig. 5a). These studies showed the best correlation of association constants with the *σ*_meta_ parameter (*R*^2^(*σ*_m_) = 0.94 *vs. σ*_para_*R*^2^(*σ*_p_) = 0.82) which they attributed to inductive/field effects being more dominant.^18^ In contrast, Diederich^19^ and Franz^22^ interestingly reported strong correlation for the *σ*_para_ parameters. Diederich evaluated XBing between 4-R-iodoethynylbenzene and quinuclidine (*R*^2^(*σ*_p_) = 0.97 *vs. R*^2^(*σ*_m_) = 0.82, Fig. 5b). The strong correlation with the *σ*_para_ parameter in this case indicated that substituents largely influence the halogen donor through resonance in this conjugated system. Finally, Erdélyi^20^ and Stilinović^21^ investigated substituent effects on XB acceptors. In Erdélyi's case, the three-center [N–I–N]^+^ XB (Fig. 5c) exhibited linear correlation (*R*^2^ = 0.97) between the calculated natural atomic populations with *σ*_para_ parameters. In all cases, substituent effects were shown to have a significant influence on XBing. Nevertheless, each of these examples showed great correlation with different parameters for their LFERs. Despite these important studies, there are no experimental examples analyzing substituent effects on adjacent noncovalent interactions. Supramolecular contacts don't occur in an isolated environment and as such, it is essential to understand whether traditional substituent effects hold true in these situations. Herein, we evaluated substituent effects on the HBeXB and used LFERs to establish whether changing the electronics of the HB donor has the same influence on the overall binding as changing the electronics of the XB donor.

Our initial evaluation of LFERs compared the bidentate ESP values (*V*_s,max_) of receptors with experimental Δ*G* values from titration studies. While XBing is known to encompass both covalent and electrostatic components,^9^ plots of ESP *vs.* Δ*G* can establish the degree of electrostatic contribution in these particular HBeXB complexes. The following results demonstrate that in this system, the substituent influence is largely electrostatic in nature.

**Fig. 6:**
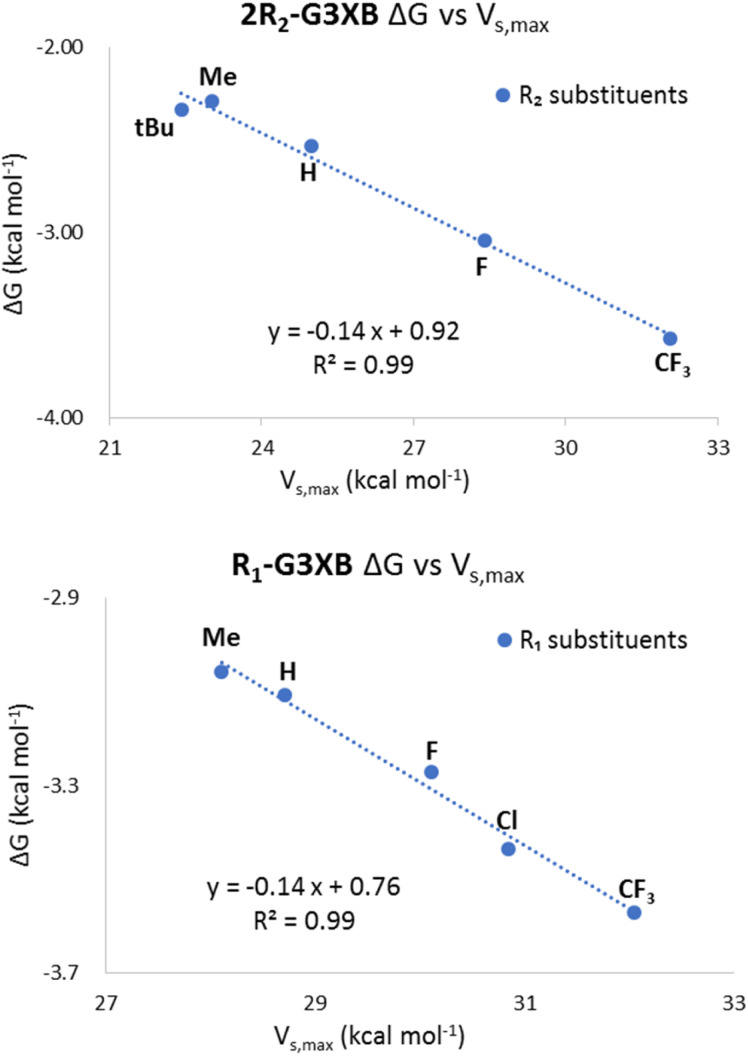
Non-normalized plots of the ESPs and binding energies for 2R_2_-G3XB (top) and R_1_-G3XB (bottom).

The 2R_2_-G3XB derivatives show strong correlation between the electrostatics (*V*_s,max_) of the XB donor and the iodide binding in solution. The non-normalized plots (Fig. 6, top) are linear for the 2R_2_-G3XB derivatives (*R*^2^ = 0.99). Since the modification was directly on the XBing ring, this finding was expected given previous LFER studies on the XB.^18^

In contrast, the R_1_-G3XB derivatives provided original insight into how the amine HB donor augments the XB donor strength. The LFER analysis for the R_1_-G3XB series certainly suggests an electrostatic origin (Fig. 6, bottom) as the plots are again linear (*R*^2^ = 0.99). Collectively the ESP *vs.* Δ*G* plots indicates that the HBeXB iodide binding of the G3XB derivatives is largely governed by electrostatics and that ESP maps accurately model the influence of intramolecular HBs on the XBs in this system.

We extended our LFER analysis by evaluating the correlation between our experimental association constants and Hammett parameters (*σ*_meta_ and *σ*_para_). While Hammett parameters were originally used to model the ionization reaction of benzoic acid, they have been increasingly used to study noncovalent interactions.^6–8^ We used Hammett parameters to analyze possible inductive (or field effects as proposed by Wheeler and Houk^30^) and resonance effects on the HBeXB receptor binding—an approach recently used by Hunter to effectively assess HB cooperativity.^31^

Normalized association constants (log(*K*_r_/*K*_H_)) and the corresponding substituent parameters (*σ*) were fit using the Hammett equation shown below. *ρ* represents the slope.log(*K*_R_/*K*_H_) = *ρσ*

**Fig. 7:**
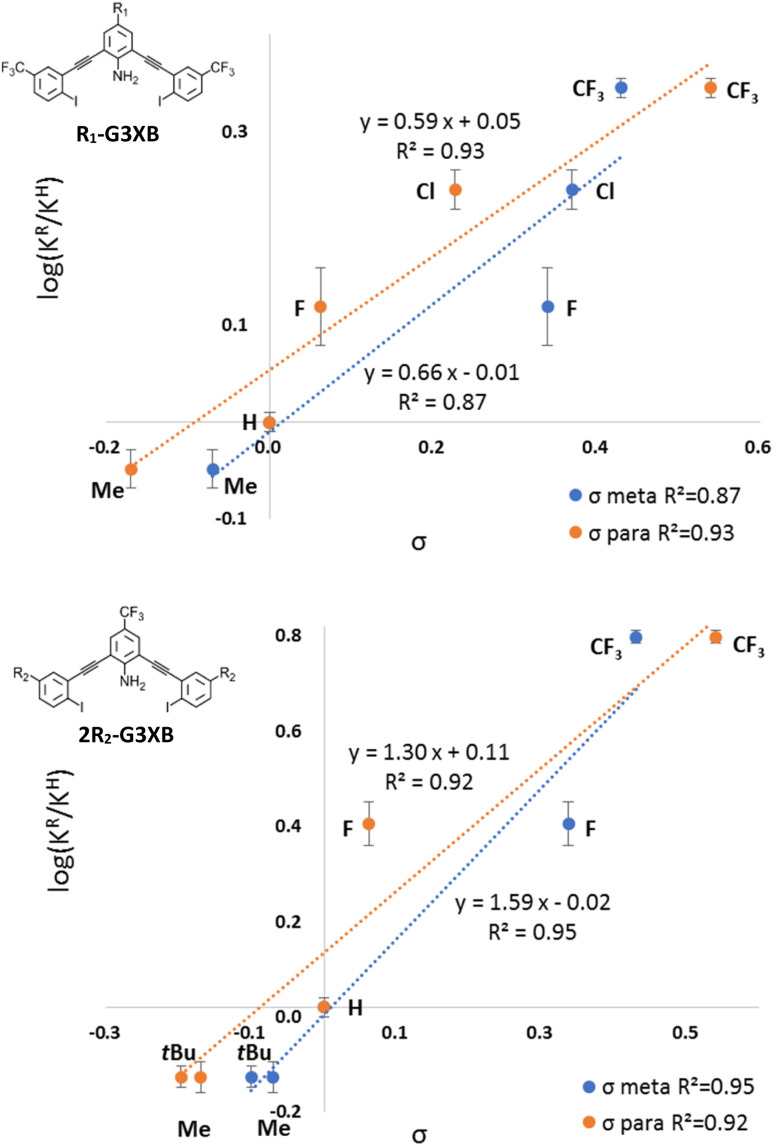
Normalized Hammett plots of *K* values of R_1_-G3XB (top) and 2R_2_-G3XB (bottom) with *σ*_meta_ (blue) and *σ*_para_ (orange).

The R_1_-G3XB derivatives show a more linear correlation with the *σ*_para_ parameter (*R*^2^(*σ*_p_) = 0.93) than with the *σ*_meta_ (*R*^2^(*σ*_m_) = 0.87), indicating resonance effects are more important than inductive effects on the HB donor of the HBeXB (Fig. 7, top). Electron withdrawing R_1_ substituents enhance the amine donor strength, thus leading to stronger intramolecular N–H⋯I HBing. A stronger HB in turn makes the XB donor more electron deficient. Thus, the resonance of the R_1_ substituent work in concert with both the HB and XB donors, resulting in a linear correlation with the *σ*_para_ parameters.

Curiously, normalized Hammett plots of *K*_a_ values for 2R_2_-G3XB resulted in similar linear fits with both the *σ*_meta_ and *σ*_para_ parameters (Fig. 7, bottom). The fit with the *σ*_meta_ parameters is *R*^2^(*σ*_meta_) = 0.95 while the fit with the *σ*_para_ parameters is *R*^2^(*σ*_para_) = 0.92, implying that inductive effects may play a modestly more important role on substituent effects. As noted above, substituent effects on the XB have been found to be attributed to either inductive^18^ or π-resonance effects.^19,20^ It is atypical in LFER studies to obtain linear fits for both the *σ*_para_ and the *σ*_meta_ parameters simultaneously.^32^ Unlike previous LFER studies, in this case there are two noncovalent interactions involved (N–H⋯I HBing and C–I⋯I^−^ XBing). The halogen here functions as both a XB donor and a HB acceptor. The electronics of the R_2_ substituents can have competing influences on the noncovalent interactions. Thus, increasing the electron density on the halogen should weaken the XB donor but strengthen the intramolecular HBs with the amine. This competing effect produces good correlation with both the *σ*_para_ and the *σ*_meta_ parameters. Thus, when competing electronic influences are present, selecting an appropriate *para* substituent for a XB donor could be tricky if relying on Hammett parameters. We also looked for linear correlations between the association constants and other parameters including Taft's *σ*_I_, *σ*_R_,^6*d*^ sEDA and pEDA.^33^ However, no linear correlations were obtained (see ESI†). The combined LFERs herein, suggest that choosing an appropriate substituent (R_1_) to tune adjacent noncovalent interactions could complement the common strategy of directly altering the electronics of the XB donor (R_2_) to modulate binding.

Classically, the slope *ρ* in the Hammett equation describes the susceptibility of the reaction to substituents. While studying noncovalent interactions, *ρ* provides a measure of how sensitive the interaction is to substituent effects as compared to the ionization of benzoic acid. The *ρ* value of the R_1_-G3XB Hammett plot (*ρ*R_1_ = 0.59) is between 0 and 1 indicating that the binding is sensitive to electronics (although not as much as the ionization of benzoic acid). More importantly, the *ρ* value for R_1_-G3XB can be compared to 2R_2_-G3XB (statistically taking into consideration the number of substituents); thereby, determining quantitatively which substituent position has a greater influence on receptor performance. There are two R_2_ groups affecting the anion binding per receptor, so half the *ρ* value was used for comparison. The 1/2*ρ* value for R_2_-G3XB (1/2*ρ*R_2_ = 0.79) is only modestly higher than the one obtained for the R_1_-G3XB series (*ρ*R_1_ = 0.59). This is notable as the R_1_ substituents are much further from the binding site and maintain minimal through bond electronic influence on the XB. The data here indicate that altering the electronics of an HB donor within the context of the HBeXB can have a similar effect on XB strength as traditional substituent effects.

For a more thorough picture of how resonance contributes to the interaction, multivariable linear regression of the normalized association constants (log(*K*_r_/*K*_H_)) with the field (*F*) and resonance (*R*) parameters in the Swain–Lupton equation^5*b*^ were conducted in Matlab. The percent resonance contribution (%*R*) in this equation affords a simple and meaningful way to assess the relative importance of field (*F*) and resonance (*R*) effects.log(*K*_X_/*K*_H_) = *ρ*_f_*F* + *ρ*_r_*R* + *i*

%*R* = *ρ*_r_/(*ρ*_f_ + *ρ*_r_) × 100

As shown in Table 4, %*R* = 49% for 2R_2_-G3XB which suggests that the binding is slightly more governed by inductive/field effects from the substituents. The %*R* for 2R_2_-G3XB reveals the similarity in the impacts of resonance and inductive/field effects on the XB donor ring's R_2_ substituents. This similarity aligns with the 2R_2_-G3XB Hammett plots, which exhibit linear relationships with both the *σ*_para_ and *σ*_meta_ parameters due to the competing influences from the electronics of the R_2_. In contrast, the substituent effects in R_1_-G3XB are more dependent on resonance where %*R* = 54%. However, it should be noted that the Hammett studies employed used relatively few parameters which limits the statistical strength of this Swain Lupton analysis.

**Table 4:** Field and resonance fitting parameters

	*ρ*_f_	*ρ*_r_	*R* ^2^	%*R*
2R_2_-G3XB	0.95	0.90	0.99	49%
R_1_-G3XB	0.37	0.44	0.99	54%

### Crystal structures

#### HBeXB impact on solid-state features

Crystal structures of several receptors were obtained to further evaluate substituent effects. While previous studies have evaluated the HBeXB in the solid-state,^11*a*,14*b*,23*a*,24*a*^ the HBeXB bidentate receptors reported here are the first structures considered the structures within the context of substituent effects.

The initial solid-state assessment of the neutral receptors focused on three species (G3XB, G3HB, nHBeXB) to identify the influence of the HBeXB. Previous generations were charged and thus in the solid state could be more influenced by induced fit binding. This series further confirms that the amine HBs promote a bidentate conformation.

**Fig. 8:**
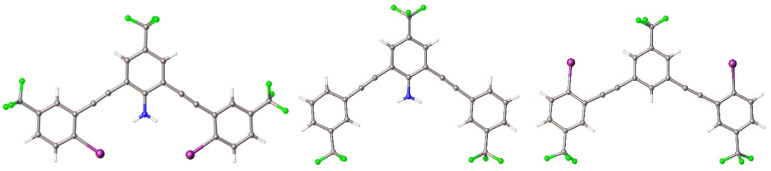
Crystal structures of G3XB molecule (left), G3HB (middle)*, and nHBeXB (right) highlighting the importance of the intramolecular HB to produce the bidentate conformation. * Representative example from the orthorhombic polymorph.

As shown in Fig. 8 (left), the G3XB structure displays convergent bidentate XBing conformations promoted by the intramolecular HBing with N–H⋯I distances and angles of 3.12(4) Å, 163(3)° and 3.20(3) Å, 165(4)°. G3XB crystalized in the monoclinic space group *P*2_1_/*c* with a single molecule in the asymmetric unit. The XB donors are directed towards an iodine atom of an adjacent molecule. One of the iodine atoms forms a XB with C–I⋯I distances and angles of 3.9379(6) and 167.38(11)° (*R*_II_ = 0.97).^34^ The other halogen while directed at an iodine, is too far to XB with a C–I⋯I distance of 4.2007(11) and 173.56(9)°.

Notably, the exchange of the central amine group for a CH proton results in a molecule that adopts the W conformation (Fig. 8 right), the form energetically favoured in the computational analysis. nHBeXB crystalizes in *P*2_1_/*c* with a single molecule in the asymmetric unit. One of the iodine atoms forms a XB with a symmetrically equivalent species on an adjacent molecule (C–I⋯I of 160.91(16)° and 3.8065(6) Å (*R*_II_ = 0.93)). The other iodine has type II halogen contacts^35^ with disordered fluorine atoms of an adjacent molecule, with contacts that are less than the sum of the van der Waals radii.

Two crystal structures were obtained of G3HB, a triclinic and orthorhombic polymorph. Neither structure adopted a bidentate conformation. The triclinic (*P*1̄) form has a single molecule in the asymmetric unit, and does not adopt one of the planar forms (*i.e.* bidentate, S, or W). The central amine does not play a significant role in the packing. The orthorhombic (*Fdd*2) structure of G3HB has two molecules in the asymmetric unit. While each molecule adopts a W conformation, one molecule is much less planar than the other. The distortion of one species in the asymmetric unit may come from the arms having to deflect to maintain a head-to-tail HBing chain.

The systematic changes of XB donors and HB donors in the G3XB, G3HB, nHBeXB series highlights that the N–H⋯I intramolecular HBing plays a key role in the receptor adopting the bidentate conformation. This is further demonstrated in the structures of derivatives containing different substituents on the flanking iodine containing arms.

#### 2R_2_-G3XB in the solid state

To further consider the interplay between the XB and HB we obtained crystal structures of species that modulate the electron density on the XB donating arms. Altering the functional group *para* to the iodine donor permitted us to evaluate two different features related to HBeXB within this system. First, the ability of the N–H⋯I HBing to bias the bidentate conformation was evaluated. Second, the first solid state evaluation of substituent effects with relation to the HB acceptor capacity of iodine donors was investigated.

Despite great efforts, only G3XB and 2H-G3XB were crystallized from the 2R_2_-G3XB series. As expected, each of the molecules in this series maintains intramolecular N–H⋯I HBs and adopt bidentate conformations. 2H-G3XB crystalizes in the monoclinic *P*2_1_ space group with two molecules in the unit cell. The 2H-G3XB molecules have HB parameters of 3.16(4) Å 164(4)°, 3.00(7) Å, 170(7)°, 3.16(7) Å 165(5)°, and 2.99(6) Å 169(6)°, that are shorter than G3XB.

The planarity of the molecules did not follow the expected trend. For example, despite having shorter HB contacts 2H-G3XB was more distorted than G3XB. Angles between flanking rings and central rings of the two unique 2H-G3XB species were 1.68° and 15.54° for one receptor and for the other was 4.66° and 17.53°. A partial explanation for this is that the two structures have different long range packing features (see ESI Fig. S90†).

The angle formed by the centroids of the three rings (*i.e.* flanking-core-flanking angle) provides another structural measurement that indicates HBing strength between the amine hydrogen and more electron rich iodine atoms. For example, a smaller angle would suggest a stronger attraction distorting the alkyne bonds linking the core to the arms. Comparing G3XB, and 2H-G3XB, the increasing electron density on the iodine led to greater distortion with angles of 118.94° for G3XB*vs.* 117.18° for 2H-G3XB.

#### R_1_-G3XB in the solid state

Several R_1_-G3XB derivatives (Me-G3XB, F-G3XB, and G3XB) that modulate the electron density of the central amine were also crystalized to evaluate the effects that these substituents have on the solid-state structures.

Similar to the 2R_2_-G3XB series, initial analysis compared the HB distances. G3XB maintained the shortest N–H⋯I HB contacts (see above for distances and angles) as suggested from the electron withdrawing nature of the CF_3_ group. In contrast, the longest N–H⋯I HB contacts came from the Me-G3XB derivative. This methyl species crystalized in the monoclinic space group *P*2_1_/*c* with a single receptor in the asymmetric unit that adopts the bidentate conformation. The N–H⋯I HB contacts were 3.16(6) Å, 154(7)°, and 3.48(5) Å, 148(5)°. The longer HB contacts are attributed to the electron donating nature of the methyl group.

Unfortunately, F-G3XB adopted the S conformation making comparisons across the series irrelevant. However, comparison of the bidendate G3XB and Me-G3XB suggests that the electronics of the aniline core may have some structural impact on the receptor. For example, the weaker HBs of Me-G3XB resulted in a less planar receptor. For Me-G3XB the angles between the planes of the core and flanking arms were 5.29° and 8.75° as compared to G3XB which was 1.06° and 8.59°. Another parameter evaluated was the angle formed by the centroids of the three rings. For the electron rich Me-G3XB this angle is 123.16° whereas for G3XB the angle is 118.94°. This suggests that the amine donors are stronger in G3XB causing a slight distortion due to the stronger HBs between the amine hydrogen atoms and the iodine atoms.

#### G3XB and derivatives as cocrystal-salts

To probe receptor binding in the solid-state the various derivatives were crystalized with tetraalkylammonium chloride salts. Despite our efforts only four successful cocrystal-salts were obtained (G3XB·Cl^−^, 2H-G3XB·Cl^−^, 2Me-G3XB·Cl^−^, and H-G3XB·Cl^−^). Unfortunately, the different tetraalkylammonium salts present and the crystal packing differences, made comparisons difficult (see ESI†). Despite this, all the structures maintain a bidentate conformation. Additionally, the XB contacts are quite strong with reduction ratios ≤0.85.

## Conclusions

In this work, we reported the first LFER studies for substituent effects on the HBeXB interaction. Electrostatic surface potentials (ESP) were used to assess the electrostatic contribution to the interaction. A strong correlation between computational ESP values and solution binding data illustrated the electrostatic nature of this cooperative interaction. Hammett plots constructed with iodide association constants for R_1_-G3XB and 2R_2_-G3XB showed that the electronics of both the HB and XB are critically important to the binding. Resonance effects of electron withdrawing R_1_ substituents strengthened both the HB and XB and enhanced the overall HBeXB binding. Electron withdrawing groups of R_1_ substituents generated a more potent HB donor which better polarized the XB and further promoted the bidentate conformation. In contrast, the electronics of the R_2_ substituents had competing effects on the HB and XB. Specifically, electron donating groups *para* to the iodine atoms (R_2_) decreased the XB donor ability but made the halogen a better HB acceptor. From a design standpoint, this implies that when modulating electron density on the halogen one can enhance the preorganization of a receptor by increasing electron density on the halogen (improved HB acceptor capacity) at the expense of a slightly weakened XB. Our X-ray crystallography studies further demonstrated the role of the HBeXB on preorganizing molecular structure. Combined, the solution experiments, computations and crystallography provided a rare example of how substituents affect proximal noncovalent interactions. The results from this study also provides important insights for the design of receptors or catalysts—altering remote substituents which electronically influence adjacent noncovalent interactions (instead of direct *para* substitution) can have similar impact to traditional substituent effects and should be considered for molecular design.

## Data availability

The corresponding data can be found in the ESI.†

## Author contributions

JS and OBB conceptualized the project. JS and EAJ conducted synthesis and characterization. VSB conducted computational studies. JS conducted the solution studies. DAD conducted solid-state studies. JS and DAD wrote the paper. OBB supervised the investigation and provided editorial assistance during manuscript preparation. All authors examined the data, results, and conclusions presented here.

## Conflicts of interest

There are no conflicts to declare.

## Supplementary Material

SC-014-D3SC02348F-s001

SC-014-D3SC02348F-s002
