# Supplementary material for: The interplay between hydrogen and halogen bonding: substituent effects and their role in the hydrogen bond enhanced halogen bond
Source: Chem Sci. 2023 Jul 21;14(33):8924–35. doi: 10.1039/d3sc02348f (PMC10445465; doi:10.1039/d3sc02348f)
Supplement: SC-014-D3SC02348F-s001 [file SC-014-D3SC02348F-s001.pdf]

Electronic Supplementary Material (ESI) for Chemical Science.
This journal is © The Royal Society of Chemistry 2023

The Interplay Between Hydrogen and Halogen Bonding: Substituent Effects and their role in the Hydrogen Bond Enhanced Halogen Bond

Jiyu Sun[a], Daniel A. Decato[a], Vyacheslav S. Bryantsev[b], Eric A. John[a], and Orion B. Berryman[a]*

a. Department of Chemistry and Biochemistry, University of Montana, 32 Campus Drive, Missoula, MT, 59812, USA.
b. Chemical Sciences Division, Oak Ridge National Laboratory, Oak Ridge, TN, 37830, USA.

Table of Contents

General methods S2

Synthetic Procedures and Characterization S2-S79

NMR Titration Details and Data S79-S116

Linear free energy relationships S116-S117

Computations S117-S155

X-ray diffraction data S155-S163

Methoxy derivative S162

References S163

General Methods

All reagents were obtained from commercial sources and were used without further purification unless otherwise noted. Thin layer chromatography (TLC) was performed using normal-phase silica gel glass-backed plates (0.25 mm, F-254, SiliCycle) and observed under UV light. Flash column chromatography was performed using normal-phase silica gel (230–400 mesh, SiliaFlash®P60, SiliCycle). Activated molecular sieves were used when anhydrous solvents were required. All compounds were dried in vacuo at room temperature as needed. High-resolution mass spectrometry was carried out using an Agilent 6520 Accurate-Mass Q-TOF LC/MS. Elemental analysis results were obtained from Micro Analysis, Inc. Nuclear magnetic resonance (NMR) spectra were obtained with a VNMRS Varian 500 MHz or a Bruker Avance 400 MHz or an Agilent 400 MHz spectrometer. Chemical shifts are reported in parts per million (ppm) from high to low frequency using the residual solvent peak as the internal reference ($CHCl_3$= 7.26 ppm or C_6H_6= 7.16 ppm). For the ^{19}F NMR spectra hexafluorobenzene C_6F_6 (δ –164.9 ppm) was used as an internal standard. Signal splitting patterns are indicated as s, singlet; d, doublet; t, triplet; m, multiplet, b, broad. Coupling constants (J) are given in Hz.

Synthetic Procedures and Characterization

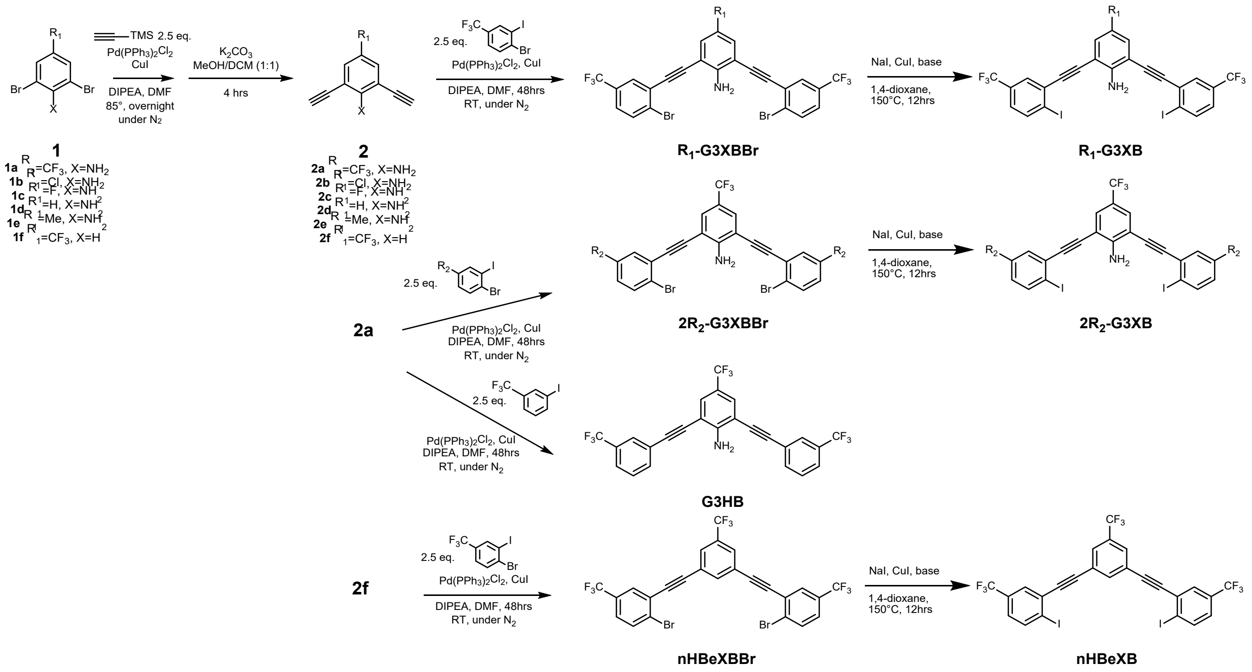

Figure S1. ChemDraw schematic of all **G3XB** derivatives molecules.

2,6-bisethynyl-4-(trifluoromethyl)aniline (2a). To an oven dried Schlenk flask was charged with 2,6-dibromo-4-(trifluoromethyl)aniline (**1a**)(0.638 g, 2 mmol), then vacuumed and backfilled with dry N_2 gas (3x). Bis(triphenylphosphine)palladium(II) dichloride (0.07 g, 0.1 mmol) was added, vacuumed and backfilled with dry N_2 (3x). Copper (I) iodide (0.038 g, 0.2 mmol) was added, vacuumed, and backfilled with dry N_2 (3x). The dry reagents were dissolved in 60 mL dry dimethylformamide (DMF). N,N-diisopropylethylamine (1.74 mL, 10 mmol) and TMS-acetylene (0.692 mL, 5 mmol) were added to the DMF solution. The flask was carefully vacuumed and backfilled with dry N_2 (3x). The dark brown solution stirred overnight at 85°C. The reaction

mixture was run through a silica plug with a hexane/ethyl acetate solvent mixture (50:50) to remove any excess salts and catalysts. Subsequent removal of DMF, hexanes and ethyl acetate by rotary evaporation left a brown liquid that was used directly in the next step with no purification. The brown liquid was dissolved in 50 mL methanol and 50 mL DCM in a 250 mL round bottom flask. Potassium carbonate (0.69 g, 0.5 mmol) was added to the organic mixture. The reaction stirred vigorously for 4 hours at room temperature and reaction progress was monitored via TLC. Upon completion, water was added to quench the reaction. The crude product was extracted with ethyl acetate, dried over magnesium sulfate and gravity filtered. The organic mixture was reduced under vacuum and crude product was purified by column chromatography (gradient from 5% EtOAc/95% Hexanes to 30% EtOAc/70% Hexanes) to afford a dark yellow solid (0.343 g, 85 % yield).

^{1}H NMR (400 MHz, CDCl$_3$): δ (ppm) = 7.55 (s, 2H), 5.17 (s, 2H), 3.46 (s, 2H).

^{13}C NMR (125 MHz, CDCl$_3$): δ (ppm) = 152.38, 130.42 (q, J = 3.8 Hz), 123.88 (q, J = 269.3 Hz), 119.28 (q, J = 33.5 Hz), 106.19, 84.23, 78.78.

^{19}F NMR (470 MHz, CDCl$_3$): δ (ppm) = –59.86.

Elemental analysis, Found: C, 60.7; H, 2.66; N, 6.70%. Calc. for $C_{11}H_6F_3N$: C, 63.1; H, 2.89; N, 6.55%.

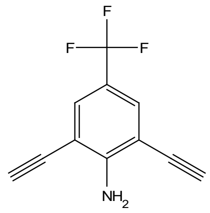

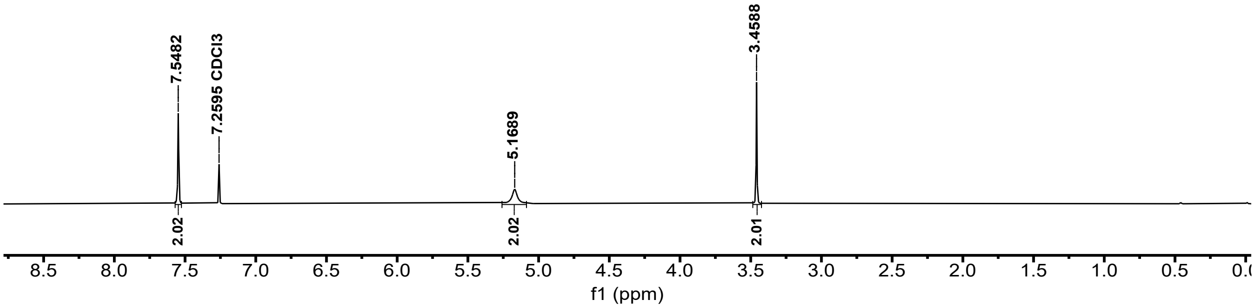

Figure S2. ^{1}H NMR of **2a** (400 MHz, $CDCl_3$).

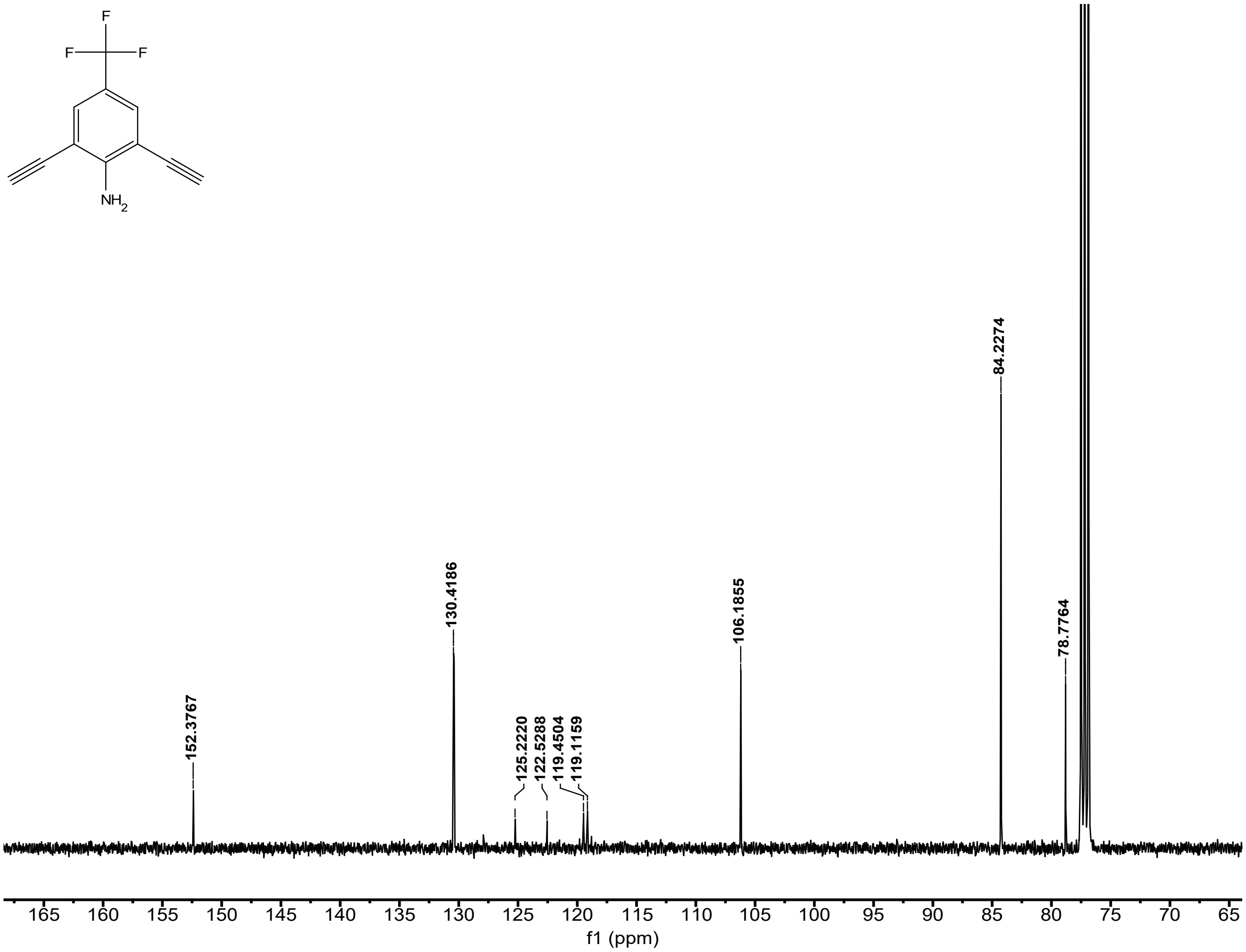

Figure S3. ^{13}C NMR of **2a** (100 MHz, $CDCl_3$).

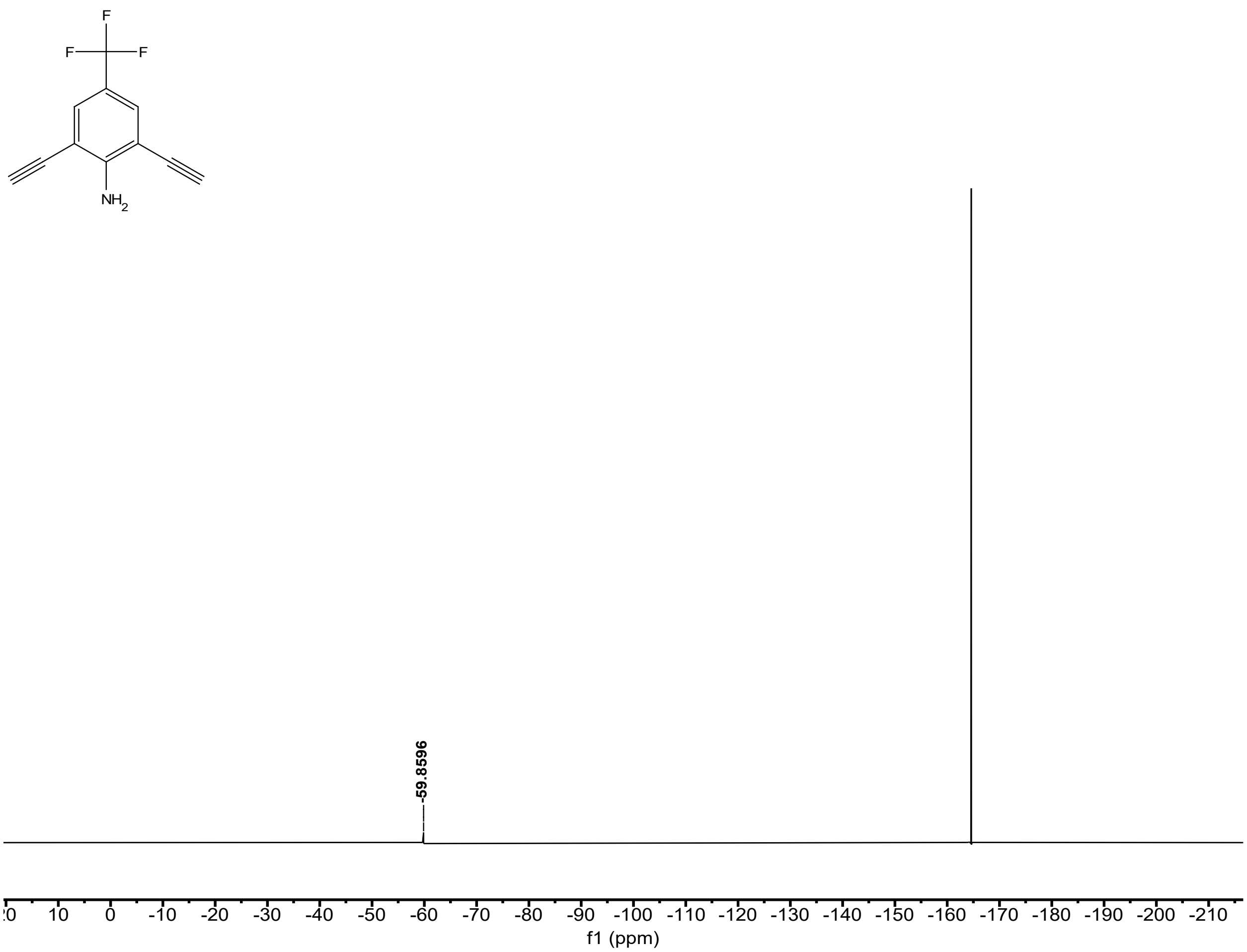

Figure S4. ^{19}F NMR of **2a** (376 MHz, $CDCl_3$). Hexafluorobenzene (C_6F_6) internal reference.

2,6-bisethynyl-4-chloroaniline (2b). To an oven dried Schlenk flask was charged with 2,6-dibromo-4-chloroaniline (**1b**)(0.571 g, 2 mmol), then vacuumed and backfilled with dry N_2 gas (3x). Bis(triphenylphosphine)palladium(II) dichloride (0.07 g, 0.1 mmol) was added, vacuumed and backfilled with dry N_2 (3x). Copper (I) iodide (0.038 g, 0.2 mmol) was added, vacuumed, and backfilled with dry N_2 (3x). The dry reagents were dissolved in 60 mL dry dimethylformamide (DMF). N,N-diisopropylethylamine (1.74 mL, 10 mmol) and TMS-acetylene (0.692 mL, 5 mmol) were added to the DMF solution. The flask was carefully vacuumed and backfilled with dry N_2 (3x). The dark brown solution stirred overnight at 75°C. The reaction mixture was run through a silica plug with a hexane/ethyl acetate solvent mixture (50:50) to remove any excess salts and catalysts. Subsequent removal of DMF, hexanes and ethyl acetate by rotary evaporation left a brown liquid that was used directly in the next step with no purification. The brown liquid was dissolved in 50 mL methanol and 50 mL DCM in a 250 mL round bottom flask. Potassium carbonate (0.69 g, 0.5 mmol) was added to the organic mixture. The reaction stirred vigorously for 2 hours at room temperature and reaction progress was monitored via TLC. Upon completion, water was added to quench the reaction. The crude product was extracted with ethyl acetate, dried over magnesium sulfate and gravity filtered. The organic mixture was reduced under

vacuum and crude product was purified by column chromatography (gradient from 5% EtOAc/95% Hexanes to 30% EtOAc/70% Hexanes) to afford a brown solid (0.295 g, 84 % yield).

^{1}H NMR (400 MHz, $CDCl_3$): δ (ppm) = 7.28 (s, 2H), 4.84 (s, 2H), 3.44 (s, 2H).

^{13}C NMR (125 MHz, $CDCl_3$): δ (ppm) = 149.08, 139.92, 121.06, 107.63, 84.11, 78.95.

Elemental analysis, Found: C, 67.85; H, 2.93; N, 8.55%. Calc. for $C_{10}H_6ClN$: C, 68.39; H, 3.44; N, 7.98%.

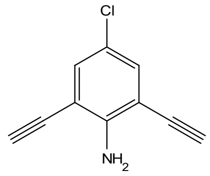

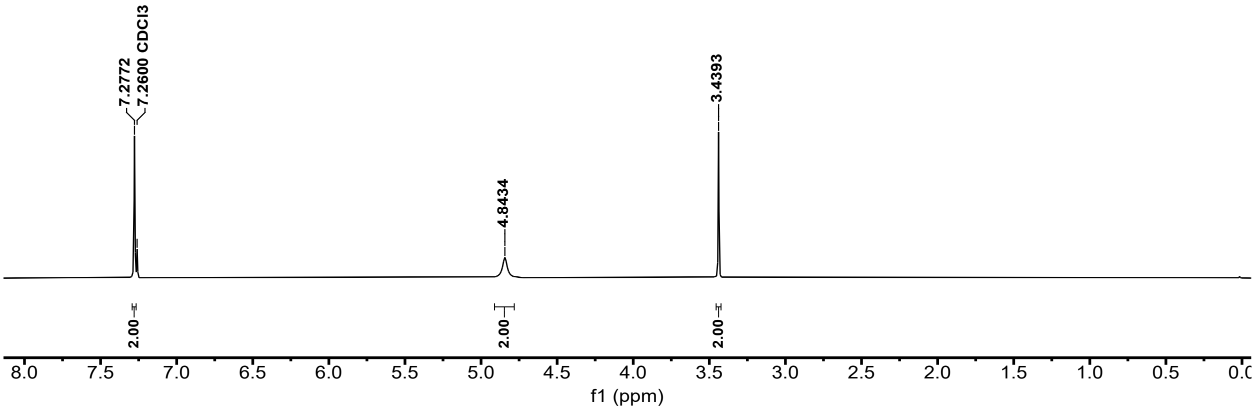

Figure S5. ^{1}H NMR of **2b** (400 MHz, $CDCl_3$).

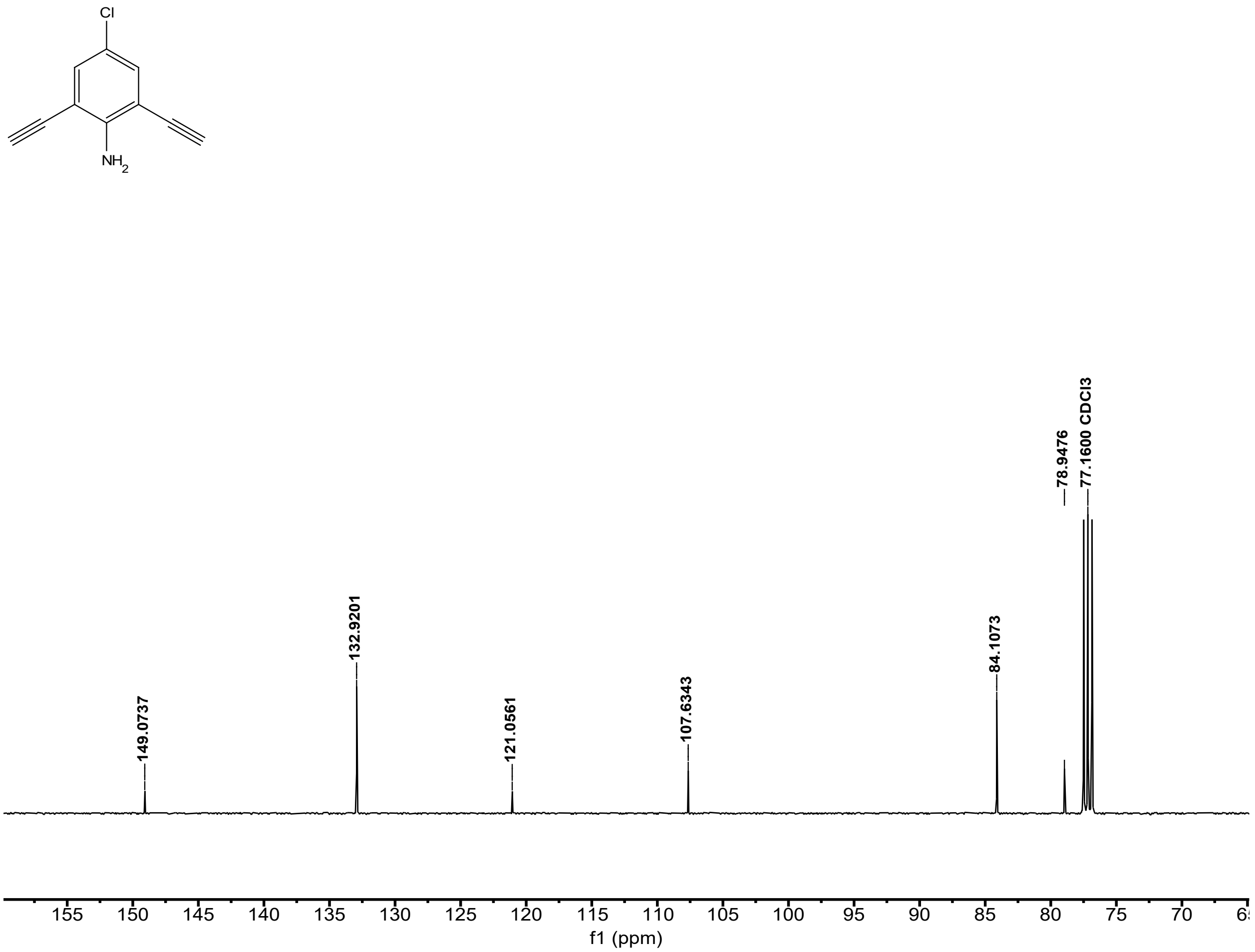

Figure S6. ^{13}C NMR of **2b** (100 MHz, $CDCl_3$).

2,6-bisethynyl-4-fluoroaniline (2c). To an oven dried Schlenk flask was charged with 2,6-dibromo-4-fluoroaniline (**1c**)(0.534 g, 2 mmol), then vacuumed and backfilled with dry N_2 gas (3x). Bis(triphenylphosphine)palladium(II) dichloride (0.07 g, 0.1 mmol) was added, vacuumed and backfilled with dry N_2 (3x). Copper (I) iodide (0.038 g, 0.2 mmol) was added, vacuumed, and backfilled with dry N_2 (3x). The dry reagents were dissolved in 60 mL dry dimethylformamide (DMF). N,N-diisopropylethylamine (1.74 mL, 10 mmol) and TMS-acetylene (0.692 mL, 5 mmol) were added to the DMF solution. The flask was carefully vacuumed and backfilled with dry N_2 (3x). The dark brown solution stirred overnight at 75°C. The reaction mixture was run through a silica plug with a hexane/ethyl acetate solvent mixture (50:50) to remove any excess salts and catalysts. Subsequent removal of DMF, hexanes and ethyl acetate by rotary evaporation left a brown liquid that was used directly in the next step with no purification. The brown liquid was dissolved in 50 mL methanol and 50 mL DCM in a 250 mL round bottom flask. Potassium carbonate (0.69 g, 0.5 mmol) was added to the organic mixture. The reaction stirred vigorously for 4 hours at room temperature and reaction progress was monitored via TLC. Upon completion, water was added to quench the reaction. The crude product was extracted with ethyl acetate, dried over magnesium sulfate and gravity filtered. The organic mixture was reduced under

vacuum and crude product was purified by column chromatography (gradient from 5% EtOAc/95% Hexanes to 30% EtOAc/70% Hexanes) to afford a yellow solid (0.251 g, 79 % yield). Molecule **2c** spectra in accordance with previously reported.[1]

2,6-bisethynylaniline (2d). To an oven dried Schlenk flask was charged with 2,6-dibromoaniline (**1d**) (0.502 g, 2 mmol), then vacuumed and backfilled with dry N_2 gas (3x). Bis(triphenylphosphine)palladium(II) dichloride (0.07 g, 0.1 mmol) was added, vacuumed and backfilled with dry N_2 (3x). Copper (I) iodide (0.038 g, 0.2 mmol) was added, vacuumed, and backfilled with dry N_2 (3x). The dry reagents were dissolved in 60 mL dry dimethylformamide (DMF) at 0°C and stirred for 1 hour. Tetraethylamine (50 mL, excess) and TMS-acetylene (0.692 mL, 5 mmol) were added to the DMF solution. The flask was carefully vacuumed and backfilled with dry N_2 (3x). The dark brown solution stirred overnight at 60°C. The reaction mixture was run through a silica plug with a hexane/ethyl acetate solvent mixture (50:50) to remove any excess salts and catalysts. Subsequent removal of DMF, hexanes and ethyl acetate by rotary evaporation left a brown liquid that was used directly in the next step with no purification. The brown liquid was dissolved in 50 mL methanol and 50 mL DCM in a 250 mL round bottom flask. Potassium carbonate (0.69 g, 0.5 mmol) was added to the organic mixture. The reaction stirred vigorously for 4 hours at room temperature and reaction progress was monitored via TLC. Upon completion, water was added to quench the reaction. The crude product was extracted with ethyl acetate, dried over magnesium sulfate and gravity filtered. The organic mixture was reduced under vacuum and crude product was purified by column chromatography (gradient from 100% Hexanes to 5% EtOAc/95% Hexanes) to afford a yellow solid (0.152 g, 54 % yield). Molecule **2d** spectra in accordance with previously reported.[2]

2,6-bisethynyl-4-methylaniline (2e). To an oven dried Schlenk flask was charged with 2,6-dibromo-4-methylaniline (**1e**) (0.530 g, 2 mmol), then vacuumed and backfilled with dry N_2 gas (3x). Bis(triphenylphosphine)palladium(II) dichloride (0.07 g, 0.1 mmol) was added, vacuumed and backfilled with dry N_2 (3x). Copper (I) iodide (0.038 g, 0.2 mmol) was added, vacuumed, and backfilled with dry N_2 (3x). The dry reagents were dissolved in 60 mL dry dimethylformamide (DMF) at 0°C and stirred for 1 hour. Tetraethylamine (50 mL, excess) and TMS-acetylene (0.692 mL, 5 mmol) were added to the DMF solution. The flask was carefully vacuumed and backfilled with dry N_2 (3x). The dark brown solution stirred overnight at 60°C. The reaction mixture was run through a silica plug with a hexane/ethyl acetate solvent mixture (50:50) to remove any excess salts and catalysts. Subsequent removal of DMF, hexanes and ethyl acetate by rotary evaporation left a brown liquid that was used directly in the next step with no purification. The brown liquid was dissolved in 50 mL methanol and 50 mL DCM in a 250 mL round bottom flask. Potassium carbonate (0.69 g, 0.5 mmol) was added to the organic mixture. The reaction stirred vigorously for 4 hours at room temperature and reaction progress was monitored via TLC. Upon completion, water was added to quench the reaction. The crude product was extracted with ethyl acetate, dried over magnesium sulfate and gravity filtered. The organic mixture was reduced under vacuum and crude product was purified by column chromatography (gradient from 100% Hexanes to 3% EtOAc/97% Hexanes) to afford a brown solid (0.139 g, 45 % yield). Molecule **2e** spectra in accordance with previously reported.[3]

3,5-bisethynyl-trifluoromethylbenzene (2f). To an oven dried Schlenk flask was charged with Bis(triphenylphosphine)palladium(II) dichloride (0.231 g, 0.33 mmol) and then vacuumed and backfilled with dry N_2 (3x). Copper (I) iodide (0.125 g, 0.66 mmol) was added, vacuumed, and backfilled with dry N_2 (3x). The dry reagents were dissolved in 150 mL dry dimethylformamide (DMF). 3,5-dibromo-trifluoromethylbenzne (**1f**) (1.0 mL, 6.6 mmol), N,N-diisopropylethylamine (5.66 mL, 33 mmol) and TMS-acetylene (2.28 mL, 16.5 mmol) were added to the DMF solution. The flask was carefully vacuumed and backfilled with dry N_2 (3x). The dark brown solution stirred overnight at 85°C. The reaction mixture was run through a silica plug with hexane to remove any excess salts and catalysts. Subsequent removal of DMF and hexane by rotary evaporation left a brown liquid that was used directly in the next step with no purification. The brown liquid was dissolved in 50 mL methanol and 50 mL DCM in a 250 mL round bottom flask. Potassium carbonate (2.28 g, 16.5 mmol) was added to the organic mixture. The reaction stirred vigorously for one hour at room temperature and reaction progress was monitored via TLC. Upon completion, water was added to quench the reaction. The crude product was extracted with ethyl acetate, dried over magnesium sulfate and gravity filtered. The organic mixture was reduced under vacuum and crude product was purified by column chromatography (gradient from 5% EtOAc/95% Hexanes to 30% EtOAc/70% Hexanes) to afford a light orange oil (1.232 g, 96 % yield). Molecule 4 spectra in accordance with previously reported.[4]

2,6-bis(2-ethynyl-4-(trifluoromethyl)bromophenyl)-4-(trifluoromethyl)aniline (G3HB). To an oven dried Schlenk flask was charged with 2,6-bisethynyl-4-(trifluoromethyl)aniline **(2a)** (0.211g 1mmol), then vacuumed and backfilled with dry N_2 gas (3x). Bis(triphenylphosphine)palladium (II) dichloride (0.035 g, 0. 05 mmol) was added, vacuumed and backfilled with dry N_2 gas (3x). Copper (I) iodide (0.019 g, 0.1 mmol) was added, vacuumed and backfilled with dry N_2 (3x). The dry reagents were dissolved in 60 mL dry DMF. 3-iodo-trifluoromethylbenzene (0.36 mL, 2.5 mmol) and N,N-diisopropylethylamine (0.86 mL, 5 mmol) were added to the DMF solution. The flask was carefully vacuumed and backfilled with dry N_2 (3x). The dark brown solution stirred overnight at room temperature. The reaction mixture was extracted with ethyl acetate then washed with water to remove any excess salts and catalysts, then was dried over magnesium sulfate and gravity filtered. Subsequent removal of DMF, hexanes and ethyl acetate by rotary evaporation left and brown solid that was purified by column chromatography (gradient from 5% DCM/95% Hexanes to 10% DCM/95% Hexanes) to afford **G3HB** (0.395 g, 60%) as a dull yellow solid.

^{1}H NMR (400 MHz, $CDCl_3$): δ (ppm) = 7.81 (s, 2H), 7.72 (d, J = 7.8 Hz, 2H), 7.64 (d, J = 6.1 Hz, 2H), 7.63 (s, 1H), 7.52 (t, J = 7.7 Hz, 2H), 5.22 (s, 2H).

^{13}C NMR (100 MHz, $CDCl_3$): δ (ppm) = 151.13, 134.78, 131.40 (q, J = 35.8 Hz), 130.03 (q, J = 4.6 Hz), 129.29, 128.51 (q, J = 3.6 Hz), 125.58 (q, J = 4.0 Hz), 123.96 (q, J = 268.9 Hz), 123.76 (q, J = 271.1 Hz), 123.50, 120.10 (q, J = 33.2 Hz), 107.01, 95.65, 85.53.

^{19}F NMR (376 MHz, $CDCl_3$): δ (ppm) = –60.19, -61.37.

HRMS (ESI neg) m/z for $C_{25}H_{11}F_9N^-$ $[M-H^+]^-$: calculated: 496.0753; found: 496.0758.

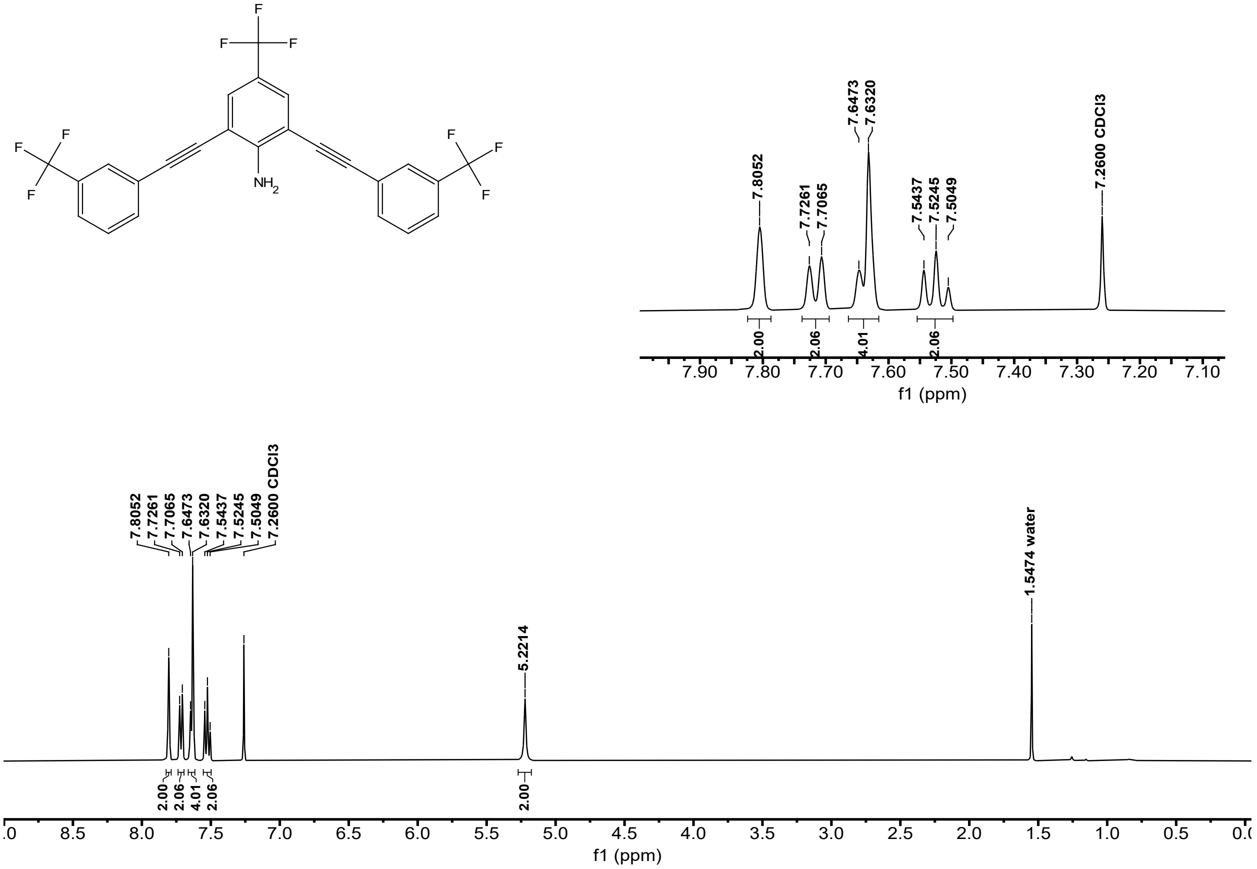

Figure S7. ^{1}H NMR of **G3HB** (400 MHz, $CDCl_3$).

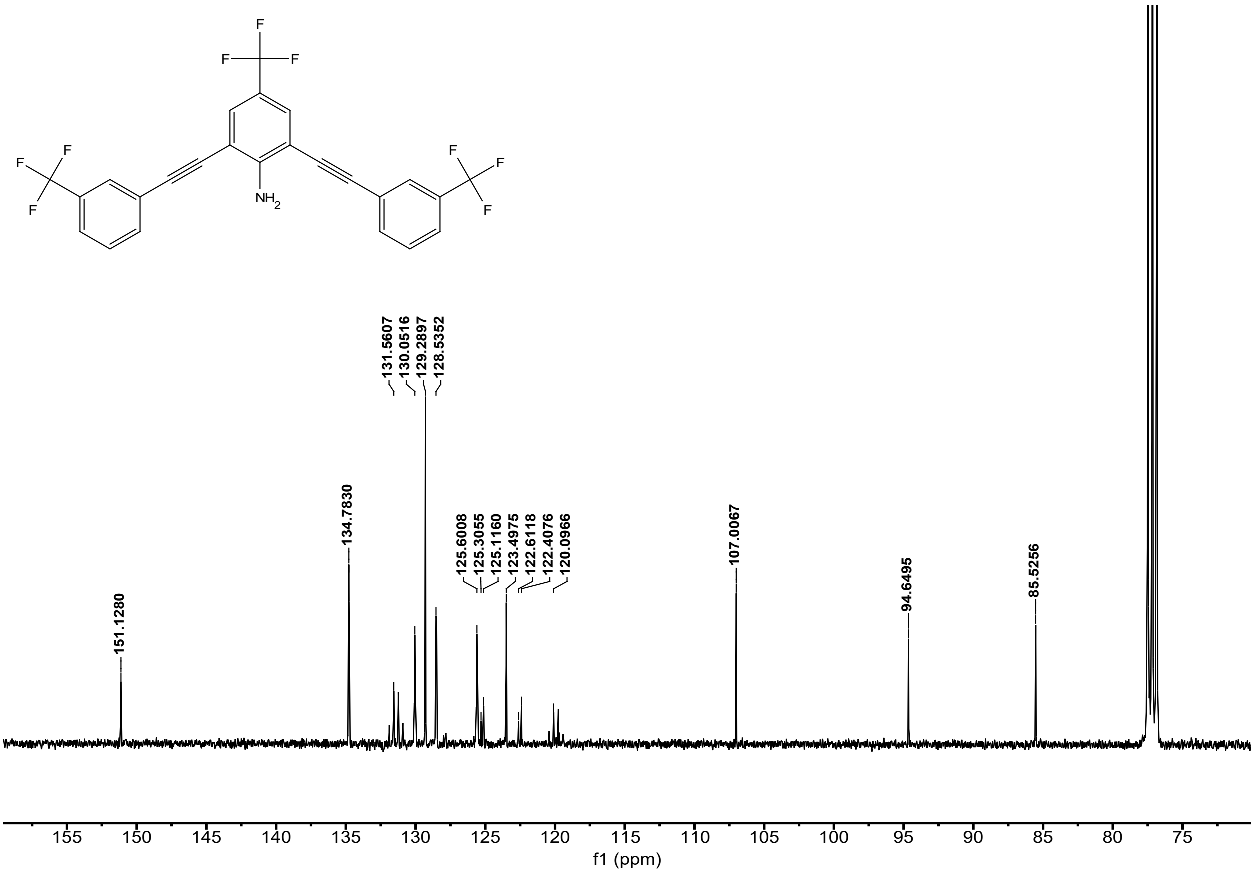

Figure S8. ^{13}C NMR of **G3HB** (100 MHz, $CDCl_3$).

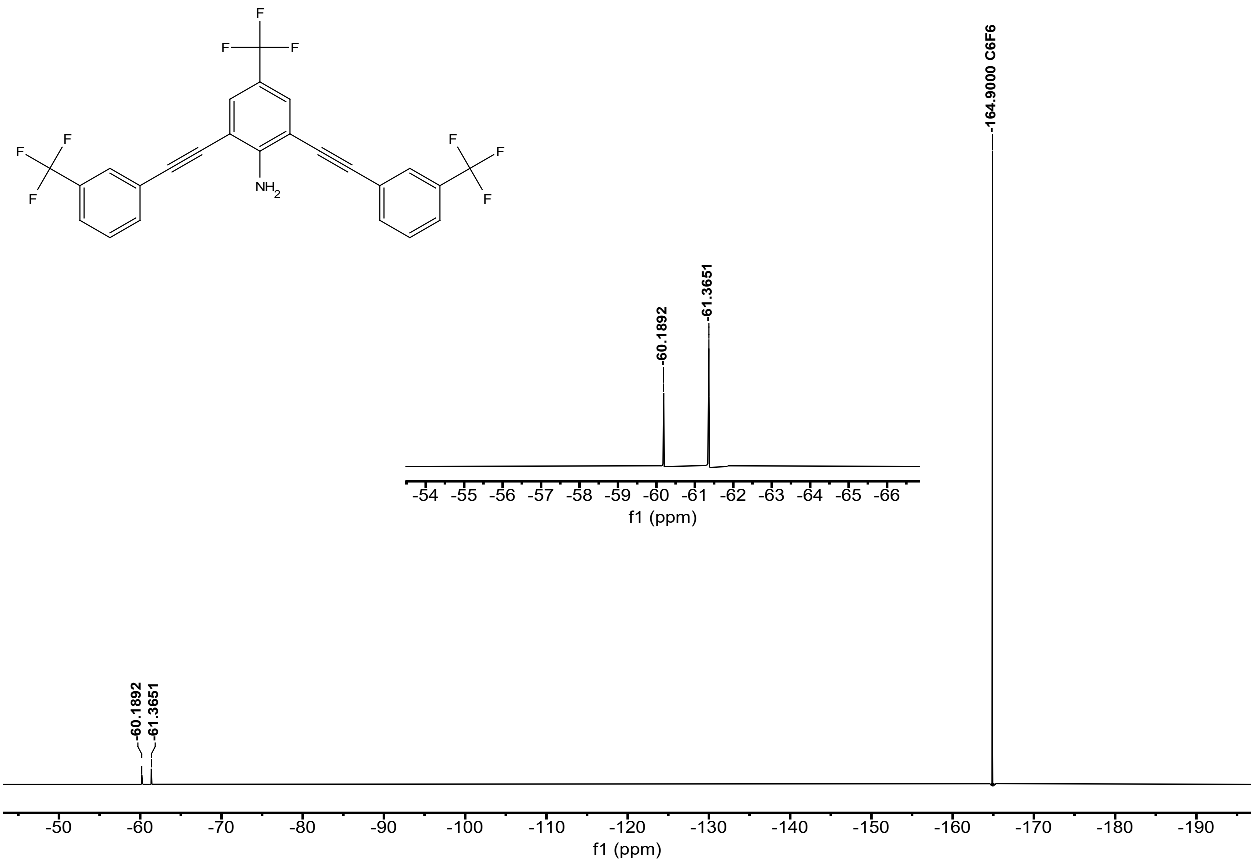

Figure S9. ^{19}F NMR of **G3HB** (376 MHz, $CDCl_3$). Hexafluorobenzene (C_6F_6) internal reference.

2,6-bis(2-ethynyl-4-(trifluoromethyl)bromophenyl)-4-(trifluoromethyl)aniline (G3XBBr). To an oven dried Schlenk flask was charged with 2,6-bisethynyl-4-(trifluoromethyl)aniline **(2a)** (0.211g 1mmol), then vacuumed and backfilled with dry N_2 gas (3x). Bis(triphenylphosphine)palladium (II) dichloride (0.034 g, 0. 05 mmol) was added, vacuumed and backfilled with dry N_2 gas (3x). Copper (I) iodide (0.019 g, 0.1 mmol) was added, vacuumed and backfilled with dry N_2 (3x). The dry reagents were dissolved in 60 mL dry DMF. 1-Bromo-2-iodo-4-(trifluoromethyl)benzene (0.4 mL, 2.5 mmol) and N,N-diisopropylethylamine (0.86 mL, 5 mmol) were added to the DMF solution. The flask was carefully vacuumed and backfilled with dry N_2 (3x). The dark brown solution stirred overnight at room temperature. The reaction mixture was extracted with ethyl acetate then washed with water to remove any excess salts and catalysts, then was dried over magnesium sulfate and gravity filtered. Subsequent removal of DMF, hexanes and ethyl acetate by rotary evaporation left and brown solid that was purified by column chromatography (gradient from 100% Hexanes to 5% EtOAc/95% Hexanes) to afford **G3XBBr** (0.492 g, 75%) as a dull yellow solid.

^{1}H NMR (400 MHz, $CDCl_3$): δ (ppm) = 7.84 (s, 2H), 7.79 (d, J = 7.8 Hz, 2H), 7.67 (s, 2H), 7.47 (d, J = 6.1 Hz, 2H), 5.65 (s, 2H).

^{13}C NMR (100 MHz, CDCl$_3$): δ (ppm) = 152.09, 133.24, 130.57 (q, J = 33.5 Hz), 130.36 (q, J = 3.7 Hz), 129.99 (q, J = 3.7 Hz), 128.98, 126.36 (q, J = 3.7 Hz), 125.82, 123.89 (q, J = 265.4 Hz), 123.47 (q, J = 269.7 Hz), 119.89 (q, J = 34.1 Hz), 106.46, 93.57, 90.49.

^{19}F NMR (376 MHz, CDCl$_3$): δ (ppm) = –60.14, -61.33.

HRMS (ESI neg) m/z for $C_{25}H_9F_9Br_2N^-$ [M-H$^+$]$^-$: calculated: 653.8943; found: 653.8943.

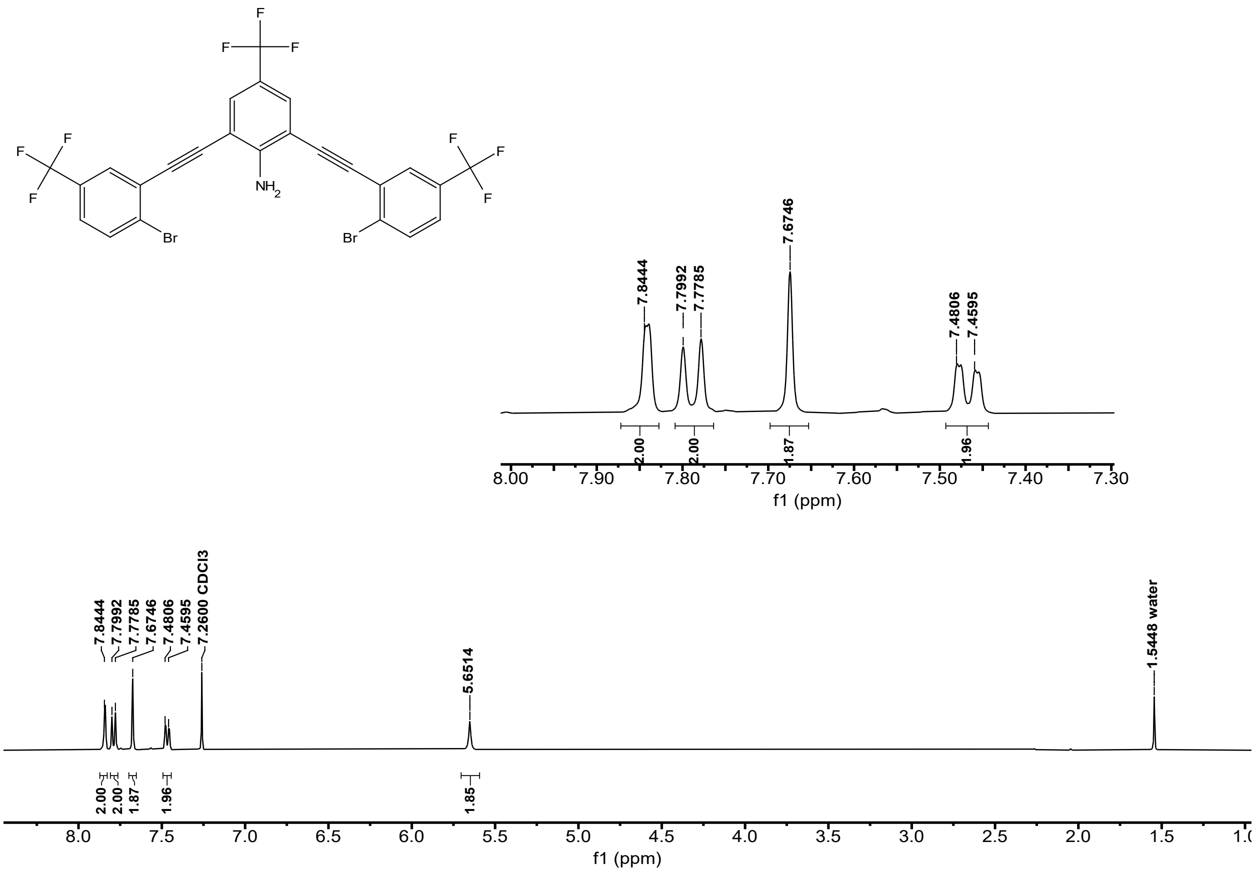

Figure S10. ^{1}H NMR of **G3XBBr** (400 MHz, CDCl$_3$).

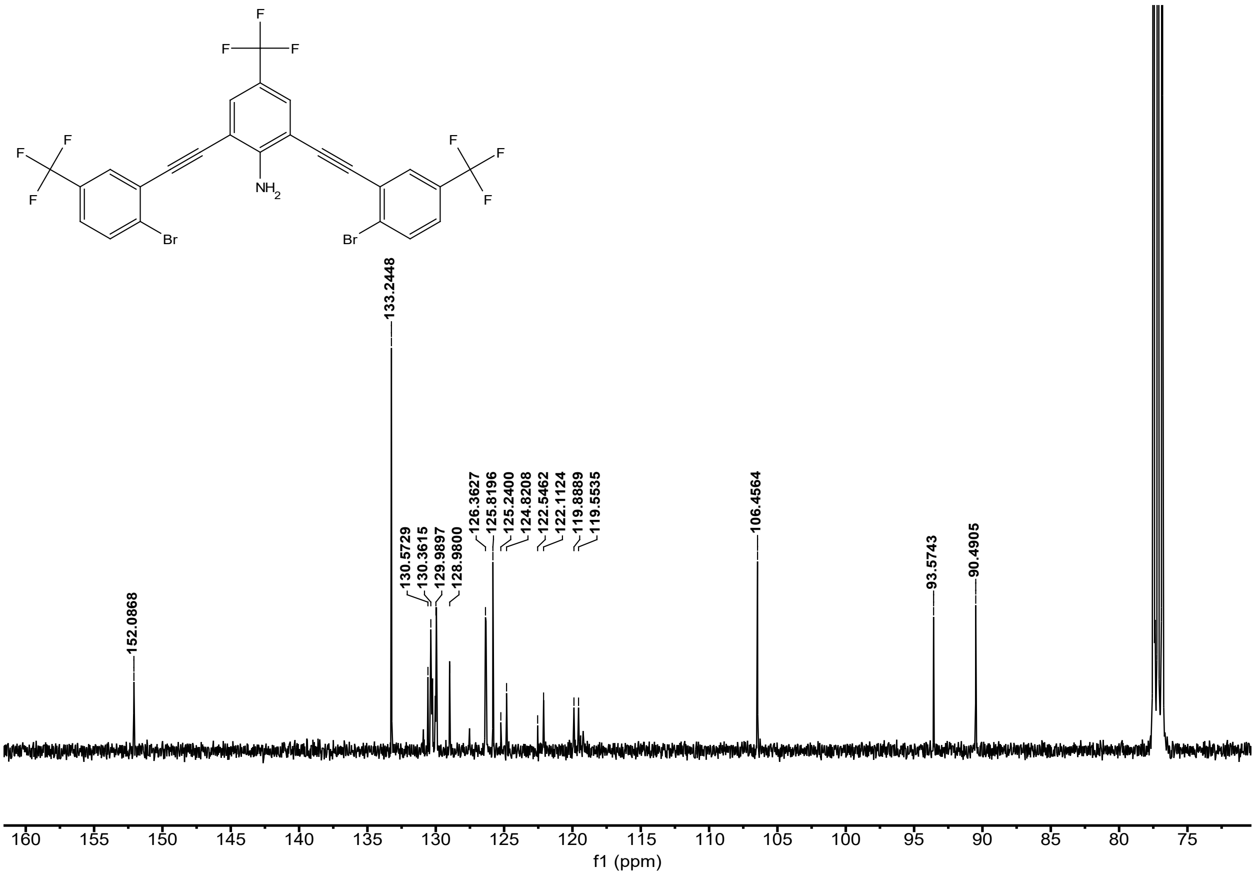

Figure S11. ^{13}C NMR of **G3XBBr** (100 MHz, $CDCl_3$).

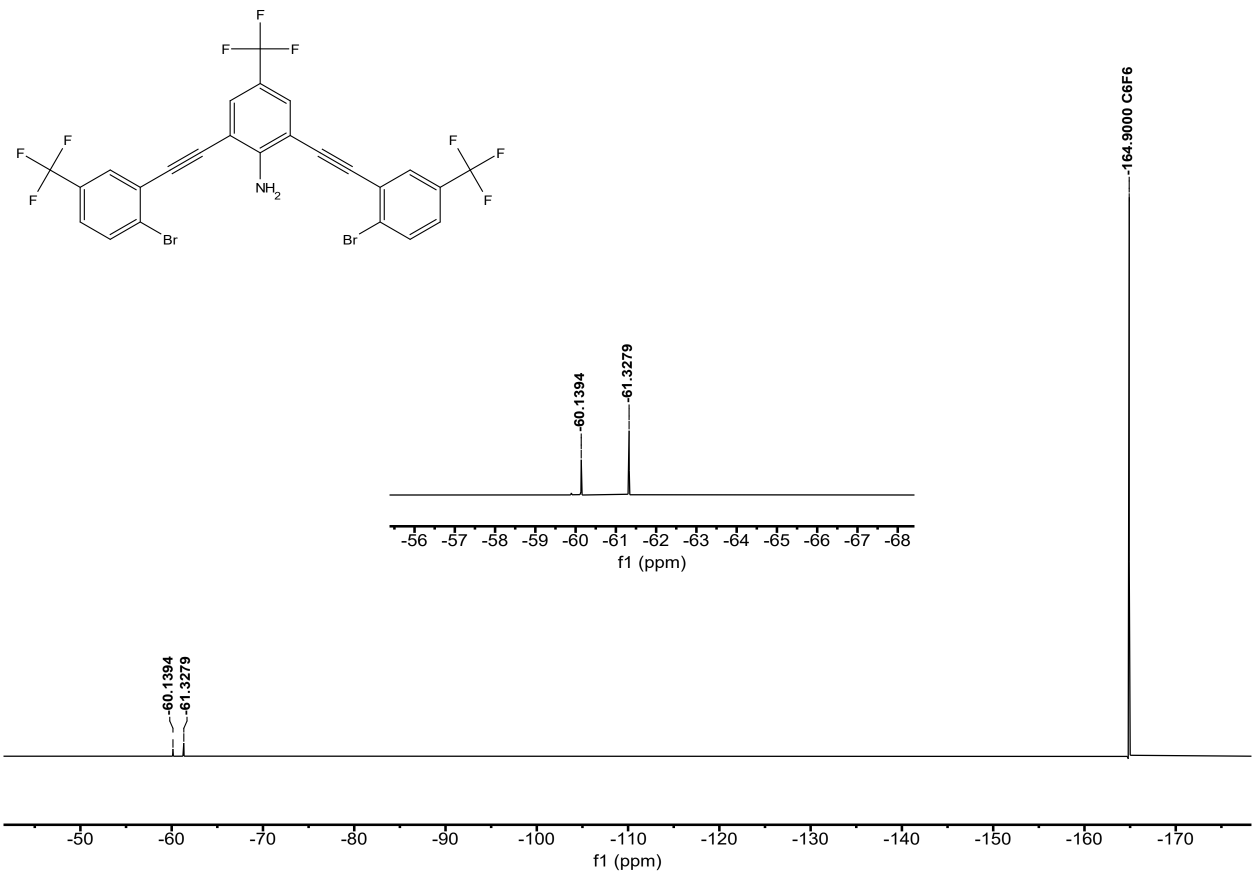

Figure S12. ^{19}F NMR of **G3XBBr** (376 MHz, $CDCl_3$). Hexafluorobenzene (C_6F_6) internal reference.

2,6-bis(2-ethynyl-4-(trifluoromethyl)iodophenyl)-4-(trifluoromethyl)aniline (G3XB). G3XBBr (0.200 g, 0.305 mmol), copper iodide (0.006 g, 0.030 mmol), sodium iodide (0.183 g, 1.22 mmol) were added to a 10-20 mL microwave reaction vial containing a stir bar and dissolved in 13 mL 1,4-dioxane. To the vibrant yellow reaction mixture, trans-N,N'-dimethylcyclohexane-1,2-diamine (0.1 mL) was added. The microwave vial was sealed and placed in a Biotage Initiator+ microwave reactor for 12.5 hours at 150 ˚C. After cooling, the reaction was run through pipet silica plug with EtOAc to remove catalysts and salts. The EtOAc crude was then ran through GCMS in order to obtain % conversion of bromines to iodines. If the conversion from bromine to iodine was 99% or greater, then the reaction was concentrated. The beige/yellow product (0.13 g, 58%) was obtained after purified via high performance liquid chromatography (reverse phase column gradient from 80% water/20% acetonitrile to 100% acetonitrile).

^{1}H NMR (400 MHz, $CDCl_3$): δ (ppm) = 8.04 (d, J = 8.2 Hz, 2H), 7.81 (s, 2H), 7.69 (s, 2H), 7.29 (d, J = 8.4 Hz, 2H), 5.73 (s, 2H).

^{13}C NMR (100 MHz, $CDCl_3$): δ (ppm) = 152.16, 145.84, 139.54, 131.05 (q, J = 33.5 Hz), 130.36 (q, J = 3.6 Hz), 129.23 (q, J = 4.0 Hz), 126.15 (q, J = 4.0 Hz), 123.91 (q, J = 269.4 Hz), 123.60 (q, J = 270.5 Hz), 119.63 (q, J = 33.2 Hz), 106.50, 104.78, 96.85, 89.67.

^{19}F NMR (376 MHz, CDCl$_3$): δ (ppm) = –60.15, -61.56.

HRMS (ESI neg) m/z for $C_{25}H_9F_9I_2N^-$ [M-H$^+$]$^-$: calculated: 747.8686; found: 747.8689.

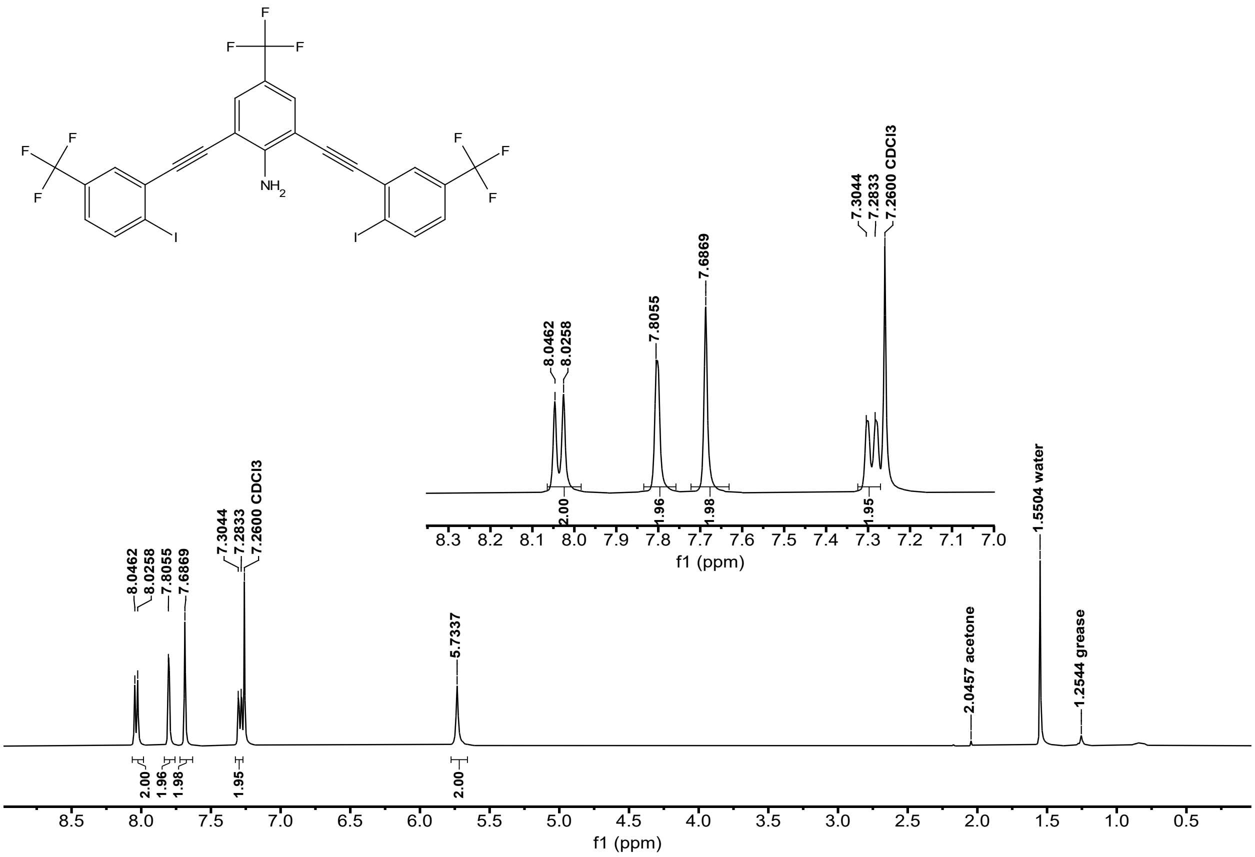

Figure S13. ^{1}H NMR of **G3XB** (400 MHz, CDCl$_3$).

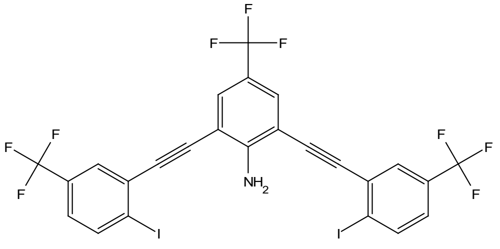

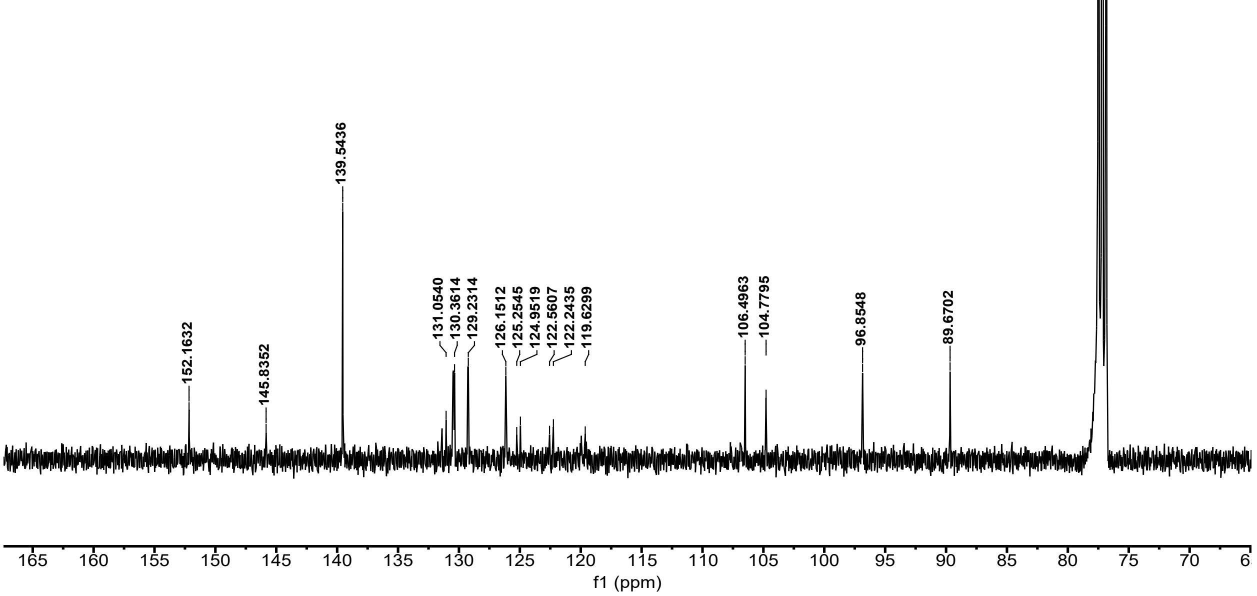

Figure S14. ^{13}C NMR of **G3XB** (100 MHz, $CDCl_3$).

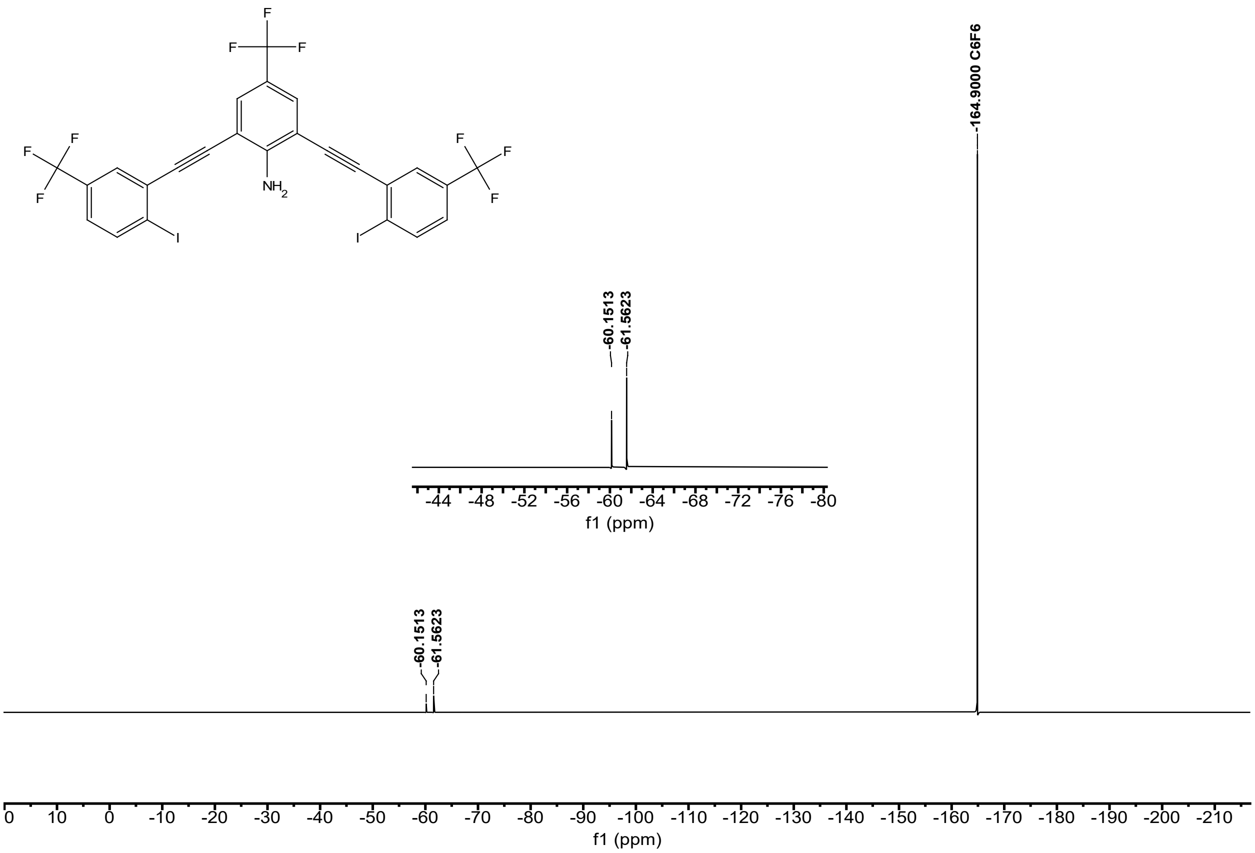

Figure S15. ^{19}F NMR of **G3XB** (376 MHz, $CDCl_3$). Hexafluorobenzene (C_6F_6) internal reference.

2,6-bis(2-ethynyl-4-(trifluoromethyl)bromophenyl)-4-(trifluoromethyl)aniline (2F-G3XBBr). To an oven dried Schlenk flask was charged with 2,6-bisethynyl-4-(trifluoromethyl)aniline **(2a)** (0.211g 1mmol), then vacuumed and backfilled with dry N_2 gas (3x). Bis(triphenylphosphine)palladium (II) dichloride (0.035 g, 0. 05 mmol) was added, vacuumed and backfilled with dry N_2 gas (3x). Copper (I) iodide (0.019 g, 0.1 mmol) was added, vacuumed and backfilled with dry N_2 (3x). The dry reagents were dissolved in 60 mL dry DMF. 1-Bromo-2-iodo-4-fluorobenzene (0.33 mL, 2.5 mmol) and N,N-diisopropylethylamine (0.86 mL, 5 mmol) were added to the DMF solution. The flask was carefully vacuumed and backfilled with dry N_2 (3x). The dark brown solution stirred overnight at room temperature. The reaction mixture was extracted with ethyl acetate then washed with water to remove any excess salts and catalysts, then was dried over magnesium sulfate and gravity filtered. Subsequent removal of DMF, hexanes and ethyl acetate by rotary evaporation left and brown solid that was purified by column chromatography (gradient from 100% Hexanes to 3% EtOAc/97% Hexanes) to afford **2F-G3XBBr** (0.480 g, 87%) as a dull yellow solid.

^1H NMR (400 MHz, $CDCl_3$): δ (ppm) = 7.82 (dd, J = 8.8 Hz, 2H), 7.65 (s, 2H), 7.30 (dd, J = 8.9 Hz, 2H), 6.87-7.82 (dt, J = 8.1 Hz, 2H), 5.76 (s, 2H).

^{13}C NMR (100 MHz, CDCl$_3$): δ (ppm) = 162.64 (d, J = 247.5 Hz), 152.19, 140.18 (d, J = 8.4 Hz), 130.96 (d, J = 9.5 Hz), 130.26 (q, J = 3.6 Hz), 123.96 (q, J = 269.4 Hz), 119.80 (d, J = 23.7 Hz), 119.73 (q, J = 33.5 Hz), 117.99 (d, J = 21.9 Hz), 106.53, 96.99, 98.93, 89.15.

^{19}F NMR (376 MHz, CDCl$_3$): δ (ppm) = –60.08, -111.79.

HRMS (ESI neg) m/z for $C_{23}H_9F_5Br_2N^-$ [M-H$^+$]$^-$: calculated: 553.9007; found: 553.9007.

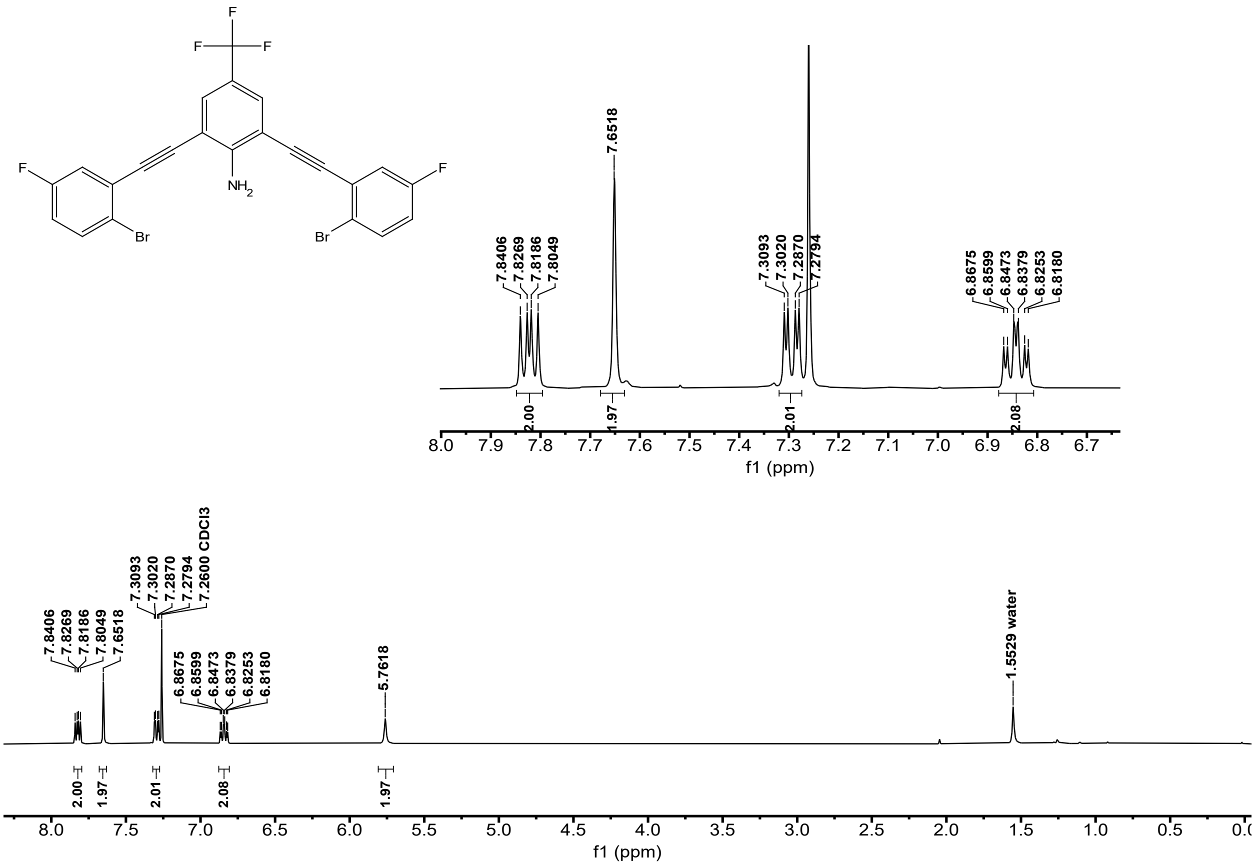

Figure S16. ^{1}H NMR of **2F-G3XBBr** (400 MHz, CDCl$_3$).

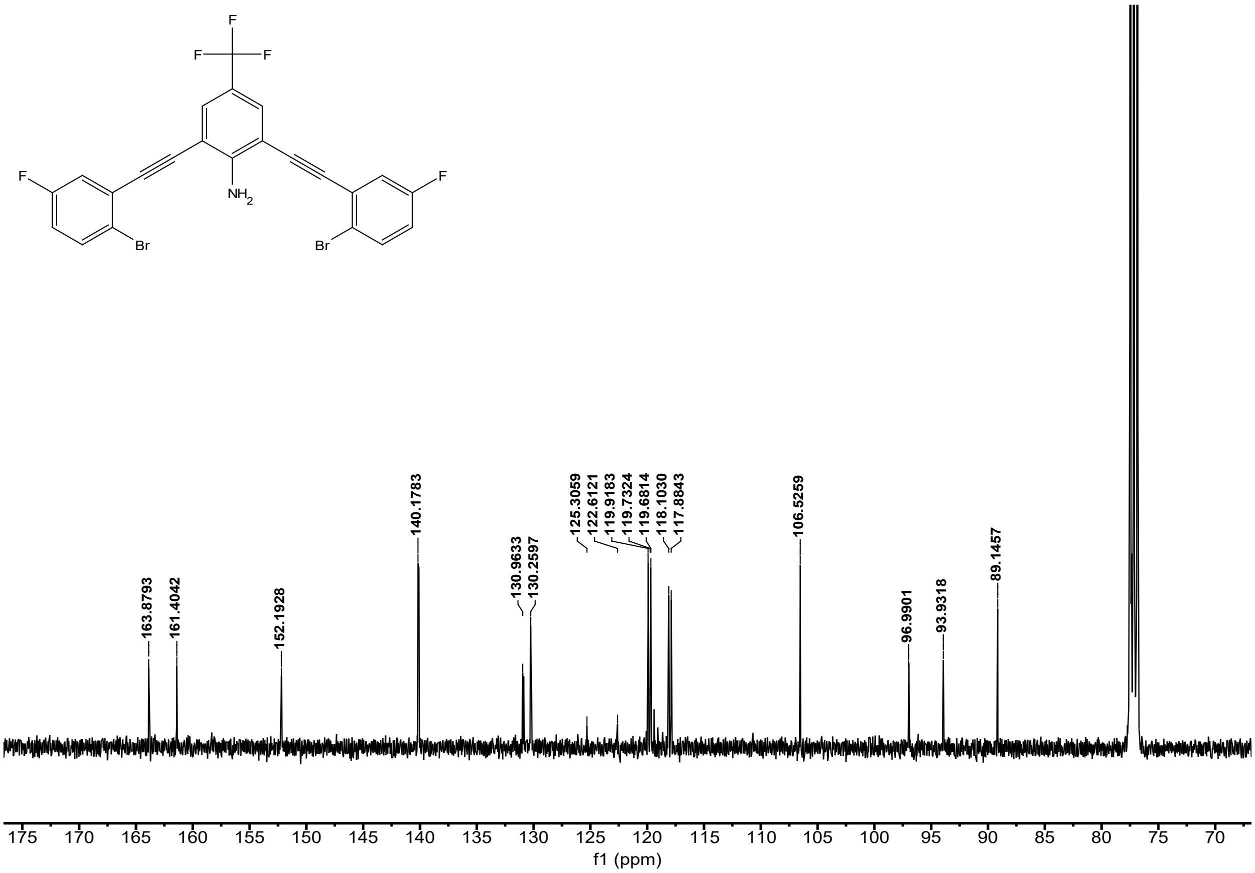

Figure S17. ^{13}C NMR of **2F-G3XBBr** (100 MHz, $CDCl_3$).

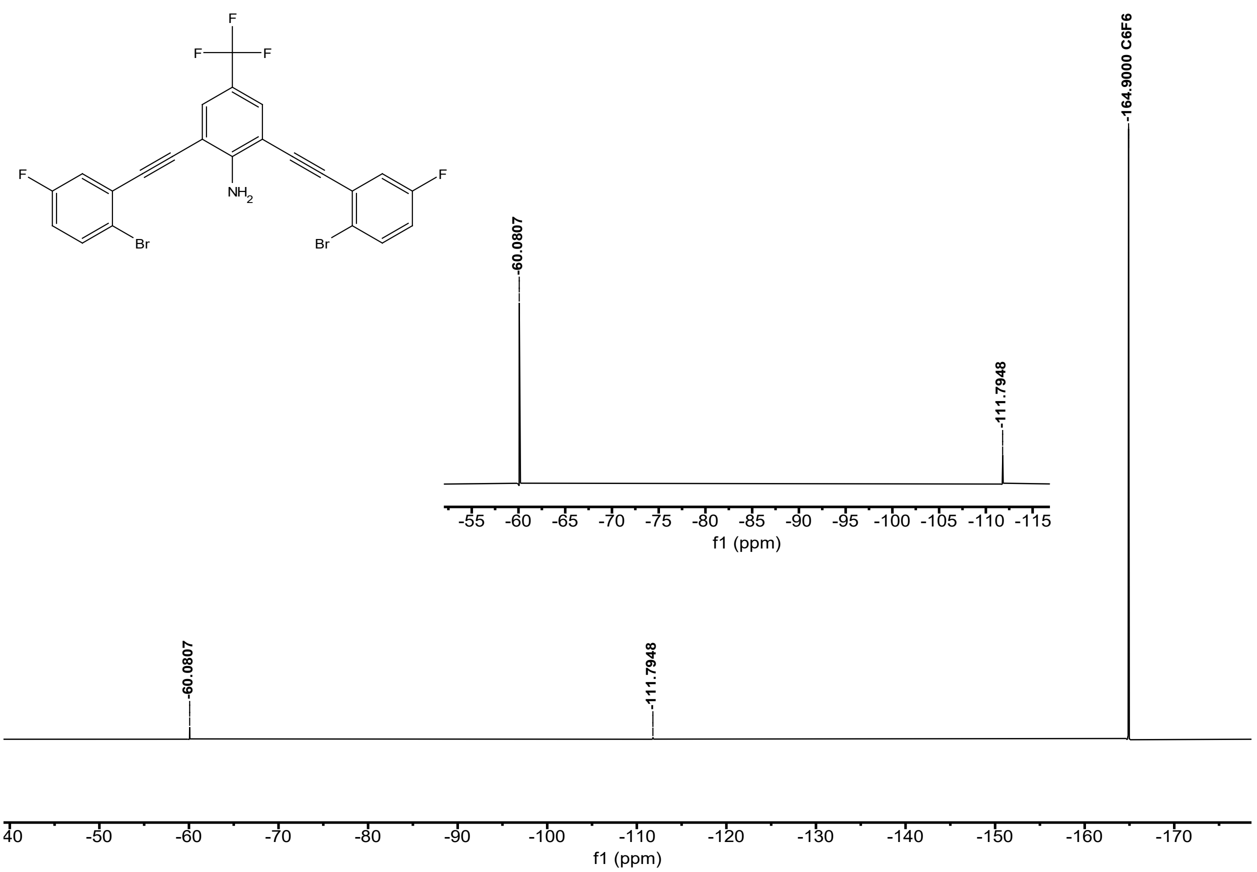

Figure S18. ^{19}F NMR of **2F-G3XBBr** (376 MHz, $CDCl_3$). Hexafluorobenzene (C_6F_6) internal reference.

2,6-bis(2-ethynyl-4-fluoro-iodophenyl)-4-(trifluoromethyl)aniline (2F-G3XB). 2F-G3XBBr (0.200 g, 0.360 mmol), copper iodide (0.006 g, 0.036 mmol), sodium iodide (0.216 g, 1.44 mmol) were added to a 10-20 mL microwave reaction vial containing a stir bar and dissolved in 13 mL 1,4-dioxane. To the vibrant yellow reaction mixture, trans-N,N'-dimethylcyclohexane-1,2-diamine (0.1 mL) was added. The microwave vial was sealed and placed in a Biotage Initiator+ microwave reactor for 16 hours at 150 ˚C. After cooling, the reaction was run through pipet silica plug with EtOAc to remove catalysts and salts. The EtOAc crude was then ran through GCMS in order to obtain % conversion of bromines to iodines. If the conversion from bromine to iodine was 99% or greater, then the reaction was concentrated. The beige/yellow product (0.187 g, 80%) was obtained after purified via high performance liquid chromatography (reverse phase column gradient from 80% water/20% acetonitrile to 100% acetonitrile).

^{1}H NMR (400 MHz, $CDCl_3$): δ (ppm) = 7.82 (dd, J = 5.5 Hz, 2H), 7.65 (s, 2H), 7.30 (dd, J = 6.0 Hz, 2H), 6.87-6.82 (dt, J = 8.0 Hz, 2H), 5.76 (s, 2H).

^{13}C NMR (100 MHz, $CDCl_3$): δ (ppm) = 162.63 (d, J = 247.5 Hz), 152.18, 140.17 (d, J = 8.4 Hz), 130.95 (d, J = 9.1 Hz), 130.21 (q, J = 3.6 Hz), 123.95 (q, J = 269.4 Hz), 119.80 (d, J = 23.7 Hz) 119.72 (q, J = 33.5 Hz), 117.98 (d, J = 21.9 Hz), 106.52, 96.95, 93.92, 89.14.

^{19}F NMR (376 MHz, CDCl$_3$): δ (ppm) = –60.08, -111.80.

HRMS (ESI neg) m/z for $C_{23}H_9F_5I_2N^-$ [M-H$^+$]$^-$: calculated: 647.8750; found: 647.8750.

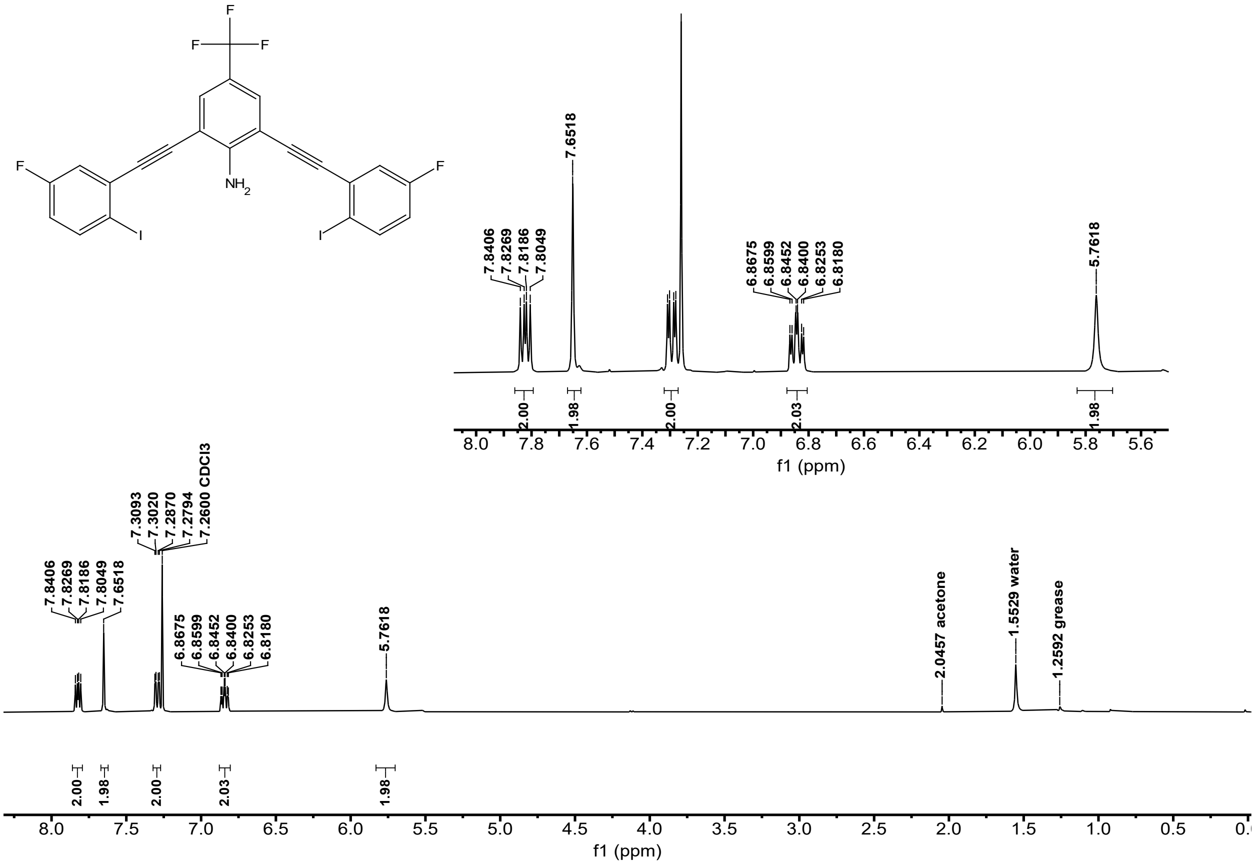

Figure S19. ^{1}H NMR of **2F-G3XB** (400 MHz, CDCl$_3$).

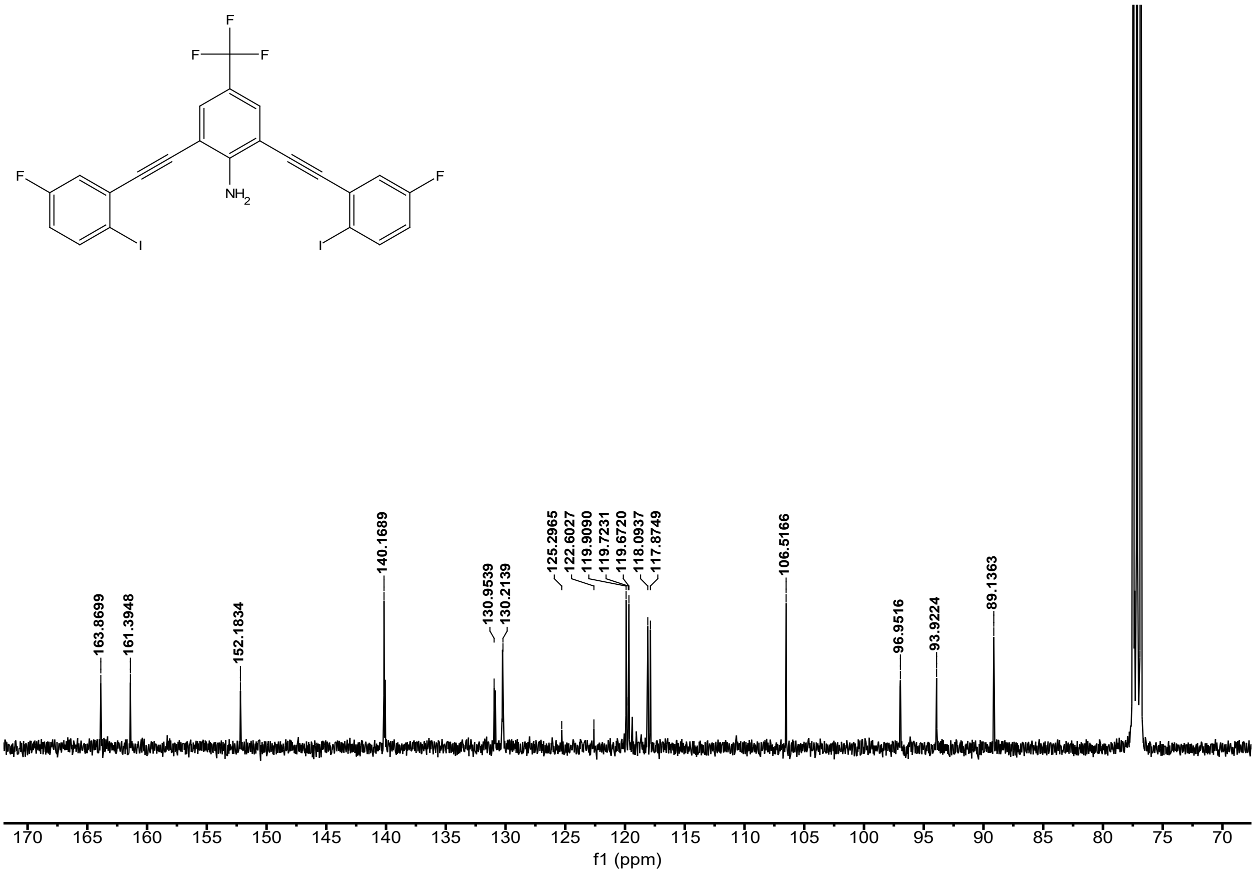

Figure S20. ^{13}C NMR of **2F-G3XB** (100 MHz, $CDCl_3$).

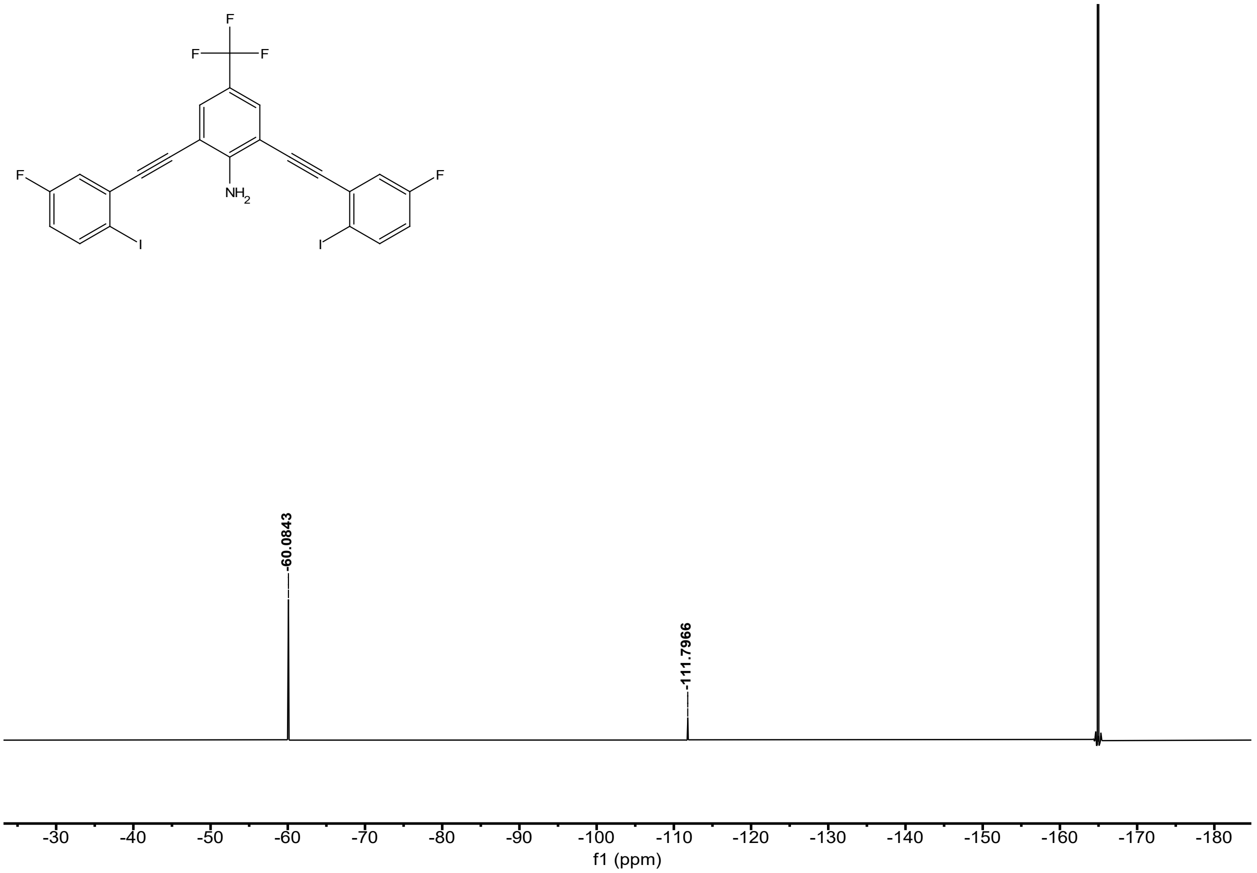

Figure S21. ^{19}F NMR of **2F-G3XB** (376 MHz, $CDCl_3$). Hexafluorobenzene (C_6F_6) internal reference.

2,6-bis(2-ethynyl-bromophenyl)-4-(trifluoromethyl)aniline (2H-G3XBBr). To an oven dried Schlenk flask was charged with 2,6-bisethynyl-4-(trifluoromethyl)aniline **(2a)** (0.375g 1.78mmol), then vacuumed and backfilled with dry N_2 gas (3x). Bis(triphenylphosphine)palladium (II) dichloride (0.063 g, 0. 089 mmol) was added, vacuumed and backfilled with dry N_2 gas (3x). Copper (I) iodide (0.034 g, 0.178 mmol) was added, vacuumed and backfilled with dry N_2 (3x). The dry reagents were dissolved in 100 mL dry DMF. 1-Bromo-2-iodo-benzene (0.57 mL, 4.45 mmol) and N,N-diisopropylethylamine (1.53 mL, 8.9 mmol) were added to the DMF solution. The flask was carefully vacuumed and backfilled with dry N_2 (3x). The dark brown solution stirred overnight at room temperature. The reaction mixture was extracted with ethyl acetate then washed with water to remove any excess salts and catalysts, then was dried over magnesium sulfate and gravity filtered. Subsequent removal of DMF, hexanes and ethyl acetate by rotary evaporation left and brown solid that was purified by column chromatography (gradient from 100% Hexanes to 5% EtOAc/95% Hexanes) to afford **2H-G3XBBr** (0.647 g, 70%) as a yellow solid.

^1H NMR (400 MHz, $CDCl_3$): δ (ppm) = 7.65 (d, J = 8.0 Hz, 2H), 7.63 (s, 2H), 7.59 (d, J = 7.7 Hz, 2H), 7.35 (t, J = 7.6 Hz, 2H), 7.23 (t, J = 7.6 Hz, 2H), 5.70 (s, 2H).

^{13}C NMR (100 MHz, $CDCl_3$): δ (ppm) = 152.02, 133.29, 132.57, 129.99, 129.61 (q, J = 3.7 Hz), 127.49, 125.33, 124.91, 124.11 (q, J = 269.4 Hz), 119.47 (q, J = 33.4 Hz), 106.91, 94.74, 89.09.

^{19}F NMR (376 MHz, CDCl$_3$): δ (ppm) = –59.99.

HRMS (ESI neg) m/z for $C_{23}H_{11}F_3Br_2N^-$ [M-H$^+$]$^-$: calculated: 517.9195; found: 517.9196.

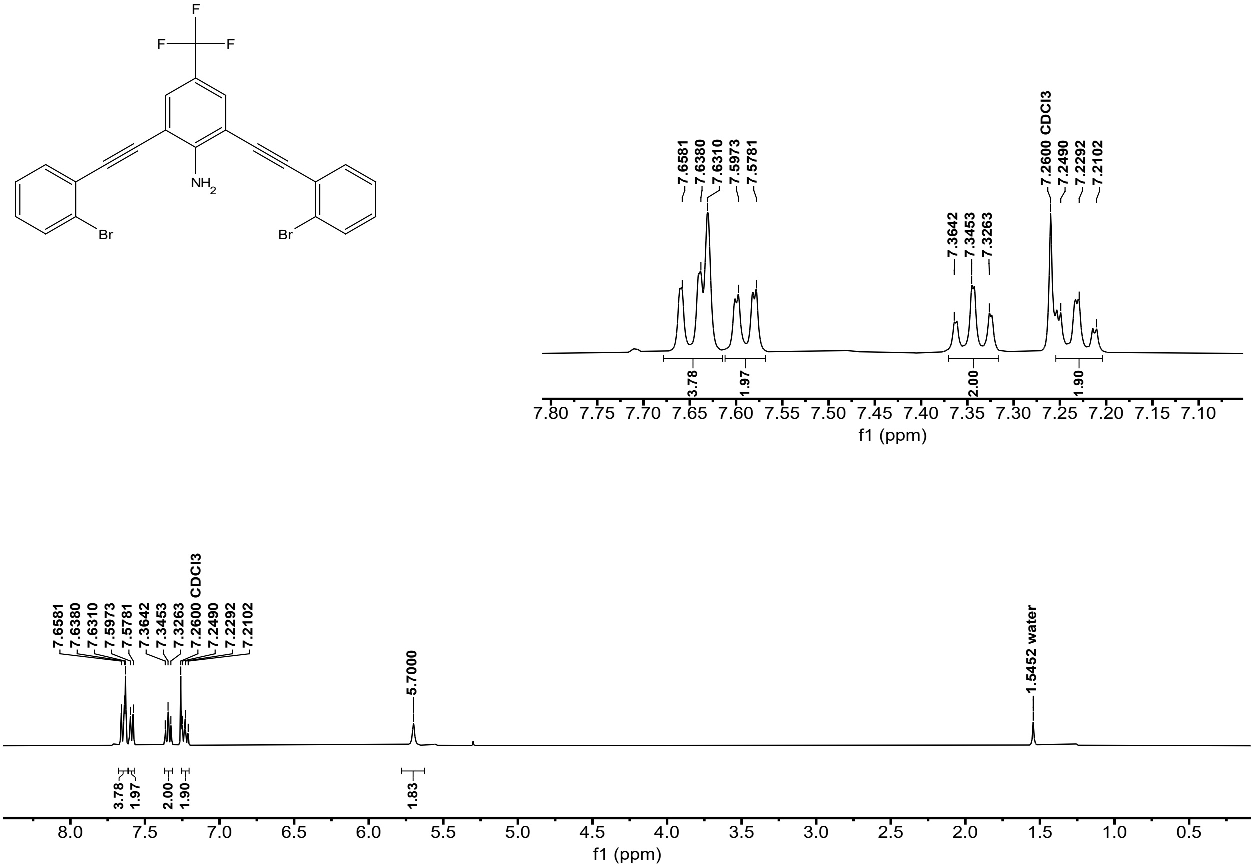

Figure S52. ^{1}H NMR of **2H-G3XBBr** (400 MHz, CDCl$_3$).

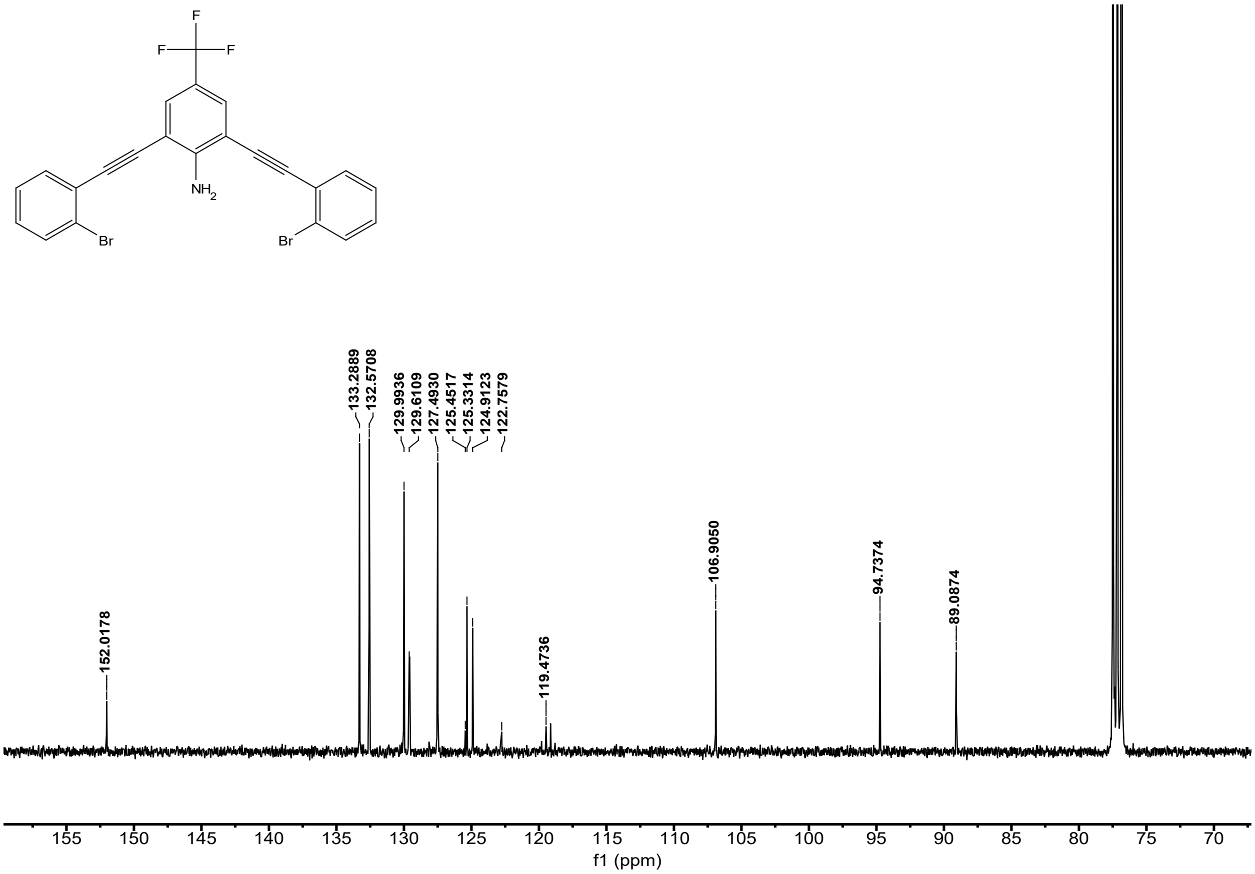

Figure S26. ^{13}C NMR of **2H-G3XBBr** (100 MHz, $CDCl_3$).

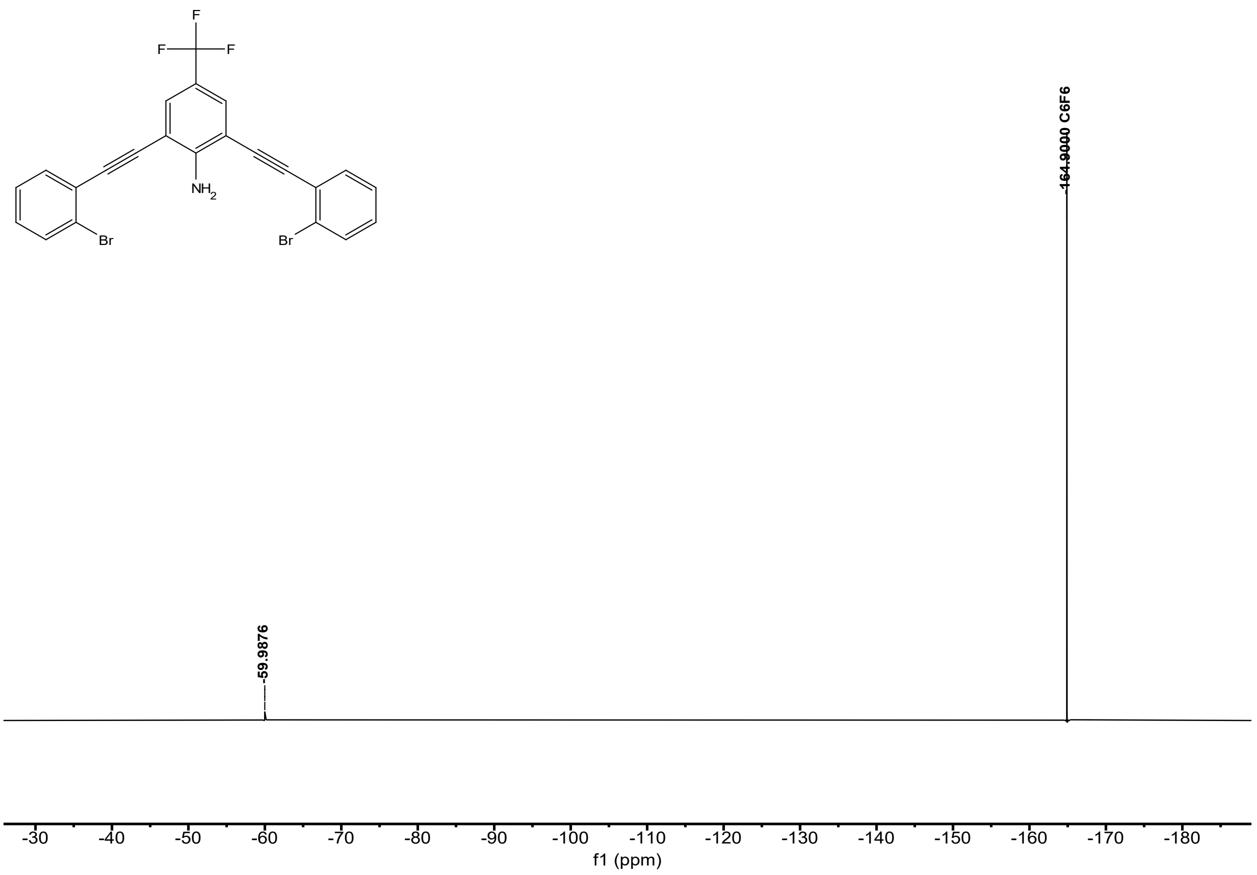

Figure S27. ^{19}F NMR of **2H-G3XBBr** (376 MHz, $CDCl_3$). Hexafluorobenzene (C_6F_6) internal reference.

2,6-bis(2-ethynyl-iodophenyl)-4-(trifluoromethyl)aniline (2H-G3XB). 2H-G3XBBr (0.121 g, 0.233 mmol), copper iodide (0.005 g, 0.026 mmol), sodium iodide (0.140 g, 0.93 mmol) were added to a 10-20 mL microwave reaction vial containing a stir bar and dissolved in 13 mL 1,4-dioxane. To the vibrant yellow reaction mixture, trans-N,N'-dimethylcyclohexane-1,2-diamine (0.1 mL) was added. The microwave vial was sealed and placed in a Biotage Initiator+ microwave reactor for 12 hours at 150 ˚C. After cooling, the reaction was run through pipet silica plug with EtOAc to remove catalysts and salts. The EtOAc crude was then ran through GCMS in order to obtain % conversion of bromines to iodines. If the conversion from bromine to iodine was 99% or greater, then the reaction was concentrated. The beige/yellow product (0.100 g, 70%) was obtained after purified via high performance liquid chromatography (reverse phase column gradient from 80% water/20% acetonitrile to 100% acetonitrile).

^{1}H NMR (400 MHz, $CDCl_3$): δ (ppm) = 7.90 (d, J = 8.0 Hz, 2H), 7.64 (s, 2H), 7.57 (d, J = 7.7 Hz, 2H), 7.38 (t, J = 8.8 Hz, 2H), 7.06 (t, J = 5.8 Hz, 2H), 5.78 (s, 2H).

^{13}C NMR (100 MHz, $CDCl_3$): δ (ppm) = 152.08, 138.83, 132.85, 129.97, 129.72 (q, J = 3.9 Hz), 129.38, 128.25, 124.10 (q, J = 269.4 Hz), 119.35 (q, J = 33.5 Hz), 106.91, 100.71, 97.93, 88.27.

^{19}F NMR (376 MHz, $CDCl_3$): δ (ppm) = –60.00.

HRMS (ESI neg) m/z for $C_{23}H_{11}F_3I_2N^-$ [M-H$^+$]$^-$: calculated: 611.8938; found: 611.8938.

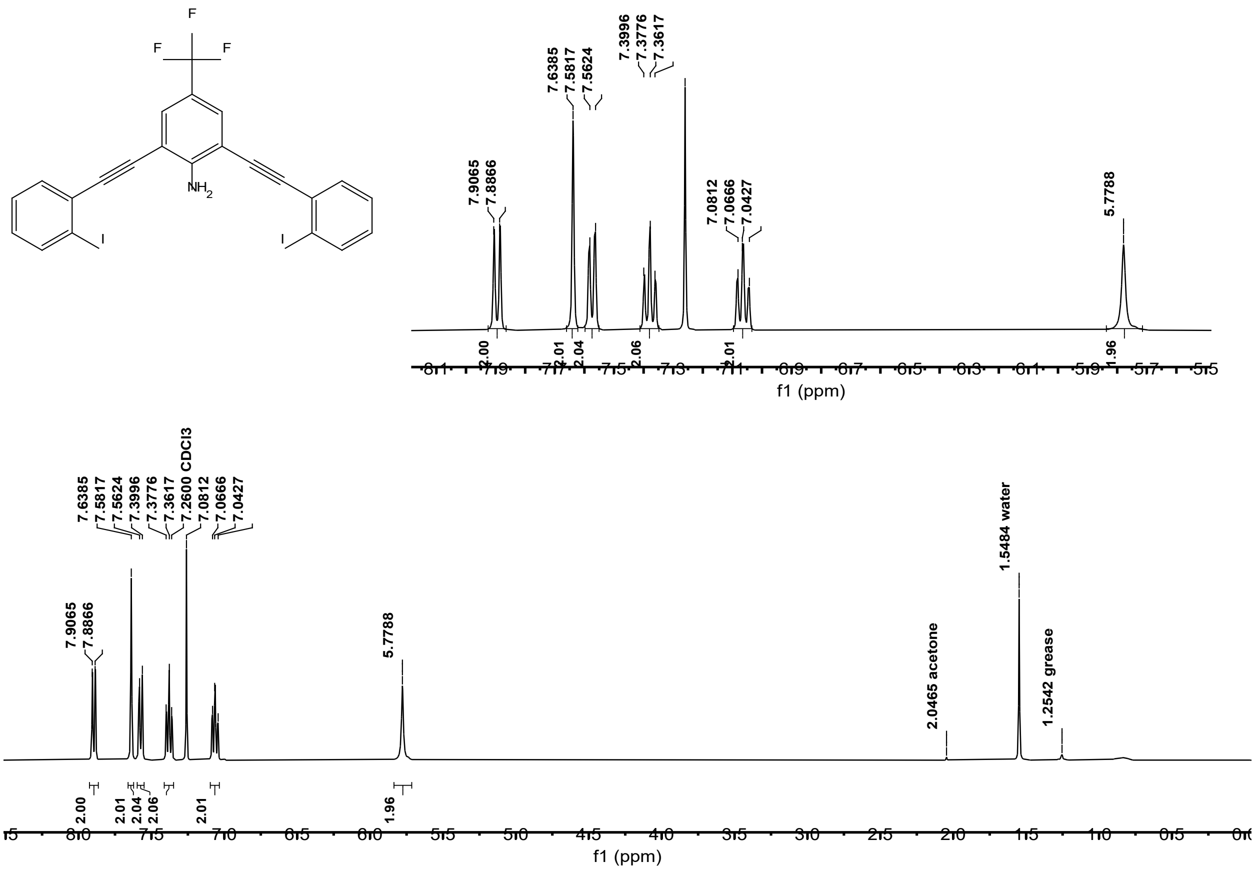

Figure S85. ^{1}H NMR of **2H-G3XB** (400 MHz, $CDCl_3$).

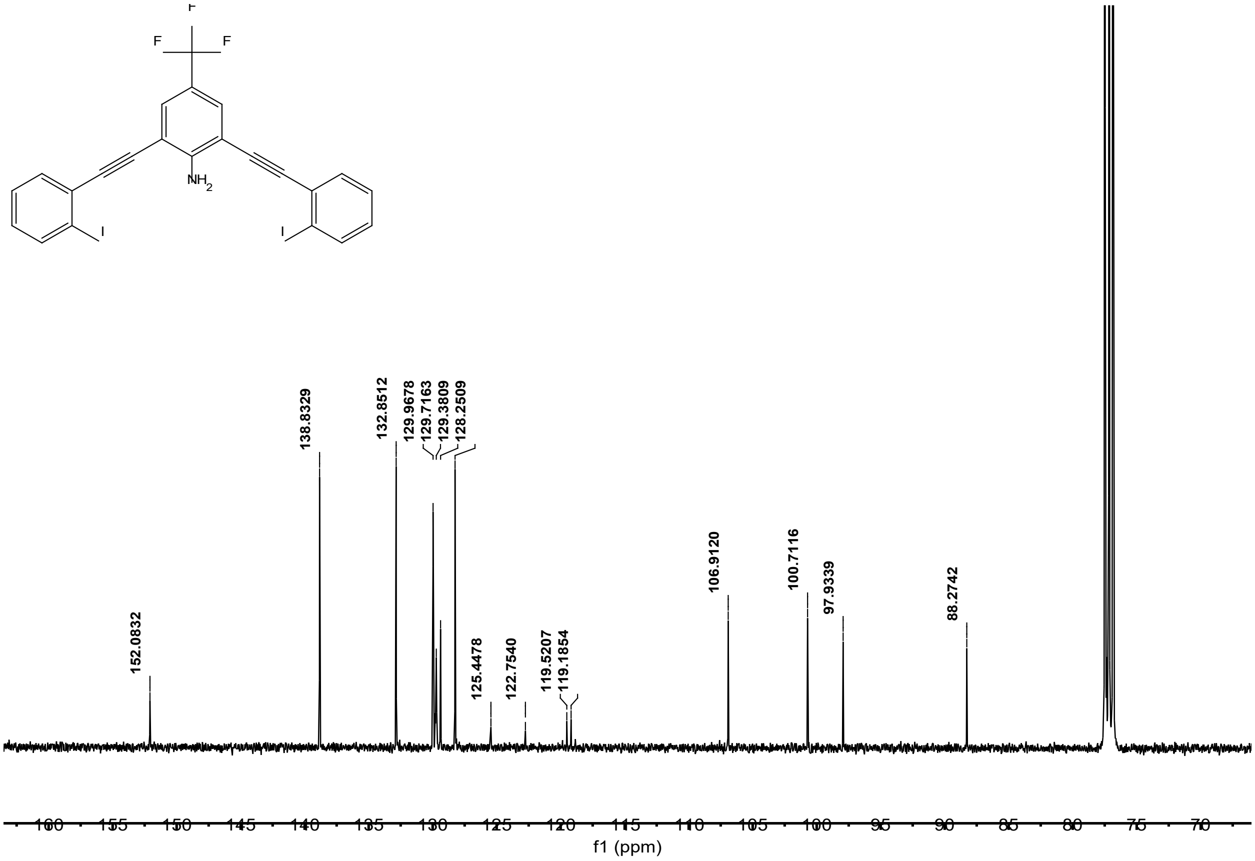

Figure S26. ^{13}C NMR of **2H-G3XB** (100 MHz, $CDCl_3$).

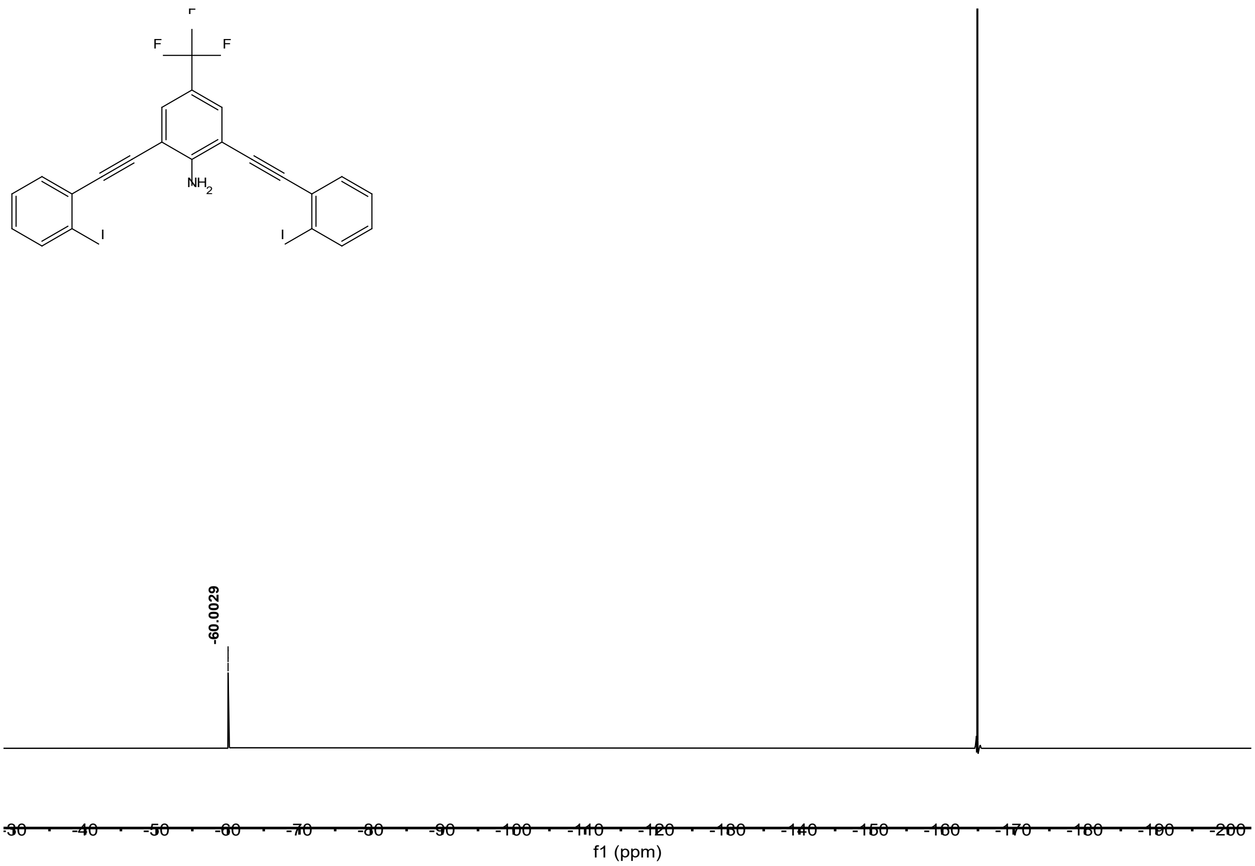

Figure S27. ^{19}F NMR of **2H-G3XB** (376 MHz, $CDCl_3$). Hexafluorobenzene (C_6F_6) internal reference.

2,6-bis(2-ethynyl-4-methyl-bromophenyl)-4-(trifluoromethyl)aniline (2Me-G3XBBr). To an oven dried Schlenk flask was charged with 2,6-bisethynyl-4-(trifluoromethyl)aniline **(2a)** (0.211g 1mmol), then vacuumed and backfilled with dry N_2 gas (3x). Bis(triphenylphosphine)palladium (II) dichloride (0.035 g, 0. 05 mmol) was added, vacuumed and backfilled with dry N_2 gas (3x). Copper (I) iodide (0.019 g, 0.1 mmol) was added, vacuumed and backfilled with dry N_2 (3x). 1-Bromo-2-iodo-4-methylbenzene (0.742 g, 2.5 mmol) was added, vacuumed and backfilled with dry N_2 gas (3x). The dry reagents were dissolved in 60 mL dry DMF. N,N-diisopropylethylamine (0.86 mL, 5 mmol) were added to the DMF solution. The flask was carefully vacuumed and backfilled with dry N_2 (3x). The dark brown solution stirred for 20 hours at room temperature. The reaction mixture was extracted with ethyl acetate then washed with water to remove any excess salts and catalysts, then was dried over magnesium sulfate and gravity filtered. Subsequent removal of DMF, hexanes and ethyl acetate by rotary evaporation left and brown solid that was purified by column chromatography (gradient from 100% Hexanes to 3% EtOAc/97% Hexanes) to afford **2Me-G3XBBr** (0.375 g, 69%) as a dull yellow solid.

^{1}H NMR (400 MHz, $CDCl_3$): δ (ppm) = 7.61 (s, 2H), 7.52-7.50 (d, J = 8.3 Hz, 2H), 7.41 (s, 2H), 7.04-7.02 (d, J = 8.2 Hz, 2H), 5.69 (s, 2H), 2.33 (s, 6H).

^{13}C NMR (100 MHz, CDCl$_3$): δ (ppm) = 152.00, 137.53, 133.80, 132.24, 131.04, 129.47 (q, J = 3.7 Hz), 124.53, 124.13 (q, J = 269.4 Hz), 121.96, 119.40 (q, J = 34.0 Hz), 106.96, 94.90, 88.67, 20.92.

^{19}F NMR (376 MHz, CDCl$_3$): δ (ppm) = –59.99.

HRMS (ESI neg) m/z for $C_{25}H_{15}F_3Br_2N^-$ [M-H$^+$]$^-$: calculated: 545.9508; found: 545.9508.

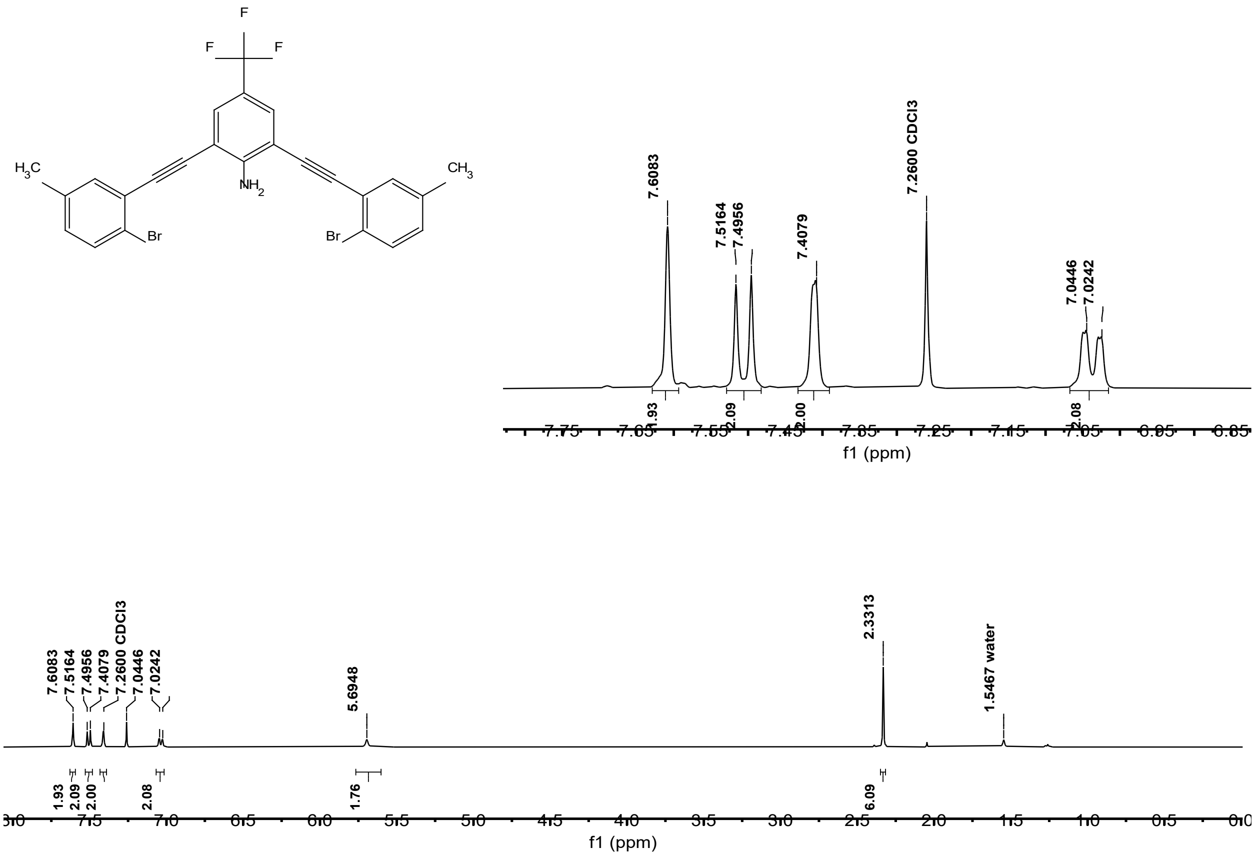

Figure S98. ^{1}H NMR of **2Me-G3XBBr** (400 MHz, CDCl$_3$).

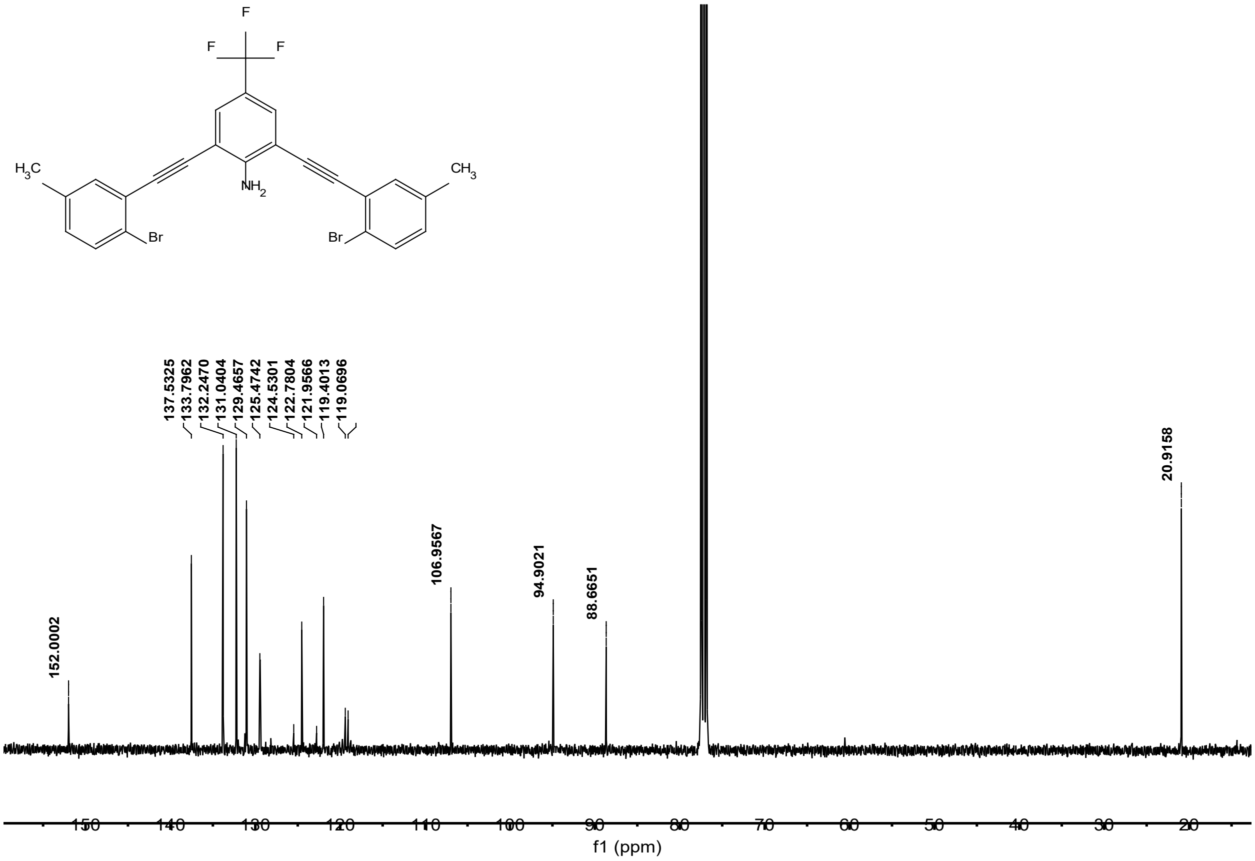

Figure S29. ^{13}C NMR of **2Me-G3XBBr** (100 MHz, $CDCl_3$).

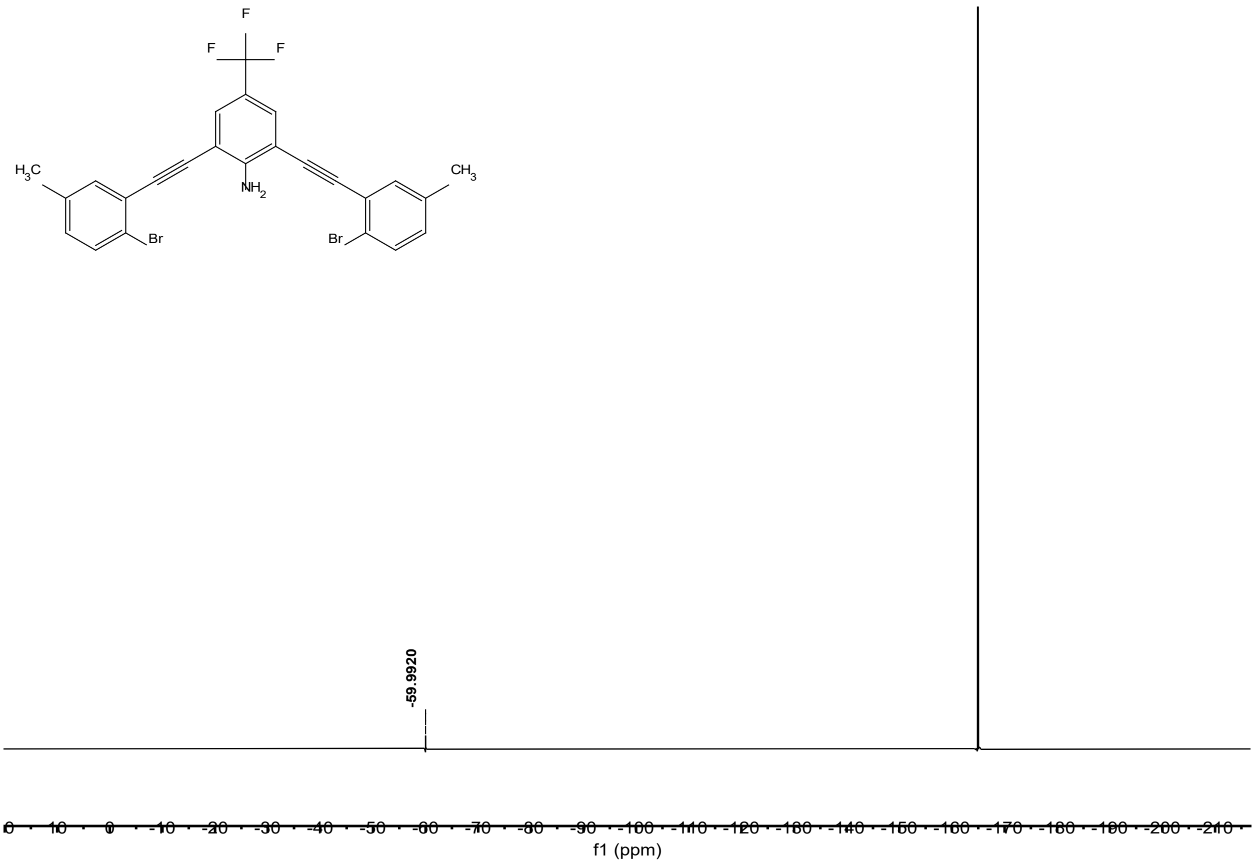

Figure S30. ^{19}F NMR of **2Me-G3XBBr** (376 MHz, $CDCl_3$). Hexafluorobenzene (C_6F_6) internal reference.

2,6-bis(2-ethynyl-4-methyl-iodophenyl)-4-(trifluoromethyl)aniline (2Me-G3XB). 2Me-G3XBBr (0.100 g, 0.183 mmol), copper iodide (0.0035 g, 0.018 mmol), sodium iodide (0.110 g, 0.732 mmol) were added to a 10-20 mL microwave reaction vial containing a stir bar and dissolved in 13 mL 1,4-dioxane. To the vibrant yellow reaction mixture, trans-N,N'-dimethylcyclohexane-1,2-diamine (0.1 mL) was added. The microwave vial was sealed and placed in a Biotage Initiator+ microwave reactor for 24 hours at 150 ˚C. After cooling, the reaction was run through pipet silica plug with EtOAc to remove catalysts and salts. The EtOAc crude was then ran through GCMS in order to obtain % conversion of bromines to iodines. If the conversion from bromine to iodine was 99% or greater, then the reaction was concentrated. The beige/yellow product (0.081 g, 69%) was obtained after purified via high performance liquid chromatography (reverse phase column gradient from 80% water/20% acetonitrile to 100% acetonitrile).

^{1}H NMR (400 MHz, $CDCl_3$): δ (ppm) = 7.74 (d, J = 8.2 Hz, 2H), 7.62 (s, 2H), 7.41 (s, 2H), 6.88 (d, J = 10.7 Hz, 2H), 5.78 (s, 2H), 2.32 (s, 6H).

^{13}C NMR (100 MHz, $CDCl_3$): δ (ppm) = 152.08, 138.54, 138.38, 133.55, 131.20, 129.62 (q, J = 3.9 Hz), 129.09, 124.13 (q, J = 269.4 Hz), 119.46 (q, J = 33.9 Hz), 106.97, 98.03, 96.60, 87.88, 20.98.

^{19}F NMR (376 MHz, $CDCl_3$): δ (ppm) = –59.99.

HRMS (ESI neg) m/z for $C_{25}H_{15}F_3I_2N^-$ [M-H$^+$]$^-$: calculated: 639.9251; found: 639.9252.

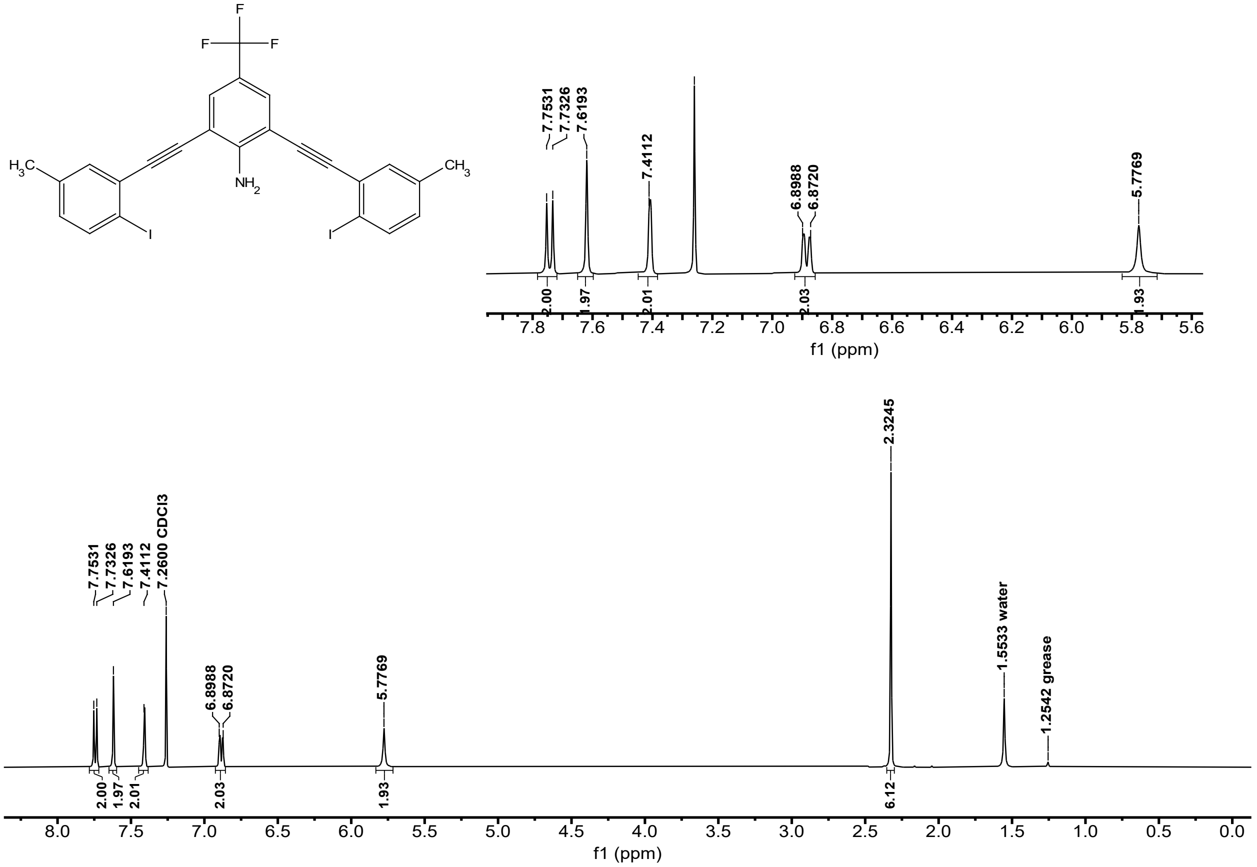

Figure S31. ^{1}H NMR of **2Me-G3XB** (400 MHz, $CDCl_3$).

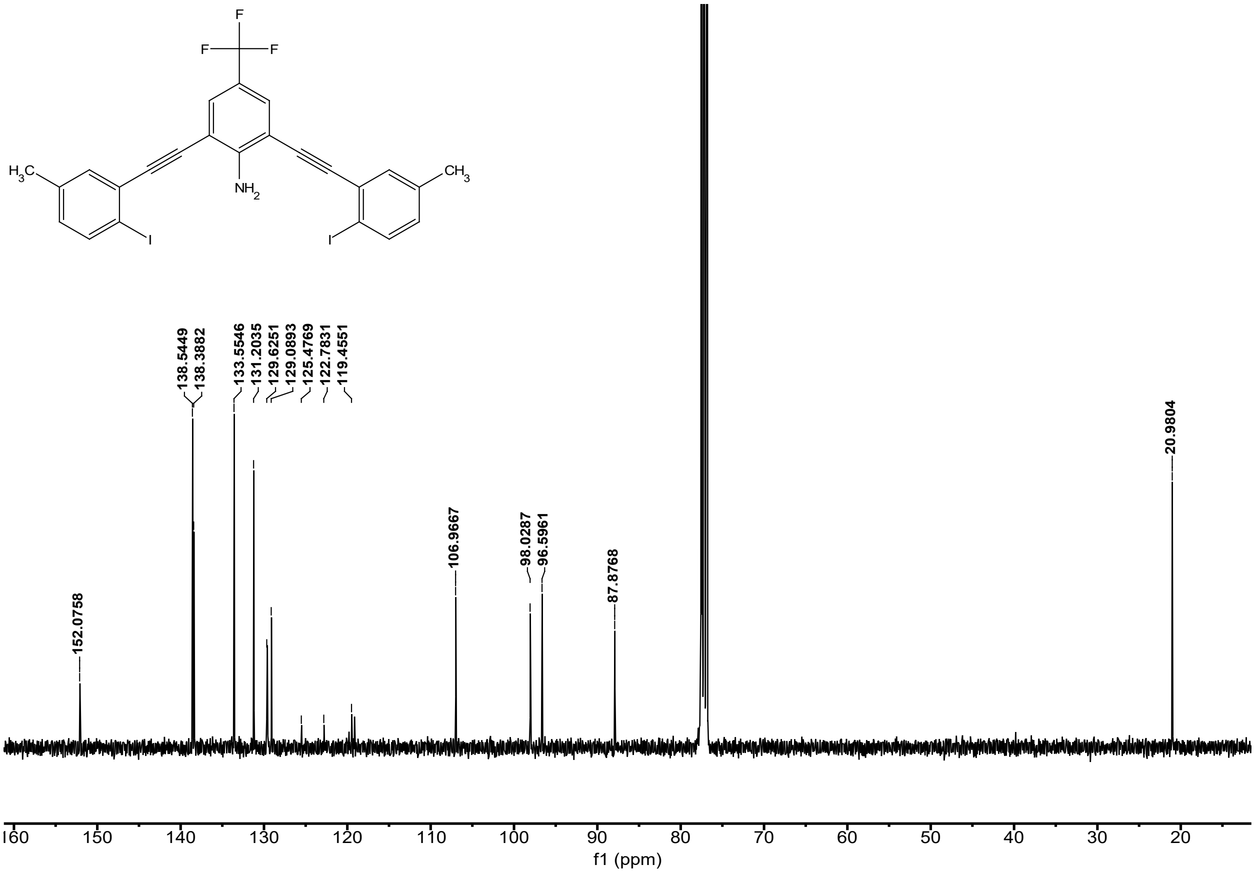

Figure S32. ^{13}C NMR of **2Me-G3XB** (100 MHz, $CDCl_3$).

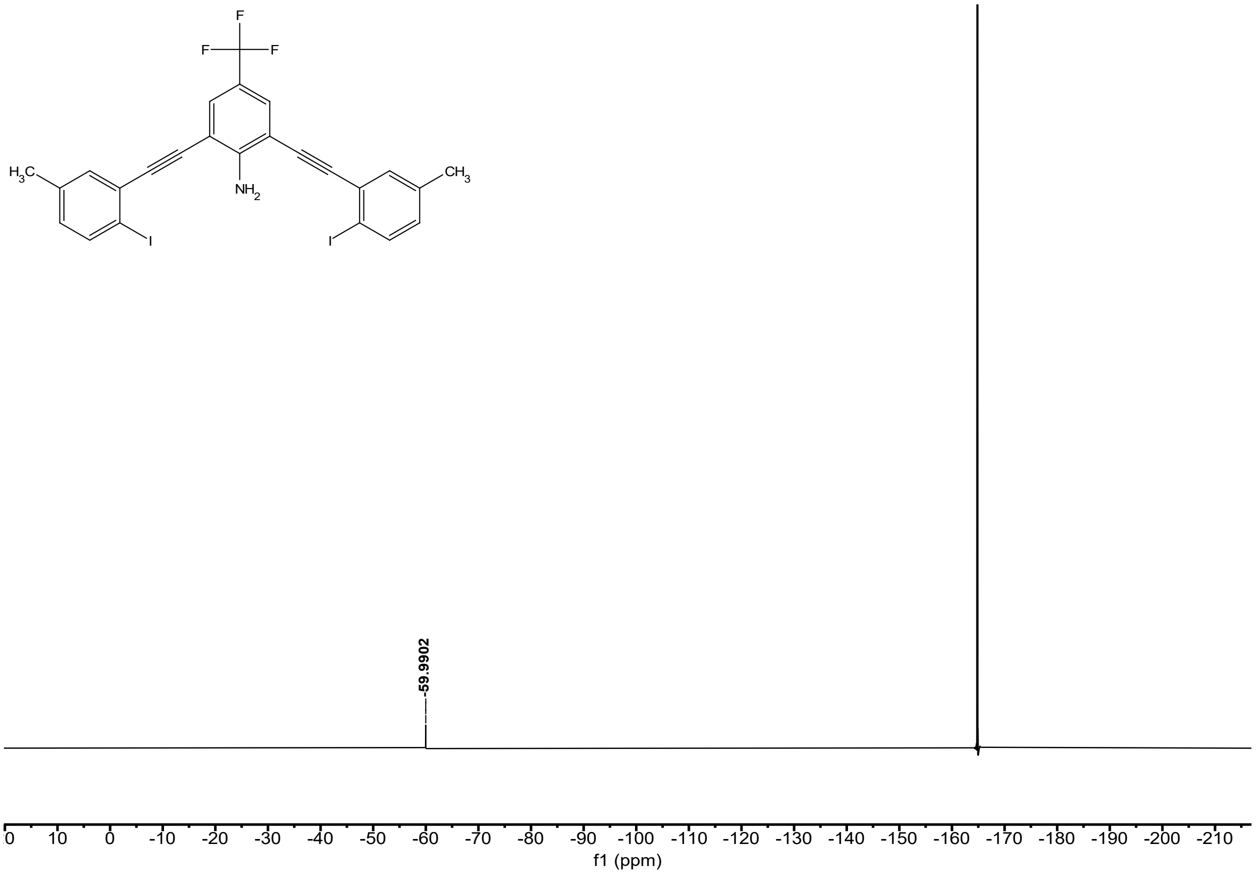

Figure S33. ^{19}F NMR of **2Me-G3XB** (376 MHz, $CDCl_3$). Hexafluorobenzene (C_6F_6) internal reference.

2,6-bis(2-ethynyl-4-*tert*-butyl-bromophenyl)-4-(trifluoromethyl)aniline (2*t*Bu-G3XBBr). To an oven dried Schlenk flask was charged with 2,6-bisethynyl-4-(trifluoromethyl)aniline **(2a)** (0.633g 3mmol), then vacuumed and backfilled with dry N_2 gas (3x). Bis(triphenylphosphine)palladium (II) dichloride (0.105 g, 0. 15 mmol) was added, vacuumed and backfilled with dry N_2 gas (3x). Copper (I) iodide (0.057 g, 0.3 mmol) was added, vacuumed and backfilled with dry N_2 (3x). 1-Bromo-2-iodo-4-*tert*-butylbenzene (1.015 g, 7.5 mmol) was added, vacuumed and backfilled with dry N_2 gas (3x). The dry reagents were dissolved in 60 mL dry DMF. N,N-diisopropylethylamine (2.58 mL, 15 mmol) were added to the DMF solution. The flask was carefully vacuumed and backfilled with dry N_2 (3x). The dark brown solution stirred for 24 hours at room temperature. The reaction mixture was extracted with ethyl acetate then washed with water to remove any excess salts and catalysts, then was dried over magnesium sulfate and gravity filtered. Subsequent removal of DMF, hexanes and ethyl acetate by rotary evaporation left and brown solid that was purified by column chromatography (gradient from 100% Hexanes to 3% EtOAc/97% Hexanes) to afford **2*t*Bu-G3XBBr** (0.9 g, 50%) as a white solid.

^{1}H NMR (400 MHz, $CDCl_3$): δ (ppm) = 7.65 (s, 2H), 7.59 (d, J = 2.3 Hz, 2H), 7. 55 (d, J = 8.5 Hz, 2H), 7. 26 (dd, J = 8.5 Hz, 2H), 5.72 (s, 2H), 1.34 (s, 18H).

^{13}C NMR (100 MHz, CDCl$_3$): δ (ppm) = 152.01, 150.89, 132.08, 130.42, 129.47 (q, J = 3.7 Hz), 127.60, 124.28, 124.15 (q, J = 269.3 Hz), 122.07, 119.38 (q, J = 33.2 Hz), 106.97, 95.25, 88.41, 34.80, 31.25.

^{19}F NMR (376 MHz, CDCl$_3$): δ (ppm) = –59.95.

HRMS (ESI neg) m/z for $C_{31}H_{27}F_3Br_2N^-$ [M-H$^+$]$^-$: calculated: 545.9508; found: 545.9508.

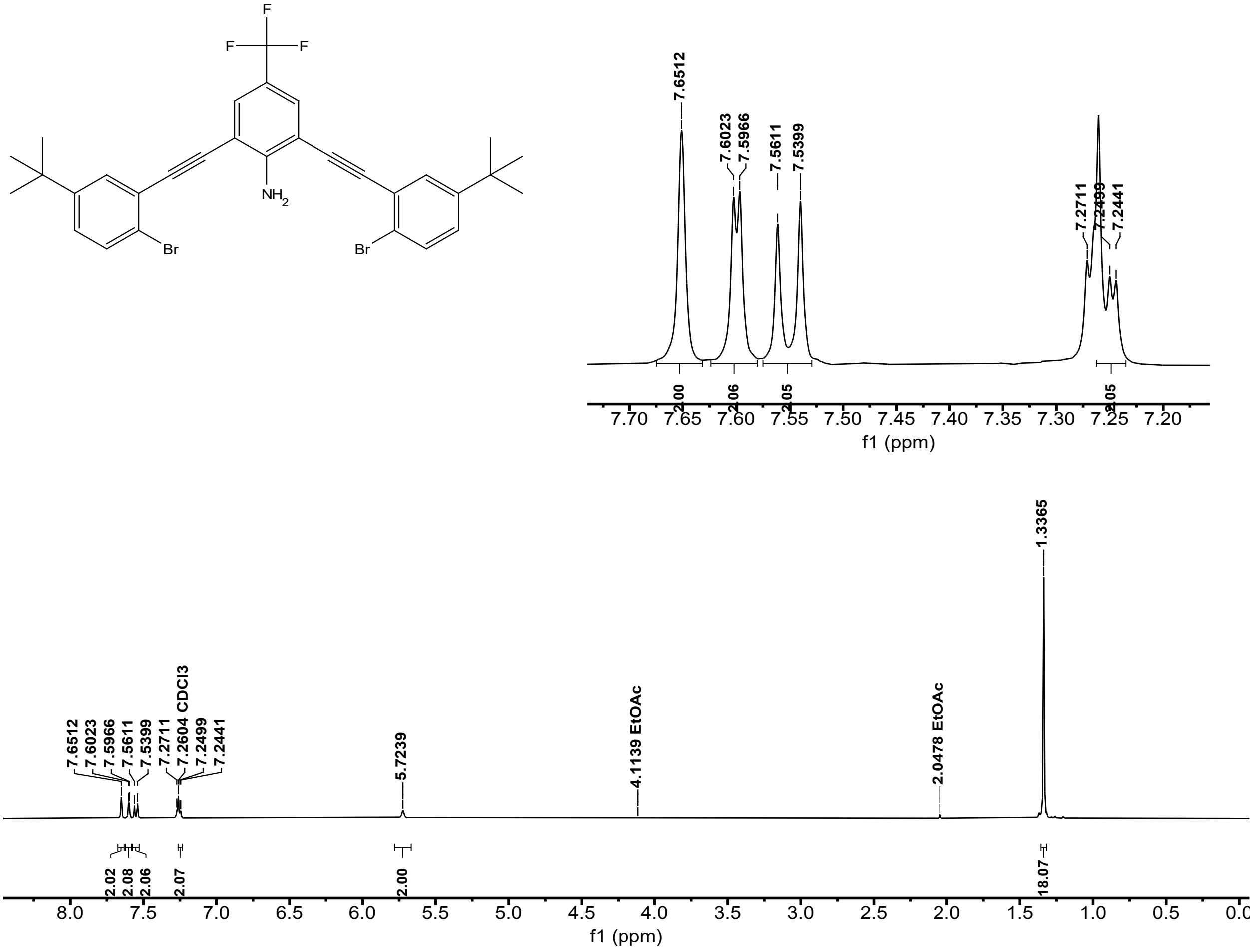

Figure S34. ^{1}H NMR of **2*t*Bu-G3XBBr** (400 MHz, CDCl$_3$).

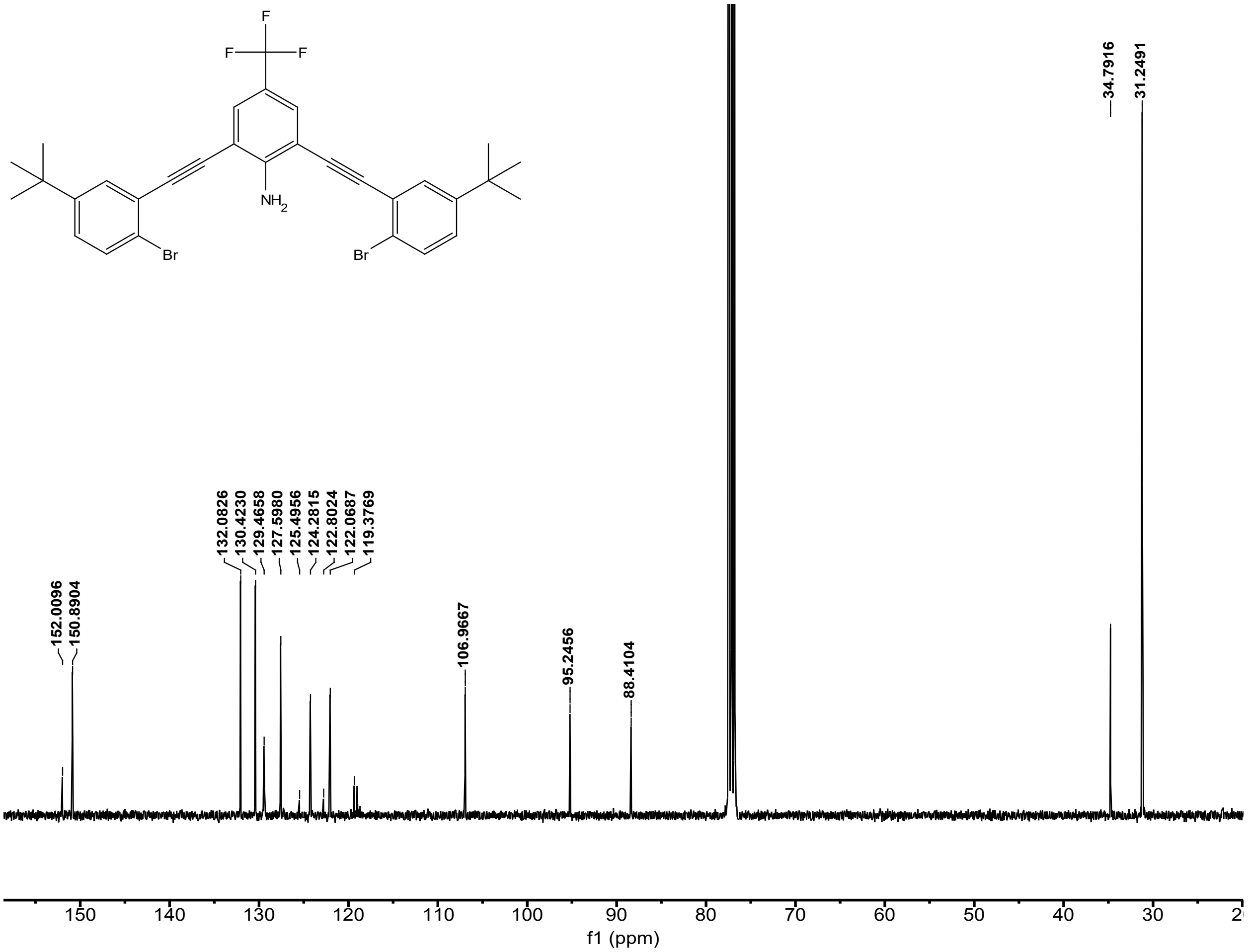

Figure S35. ^{13}C NMR of **2*t*Bu-G3XBBr** (100 MHz, $CDCl_3$).

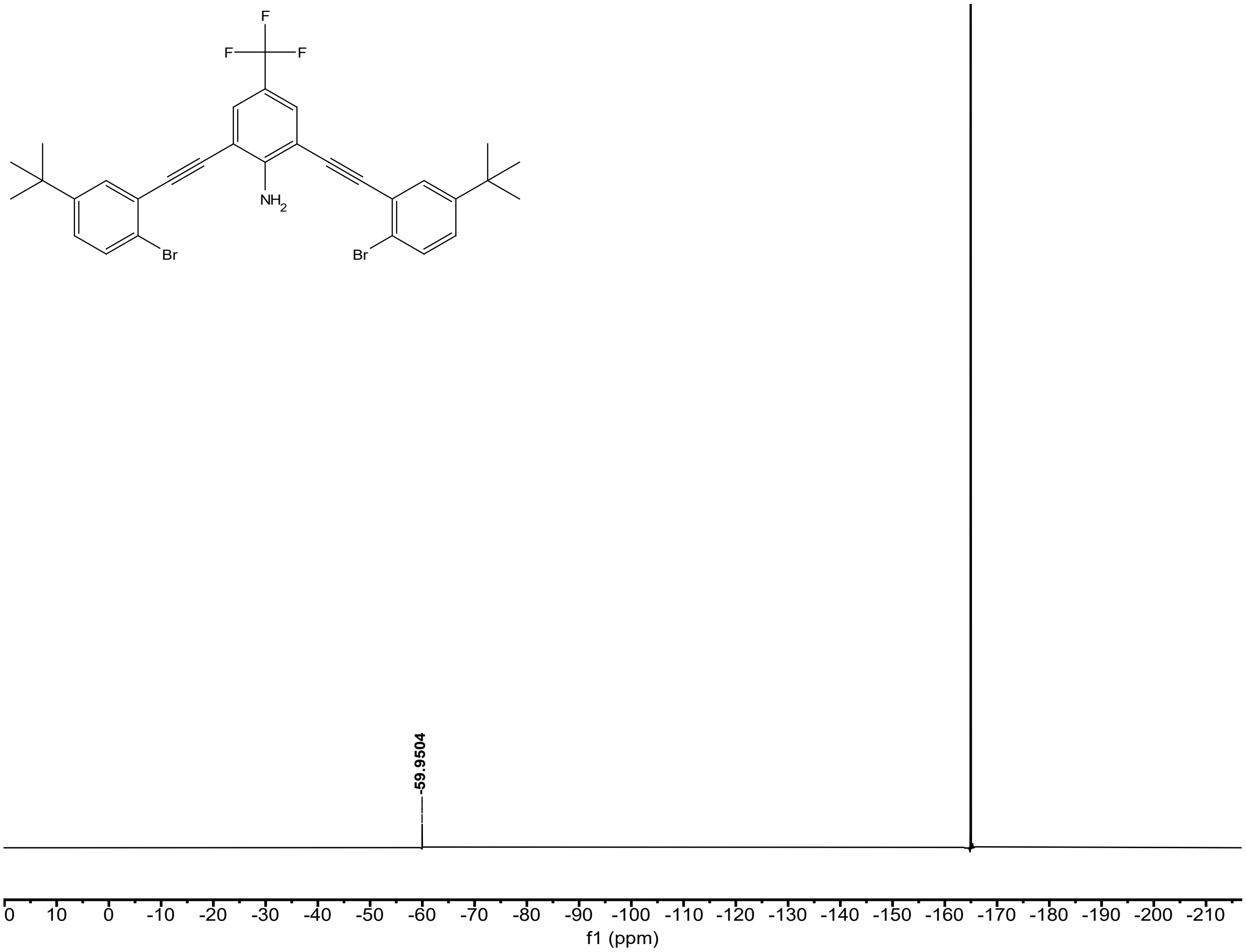

Figure S36. ^{19}F NMR of **2*t*Bu-G3XBBr** (376 MHz, $CDCl_3$). Hexafluorobenzene (C_6F_6) internal reference.

2,6-bis(2-ethynyl-4-*tert*-butyl-iodophenyl)-4-(trifluoromethyl)aniline (2*t*Bu-G3XB). 2*t*Bu-G3XBBr (0.200 g, 0.317 mmol), copper iodide (0.0060 g, 0.032 mmol), sodium iodide (0.190 g, 1.267 mmol) were added to a 10-20 mL microwave reaction vial containing a stir bar and dissolved in 13 mL 1,4-dioxane. To the vibrant yellow reaction mixture, trans-N,N'-dimethylcyclohexane-1,2-diamine (0.1 mL) was added. The microwave vial was sealed and placed in a Biotage Initiator+ microwave reactor for 64 hours at 160˚C. After cooling, the reaction was run through pipet silica plug with EtOAc to remove catalysts and salts. The EtOAc crude was then ran through GCMS in order to obtain % conversion of bromines to iodines. If the conversion from bromine to iodine was 99% or greater, then the reaction was concentrated. The white product (0.154 g, 67%) was obtained after purified via high performance liquid chromatography (reverse phase column gradient from 80% water/20% acetonitrile to 100% acetonitrile).

^{1}H NMR (400 MHz, $CDCl_3$): δ (ppm) = 7.79 (d, J = 8.4 Hz, 2H), 7.66 (s, 2H), 7.59 (d, J = 2.2 Hz, 2H), 7.10 (dd, J = 8.4 Hz, 2H), 5.81 (s, 2H), 1.33 (s, 18H).

^{13}C NMR (100 MHz, CDCl$_3$): δ (ppm) = 152.10, 151.77, 138.39, 130.15, 129.63 (q, J = 3.7 Hz), 128.86, 127.73, 124.15 (q, J = 269.3 Hz), 119.42 (q, J = 33.2 Hz), 106.97, 98.35, 96.93, 87.61, 34.83, 31.20.

^{19}F NMR (376 MHz, CDCl$_3$): δ (ppm) = –59.92.

HRMS (ESI neg) m/z for $C_{31}H_{27}F_3I_2N^-$ [M-H$^+$]$^-$: calculated: 724.0190; found: 724.0194.

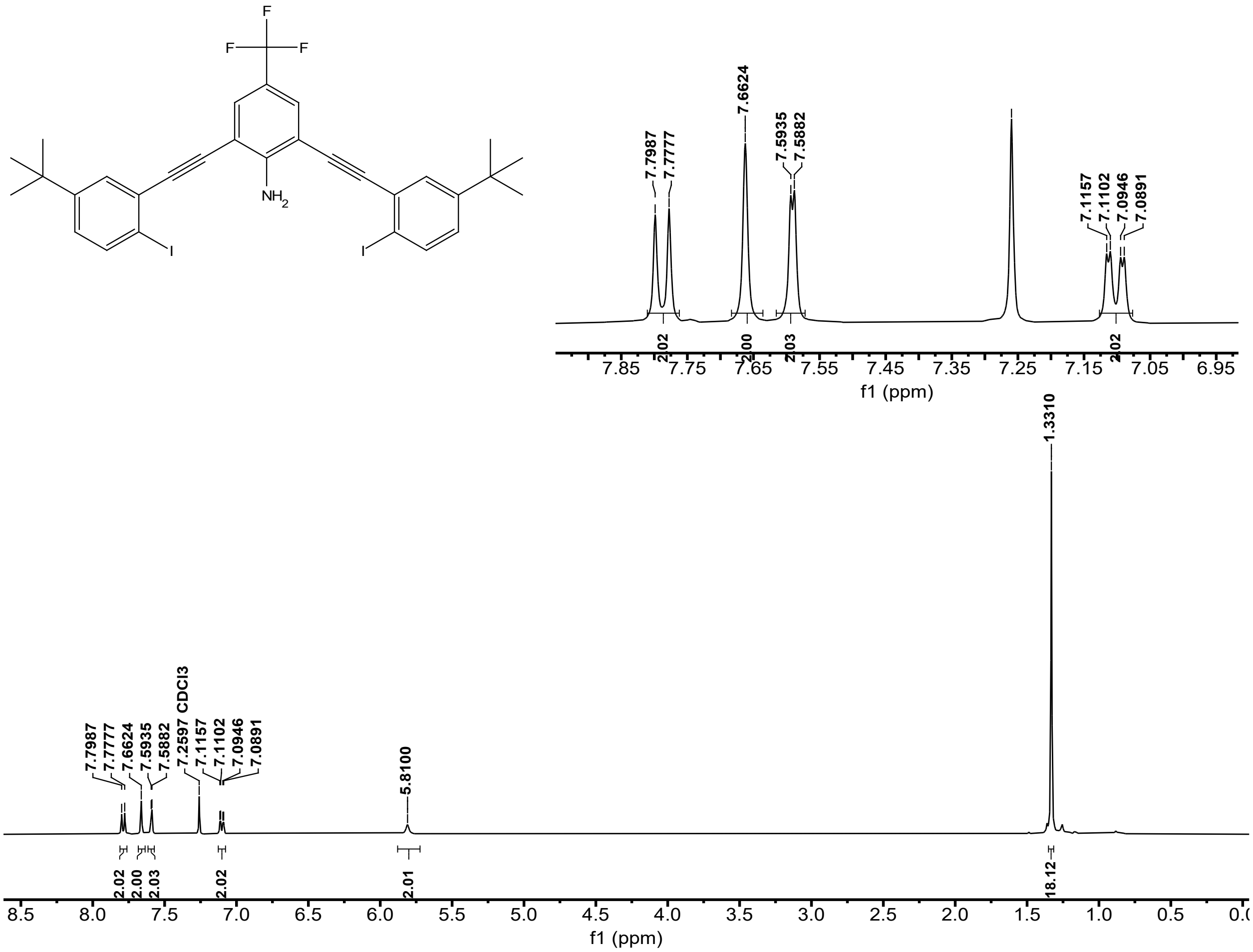

Figure S37. ^{1}H NMR of **2*t*Bu-G3XB** (400 MHz, CDCl$_3$).

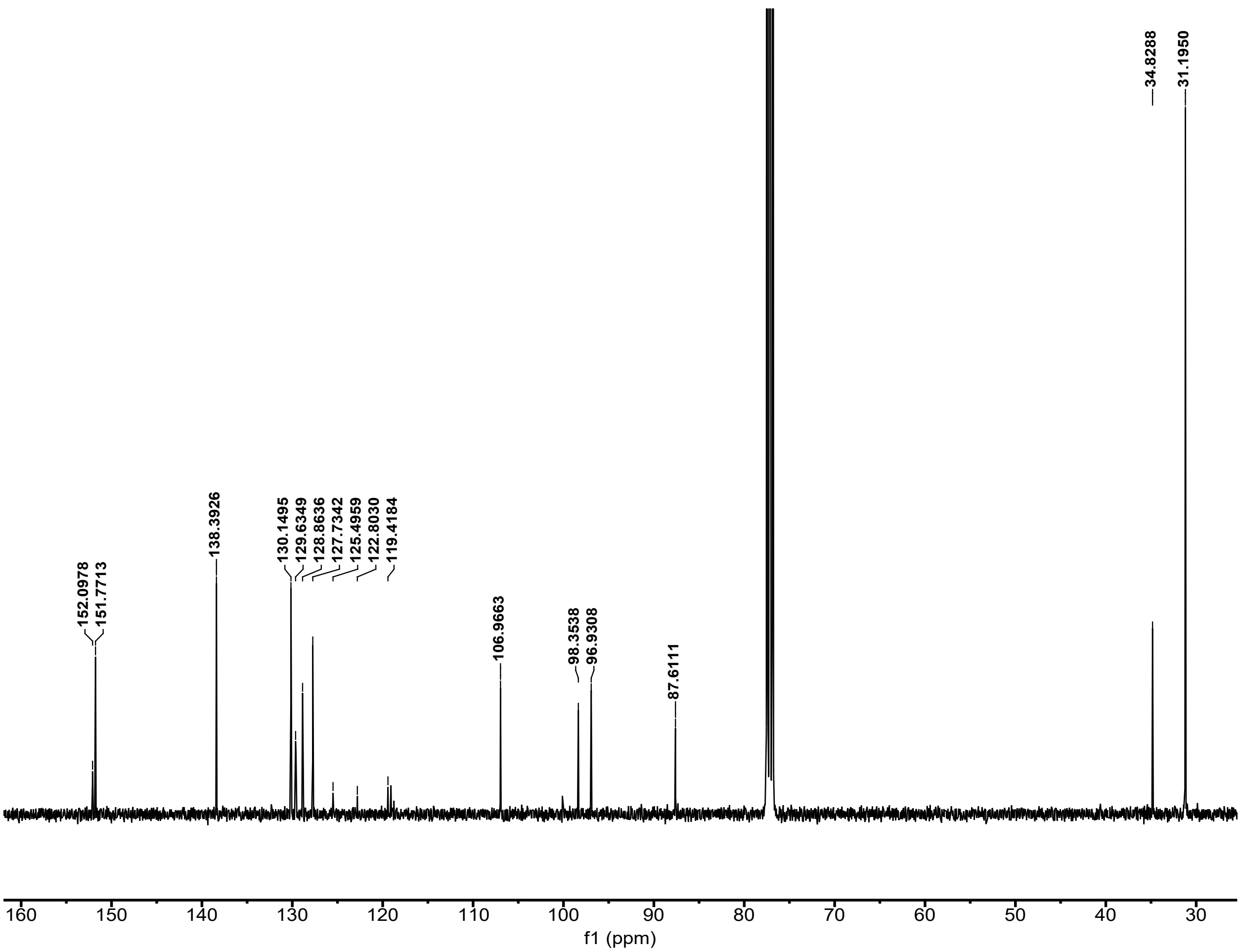

Figure S38. ^{13}C NMR of **2*t*Bu-G3XB** (100 MHz, $CDCl_3$).

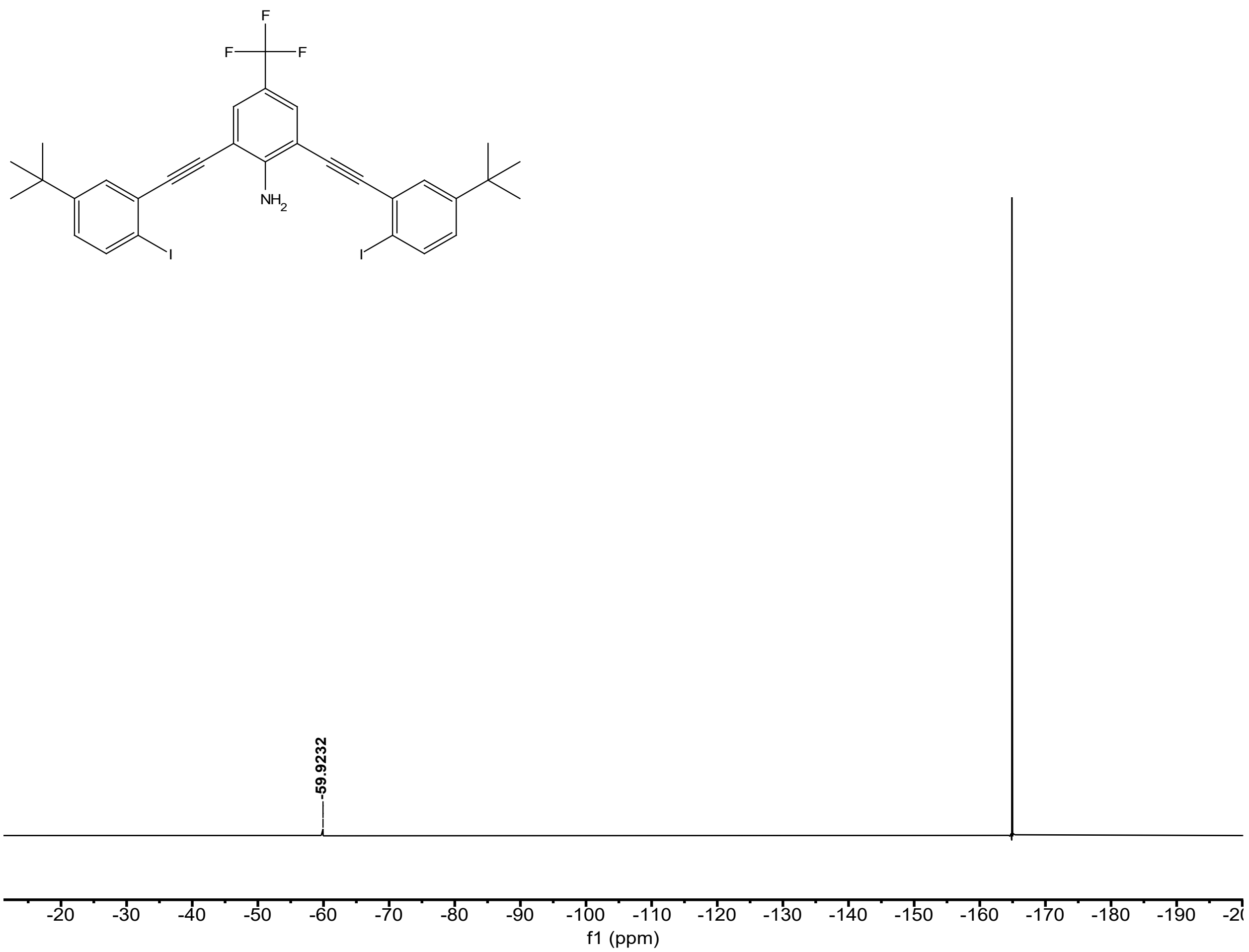

Figure S39. ^{19}F NMR of **2*t*Bu-G3XB** (376 MHz, $CDCl_3$). Hexafluorobenzene (C_6F_6) internal reference.

2,6-bis(2-ethynyl-4-methoxy-bromophenyl)-4-(trifluoromethyl)aniline (2MeO-G3XBBr). To an oven dried Schlenk flask was charged with 2,6-bisethynyl-4-(trifluoromethyl)aniline **(2a)** (0.211g 1mmol), then vacuumed and backfilled with dry N_2 gas (3x). Bis(triphenylphosphine)palladium (II) dichloride (0.035 g, 0. 05 mmol) was added, vacuumed and backfilled with dry N_2 gas (3x). Copper (I) iodide (0.019 g, 0.1 mmol) was added, vacuumed and backfilled with dry N_2 (3x). The dry reagents were dissolved in 60 mL dry DMF. 1-Bromo-2-iodo-4-methoxybenzene (0.38 mL, 2.5 mmol) and N,N-diisopropylethylamine (0.86 mL, 5 mmol) were added to the DMF solution. The flask was carefully vacuumed and backfilled with dry N_2 (3x). The dark brown solution stirred for 20 hours at room temperature. The reaction mixture was extracted with ethyl acetate then washed with water to remove any excess salts and catalysts, then was dried over magnesium sulfate and gravity filtered. Subsequent removal of DMF, hexanes and ethyl acetate by rotary evaporation left and brown solid that was purified by column chromatography (gradient from 100% Hexanes to 3% EtOAc/97% Hexanes) to afford **2MeO-G3XBBr** (0.357 g, 62%) as a dull yellow solid.

^{1}H NMR (400 MHz, CDCl$_3$): δ (ppm) = 7.63 (s, 2H), 7.50 (d, J = 8.9 Hz, 2H), 7.110 (d, J = 3.0 Hz, 2H), 6.81 (dd, J = 8.9 Hz, 2H), 5.72 (s, 2H), 3.83 (s, 6H).

^{13}C NMR (100 MHz, CDCl$_3$): δ (ppm) = 158.82, 152.11, 133.20, 129.62 (q, J = 3.6 Hz), 125.39, 124.09 (q, J = 269.3 Hz), 119.26 (q, J = 33.0 Hz), 117.68, 117.18, 115.97, 106.80, 94.80, 88.83, 55.80.

^{19}F NMR (376 MHz, CDCl$_3$): δ (ppm) = –59.99.

HRMS (ESI neg) m/z for $C_{25}H_{15}F_3Br_2NO_2^-$ [M-H$^+$]$^-$: calculated: 577.9407; found: 577.9406.

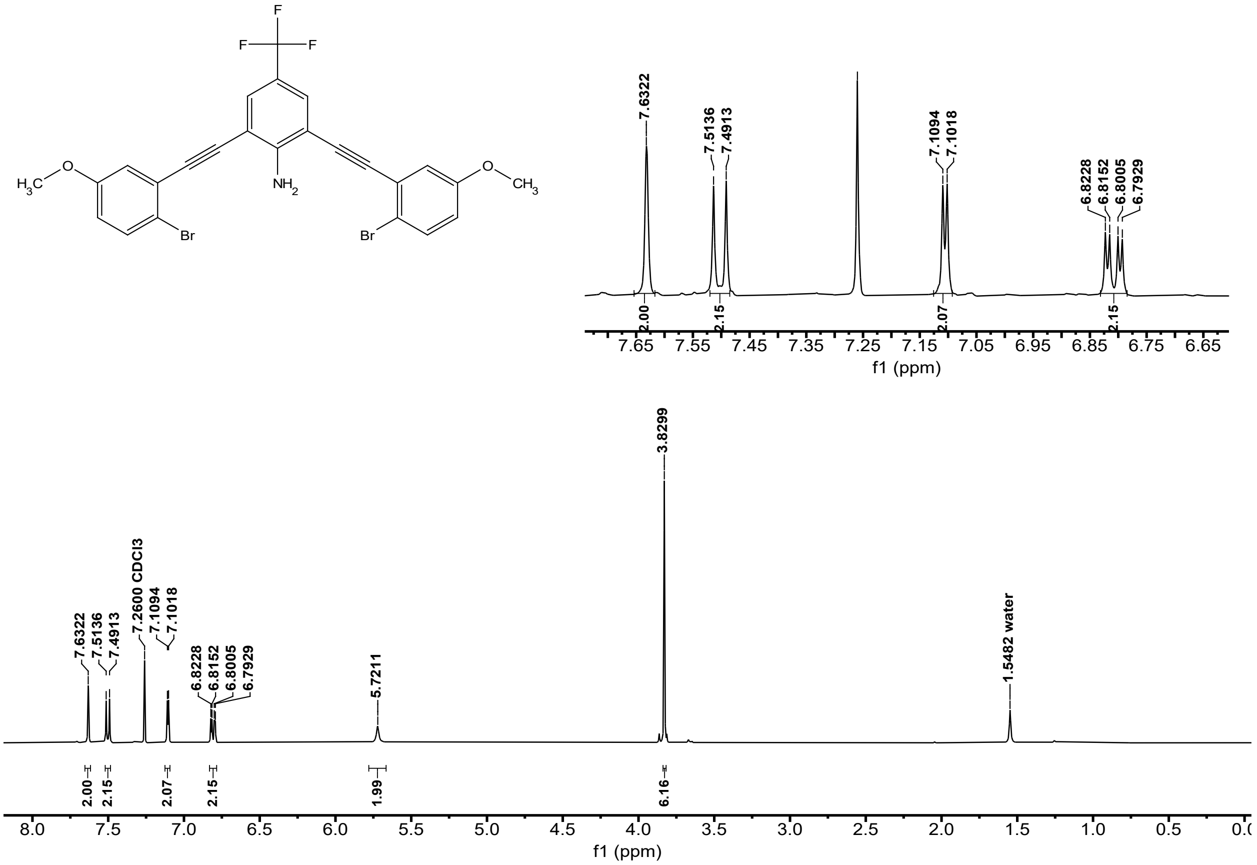

Figure S40. ^{1}H NMR of **2MeO-G3XBBr** (400 MHz, CDCl$_3$).

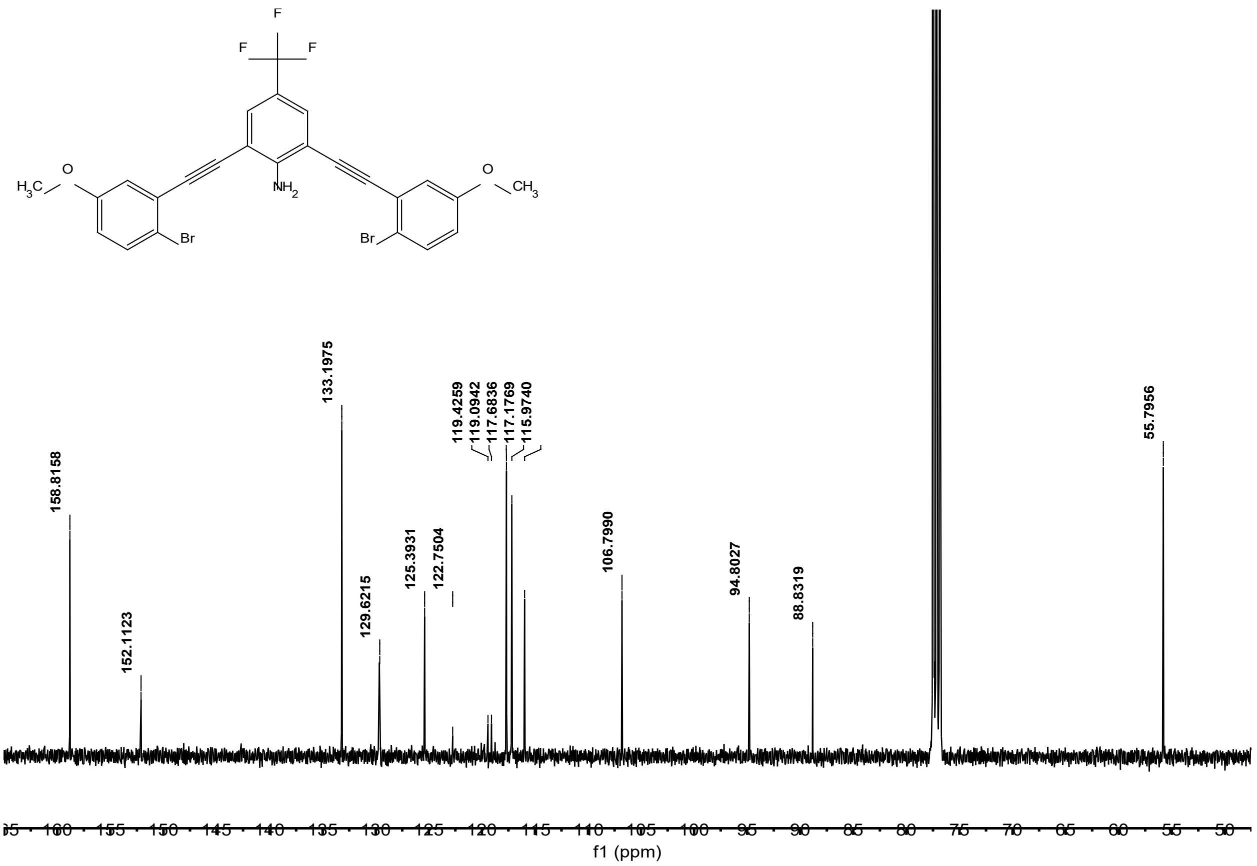

Figure S41. ^{13}C NMR of **2MeO-G3XBBr** (100 MHz, $CDCl_3$).

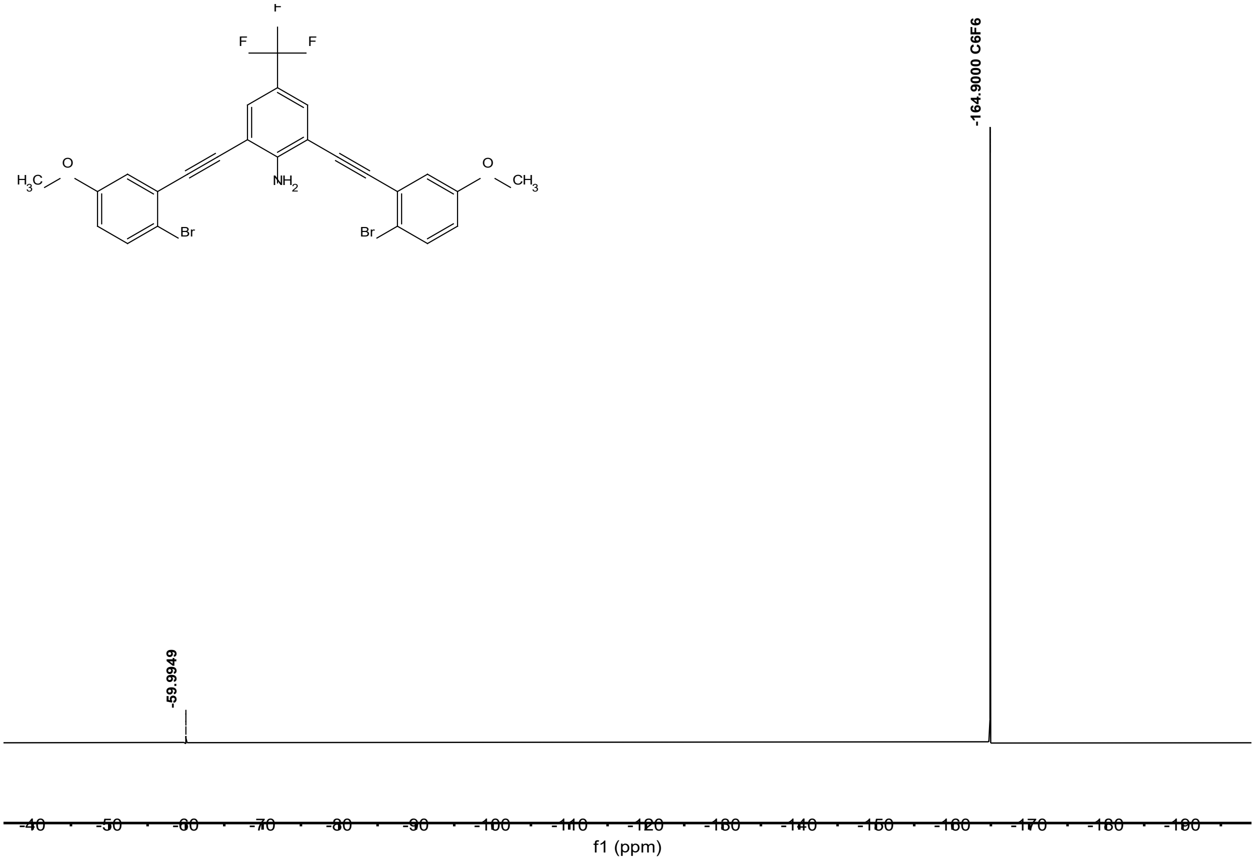

Figure S42. ^{19}F NMR of **2MeO-G3XBBr** (376 MHz, $CDCl_3$). Hexafluorobenzene (C_6F_6) internal reference.

2,6-bis(2-ethynyl-4-methoxy-iodophenyl)-4-(trifluoromethyl)aniline (2MeO-G3XB). 2MeO-G3XBBr (0.100 g, 0.173 mmol), copper iodide (0.0033 g, 0.017 mmol), sodium iodide (0.104 g, 0.692 mmol) were added to a 10-20 mL microwave reaction vial containing a stir bar and dissolved in 13 mL 1,4-dioxane. To the vibrant yellow reaction mixture, trans-N,N'-dimethylcyclohexane-1,2-diamine (0.1 mL) was added. The microwave vial was sealed and placed in a Biotage Initiator+ microwave reactor for 12 hours at 150 ˚C. After cooling, the reaction was run through pipet silica plug with EtOAc to remove catalysts and salts. The EtOAc crude was then ran through GCMS in order to obtain % conversion of bromines to iodines. If the conversion from bromine to iodine was 99% or greater, then the reaction was concentrated. The beige/yellow product (0.98 g, 84.4%) was obtained after purified via high performance liquid chromatography (reverse phase column gradient from 80% water/20% acetonitrile to 100% acetonitrile).

^{1}H NMR (400 MHz, $CDCl_3$): δ (ppm) = 7.72 (d, J = 8.9 Hz, 2H), 7.64 (s, 2H), 7.12 (d, J = 3.0 Hz, 2H), 6.68 (dd, J = 8.8 Hz, 2H), 5.81 (s, 2H), 3.83 (s, 6H).

^{13}C NMR (100 MHz, $CDCl_3$): δ (ppm) = 159.76, 152.19, 139.38, 129.96, 129.78 (q, J = 3.6 Hz), 124.10 (q, J = 269.4 Hz), 119.46 (q, J = 33.4 Hz), 117.71, 117.54, 106.80, 97.86, 89.40, 88.03, 55.69.

^{19}F NMR (376 MHz, $CDCl_3$): δ (ppm) = –59.98.

HRMS (ESI neg) m/z for $C_{25}H_{15}F_3I_2NO_2^-$ [M-H$^+$]$^-$: calculated: 671.9150; found: 671.9150.

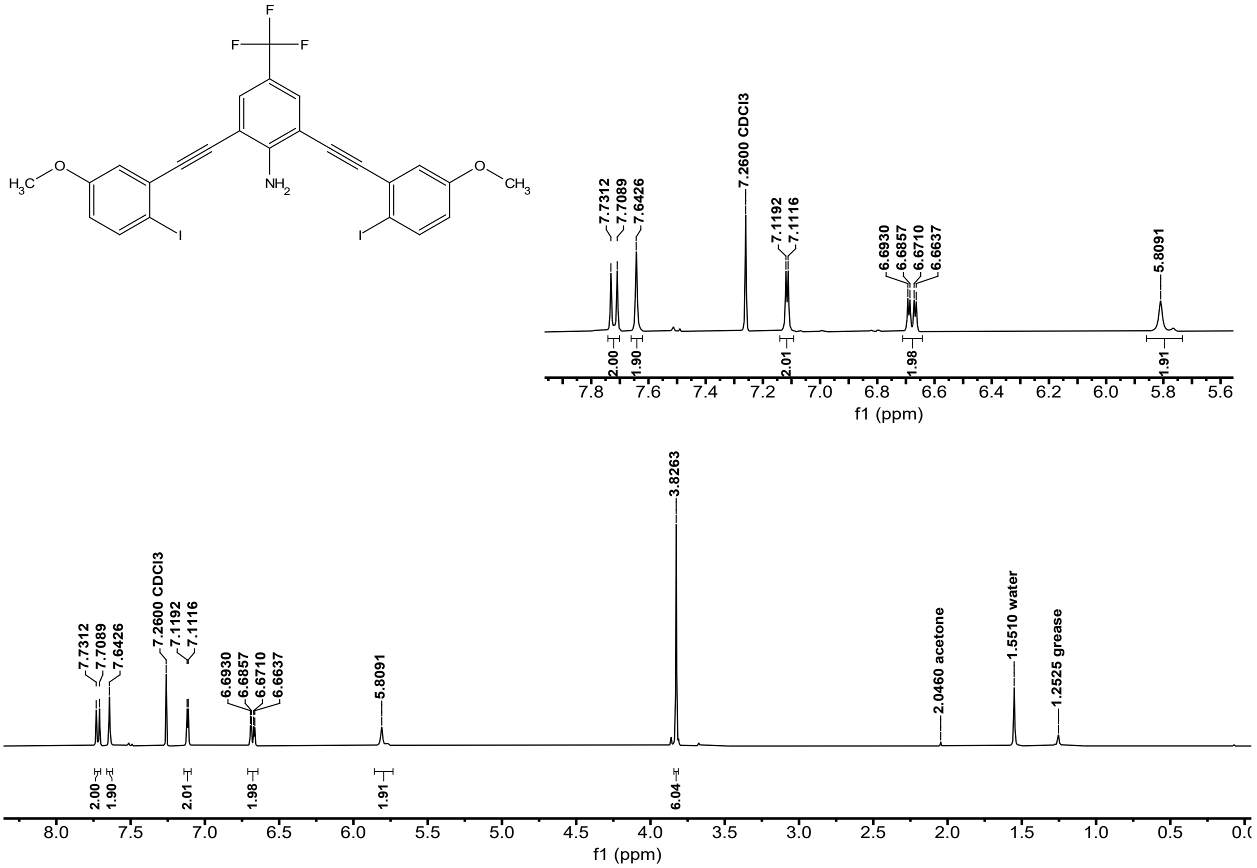

Figure S43. ^{1}H NMR of **2MeO-G3XB** (400 MHz, $CDCl_3$).

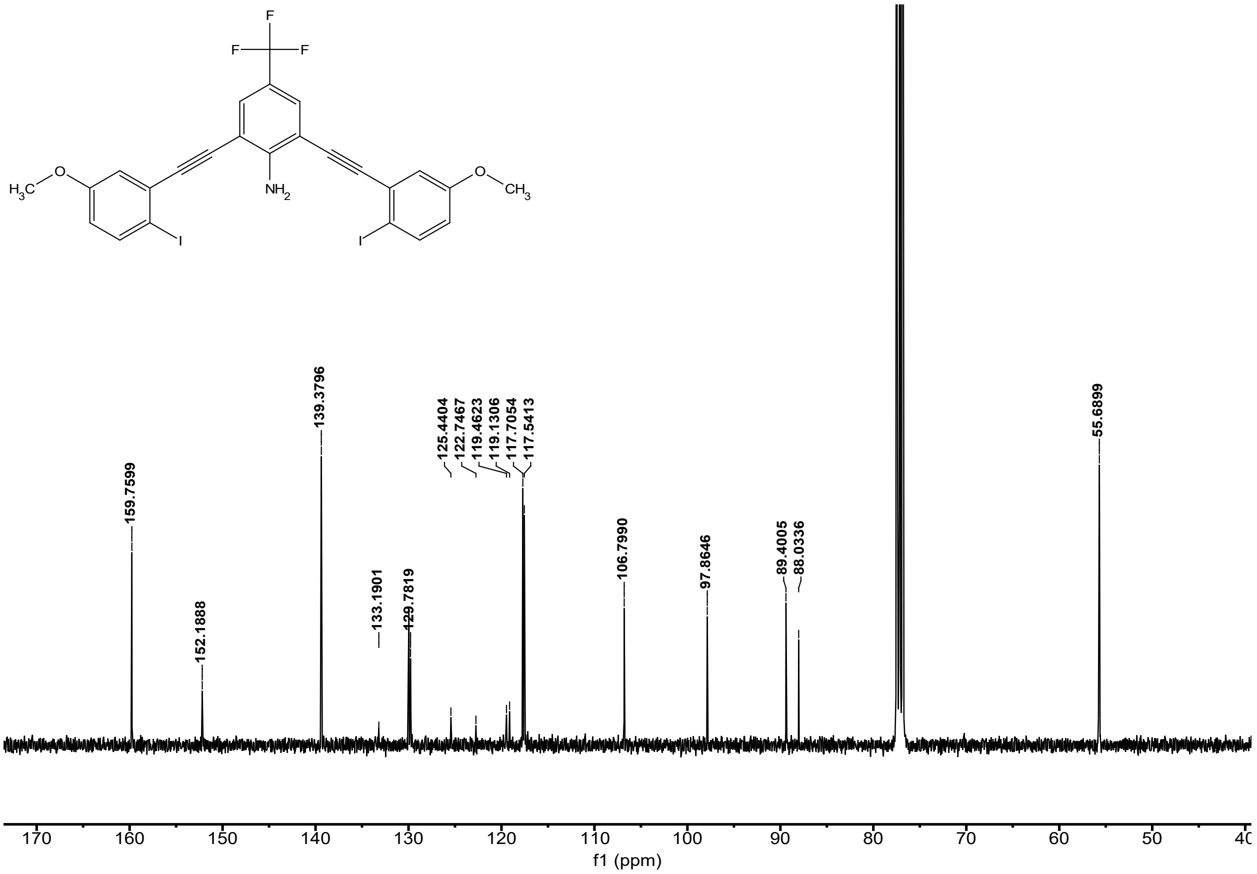

Figure S44. ^{13}C NMR of **2MeO-G3XB** (100 MHz, $CDCl_3$).

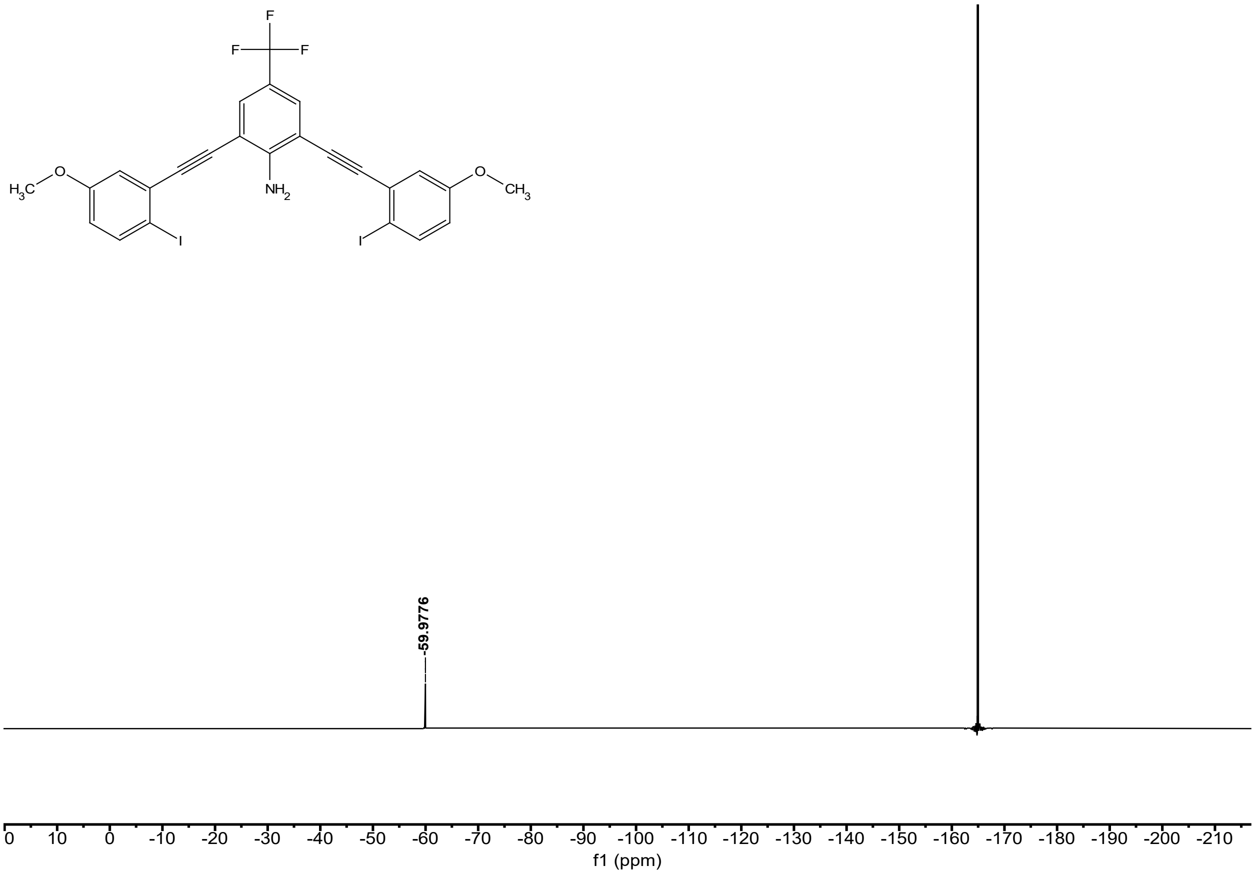

Figure S45. ^{19}F NMR of **2MeO-G3XB** (376 MHz, $CDCl_3$). Hexafluorobenzene (C_6F_6) internal reference.

2,6-bis(2-ethynyl-4-(trifluoromethyl)bromophenyl)-4-chloroaniline (Cl-G3XBBr). To an oven dried Schlenk flask was charged with 2,6-bisethynyl-4-chloroaniline **(2b)** (0.43g 2.45mmol), then vacuumed and backfilled with dry N_2 gas (3x). Bis(triphenylphosphine)palladium (II) dichloride (0.086 g, 0. 125 mmol) was added, vacuumed and backfilled with dry N_2 gas (3x). Copper (I) iodide (0.047 g, 0.245 mmol) was added, vacuumed and backfilled with dry N_2 (3x). The dry reagents were dissolved in 100 mL dry DMF. 1-Bromo-2-iodo-4-(trifluoromethyl)benzene (0.98 mL, 6.1 mmol) and N,N-diisopropylethylamine (2.11 mL, 12.25 mmol) were added to the DMF solution. The flask was carefully vacuumed and backfilled with dry N_2 (3x). The dark brown solution stirred overnight at room temperature. The reaction mixture was extracted with ethyl acetate then washed with water to remove any excess salts and catalysts, then was dried over magnesium sulfate and gravity filtered. Subsequent removal of DMF, hexanes and ethyl acetate by rotary evaporation left and brown solid that was purified by column chromatography (gradient from 100% Hexanes to 10% EtOAc/90% Hexanes) to afford **Cl-G3XBBr** (1.18 g, 78%) as a dull yellow solid.

^{1}H NMR (400 MHz, $CDCl_3$): δ (ppm) = 7.82 (s, 2H), 7.78 (d, J = 8.2 Hz, 2H), 7.46 (d, J = 8.5 Hz, 2H), 7.41 (s, 2H), 5.32 (s, 2H).

^{13}C NMR (100 MHz, CDCl$_3$): δ (ppm) = 148.91, 133.24, 132.87, 130.50 (q, J = 33.2 Hz), 129.93 (q, J = 3.7 Hz), 129.00, 126.22 (q, J = 3.7 Hz), 125.95, 123.95 (q, J = 270.0 Hz), 121.50, 107.82, 93.55, 90.75.

^{19}F NMR (376 MHz, CDCl$_3$): δ (ppm) = –61.31.

HRMS (ESI neg) m/z for $C_{24}H_9F_6Br_2ClN^-$ [M-H$^+$]$^-$: calculated: 619.8680; found: 619.8680.

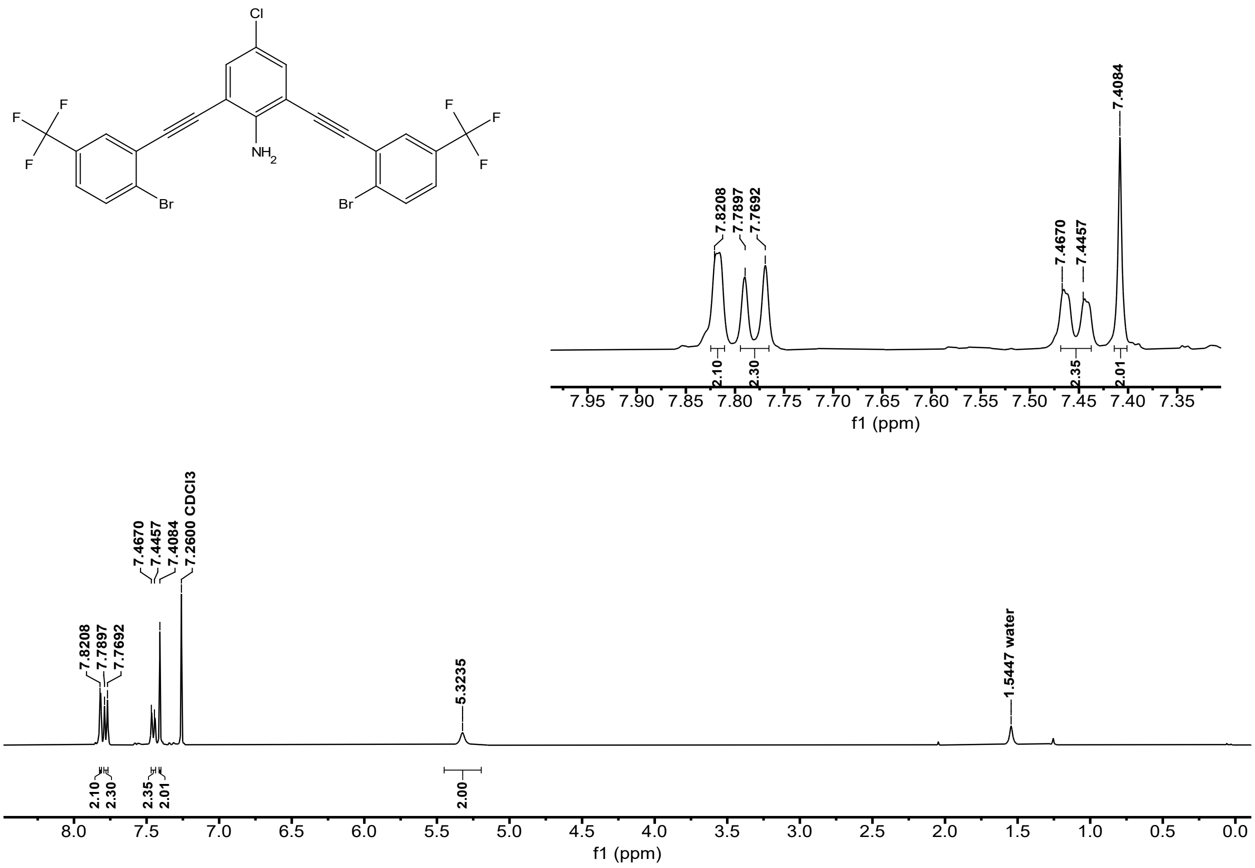

Figure S46. ^{1}H NMR of **Cl-G3XBBr** (400 MHz, CDCl$_3$).

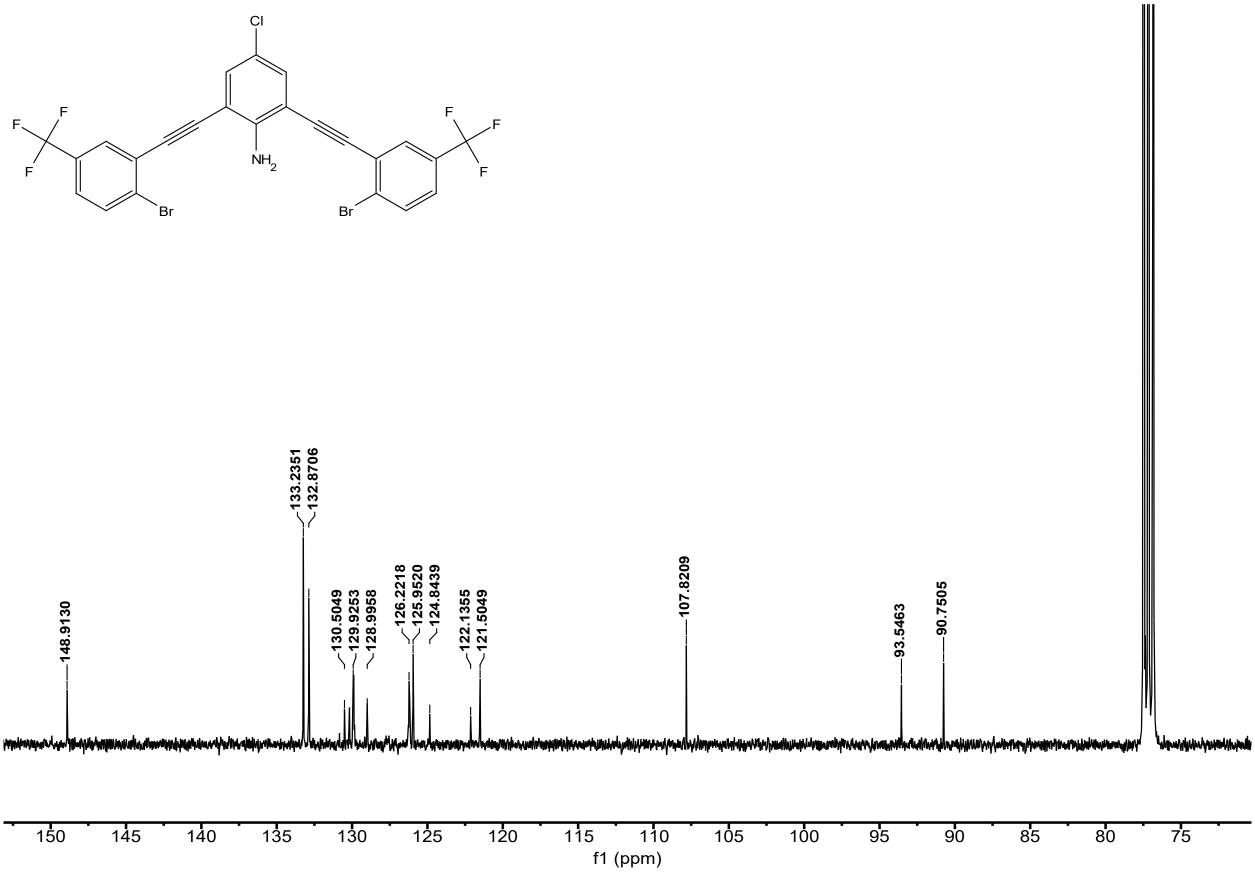

Figure S47. ^{13}C NMR of **Cl-G3XBBr** (100 MHz, $CDCl_3$).

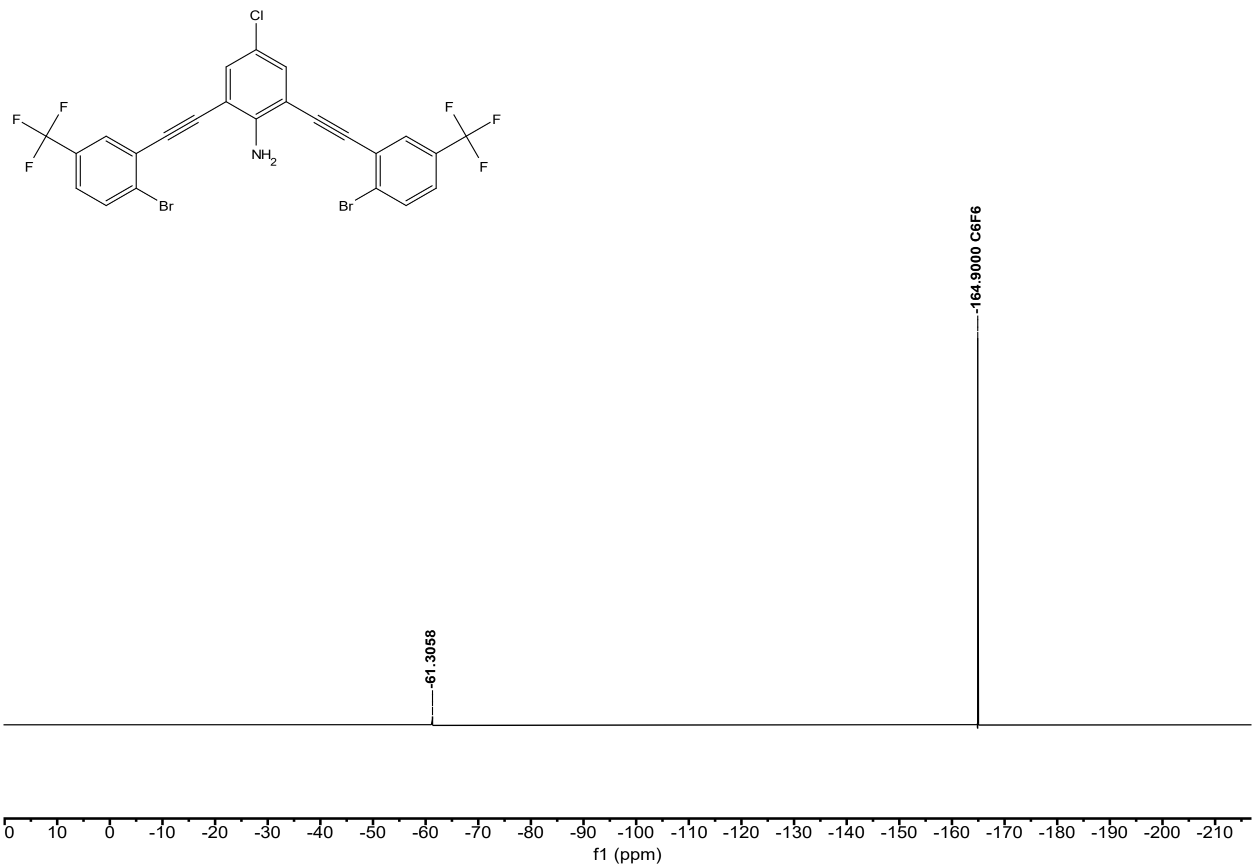

Figure S48. ^{19}F NMR of **Cl-G3XBBr** (376 MHz, $CDCl_3$). Hexafluorobenzene (C_6F_6) internal reference.

2,6-bis(2-ethynyl-4-(trifluoromethyl)iodophenyl)-4-chloroaniline (Cl-G3XB). Cl-G3XBBr (0.200 g, 0.323 mmol), copper iodide (0.006 g, 0.030 mmol), sodium iodide (0.193 g, 1.29 mmol) were added to a 10-20 mL microwave reaction vial containing a stir bar and dissolved in 13 mL 1,4-dioxane. To the vibrant yellow reaction mixture, trans-N,N'-dimethylcyclohexane-1,2-diamine (0.15 mL) was added. The microwave vial was sealed and placed in a Biotage Initiator+ microwave reactor for 12.5 hours at 150 ˚C. After cooling, the reaction was run through pipet silica plug with EtOAc to remove catalysts and salts. The EtOAc crude was then ran through GCMS in order to obtain % conversion of bromines to iodines. If the conversion from bromine to iodine was 99% or greater, then the reaction was concentrated. The beige/yellow product (0.076 g, 33%) was obtained after purified via high performance liquid chromatography (reverse phase column gradient from 80% water/20% acetonitrile to 100% acetonitrile).

^{1}H NMR (400 MHz, $CDCl_3$): δ (ppm) = 8.03 (d, J = 8.2 Hz, 2H), 7.77 (s, 2H), 7.42 (s, 2H), 7.28 (d, J = 9.2 Hz, 2H), 5.39 (s, 2H).

^{13}C NMR (100 MHz, $CDCl_3$): δ (ppm) = 148.96, 139.53, 132.97, 131.29 (q, J = 42.7 Hz), 130.46, 129.17 (q, J = 5.9 Hz), 126.01 (q, J = 3.5 Hz), 123.62 (q, J = 270.8 Hz), 121.53, 107.84, 104.83, 96.84, 89.91.

^{19}F NMR (376 MHz, CDCl$_3$): δ (ppm) = –61.52.

HRMS (ESI neg) m/z for $C_{24}H_9F_6I_2ClN^-$ [M-H$^+$]$^-$: calculated: 713.8423; found: 713.8422.

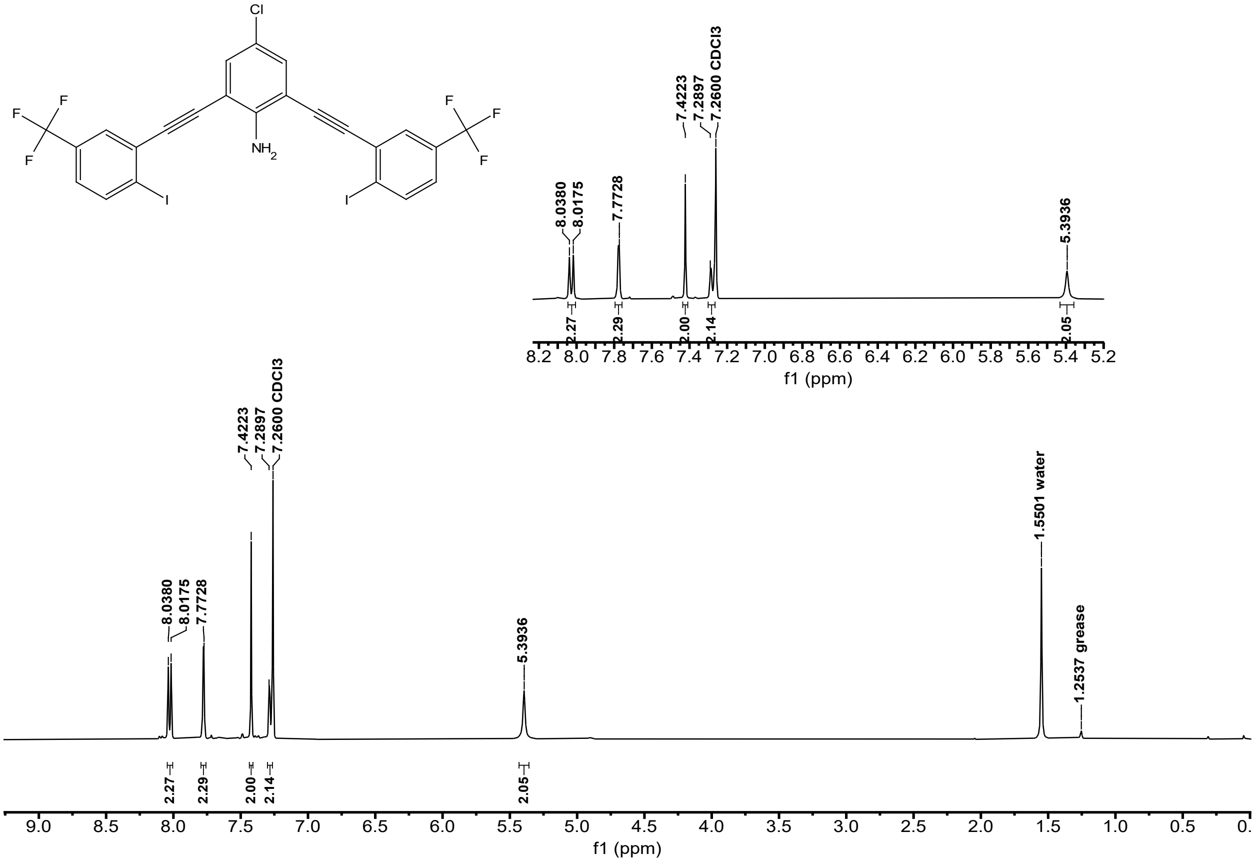

Figure S49. ^{1}H NMR of **Cl-G3XB** (400 MHz, CDCl$_3$).

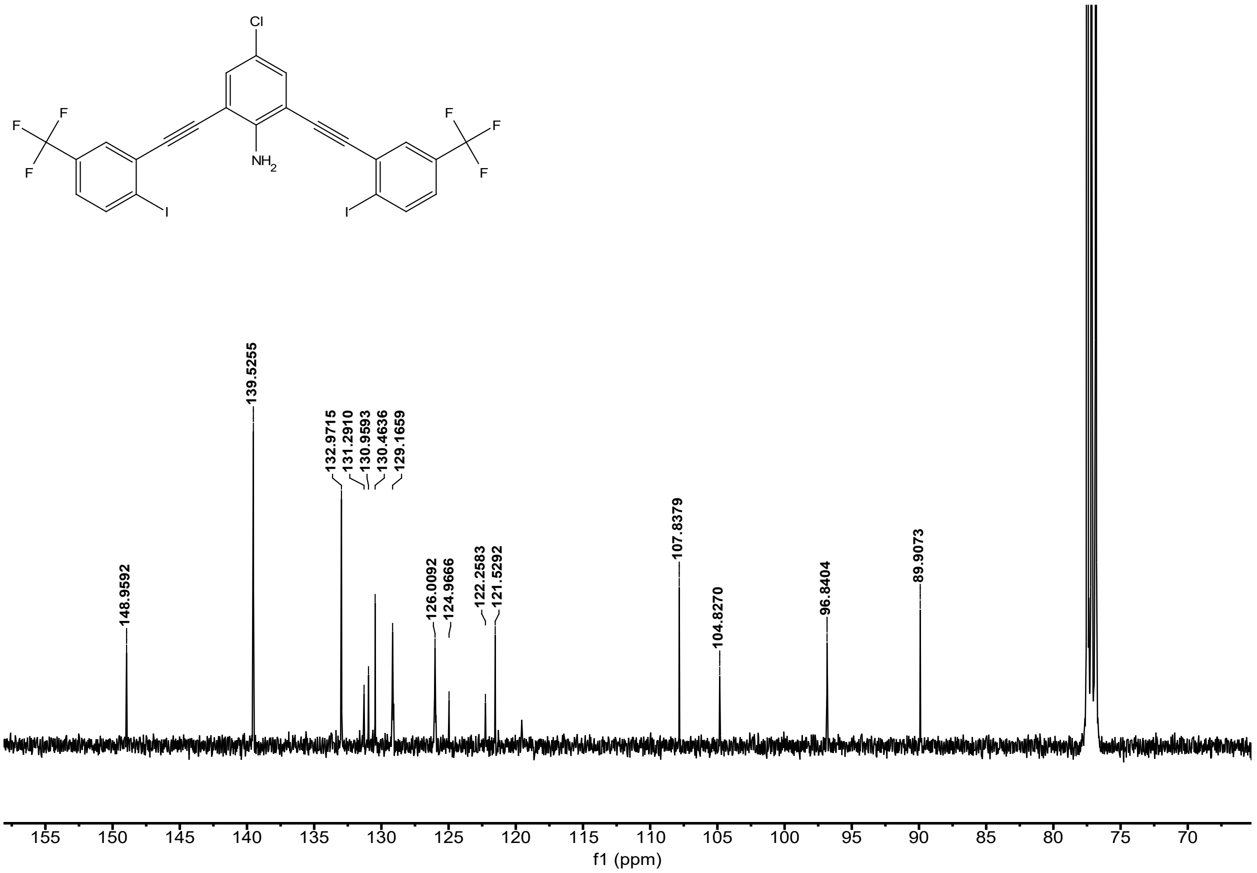

Figure S50. ^{13}C NMR of **Cl-G3XB** (100 MHz, $CDCl_3$).

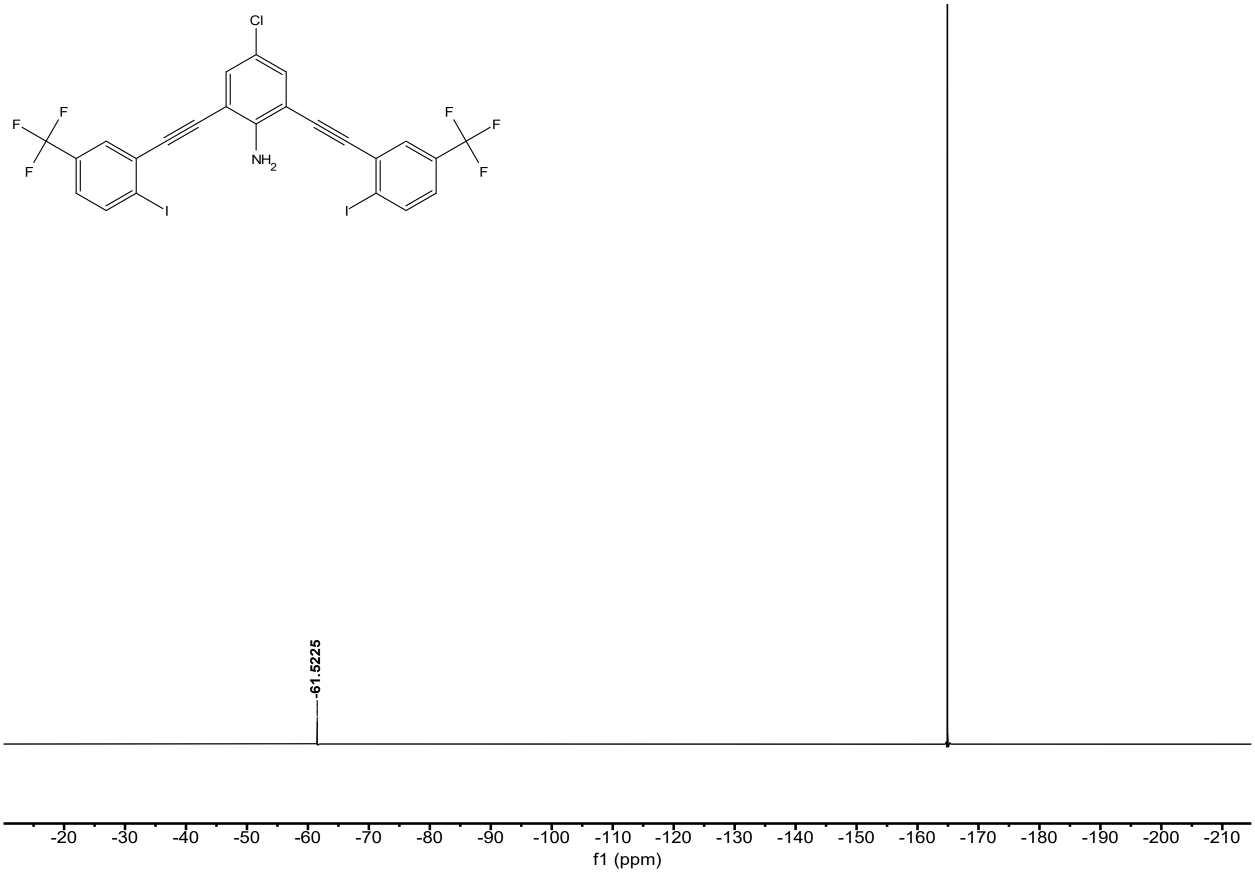

Figure S51. ^{19}F NMR of **Cl-G3XB** (376 MHz, $CDCl_3$). Hexafluorobenzene (C_6F_6) internal reference.

2,6-bis(2-ethynyl-4-(trifluoromethyl)bromophenyl)-4-fluoroaniline (F-G3XBBr). To an oven dried Schlenk flask was charged with 2,6-bisethynyl-4-fluoroaniline **(2c)** (0.159g 1mmol), then vacuumed and backfilled with dry N_2 gas (3x). Bis(triphenylphosphine)palladium (II) dichloride (0.035 g, 0. 05 mmol) was added, vacuumed and backfilled with dry N_2 gas (3x). Copper (I) iodide (0.019 g, 0.1 mmol) was added, vacuumed and backfilled with dry N_2 (3x). The dry reagents were dissolved in 60 mL dry DMF. 1-Bromo-2-iodo-4-(trifluoromethyl)benzene (0.4 mL, 2.5 mmol) and N,N-diisopropylethylamine (0.86 mL, 5 mmol) were added to the DMF solution. The flask was carefully vacuumed and backfilled with dry N_2 (3x). The dark brown solution stirred overnight at room temperature. The reaction mixture was extracted with ethyl acetate then washed with water to remove any excess salts and catalysts, then was dried over magnesium sulfate and gravity filtered. Subsequent removal of DMF, hexanes and ethyl acetate by rotary evaporation left and brown solid that was purified by column chromatography (gradient from 100% Hexanes to 3% EtOAc/97% Hexanes) to afford **F-G3XBBr** (0.28 g, 53%) as a dull yellow solid.

^{1}H NMR (400 MHz, $CDCl_3$): δ (ppm) = 7.82 (s, 2H), 7.78 (d, J = 8.4 Hz, 2H), 7.45 (d, J = 8.4 Hz, 2H), 7.18 (d, J = 8.4 Hz, 2H), 5.16 (s, 2H).

^{13}C NMR (100 MHz, $CDCl_3$): δ (ppm) = 153.95 (d, J = 235.2 Hz), 147.23, 133.23, 130.48 (q, J = 33.2 Hz), 129.95 (q, J = 4.0 Hz), 129.00, 126.21 (q, J = 3.6 Hz), 125.96, 123.50 (q, J = 272.1 Hz), 120.18 (d, J = 24.0 Hz), 107.23 (d, J = 9.8 Hz), 93.33, 91.08 (d, J = 3.4 Hz).

^{19}F NMR (376 MHz, $CDCl_3$): δ (ppm) = –61.31, -125.31.

HRMS (ESI neg) m/z for $C_{24}H_9F_7Br_2N^-$ [M-H$^+$]$^-$: calculated: 603.8975; found: 603.8976.

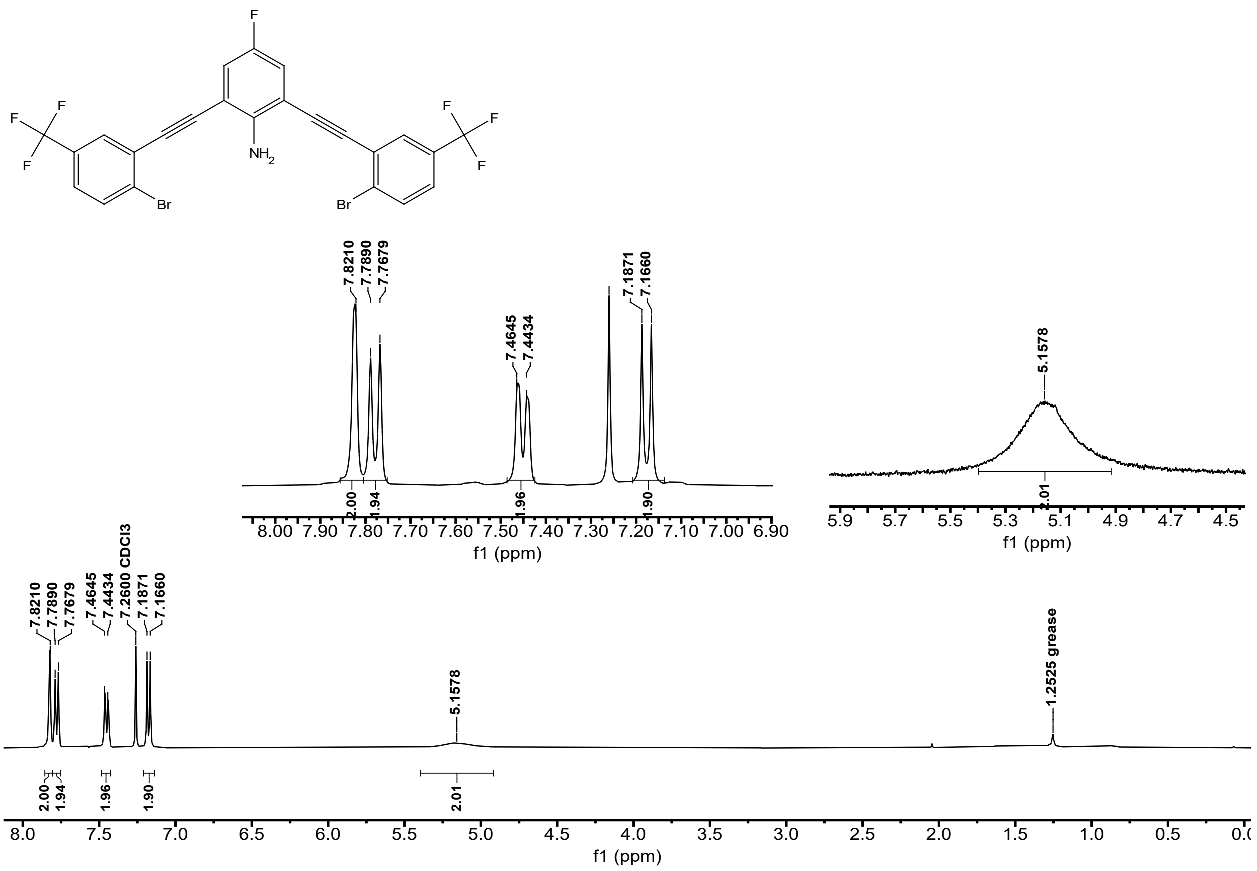

Figure S52. ^{1}H NMR of **F-G3XBBr** (400 MHz, $CDCl_3$).

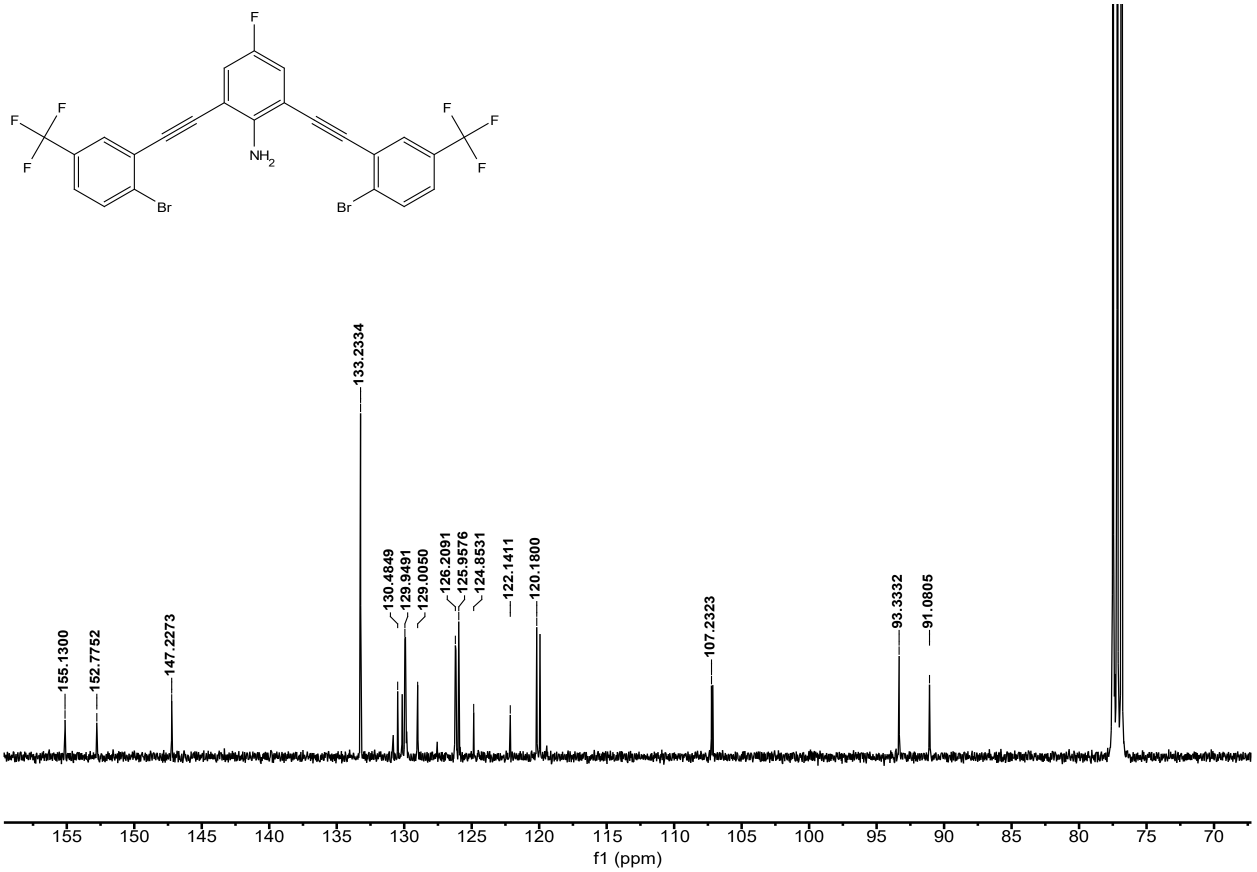

Figure S53. ^{13}C NMR of **F-G3XBBr** (100 MHz, $CDCl_3$).

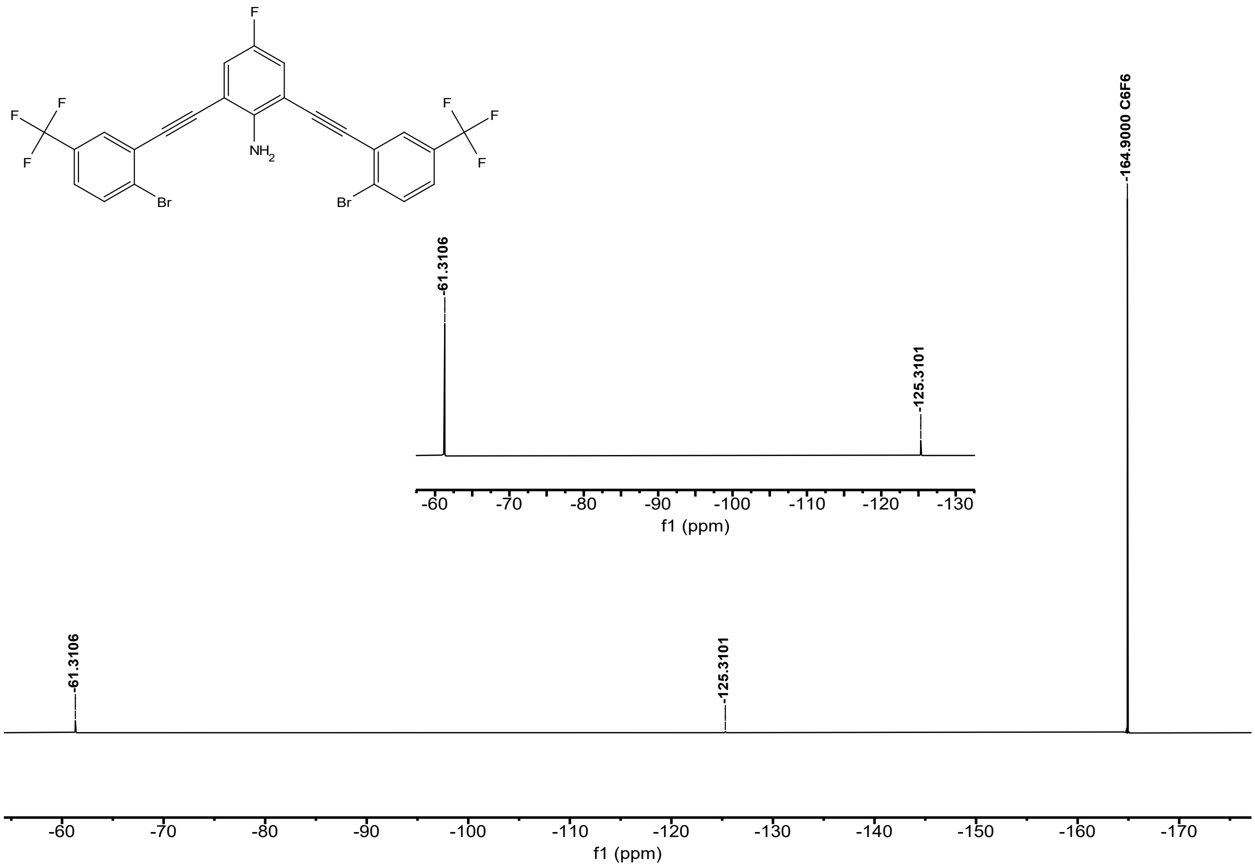

Figure S54. ^{19}F NMR of **F-G3XBBr** (376 MHz, $CDCl_3$). Hexafluorobenzene (C_6F_6) internal reference.

2,6-bis(2-ethynyl-4-(trifluoromethyl)iodophenyl)-4-fluoroaniline (F-G3XB). F-G3XBBr (0.100 g, 0.165 mmol), copper iodide (0.003 g, 0.016 mmol), sodium iodide (0.102 g, 0.68 mmol) were added to a 10-20 mL microwave reaction vial containing a stir bar and dissolved in 13 mL 1,4-dioxane. To the vibrant yellow reaction mixture, trans-N,N'-dimethylcyclohexane-1,2-diamine (0.1 mL) was added. The microwave vial was sealed and placed in a Biotage Initiator+ microwave reactor for 13 hours at 150 ˚C. After cooling, the reaction was run through pipet silica plug with EtOAc to remove catalysts and salts. The EtOAc crude was then ran through GCMS in order to obtain % conversion of bromines to iodines. If the conversion from bromine to iodine was 99% or greater, then the reaction was concentrated. The beige/yellow product (0.099 g, 86%) was obtained after purified via high performance liquid chromatography (reverse phase column gradient from 80% water/20% acetonitrile to 100% acetonitrile).

^{1}H NMR (400 MHz, $CDCl_3$): δ (ppm) = 8.03 (d, J = 8.2 Hz, 2H), 7.79 (s, 2H), 7.28 (d, J = 8.0 Hz, 2H), 7.20 (d, J = 6.4 Hz, 2H), 5.25 (s, 2H).

^{13}C NMR (100 MHz, $CDCl_3$): δ (ppm) = 153.99 (d, J = 235.1 Hz), 147.26, 139.54, 131.28 (q, J = 33.3 Hz), 130.47, 129.20 (q, J = 3.8 Hz), 126.01 (q, J = 3.7 Hz), 123.62 (q, J = 270.0 Hz), 120.29 (d, J = 23.3 Hz), 107.27 (d, J = 11.9 Hz), 104.83, 96.62, 90.23 (d, J = 3.3 Hz).

^{19}F NMR (376 MHz, CDCl$_3$): δ (ppm) = –61.51, -125.30.

HRMS (ESI neg) m/z for $C_{24}H_9F_7I_2N^-$ [M-H$^+$]$^-$: calculated: 697.8718; found: 697.8718.

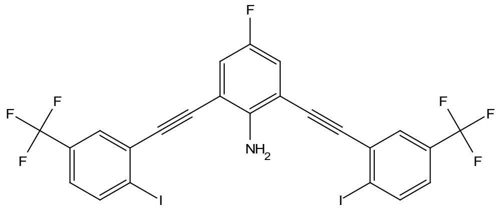

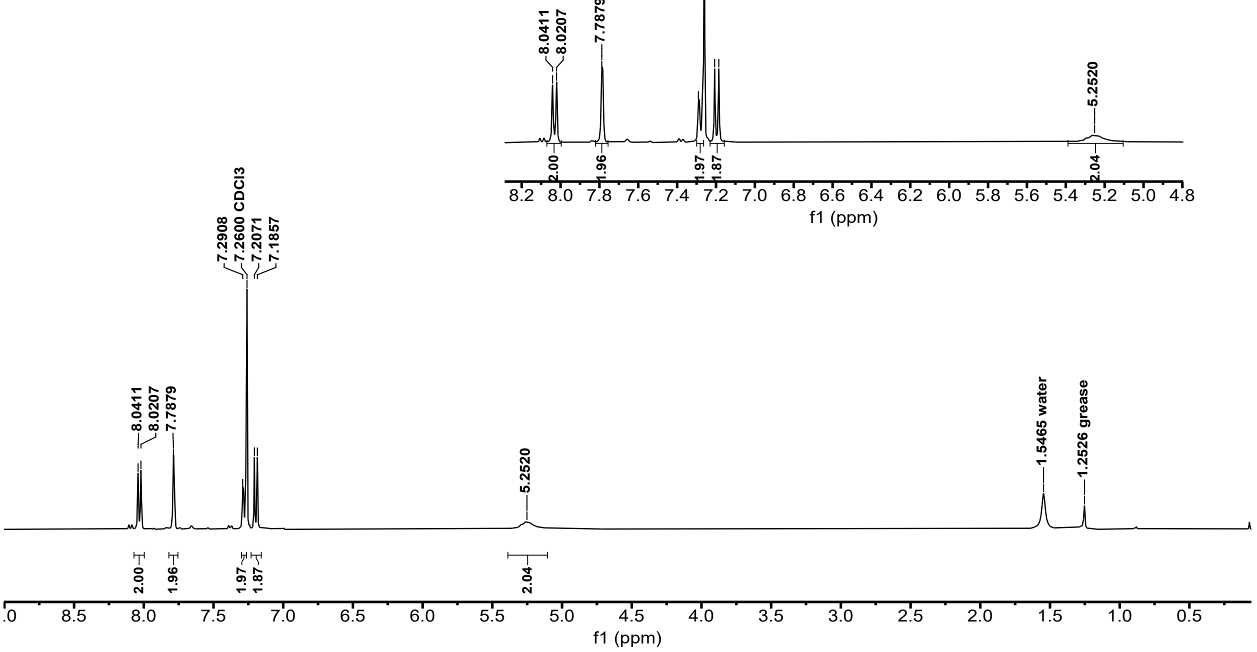

Figure S55. ^{1}H NMR of **F-G3XB** (400 MHz, CDCl$_3$).

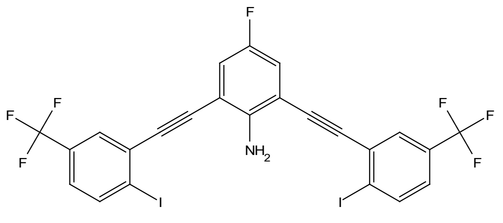

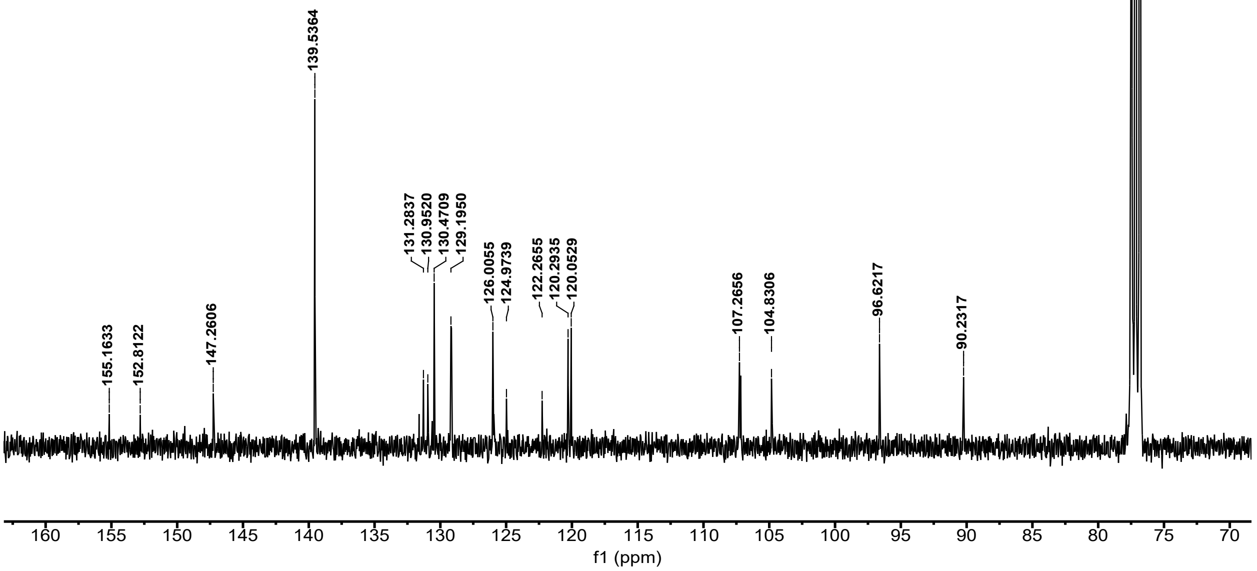

Figure S56. ^{13}C NMR of **F-G3XB** (100 MHz, $CDCl_3$).

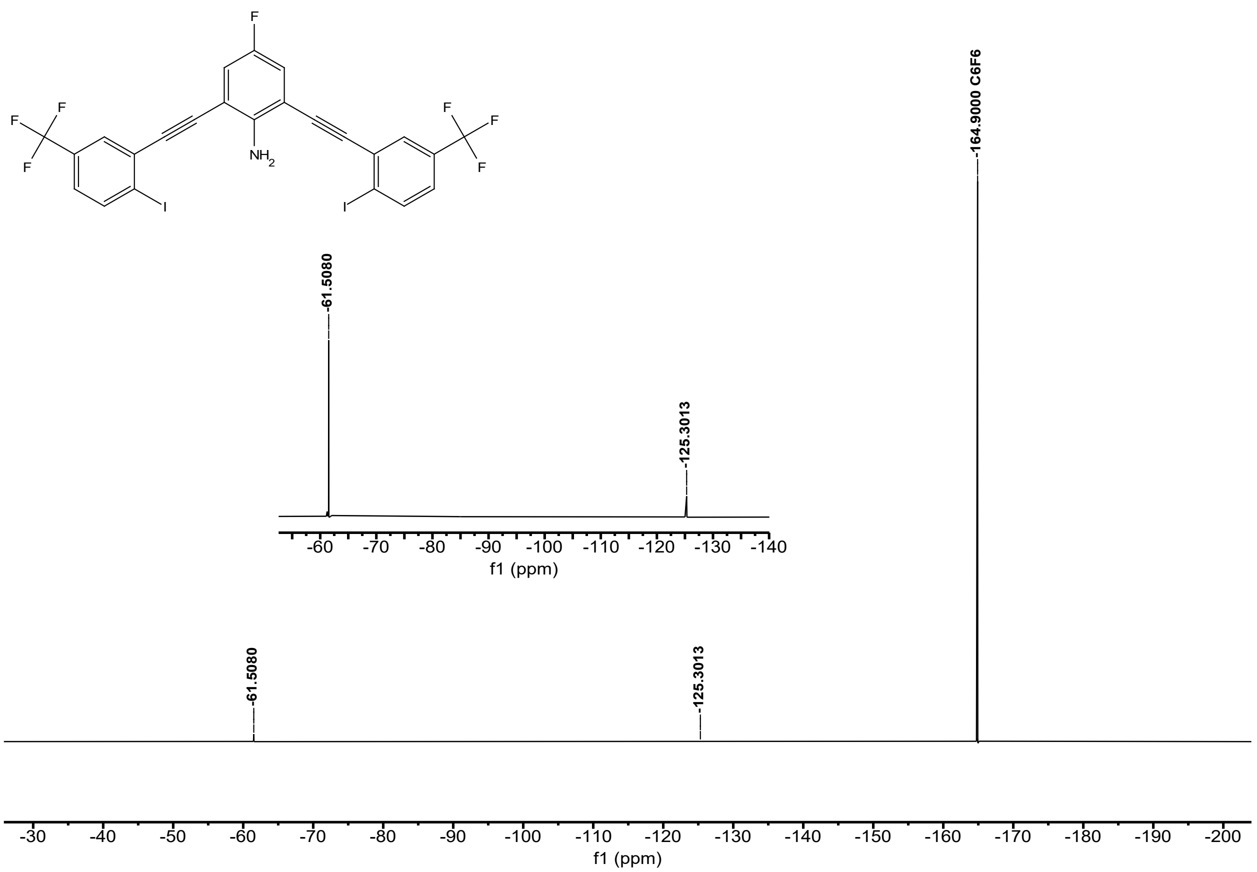

Figure S57. ^{19}F NMR of **F-G3XB** (376 MHz, $CDCl_3$). Hexafluorobenzene (C_6F_6) internal reference.

2,6-bis(2-ethynyl-4-(trifluoromethyl)bromophenyl)-aniline (H-G3XBBr). To an oven dried Schlenk flask was charged with 2,6-bisethynyl-aniline **(2d)** (0.305g 2.16 mmol), then vacuumed and backfilled with dry N_2 gas (3x). Bis(triphenylphosphine)palladium (II) dichloride (0.077 g, 0. 11 mmol) was added, vacuumed and backfilled with dry N_2 gas (3x). Copper (I) iodide (0.042 g, 0.22 mmol) was added, vacuumed and backfilled with dry N_2 (3x). The dry reagents were dissolved in 60 mL dry DMF. 1-Bromo-2-iodo-4-(trifluoromethyl)benzene (0.86 mL, 5.4 mmol) and N,N-diisopropylethylamine (1.86 mL, 10.8 mmol) were added to the DMF solution. The flask was carefully vacuumed and backfilled with dry N_2 (3x). The dark brown solution stirred for 48 hours at room temperature. The reaction mixture was extracted with ethyl acetate then washed with water to remove any excess salts and catalysts, then was dried over magnesium sulfate and gravity filtered. Subsequent removal of DMF, hexanes and ethyl acetate by rotary evaporation left and brown solid that was purified by column chromatography (gradient from 100% Hexanes to 3% EtOAc/97% Hexanes) to afford **H-G3XBBr** (0.773 g, 61%) as a dull yellow solid.

^{1}H NMR (400 MHz, $CDCl_3$): δ (ppm) = 7.83 (s, 2H), 7.78 (d, J = 8.3 Hz, 2H), 7.44 (d, J = 7.6 Hz, 2H), 7.44 (d, J = 7.2 Hz, 2H), 7.73 (t, J = 7.7 Hz, 1H), 5.30 (s, 2H).

^{13}C NMR (100 MHz, CDCl$_3$): δ (ppm) = 150.34, 134.68, 133.17, 130.43 (q, J = 33.2 Hz), 129.82 (q, J = 4.0 Hz), 128.91, 126.42, 125.84 (q, J = 3.6 Hz), 123.57 (q, J = 270.8 Hz), 117.41, 106.54, 92.78, 92.14.

^{19}F NMR (376 MHz, CDCl$_3$): δ (ppm) = –61.29.

HRMS (ESI neg) m/z for $C_{24}H_{10}F_6Br_2N^-$ [M-H$^+$]$^-$: calculated: 585.9069; found: 585.9069.

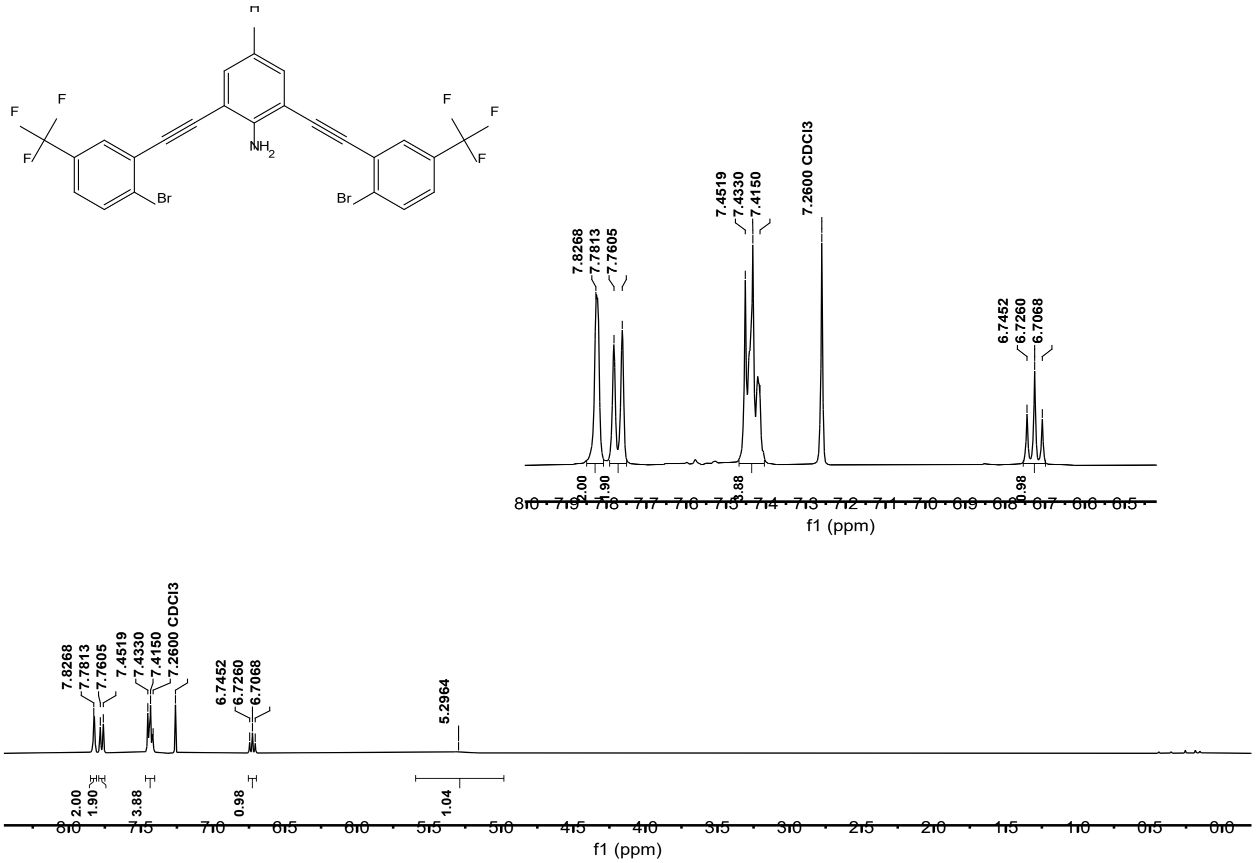

Figure S58. ^{1}H NMR of **H-G3XBBr** (400 MHz, CDCl$_3$).

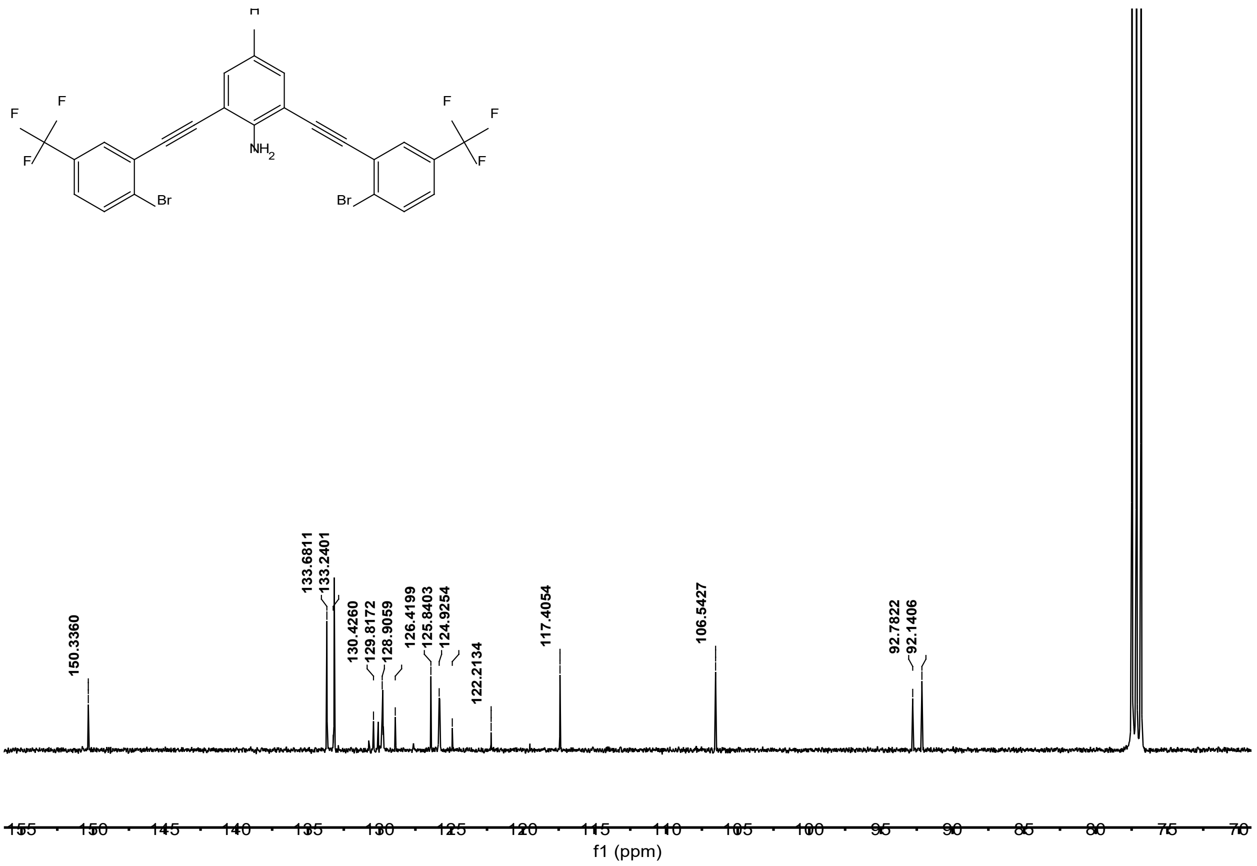

Figure S59. ^{13}C NMR of **H-G3XBBr** (100 MHz, $CDCl_3$).

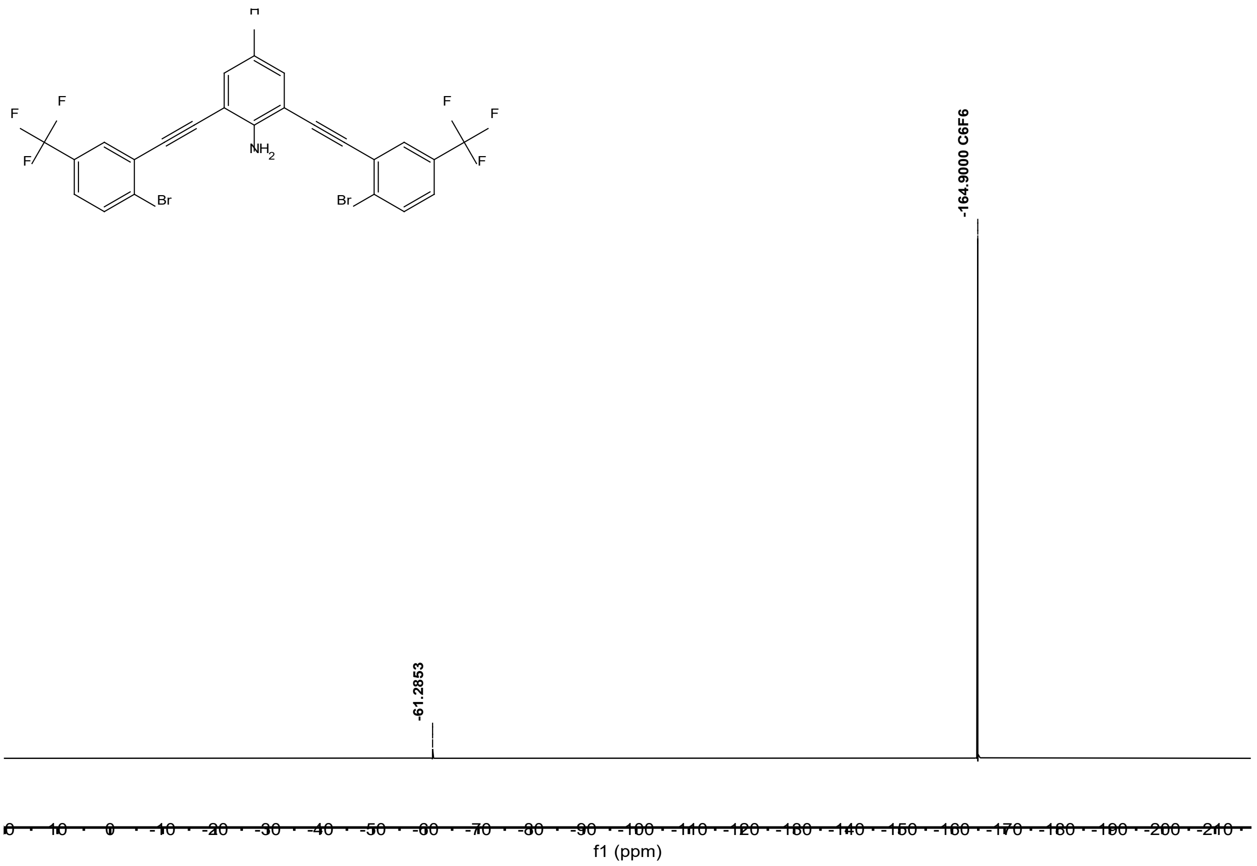

Figure S60. ^{19}F NMR of **H-G3XBBr** (376 MHz, $CDCl_3$). Hexafluorobenzene (C_6F_6) internal reference.

2,6-bis(2-ethynyl-4-(trifluoromethyl)iodophenyl)-aniline (H-G3XB). H-G3XBBr (0.200 g, 0.341 mmol), copper iodide (0.006 g, 0.030 mmol), sodium iodide (0.183 g, 1.22 mmol) were added to a 10-20 mL microwave reaction vial containing a stir bar and dissolved in 13 mL 1,4-dioxane. To the vibrant yellow reaction mixture, trans-N,N'-dimethylcyclohexane-1,2-diamine (0.15 mL) was added. The microwave vial was sealed and placed in a Biotage Initiator+ microwave reactor for 16 hours at 150 ˚C. After cooling, the reaction was run through pipet silica plug with EtOAc to remove catalysts and salts. The EtOAc crude was then ran through GCMS in order to obtain % conversion of bromines to iodines. If the conversion from bromine to iodine was 99% or greater, then the reaction was concentrated. The beige/yellow product (0.103 g, 44%) was obtained after purified via high performance liquid chromatography (reverse phase column gradient from 80% water/20% acetonitrile to 100% acetonitrile).

^{1}H NMR (400 MHz, $CDCl_3$): δ (ppm) = 8.02 (d, J = 8.3 Hz, 2H), 7.78 (s, 2H), 7.46 (d, J = 7.7 Hz, 2H), 7.25 (d, J = 7.4 Hz, 2H), 6.73 (t, J = 7.7 Hz, 1H), 5.35 (s, 2H).

^{13}C NMR (100 MHz, $CDCl_3$): δ (ppm) = 150.37, 139.46, 133.80, 131.04 (q, J = 33.0 Hz), 130.02, 128.99 (q, J = 3.7 Hz), 125.65 (q, J = 3.7 Hz), 123.69 (q, J = 270.8 Hz), 117.44, 106.53, 104.79, 96.09, 91.30.

^{19}F NMR (376 MHz, CDCl$_3$): δ (ppm) = –61.50.

HRMS (ESI neg) m/z for $C_{24}H_{10}F_6I_2N^-$ [M-H$^+$]$^-$: calculated: 679.8812; found: 679.8812.

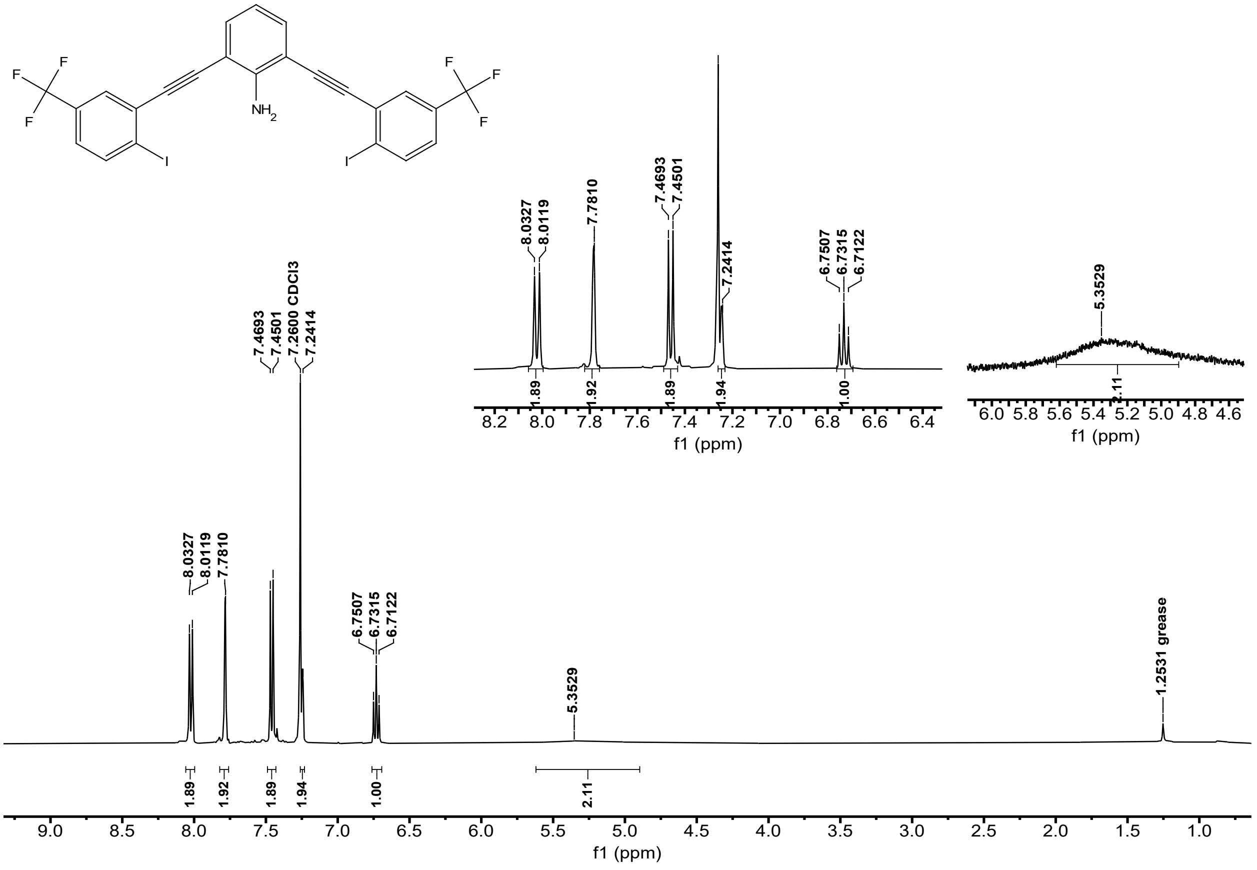

Figure S61. ^{1}H NMR of **H-G3XB** (400 MHz, CDCl$_3$).

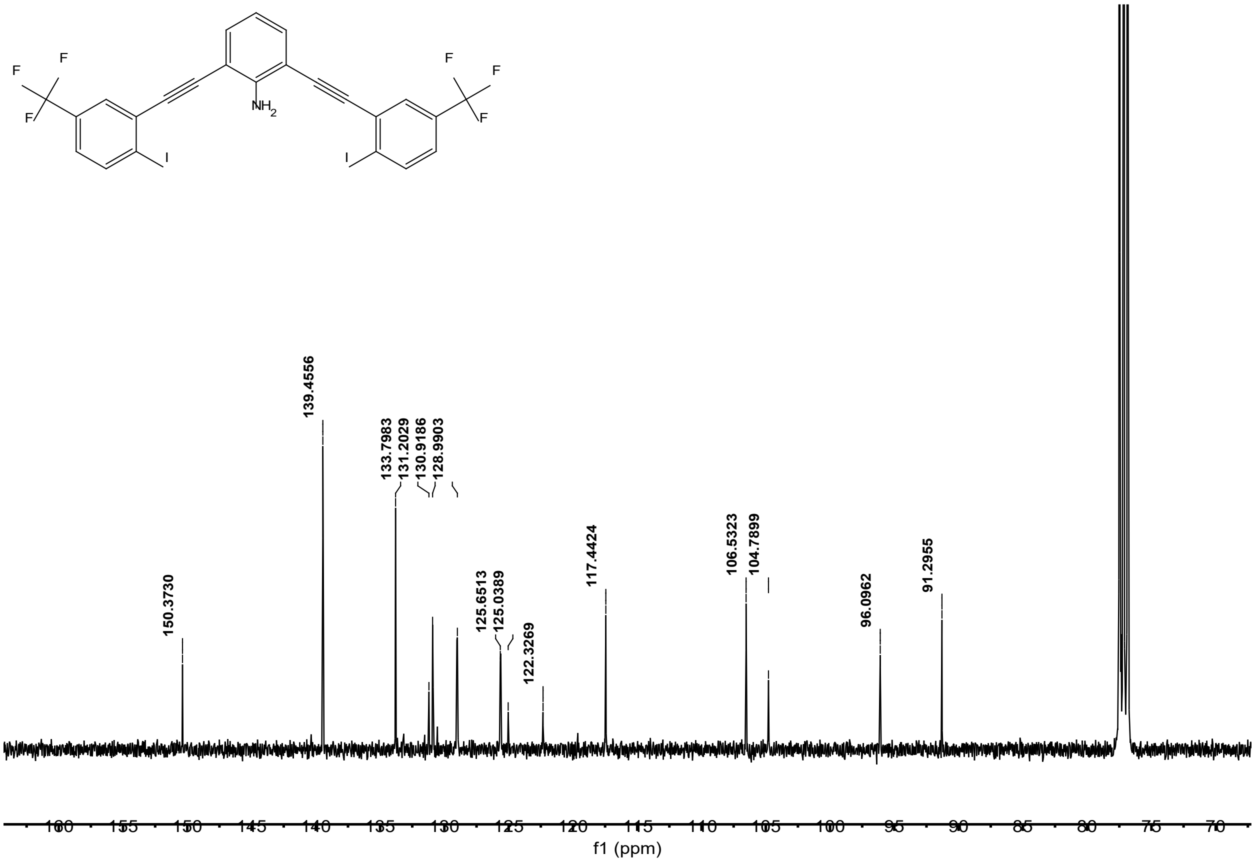

Figure S62. ^{13}C NMR of **H-G3XB** (100 MHz, $CDCl_3$).

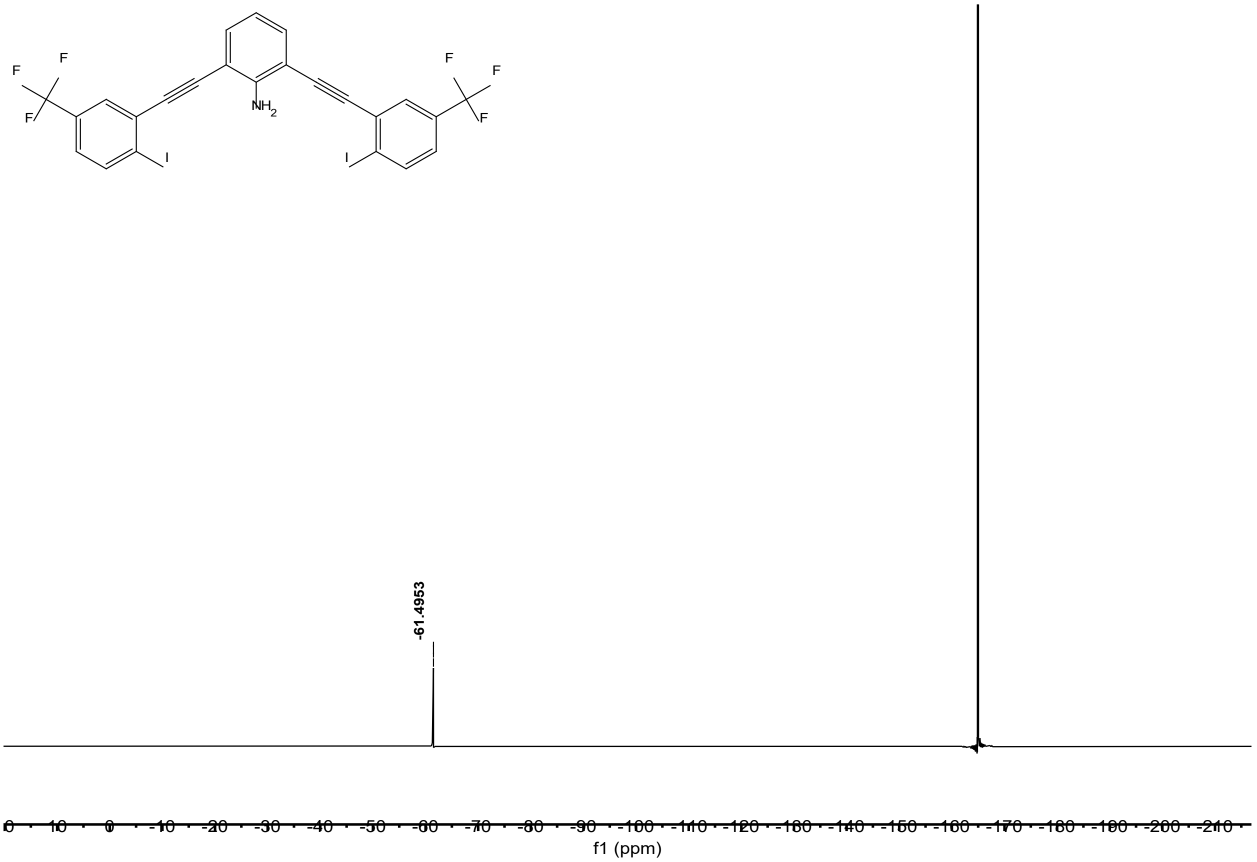

Figure S63. ^{19}F NMR of **H-G3XB** (376 MHz, $CDCl_3$). Hexafluorobenzene (C_6F_6) internal reference.

2,6-bis(2-ethynyl-4-(trifluoromethyl)bromophenyl)-4-methylaniline (Me-G3XBBr). To an oven dried Schlenk flask was charged with 2,6-bisethynyl-4-methylaniline **(2e)** (0.133 g 0.86 mmol), then vacuumed and backfilled with dry N_2 gas (3x). Bis(triphenylphosphine)palladium (II) dichloride (0.035 g, 0. 05 mmol) was added, vacuumed and backfilled with dry N_2 gas (3x). Copper (I) iodide (0.019 g, 0.1 mmol) was added, vacuumed and backfilled with dry N_2 (3x). The dry reagents were dissolved in 60 mL dry DMF. 1-Bromo-2-iodo-4-(trifluoromethyl)benzene (0.4 mL, 2.5 mmol) and N,N-diisopropylethylamine (0.86 mL, 5 mmol) were added to the DMF solution. The flask was carefully vacuumed and backfilled with dry N_2 (3x). The dark brown solution stirred overnight at room temperature. The reaction mixture was extracted with ethyl acetate then washed with water to remove any excess salts and catalysts, then was dried over magnesium sulfate and gravity filtered. Subsequent removal of DMF, hexanes and ethyl acetate by rotary evaporation left and brown solid that was purified by column chromatography (gradient from 100% Hexanes to 3% EtOAc/97% Hexanes) to afford **Me-G3XBBr** (0.344 g, 67%) as a dull yellow solid.

^{1}H NMR (400 MHz, $CDCl_3$): δ (ppm) = 7.80 (s, 2H), 7.75 (d, J = 8.4 Hz, 2H), 7.41 (d, J = 8.4 Hz, 2H), 7.25 (s, 2H), 5.13 (s, 2H), 2.25 (s, 3H).

^{13}C NMR (100 MHz, CDCl$_3$): δ (ppm) = 148.35, 139.45, 134.27, 131.01 (q, J = 32.8 Hz), 131.00, 128.98 (q, J = 4.0 Hz), 126.72, 125.57 (q, J = 3.7 Hz), 123.70 (q, J = 270.8 Hz), 106.57, 104.79, 95.85, 91.54, 20.24.

^{19}F NMR (376 MHz, CDCl$_3$): δ (ppm) = -61.29.

HRMS (ESI neg) m/z for $C_{25}H_{12}F_6Br_2N^-$ [M-H$^+$]$^-$: calculated: 599.9226; found: 599.9224.

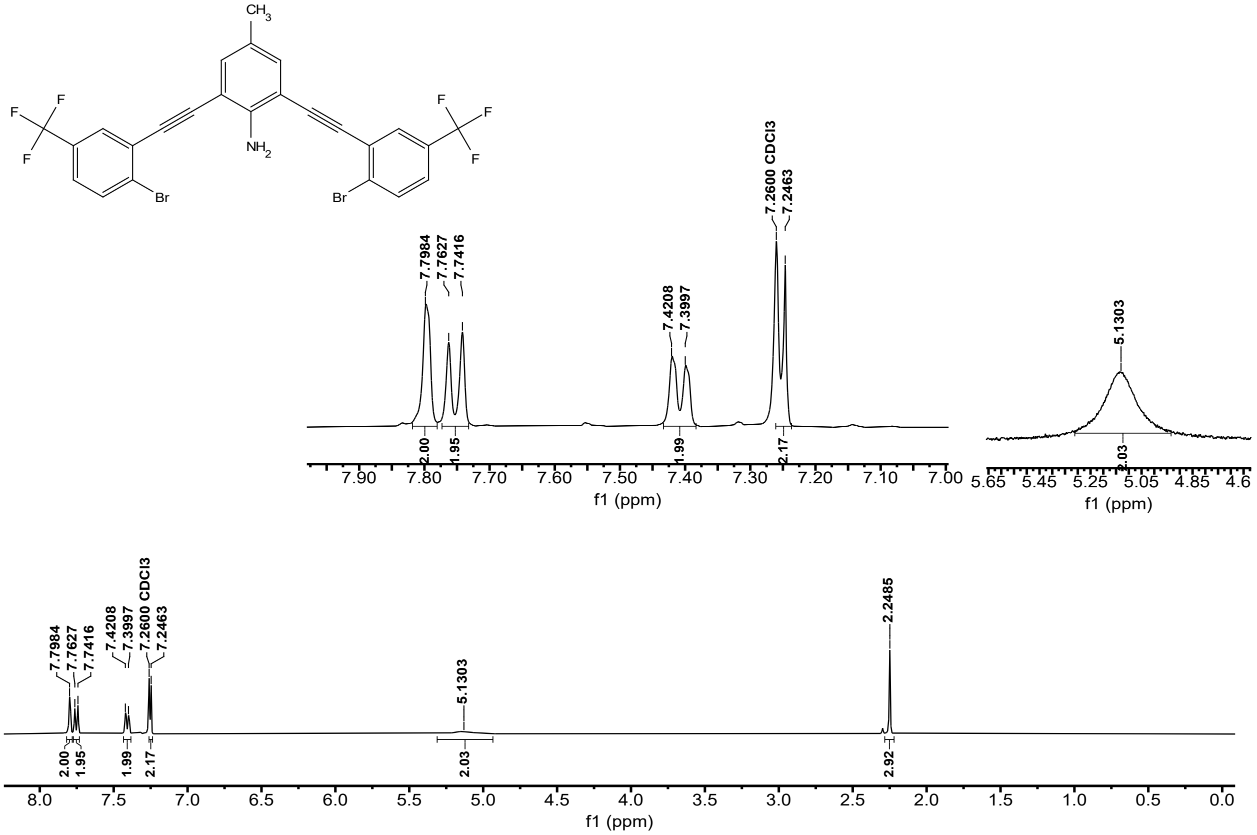

Figure S64. ^{1}H NMR of **Me-G3XBBr** (400 MHz, CDCl$_3$).

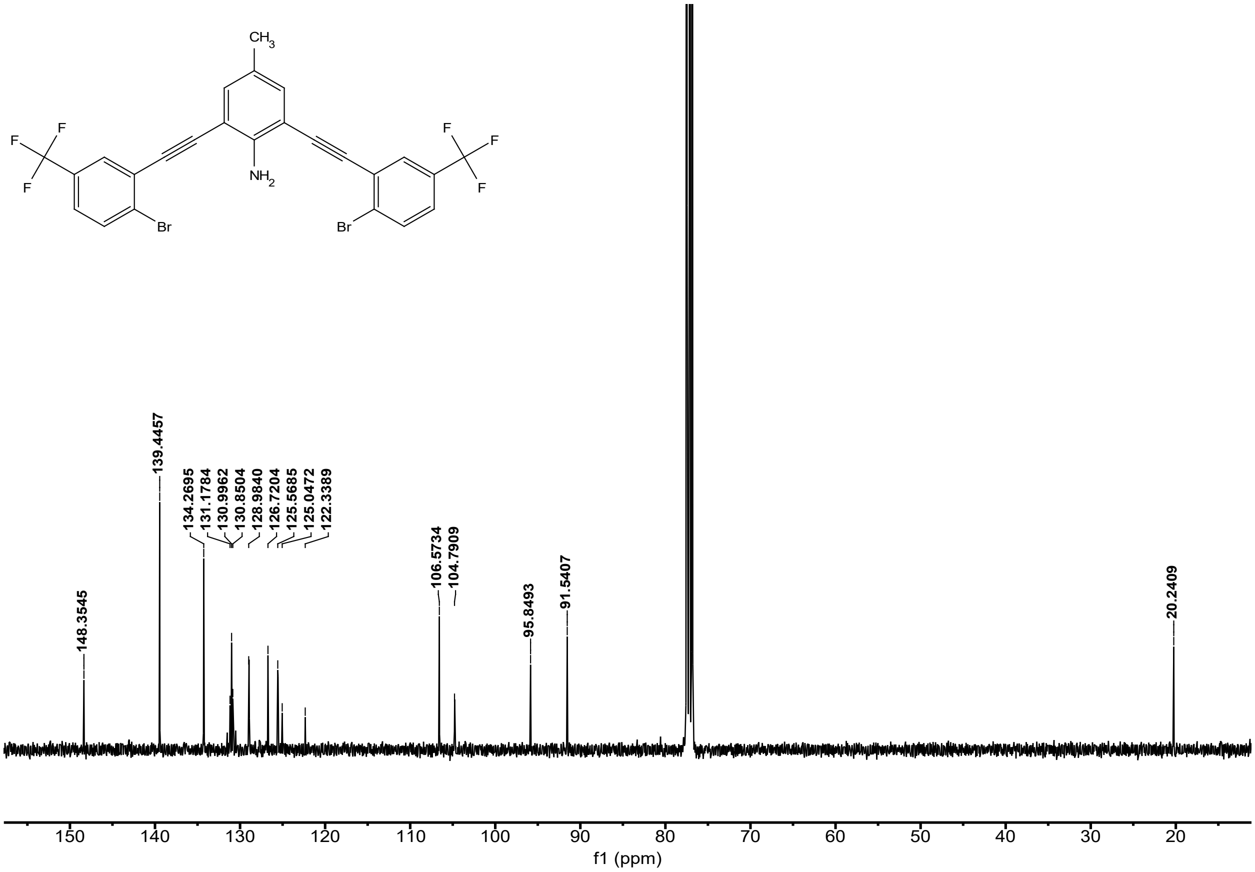

Figure S65. ^{13}C NMR of **Me-G3XBBr** (100 MHz, $CDCl_3$).

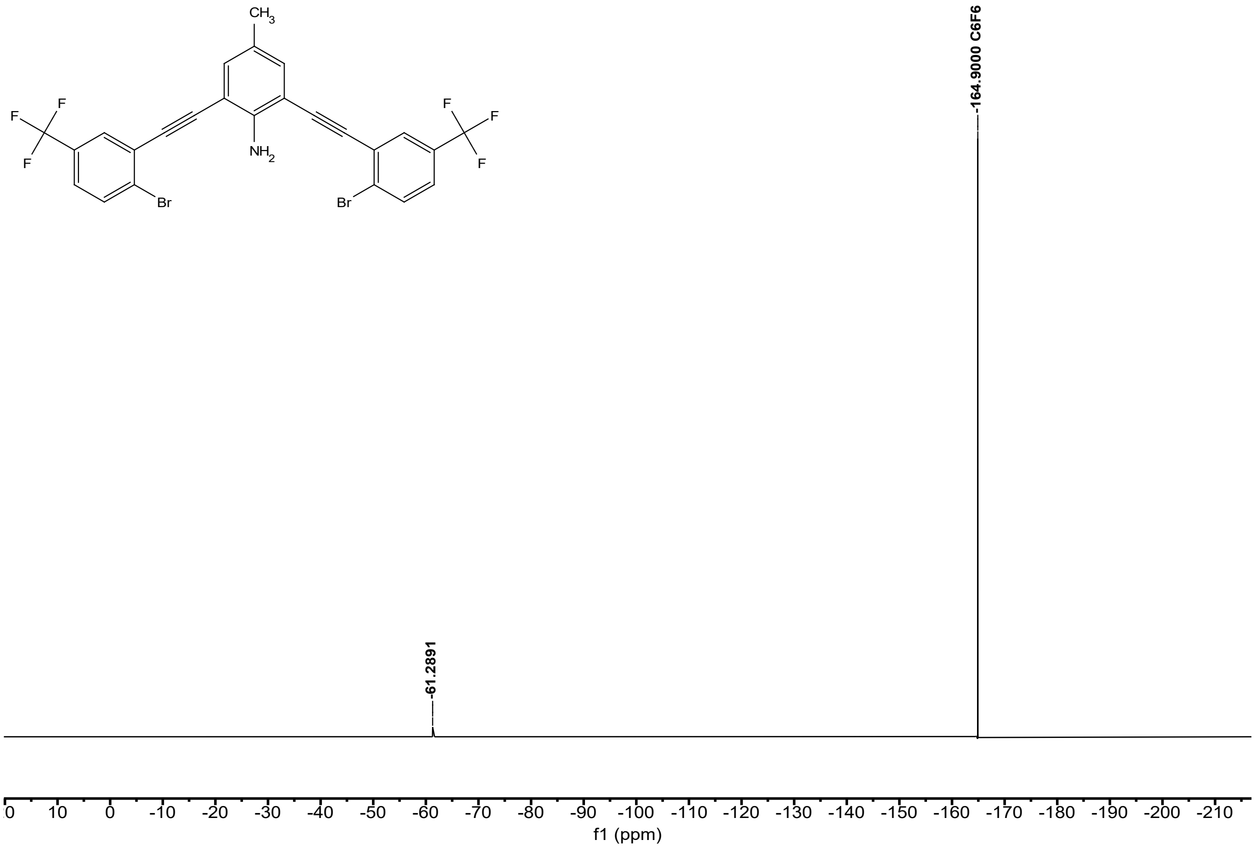

Figure S66. ^{19}F NMR of **Me-G3XBBr** (376 MHz, $CDCl_3$). Hexafluorobenzene (C_6F_6) internal reference.

2,6-bis(2-ethynyl-4-(trifluoromethyl)iodophenyl)-4-methylaniline (Me-G3XB). Me-G3XBBr (0.200 g, 0.332 mmol), copper iodide (0.006 g, 0.033 mmol), sodium iodide (0.200 g, 1.33 mmol) were added to a 10-20 mL microwave reaction vial containing a stir bar and dissolved in 13 mL 1,4-dioxane. To the vibrant yellow reaction mixture, trans-N,N'-dimethylcyclohexane-1,2-diamine (0.15 mL) was added. The microwave vial was sealed and placed in a Biotage Initiator+ microwave reactor for 17 hours at 150 ˚C. After cooling, the reaction was run through pipet silica plug with EtOAc to remove catalysts and salts. The EtOAc crude was then ran through GCMS in order to obtain % conversion of bromines to iodines. If the conversion from bromine to iodine was 99% or greater, then the reaction was concentrated. The beige/yellow product (0.100 g, 43%) was obtained after purified via high performance liquid chromatography (reverse phase column gradient from 80% water/20% acetonitrile to 100% acetonitrile).

^{1}H NMR (400 MHz, $CDCl_3$): δ (ppm) = 8.02 (d, J = 8.2 Hz, 2H), 7.77 (s, 2H), 7.29 (s, 2H), 7.64 (d, J = 6.1 Hz, 2H), 7.25 (d, J = 5.8 Hz, 2H), 5.20 (s, 2H), 2.27 (s, 3H).

^{13}C NMR (100 MHz, $CDCl_3$): δ (ppm) = 148.35, 139.45, 134.27, 131.01 (q, J = 32.8 Hz), 131.00, 128.98 (q, J = 4.0 Hz), 126.72, 125.57 (q, J = 3.7 Hz), 123.70 (q, J = 270.8 Hz), 106.57, 104.79, 95.85, 91.54, 20.24.

^{19}F NMR (376 MHz, $CDCl_3$): δ (ppm) = –61.50.

HRMS (ESI neg) m/z for $C_{25}H_{12}F_6Br_2N^-$ [M-H$^+$]$^-$: calculated: 693.8969; found: 693.8968.

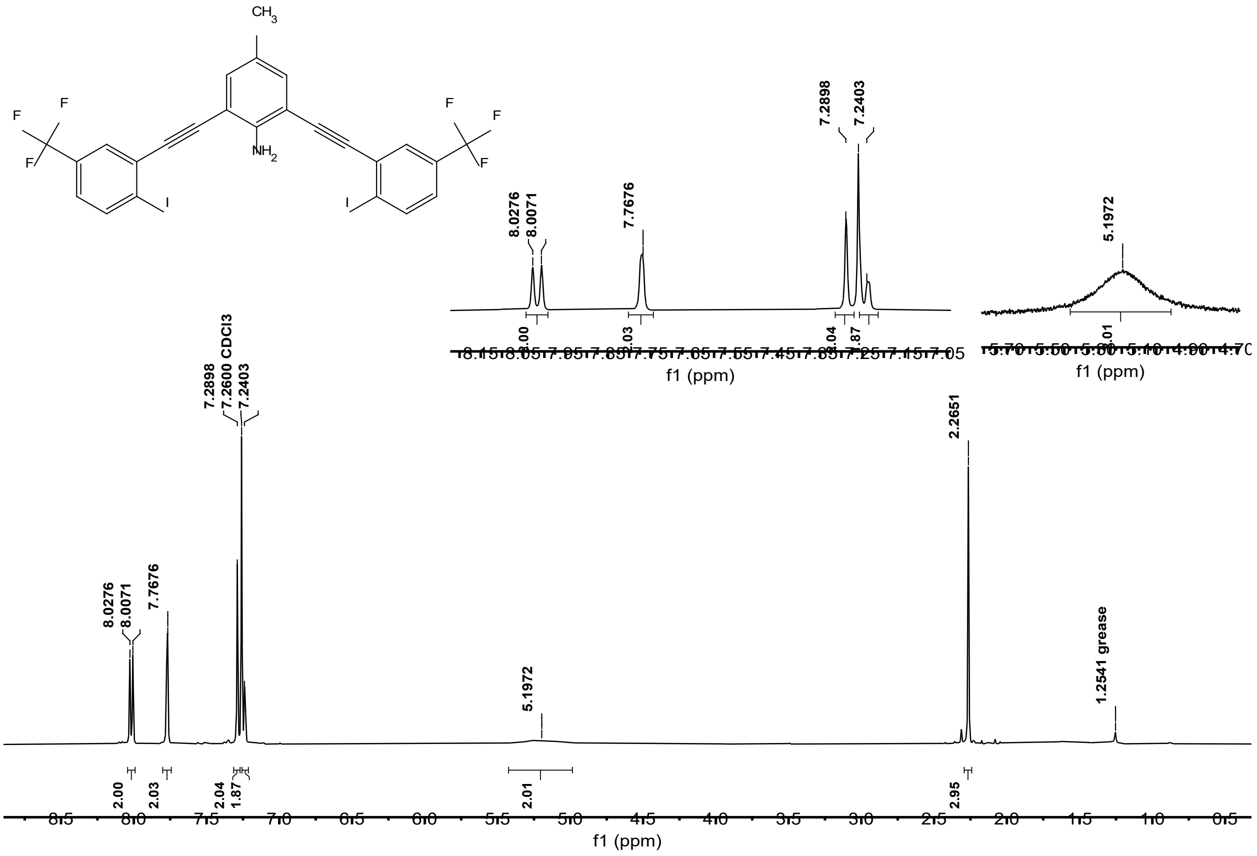

Figure S67. ^{1}H NMR of **Me-G3XB** (400 MHz, $CDCl_3$).

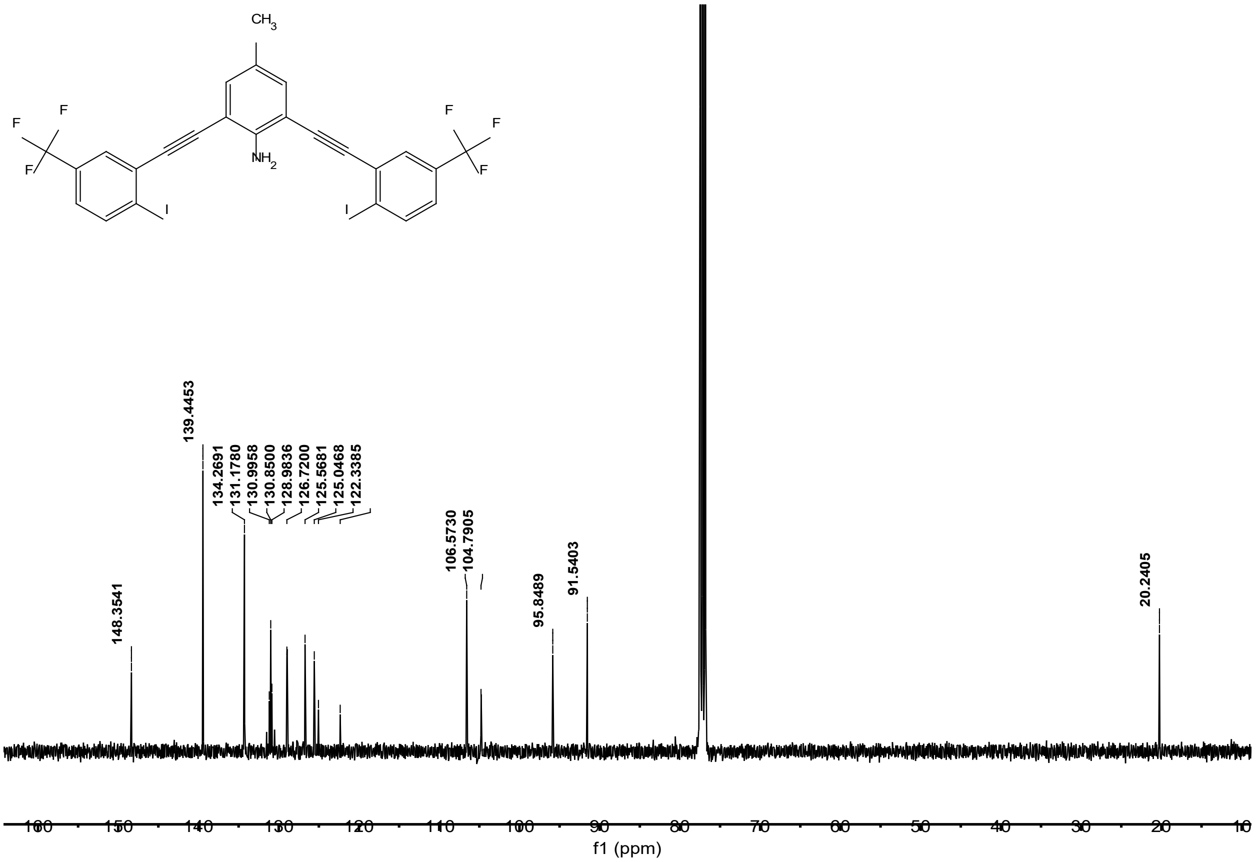

Figure S68. ^{13}C NMR of **Me-G3XB** (100 MHz, $CDCl_3$).

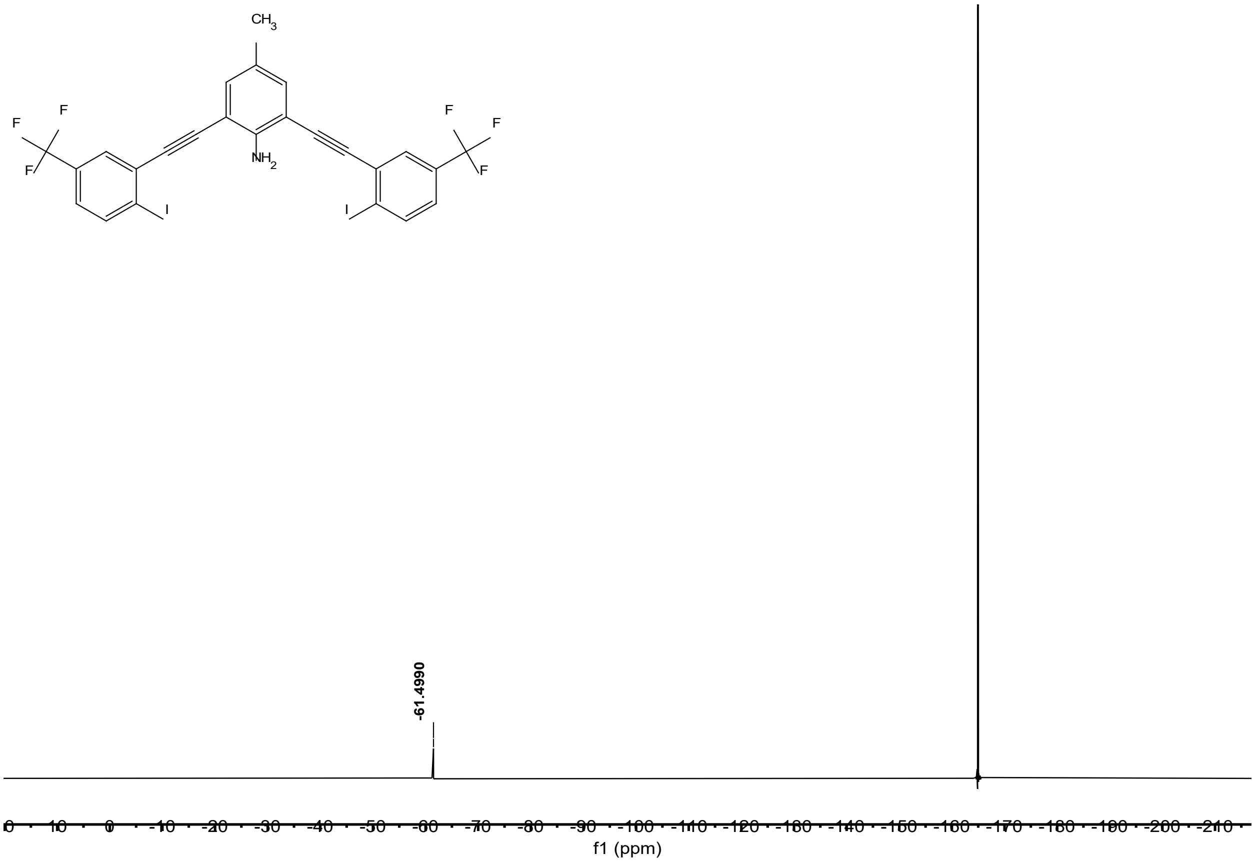

Figure S69. ^{19}F NMR of **Me-G3XB** (376 MHz, $CDCl_3$). Hexafluorobenzene (C_6F_6) internal reference.

3,5-bis(2-ethynyl-4-(trifluoromethyl)bromophenyl)-(trifluoromethyl)benzene (nHBeXBBr). To an oven dried Schlenk flask was charged with 3,5-bisethynyl-trifluoromethylbenzene **(2f)** (0.234g 1.2 mmol), then vacuumed and backfilled with dry N_2 gas (3x). Bis(triphenylphosphine)palladium (II) dichloride (0.042 g, 0. 06 mmol) was added, vacuumed and backfilled with dry N_2 gas (3x). Copper (I) iodide (0.023 g, 0.12 mmol) was added, vacuumed and backfilled with dry N_2 (3x). The dry reagents were dissolved in 60 mL dry DMF. 1-Bromo-2-iodo-4-(trifluoromethyl)benzene (0.48 mL, 3 mmol) and N,N-diisopropylethylamine (1.03 mL, 6 mmol) were added to the DMF solution. The flask was carefully vacuumed and backfilled with dry N_2 (3x). The dark brown solution stirred overnight at room temperature. The reaction mixture was extracted with ethyl acetate then washed with water to remove any excess salts and catalysts, then was dried over magnesium sulfate and gravity filtered. Subsequent removal of DMF, hexanes and ethyl acetate by rotary evaporation left and brown solid that was purified by column chromatography (gradient from 100% Hexanes to 5% EtOAc/95% Hexanes) to afford **nHBeXBBr** (0.466 g, 60%) as a dull yellow solid.

^1H NMR (400 MHz, $CDCl_3$): δ (ppm) = 7.94 (s, 1H), 7.83 (s, 4H), 7.78 (d, J = 8.3 Hz, 2H), 7.48 (d, J = 8.4 Hz, 2H).

^{13}C NMR (100 MHz, CDCl$_3$): δ (ppm) = 137.73, 133.42, 132.12 (q, J = 33.2 Hz), 130.221 (q, J = 33.2 Hz), 130.37 (q, J = 4.0 Hz), 129.72, 128.82 (q, J = 3.7 Hz), 126.64 (q, J = 3.6 Hz), 125.62, 123.49 (q, J = 270.8 Hz), 124.07, 123.25 (q, J = 271.2 Hz), 92.71, 89.03.

^{19}F NMR (376 MHz, CDCl$_3$): δ (ppm) = –61.32, -61.48.

Elemental analysis, Found: C, 47.02; H, 1.39%. Calc. for $C_{25}H_9Br_2F_9$: C, 46.91; H, 1.42%.

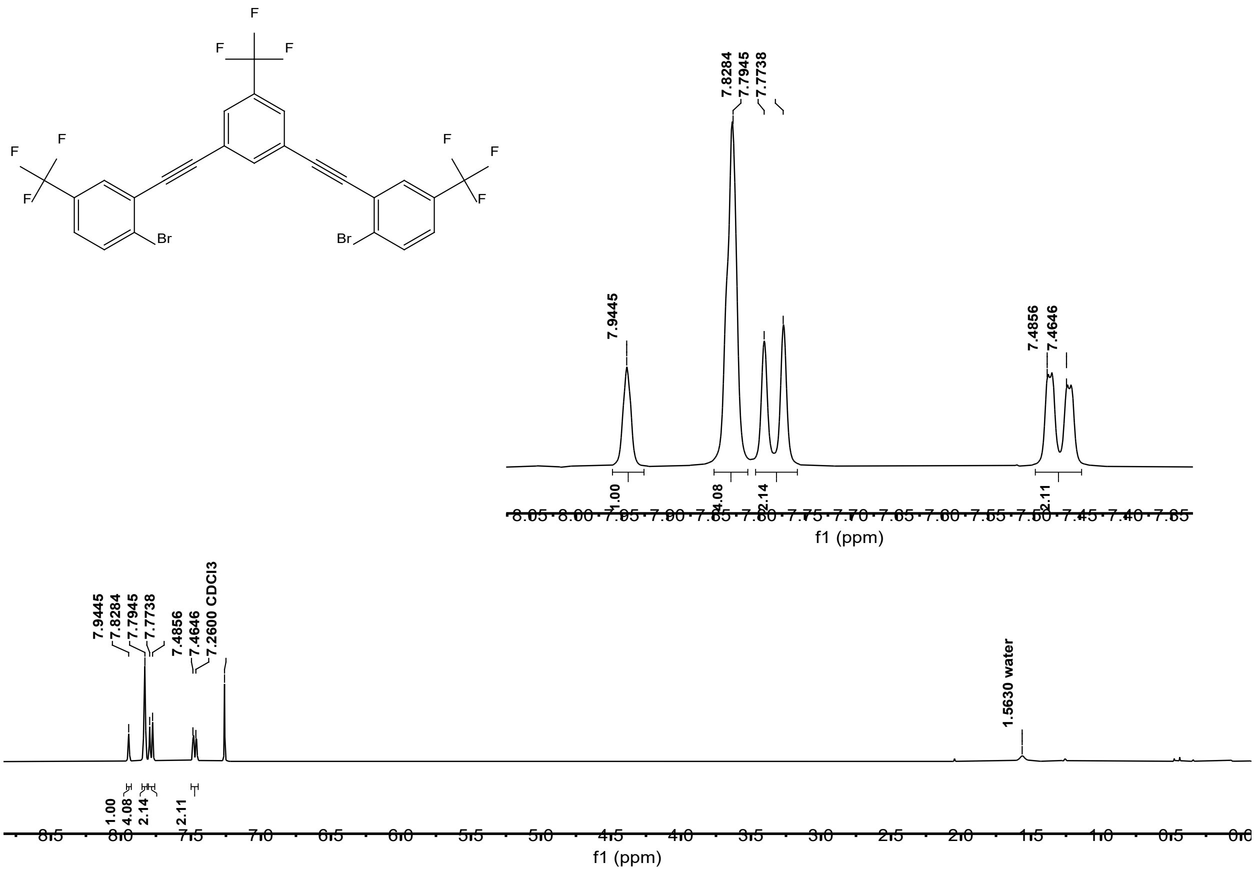

Figure S70. ^{1}H NMR of **nHBeXBBr** (400 MHz, CDCl$_3$).

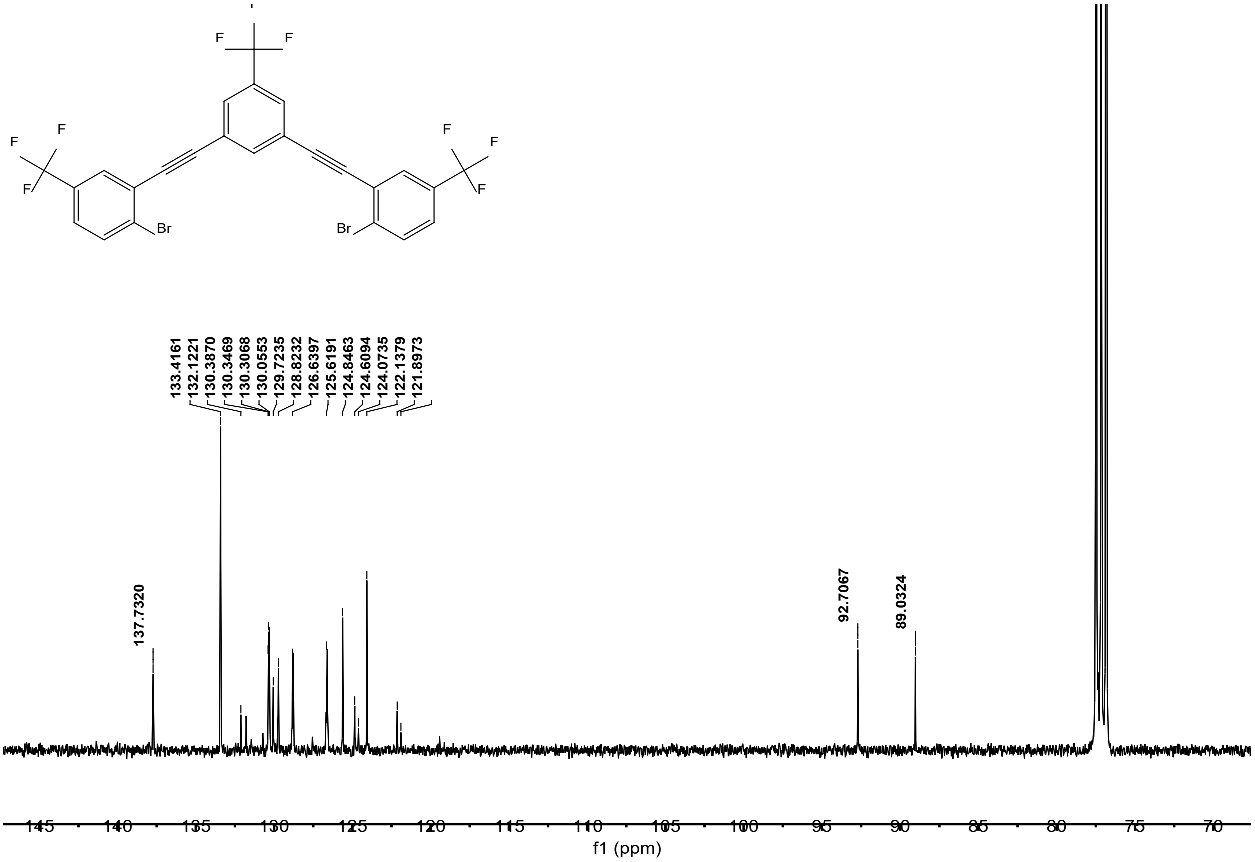

Figure S71. ^{13}C NMR of **nHBeXBBr** (100 MHz, $CDCl_3$).

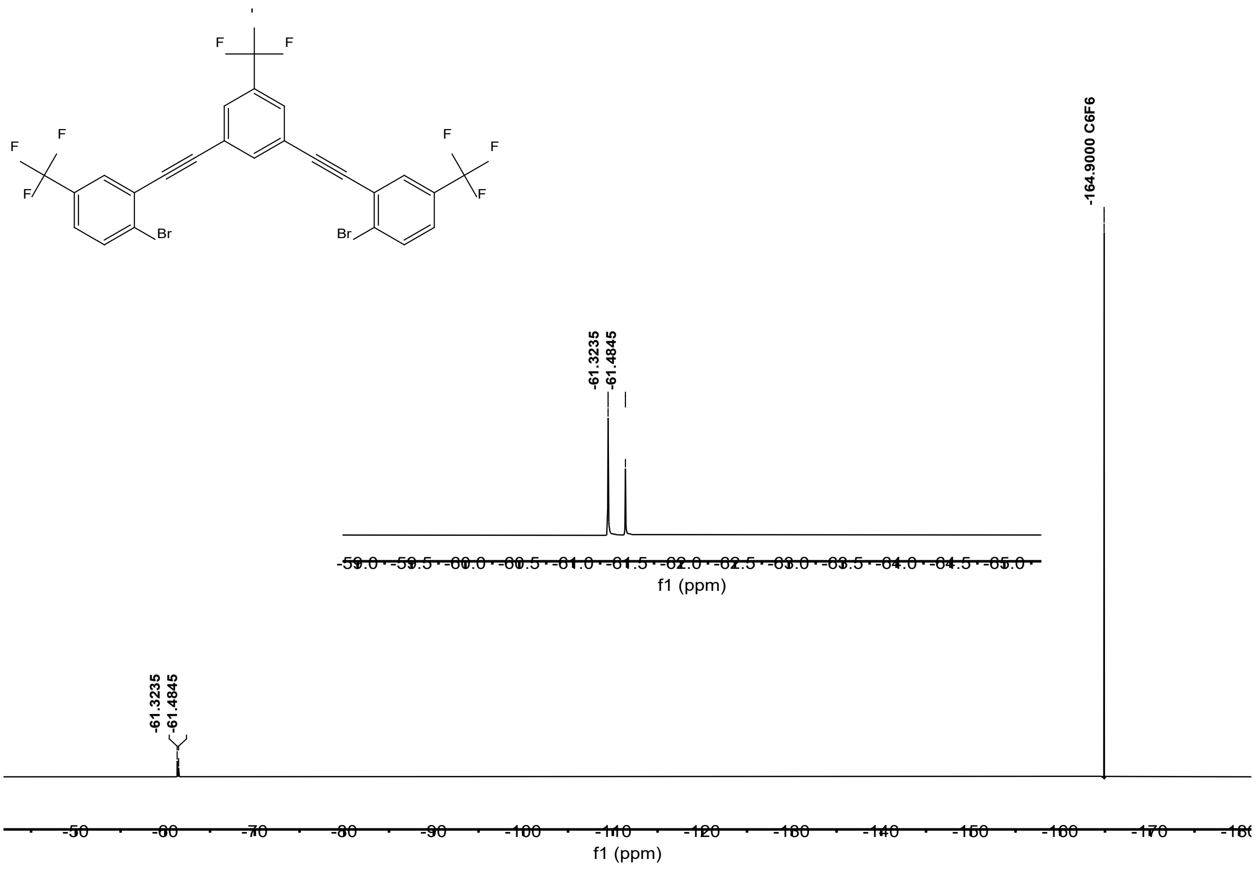

Figure S72. ^{19}F NMR of **nHBeXBBr** (376 MHz, $CDCl_3$). Hexafluorobenzene (C_6F_6) internal reference.

2,6-bis(2-ethynyl-4-(trifluoromethyl)iodophenyl)-4-trifluoromethylbenzene (nHBeXB). **nHBeXBBr** (0.200 g, 0.313 mmol), copper iodide (0.006 g, 0.030 mmol), sodium iodide (0.200 g, 1.33 mmol) were added to a 10-20 mL microwave reaction vial containing a stir bar and dissolved in 13 mL 1,4-dioxane. To the vibrant yellow reaction mixture, trans-N,N'-dimethylcyclohexane-1,2-diamine (0.1 mL) was added. The microwave vial was sealed and placed in a Biotage Initiator+ microwave reactor for 17 hours at 150 ˚C. After cooling, the reaction was run through pipet silica plug with EtOAc to remove catalysts and salts. The EtOAc crude was then ran through GCMS in order to obtain % conversion of bromines to iodines. If the conversion from bromine to iodine was 99% or greater, then the reaction was concentrated. The beige/yellow product (0.144 g, 63%) was obtained after purified via high performance liquid chromatography (reverse phase column gradient from 80% water/20% acetonitrile to 100% acetonitrile).

^{1}H NMR (400 MHz, $CDCl_3$): δ (ppm) = 8.04 (d, J = 8.3 Hz, 2H), 7.97 (s, 1H), 7.84 (s, 2H), 7.79 (s, 2H), 7.30 (d, J = 8.3 Hz, 2H).

^{13}C NMR (100 MHz, $CDCl_3$): δ (ppm) = 139.73, 137.58, 132.15 (q, J = 34.2 Hz), 131.21 (q, J = 32.8 Hz), 130.04, 129.32 (q, J = 3.7 Hz), 128.72(q, J = 4.0 Hz), 126.42 (q, J = 3.7 Hz), 124.12, 123.62 (q, J = 270.5 Hz), 123.27 (q, J = 271.6 Hz), 105.46, 92.62, 91.82.

^{19}F NMR (376 MHz, CDCl$_3$): δ (ppm) = –61.51, -61.57.

Elemental analysis, Found: C, 42.29; H, 1.14%. Calc. for $C_{25}H_9I_2F_9$: C, 40.90; H, 1.24%.

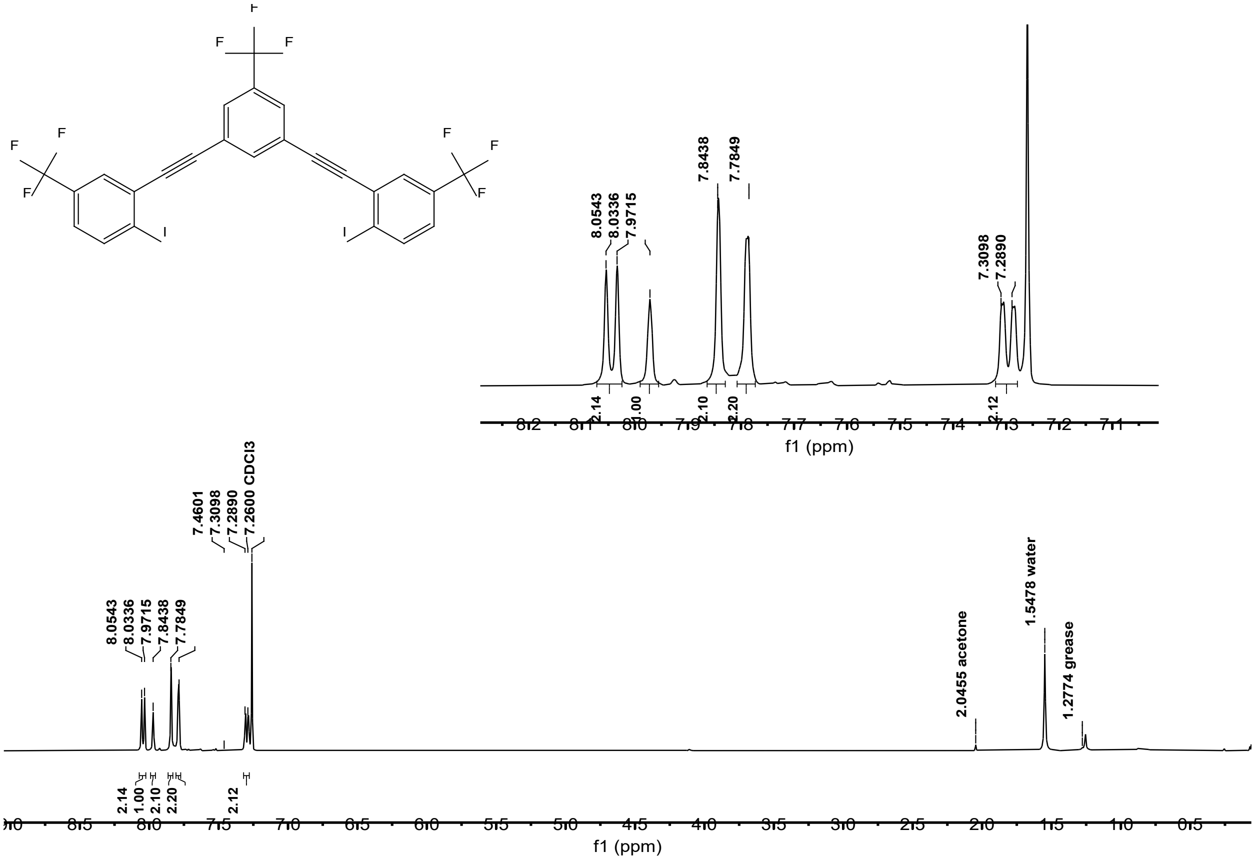

Figure S73. ^{1}H NMR of **nHBeXB** (400 MHz, CDCl$_3$).

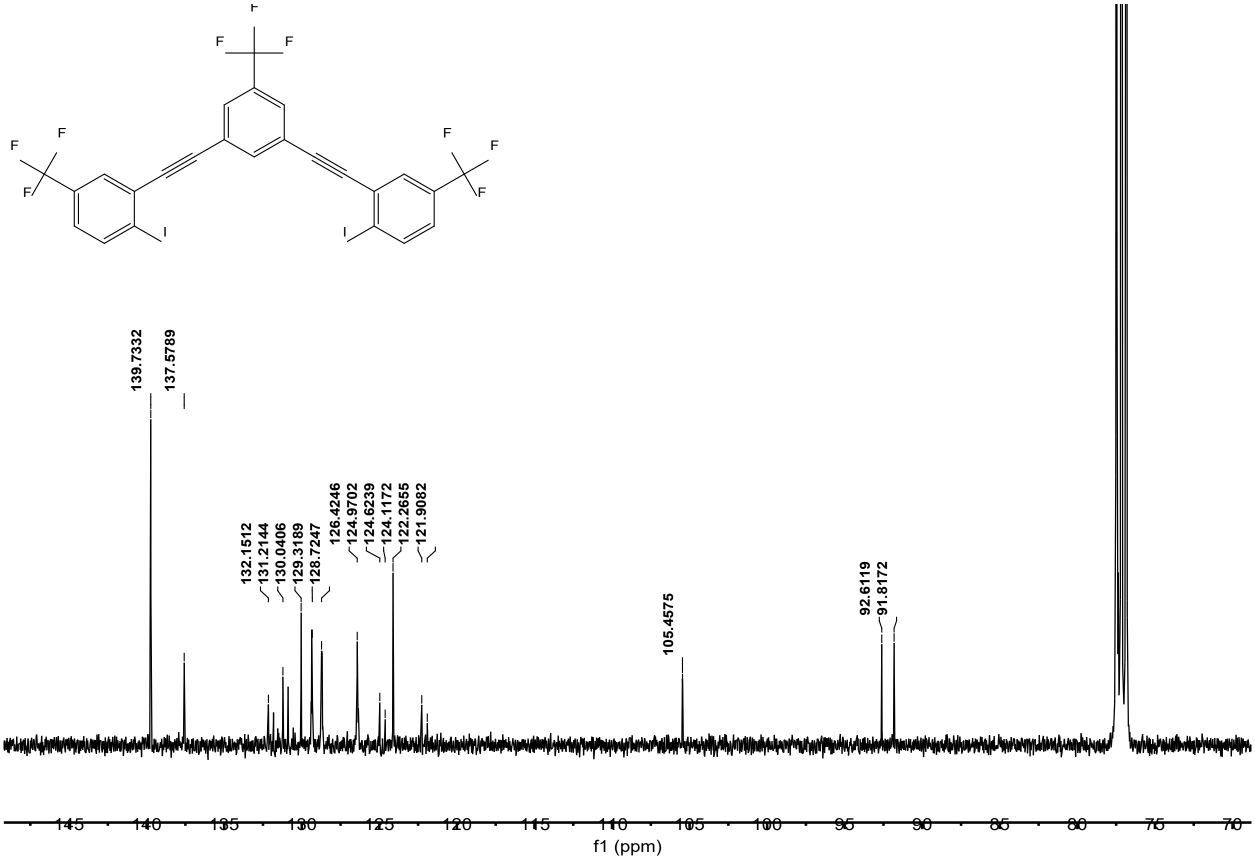

Figure S74. ^{13}C NMR of **nHBeXB** (100 MHz, $CDCl_3$).

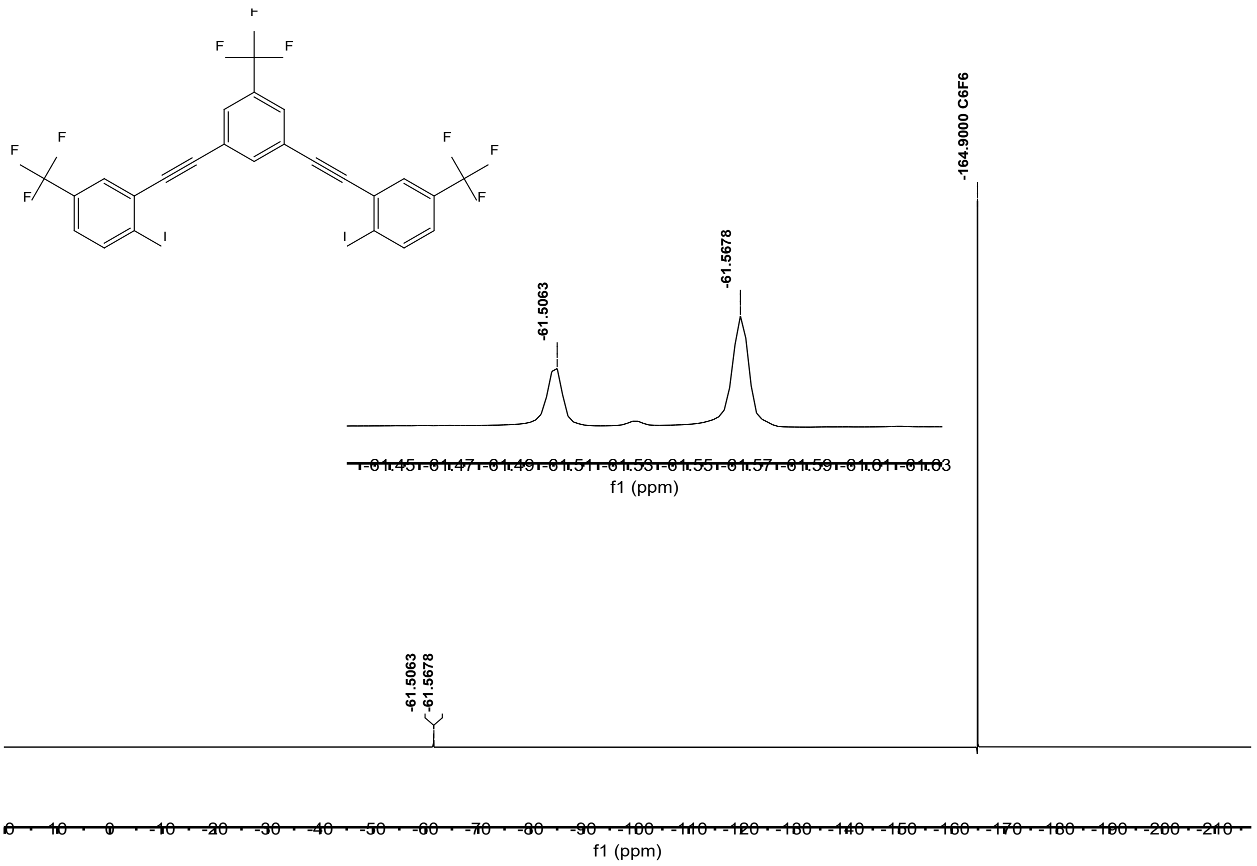

Figure S75. ^{19}F NMR of **nHBeXB** (376 MHz, $CDCl_3$). Hexafluorobenzene (C_6F_6) internal reference.

NMR titration Details and Data

All experiments were performed on a Varian Drive Direct 500 MHz NMR Spectrometer. C_6D_6 was dried over 3 Å molecular sieves. THA$^+$ salts (Cl^-, Br^-, I^-) was dried under vacuum and stored in a desiccator.

^1H NMR Titrations

Stock solutions of all receptors (3.993-5.745 mM) were prepared in 1.4 mL of deuterated benzene (C_6D_6). 0.500 mL aliquots from each stock solution were syringed into three separate NMR tubes with screw caps and septa. The stock solution of each host was then used to make guest solutions. After obtaining free-host spectra of each receptor, aliquots of corresponding guest solution were added to their respective NMR tubes. Spectra were obtained after each addition (20x). A constant host concentration was maintained, while THA iodide salt concentrations gradually increased throughout titration (see data below). Intuitions of stoichiometric displacement led to the stepwise anion exchange model:

$$H + G \rightleftharpoons HG, K_{11} = [HG]/[H][G]$$

Dimerization and higher order binding were ruled out due to the emergence of an obvious pattern in residuals, unrealistic assigned shifts, poor convergence, and/or larger standard deviations. Bindfit[5] was used to refine the isothermal fits of multiple signals simultaneously.

Association constants for binding of THA iodide to all receptors are shown in Table S1. Full binding data and fitting parameters for each titration can be obtained from the Bindfit[5] using the following links.

Table S1. Association constants for binding of THA iodide to all receptors in C_6D_6 at 298 K.

Receptor	Assay 1 (M^{-1})	Assay 2 (M^{-1})	Assay 3 (M^{-1})	Average (M^{-1})
**G3XB**	428.8	406.5	410.8	415.6
**2F-G3XB**	152.9	177.4	179.6	170.0
**2H-G3XB**	69.9	75.5	70.3	71.9
**2Me-G3XB**	51.1	44.9	46.6	47.5
**2*t*Bu-G3XB**	54.1	51.0	49.2	51.4
**2MeO-G3XB**	60.2	63.6	63.2	62.4
**Cl-G3XB**	330.0	317.7	345.4	331.1
**F-G3XB**	236.4	274.4	241.1	250.7
**H-G3XB**	194.6	199.1	188.8	194.2
**Me-G3XB**	182.0	171.7	170.2	174.6
**nHBeXB**	12.4	4.4	8.0	8.3
**G3HB**	36.7	22.5	23.2	27.5

G3XB with THA iodide

Assay 1 K_a= 428.8 M^{-1} http://app.supramolecular.org/bindfit/view/d05dc4b3-cdea-4bc5-a05a-cf9ff564b20e

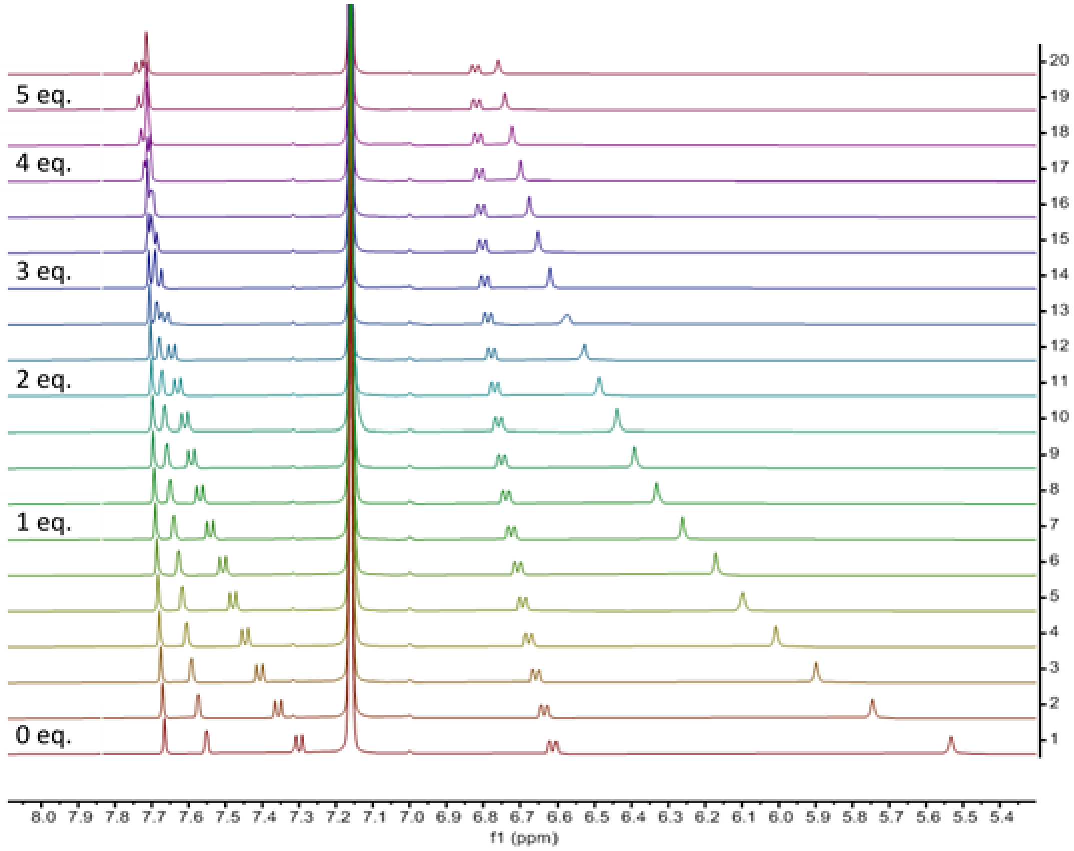

Figure S106-1. ^{1}H NMR spectra of **G3XB** upon addition of increasing equivalents of THA·I (C_6D_6, 298 K, 500 MHz).

Assay 2 K_a= 406.5 M^{-1} http://app.supramolecular.org/bindfit/view/f6b13944-604f-4a0d-89a6-894a7605e731

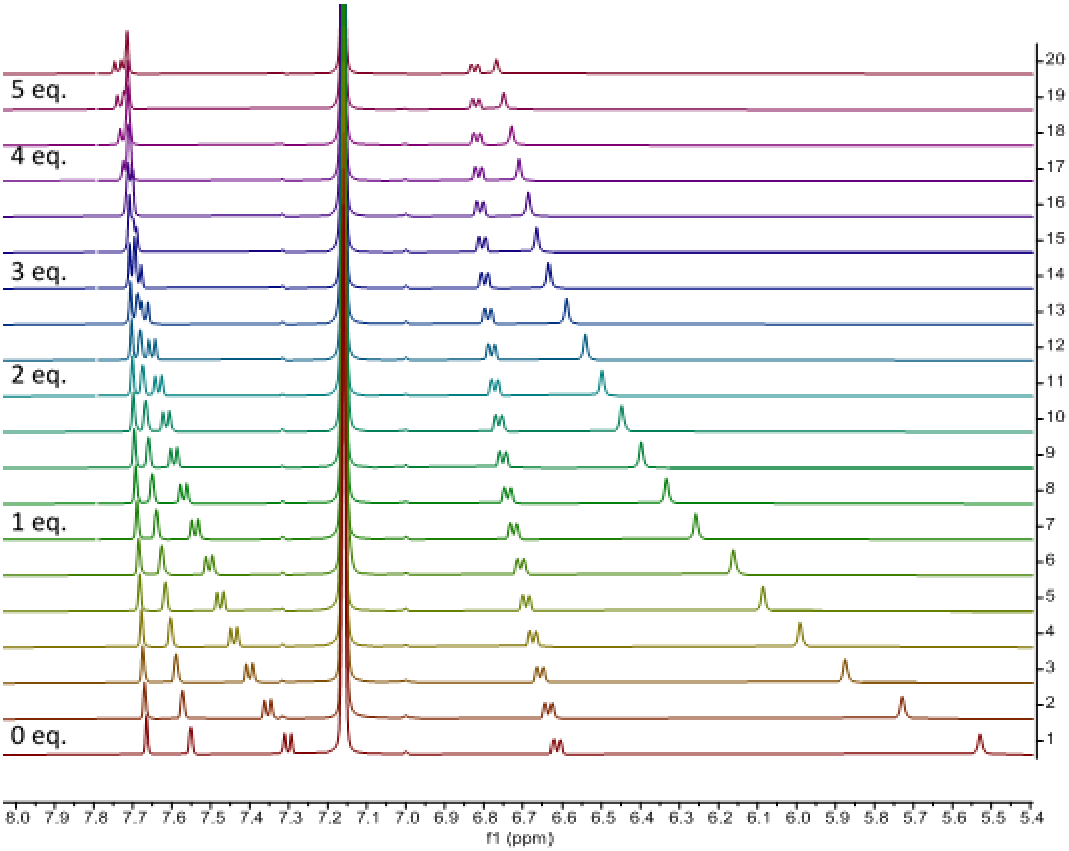

Figure S116-2. ^{1}H NMR spectra of **G3XB** upon addition of increasing equivalents of THA·I (C_6D_6, 298 K, 500 MHz).

Assay 3 K_a= 410.8 M^{-1} http://app.supramolecular.org/bindfit/view/d6cdf7b6-4e20-40a2-8af2-91b2beade8c0

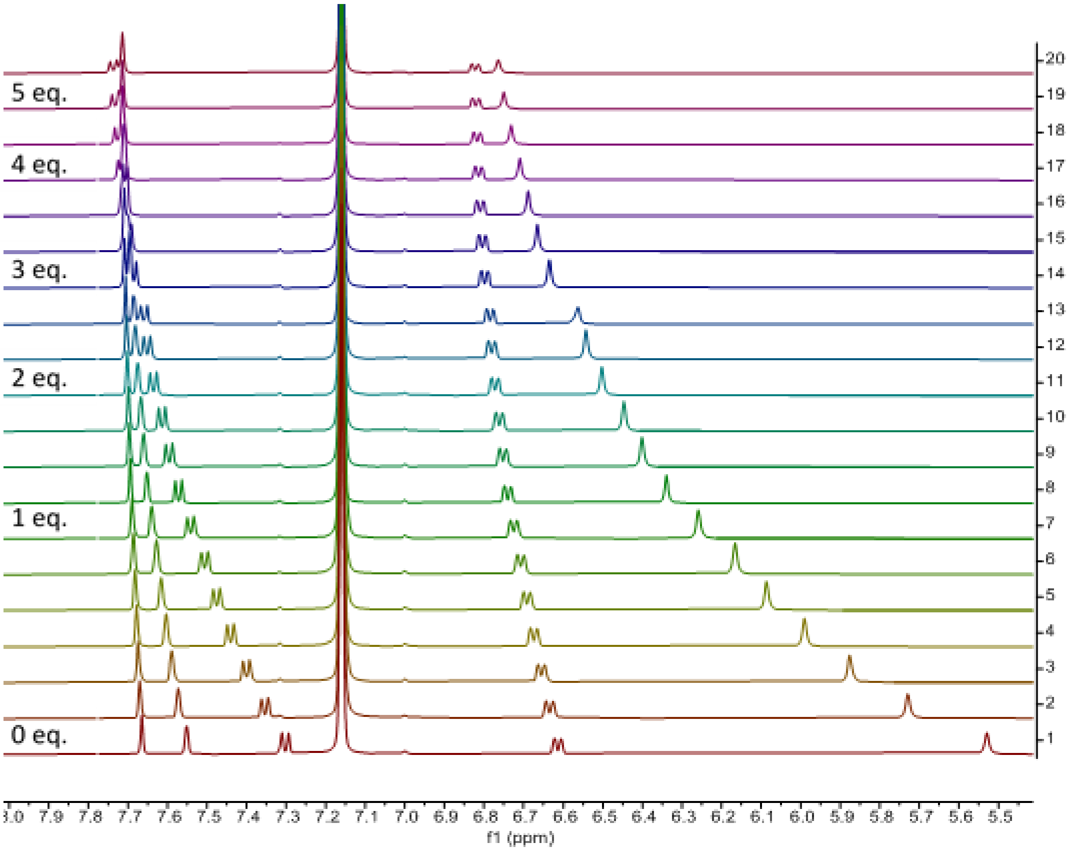

Figure S126-3. ^{1}H NMR spectra of **G3XB** upon addition of increasing equivalents of THA·I (C_6D_6, 298 K, 500 MHz).

2F-G3XB with THA iodide

Assay 1 K_a= 152.9 M^{-1} http://app.supramolecular.org/bindfit/view/e2455db7-87fd-4a8d-b961-b6d6f52084b5

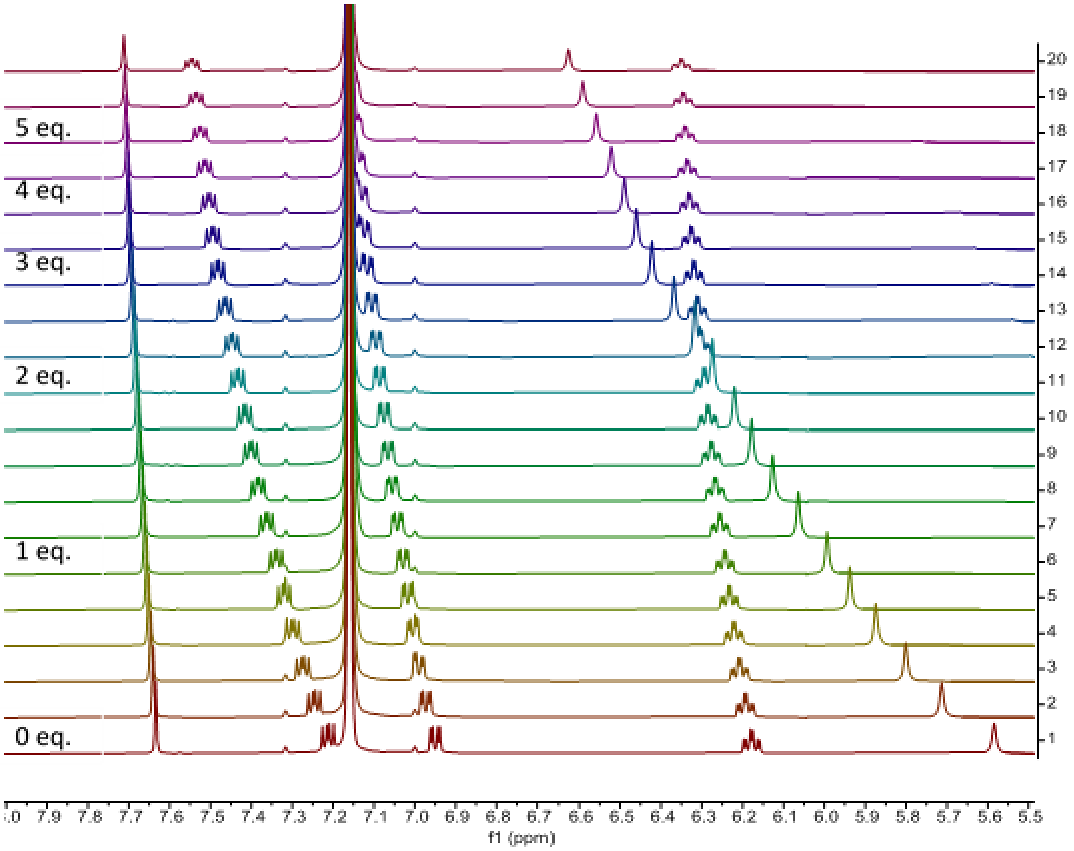

Figure S137-1. ^{1}H NMR spectra of **2F-G3XB** upon addition of increasing equivalents of THA·I (C_6D_6, 298 K, 500 MHz).

Assay 2 K_a= 177.4 M^{-1} http://app.supramolecular.org/bindfit/view/2bf8af11-212f-4434-809e-b11fe57f9649

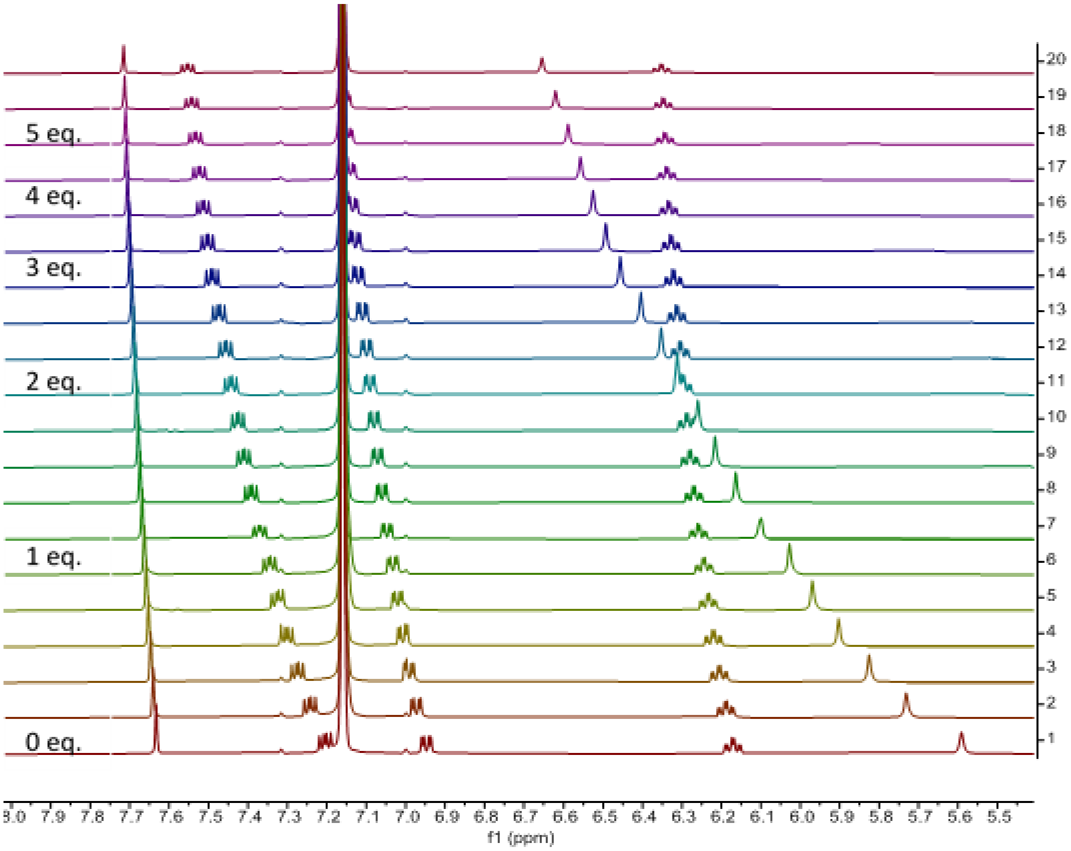

Figure S147-2. ^{1}H NMR spectra of **2F-G3XB** upon addition of increasing equivalents of THA·I (C_6D_6, 298 K, 500 MHz).

Assay 3 K_a= 179.6 M^{-1} http://app.supramolecular.org/bindfit/view/3646ae56-682e-467c-8f3f-0c1e1cfcb685

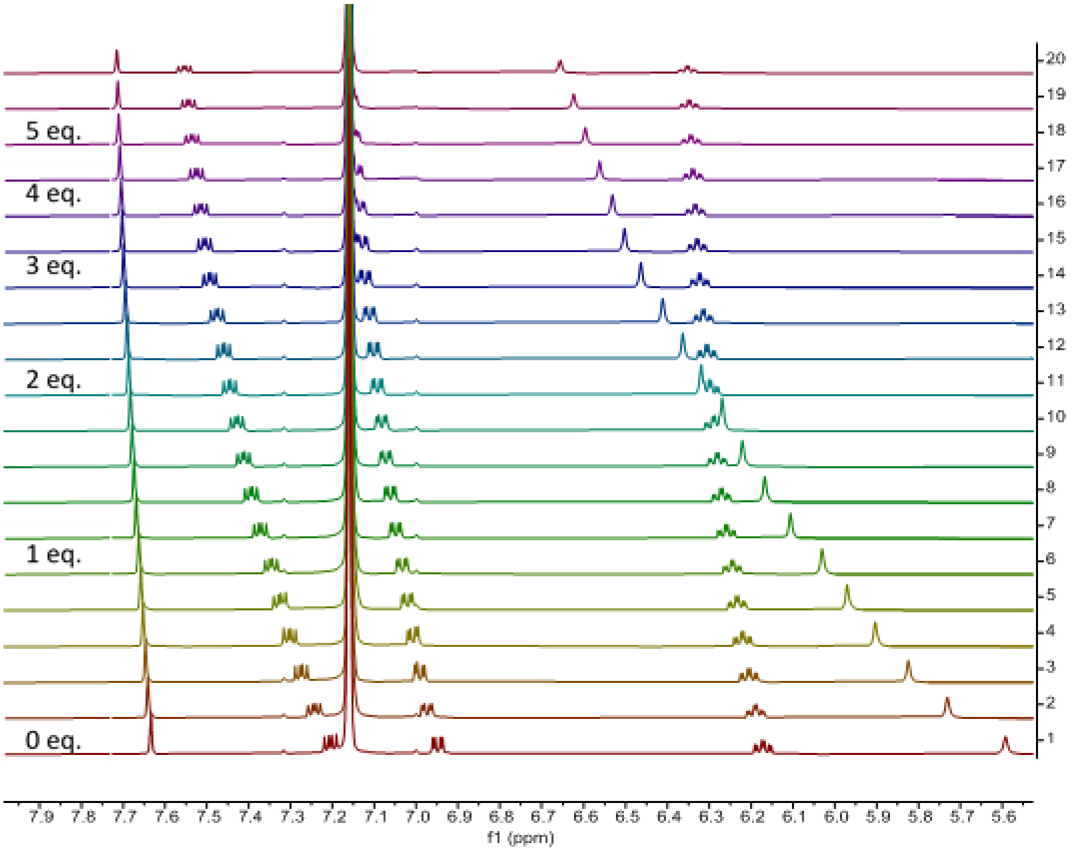

Figure S157-3. ^{1}H NMR spectra of **2F-G3XB** upon addition of increasing equivalents of THA·I (C_6D_6, 298 K, 500 MHz).

2H-G3XB with THA iodide

Assay 1 K_a= 69.9 M^{-1} http://app.supramolecular.org/bindfit/view/4e544414-0f49-474d-b021-4730b8a315ea

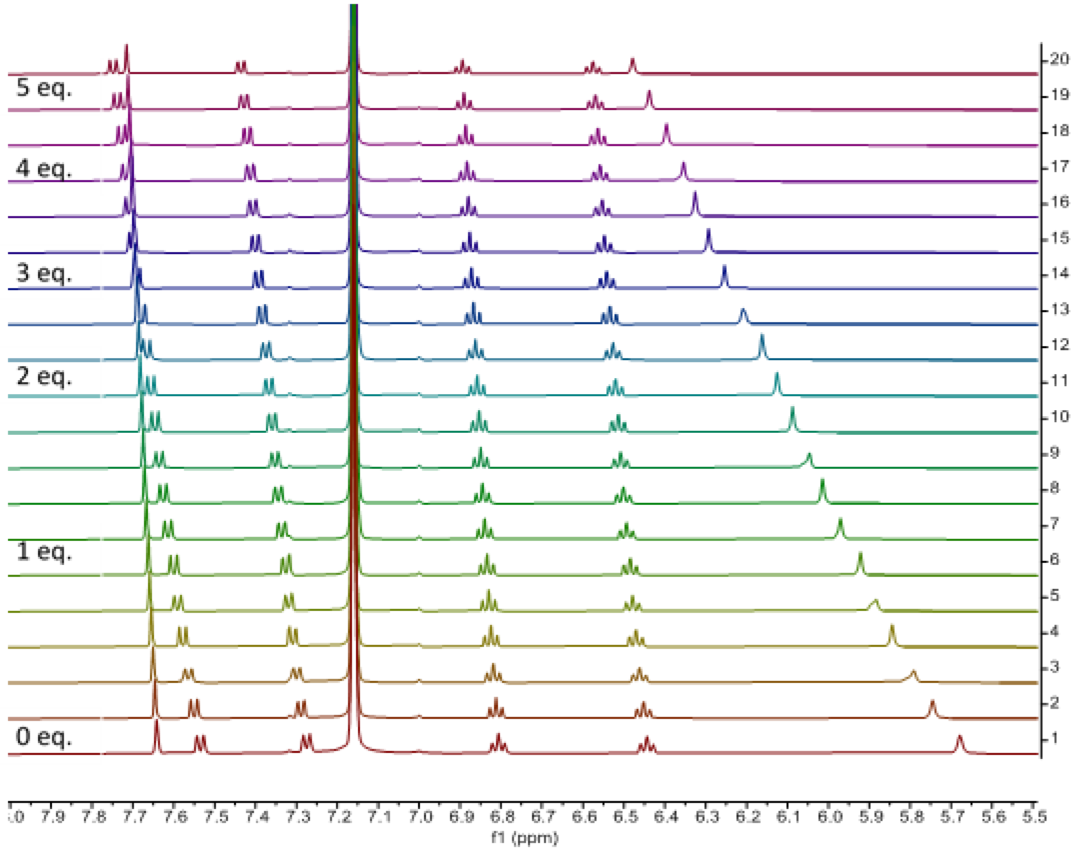

Figure S168-1. ^{1}H NMR spectra of **2H-G3XB** upon addition of increasing equivalents of THA·I (C_6D_6, 298 K, 500 MHz).

Assay 2 K_a= 75.5 M^{-1} http://app.supramolecular.org/bindfit/view/31ef807a-a956-4372-9614-45f31dd9c034

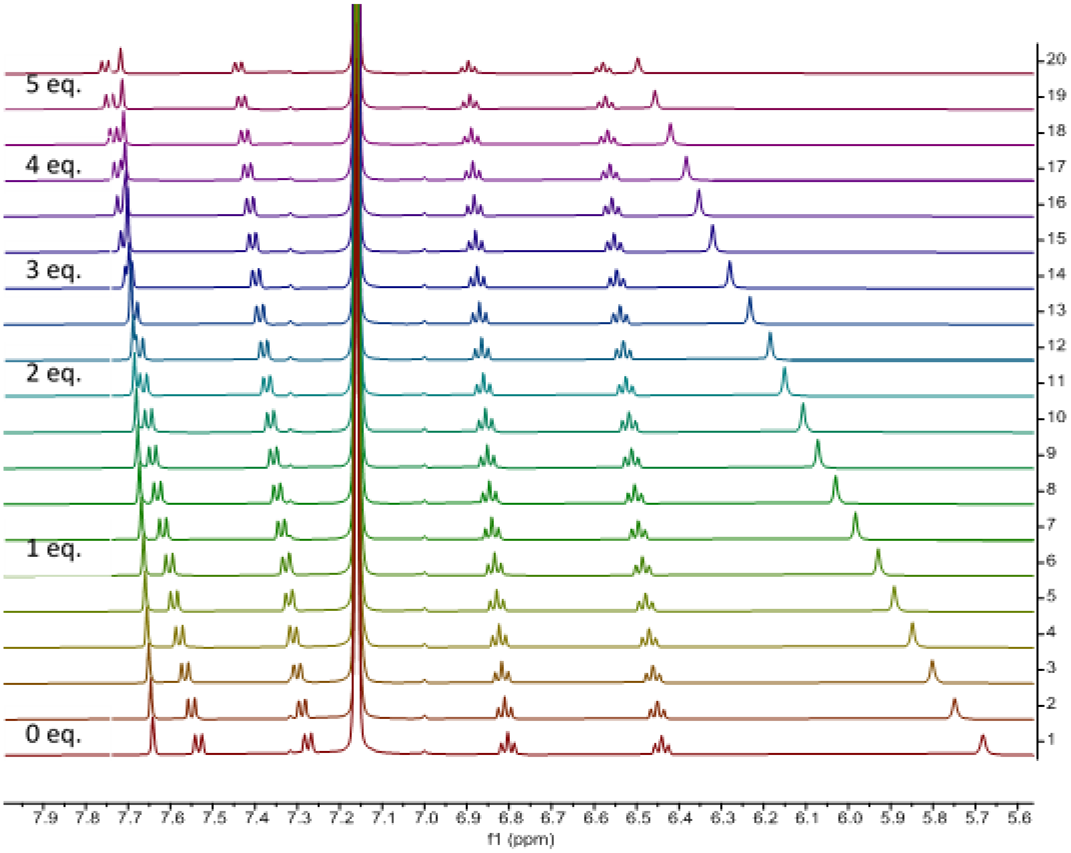

Figure S178-2. ^{1}H NMR spectra of **2H-G3XB** upon addition of increasing equivalents of THA·I (C_6D_6, 298 K, 500 MHz).

Assay 3 K_a= 70.3 M^{-1} http://app.supramolecular.org/bindfit/view/f27bf235-649a-45d9-a5f8-97a8130e9fcc

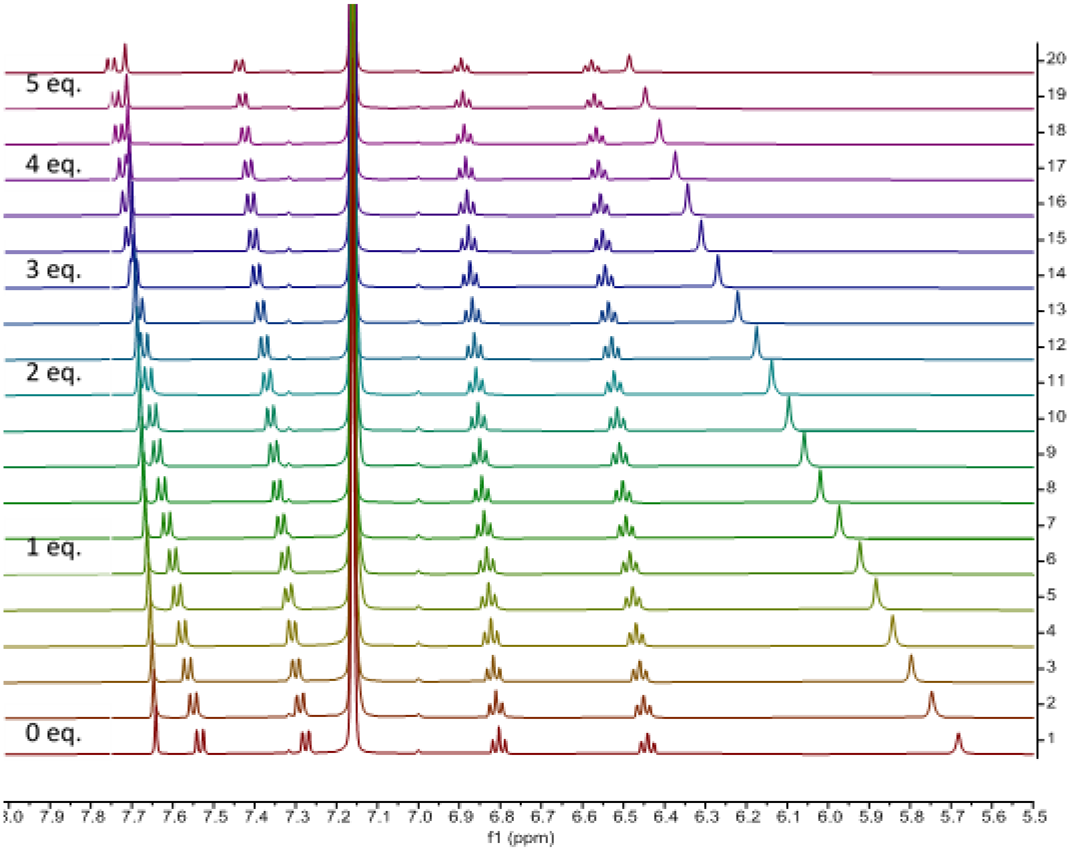

Figure S188-3. ^{1}H NMR spectra of **2H-G3XB** upon addition of increasing equivalents of THA·I (C_6D_6, 298 K, 500 MHz).

2Me-G3XB with THA iodide

Assay 1 K_a= 51.1 M^{-1} http://app.supramolecular.org/bindfit/view/f3b0725c-ef3b-4907-9cc0-439160c61860

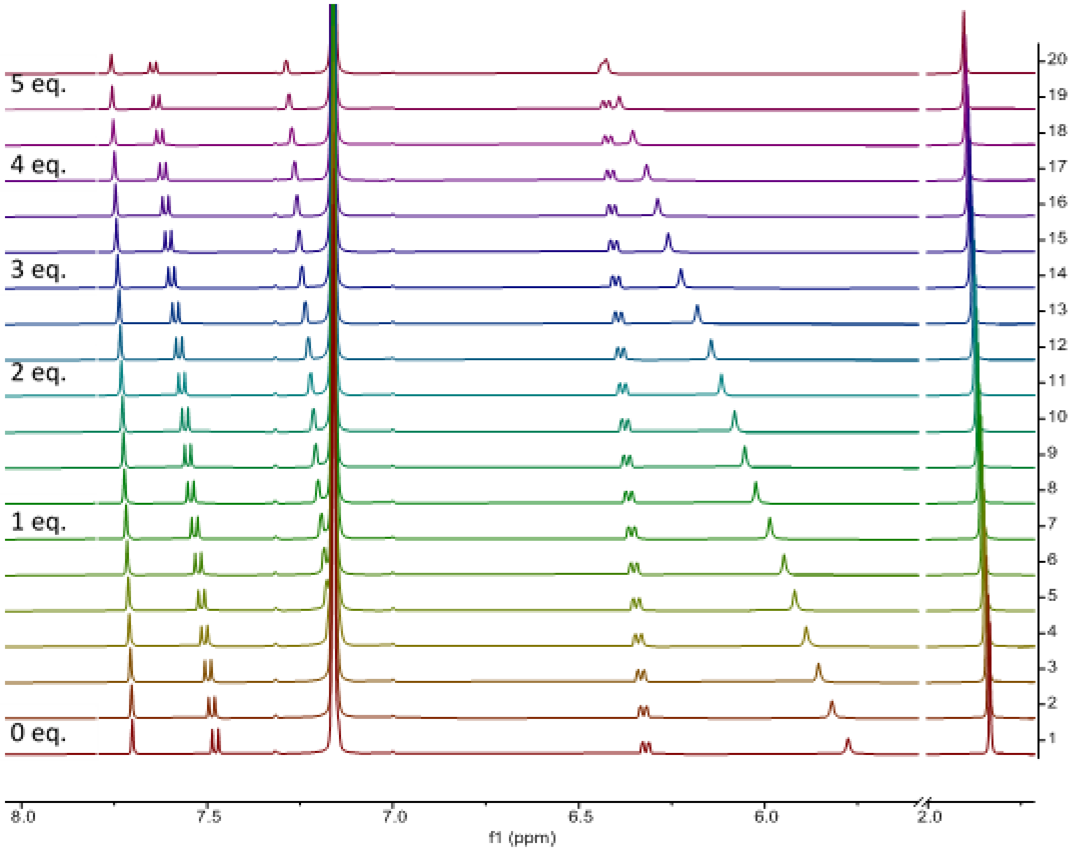

Figure S199-1. ^{1}H NMR spectra of **2Me-G3XB** upon addition of increasing equivalents of THA·I (C_6D_6, 298 K, 500 MHz).

Assay 2 K_a= 44.9 M^{-1} http://app.supramolecular.org/bindfit/view/a83b6928-477c-47d9-b2ef-a5bb372d5e63

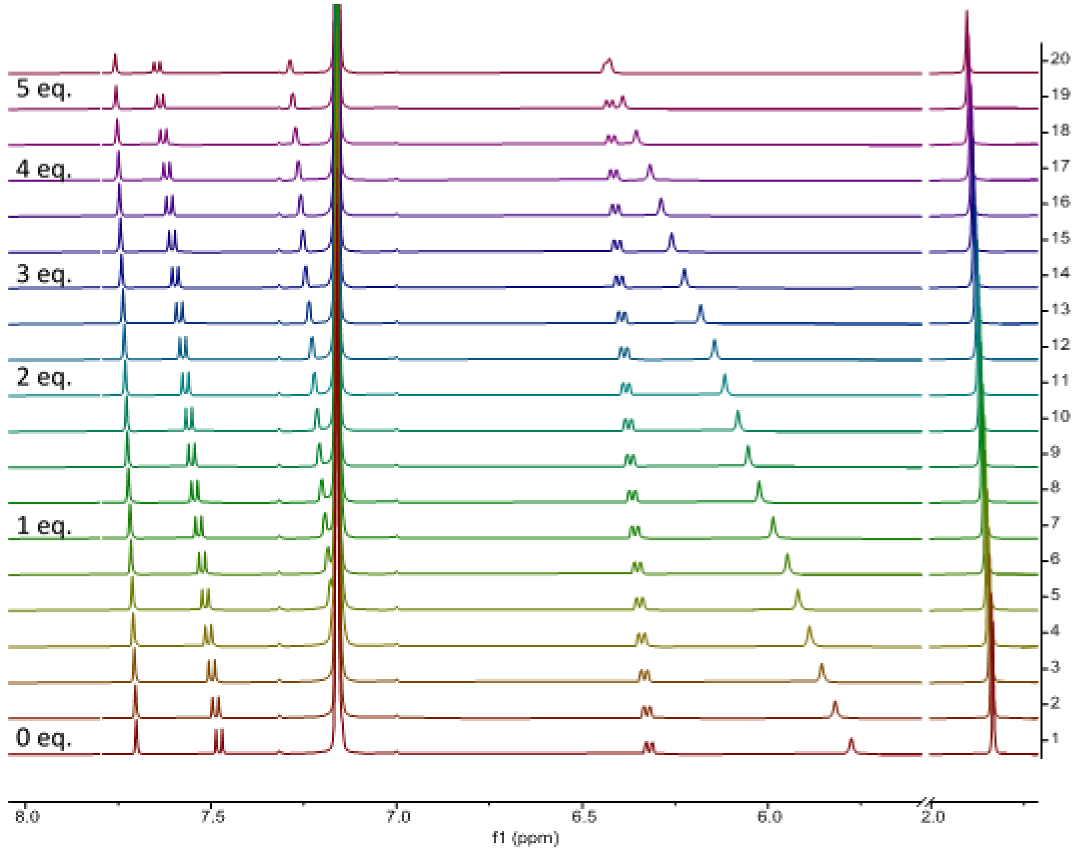

Figure S209-2. ^{1}H NMR spectra of **2Me-G3XB** upon addition of increasing equivalents of THA·I (C_6D_6, 298 K, 500 MHz).

Assay 3 K_a= 46.6 M^{-1} http://app.supramolecular.org/bindfit/view/54c86164-8816-47ad-b588-8eaad5e20311

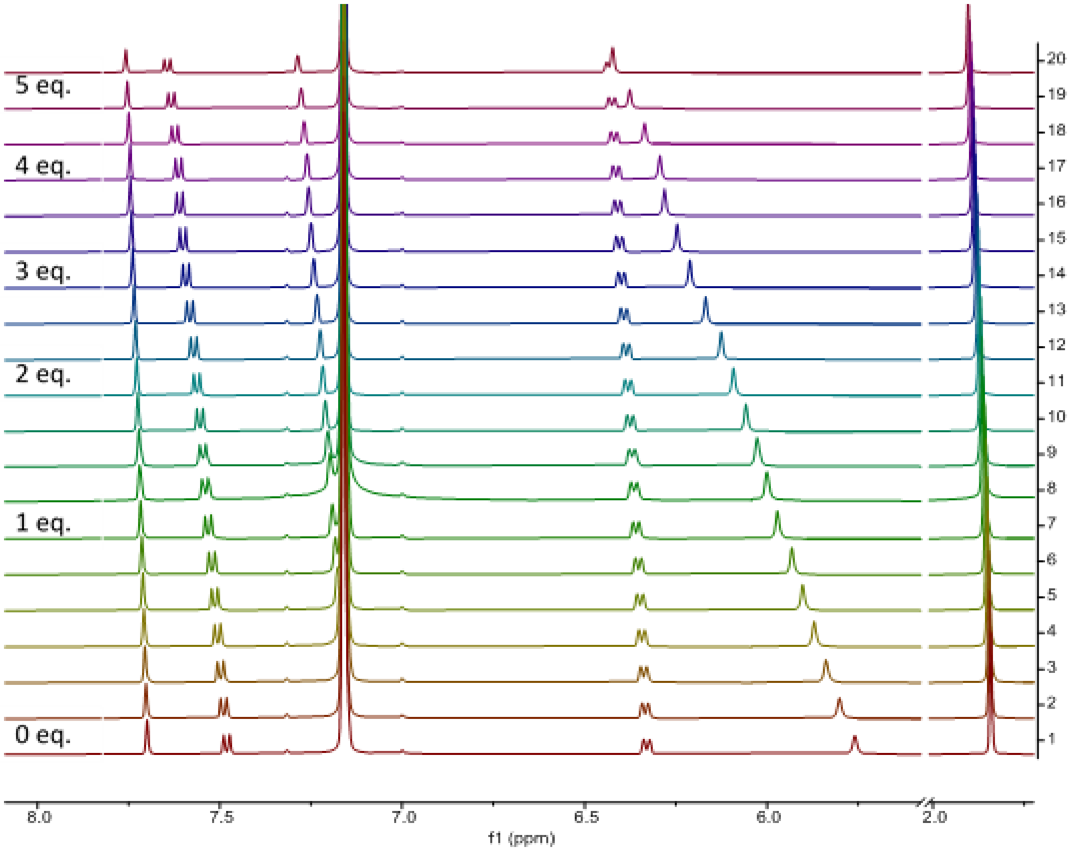

Figure S219-3. ^{1}H NMR spectra of **2Me-G3XB** upon addition of increasing equivalents of THA·I (C_6D_6, 298 K, 500 MHz).

2*t*Bu-G3XB with THA iodide

Assay 1 K_a= 54.1 M^{-1} http://app.supramolecular.org/bindfit/view/683b035e-6918-4f6e-8995-c71aca7c4dcd

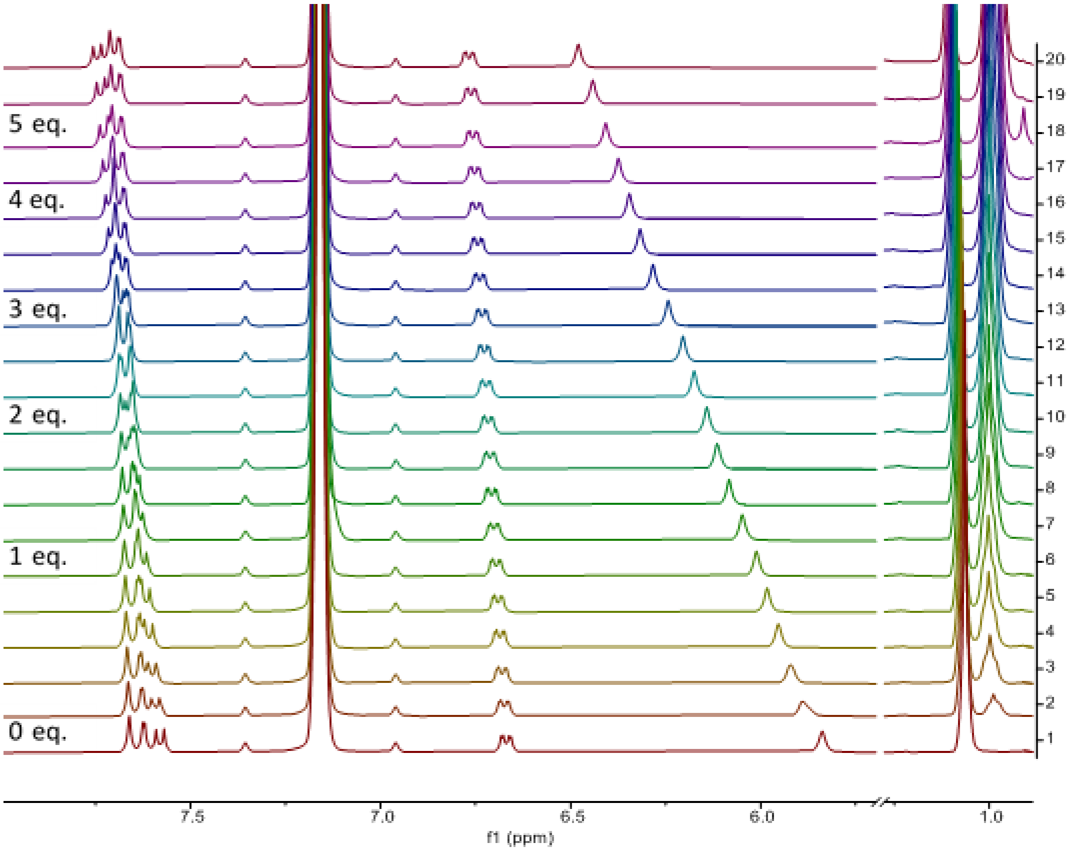

Figure S80-1. ^{1}H NMR spectra of **2*t*Bu-G3XB** upon addition of increasing equivalents of THA·I (C_6D_6, 298 K, 400 MHz).

Assay 2 K_a= 51.0 M^{-1} http://app.supramolecular.org/bindfit/view/fd255dc6-e87a-4e8c-84ca-b1f17ef54f0a

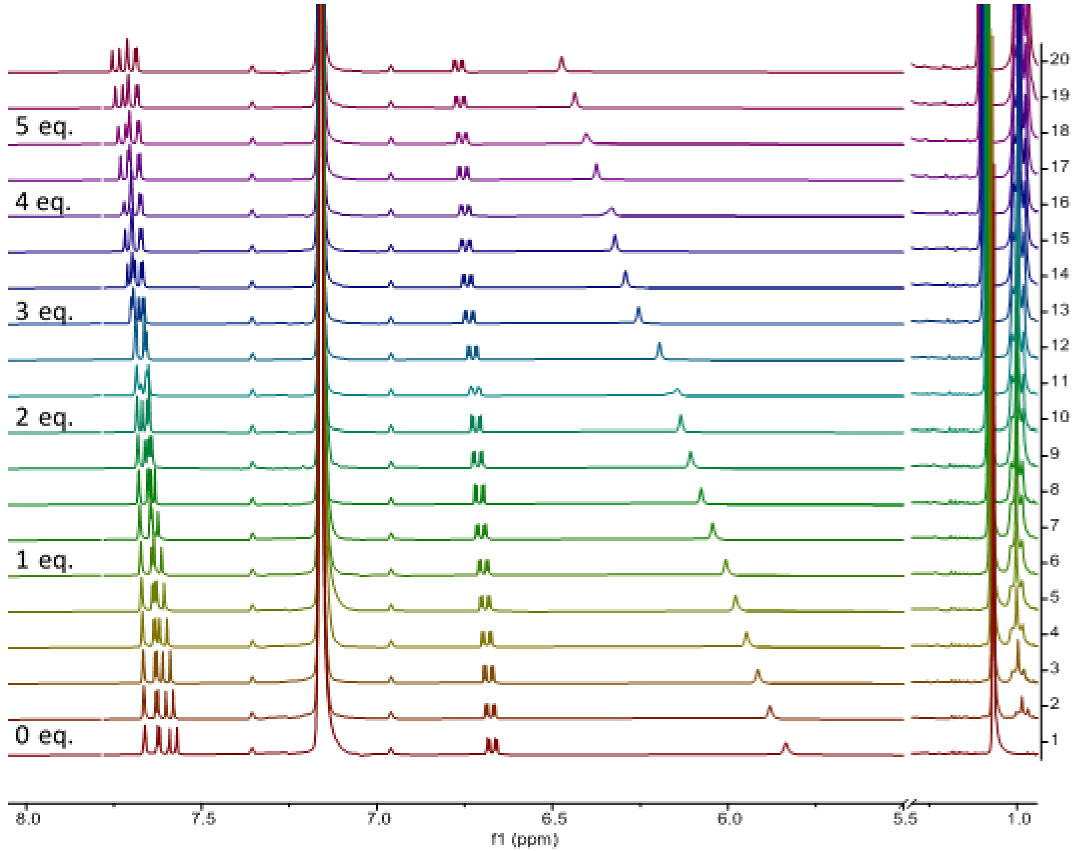

Figure S80-2. ^{1}H NMR spectra of **2*t*Bu-G3XB** upon addition of increasing equivalents of THA·I (C_6D_6, 298 K, 400 MHz).

Assay 3 K_a= 49.2 M^{-1} http://app.supramolecular.org/bindfit/view/429c33a3-777b-42b5-b81f-4d1995dc04bd

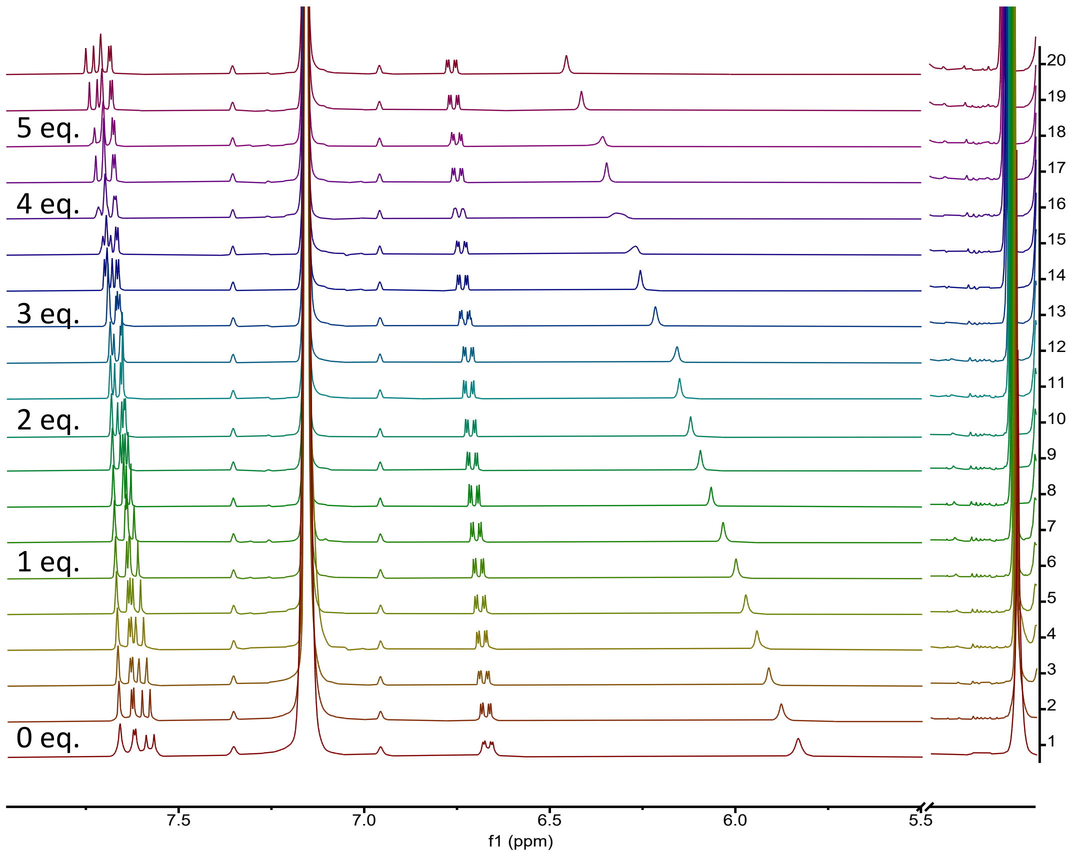

Figure S80-3. ^{1}H NMR spectra of **2*t*Bu-G3XB** upon addition of increasing equivalents of THA·I (C_6D_6, 298 K, 400 MHz).

2MeO-G3XB with THA iodide

Assay 1 K_a= 60.2 M^{-1} http://app.supramolecular.org/bindfit/view/e2d156d9-6430-4b9a-8181-8ba092d2dc8a

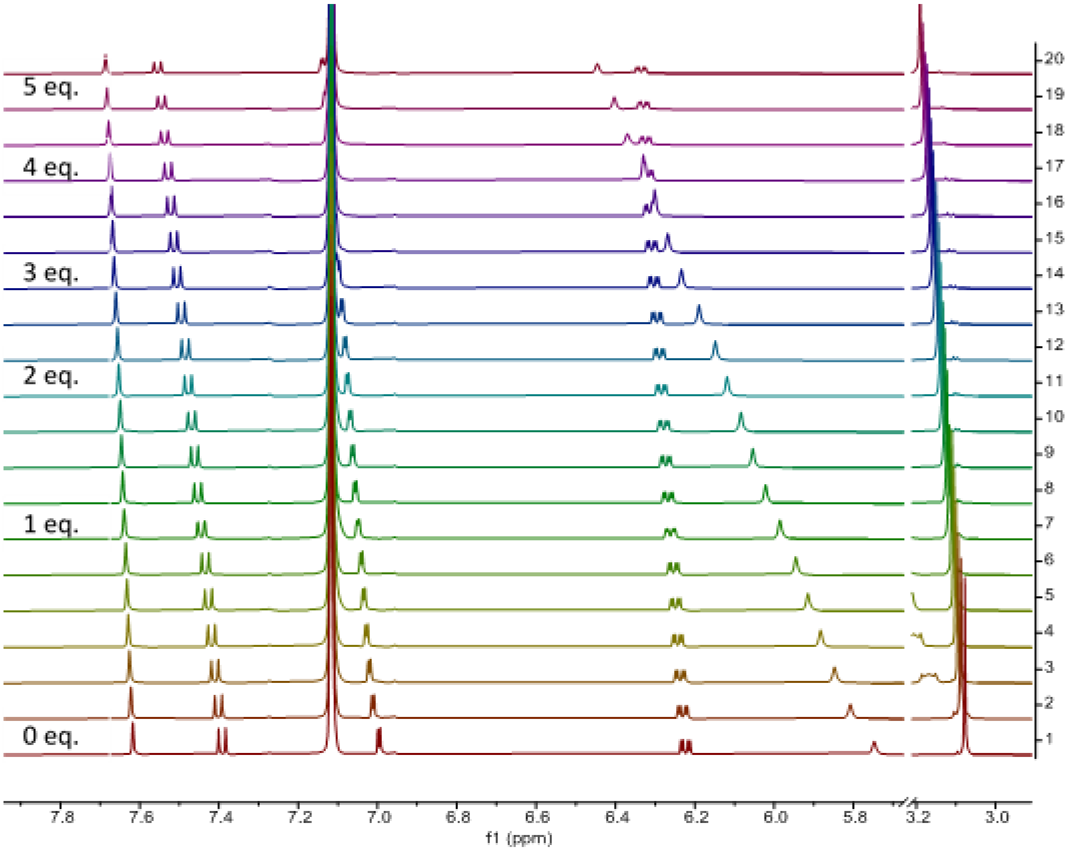

Figure S81-1. ^{1}H NMR spectra of **2MeO-G3XB** upon addition of increasing equivalents of THA·I (C_6D_6, 298 K, 500 MHz).

Assay 2 K_a= 63.6 M^{-1} http://app.supramolecular.org/bindfit/view/911973d0-3399-4738-92a4-fa05f18e5959

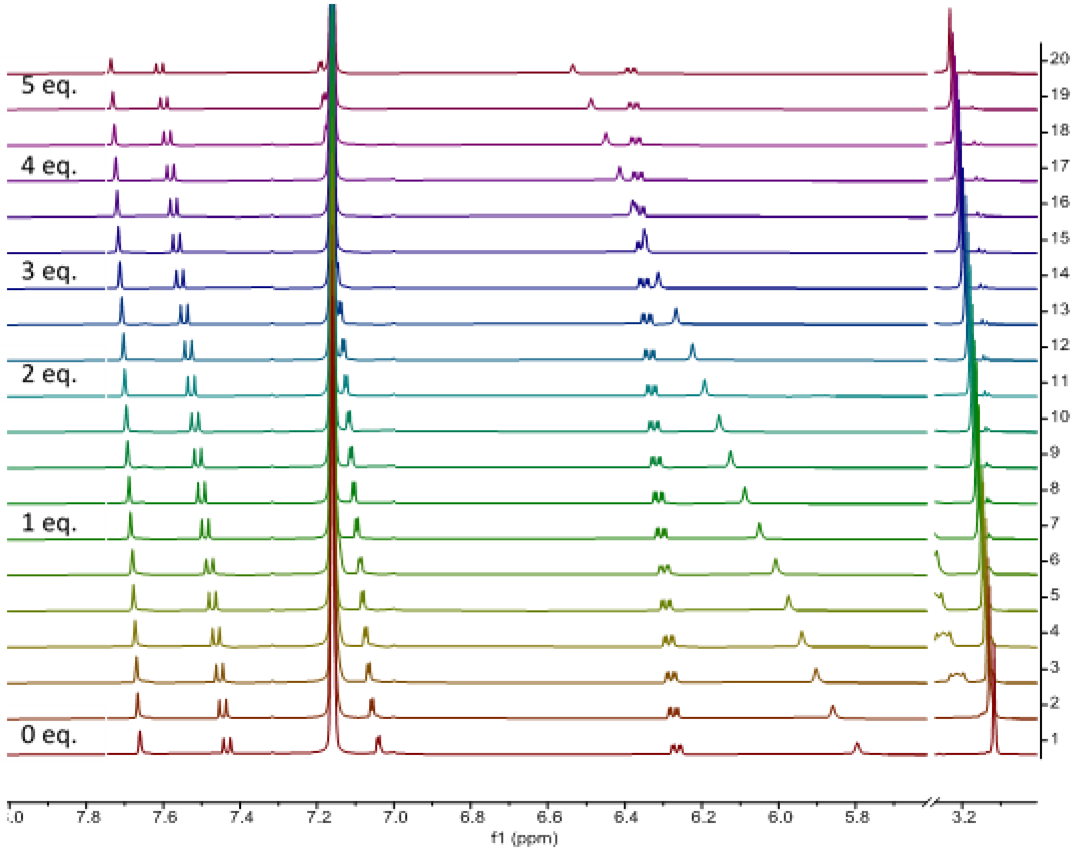

Figure S81-2. ^{1}H NMR spectra of **2MeO-G3XB** upon addition of increasing equivalents of THA·I (C_6D_6, 298 K, 500 MHz).

Assay 3 K_a= 63.2 M^{-1} http://app.supramolecular.org/bindfit/view/c15738b4-3ebf-430b-95e8-2cb0a3388a03

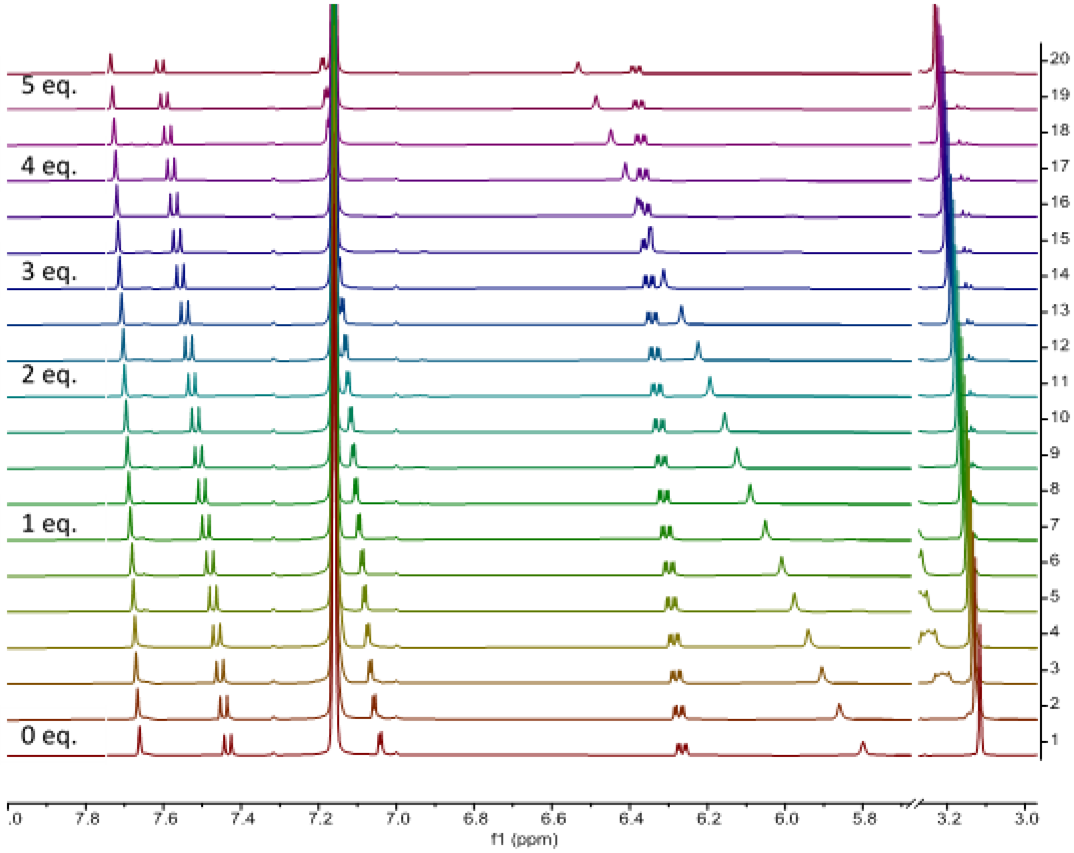

Figure S81-3. ^{1}H NMR spectra of **2MeO-G3XB** upon addition of increasing equivalents of THA·I (C_6D_6, 298 K, 500 MHz).

Cl-G3XB with THA iodide

Assay 1 K_a=330.0 M^{-1} http://app.supramolecular.org/bindfit/view/75f56ee4-d8a9-4703-9cae-0c7493e2ca0a

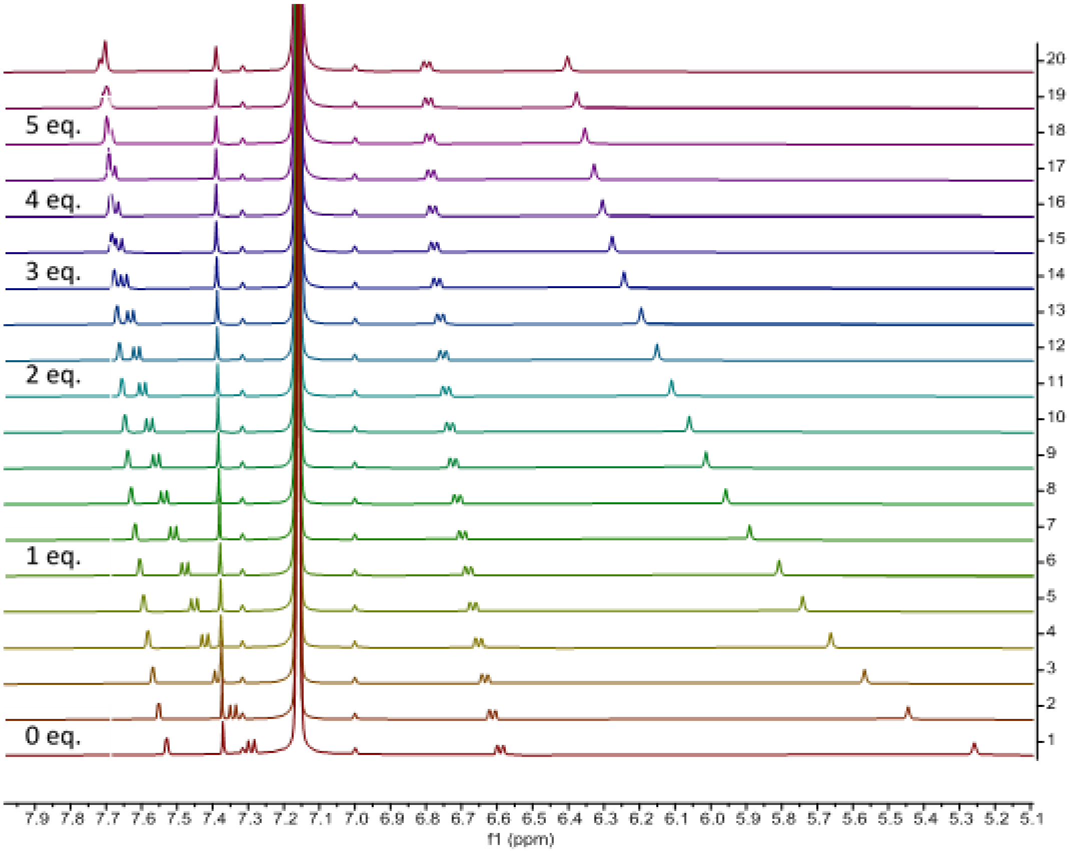

Figure S82-1. ^{1}H NMR spectra of **Cl-G3XB** upon addition of increasing equivalents of THA·I (C_6D_6, 298 K, 500 MHz).

Assay 2 K_a=317.7 M^{-1} http://app.supramolecular.org/bindfit/view/f0c61398-8b18-4b85-89cf-594c0491549e

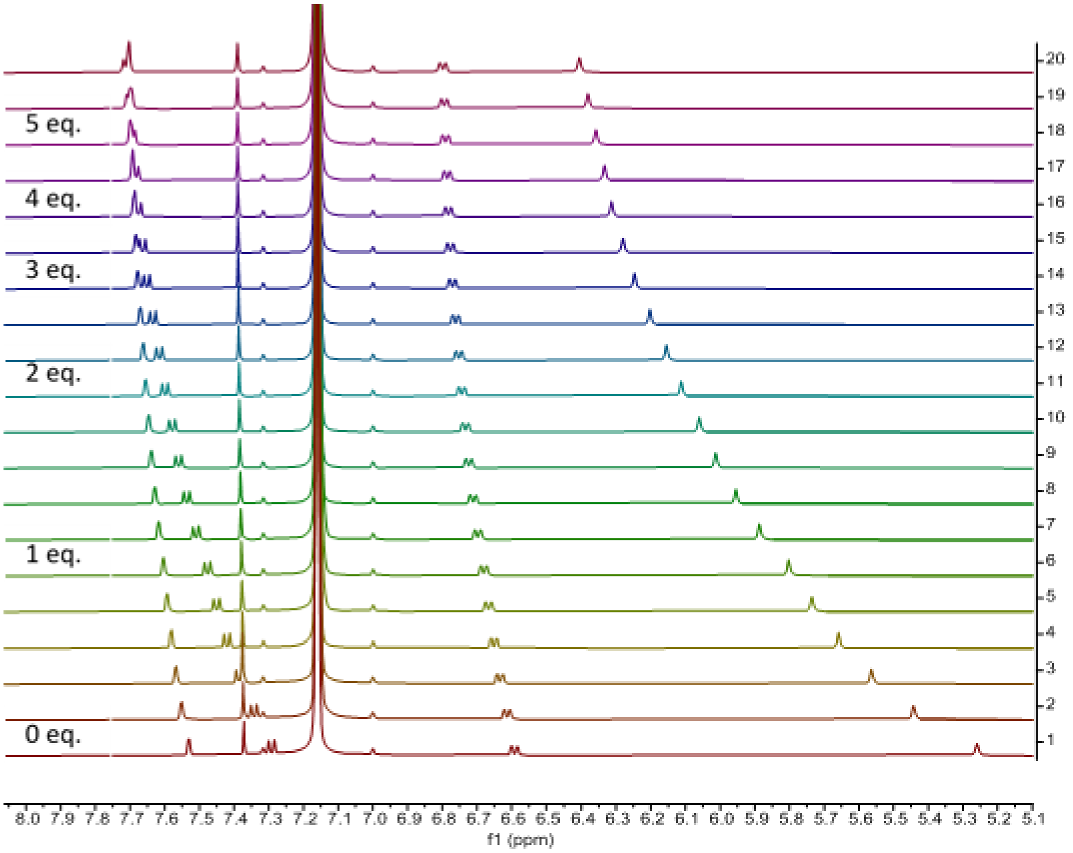

Figure S82-2. ^{1}H NMR spectra of **Cl-G3XB** upon addition of increasing equivalents of THA·I (C_6D_6, 298 K, 500 MHz).

Assay 3 K_a= 345.4 M^{-1} http://app.supramolecular.org/bindfit/view/1cb0201b-3798-4796-9753-9e3bc55ccd83

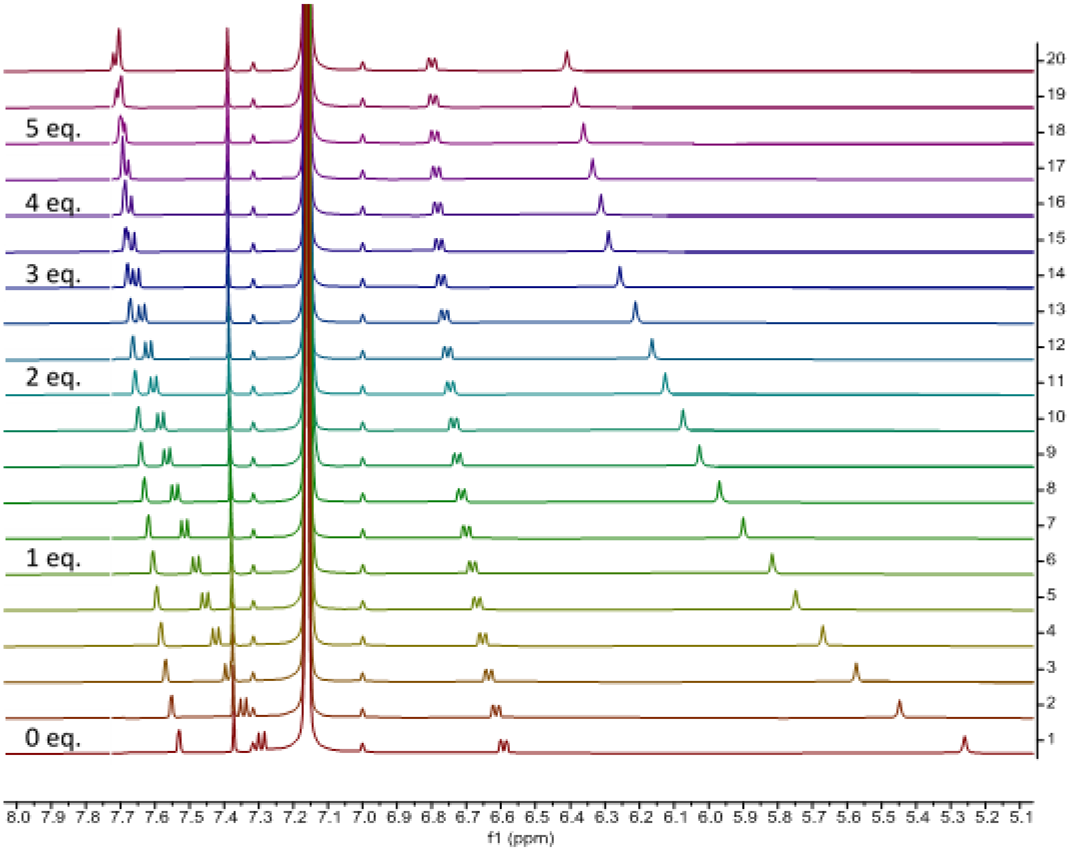

Figure S82-3. ^{1}H NMR spectra of **Cl-G3XB** upon addition of increasing equivalents of THA·I (C_6D_6, 298 K, 500 MHz).

F-G3XB with THA iodide

Assay 1 K_a= 236.4 M^{-1} http://app.supramolecular.org/bindfit/view/7b565776-3cc2-4956-ae6e-249da1dc7ee6

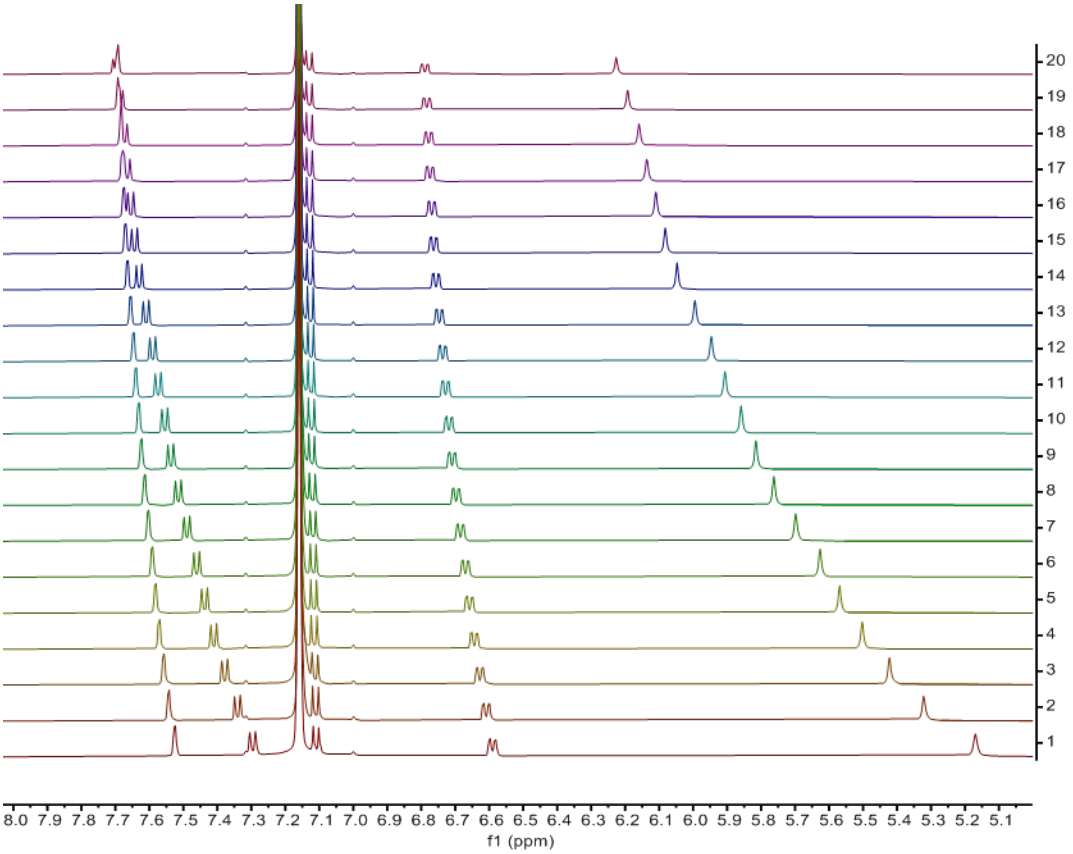

Figure S83-1. ^{1}H NMR spectra of **F-G3XB** upon addition of increasing equivalents of THA·I (C_6D_6, 298 K, 500 MHz).

Assay 2 K_a= 274.4 M^{-1} http://app.supramolecular.org/bindfit/view/a5ed5e27-8f4b-43a7-975b-1f0fb3b071b8

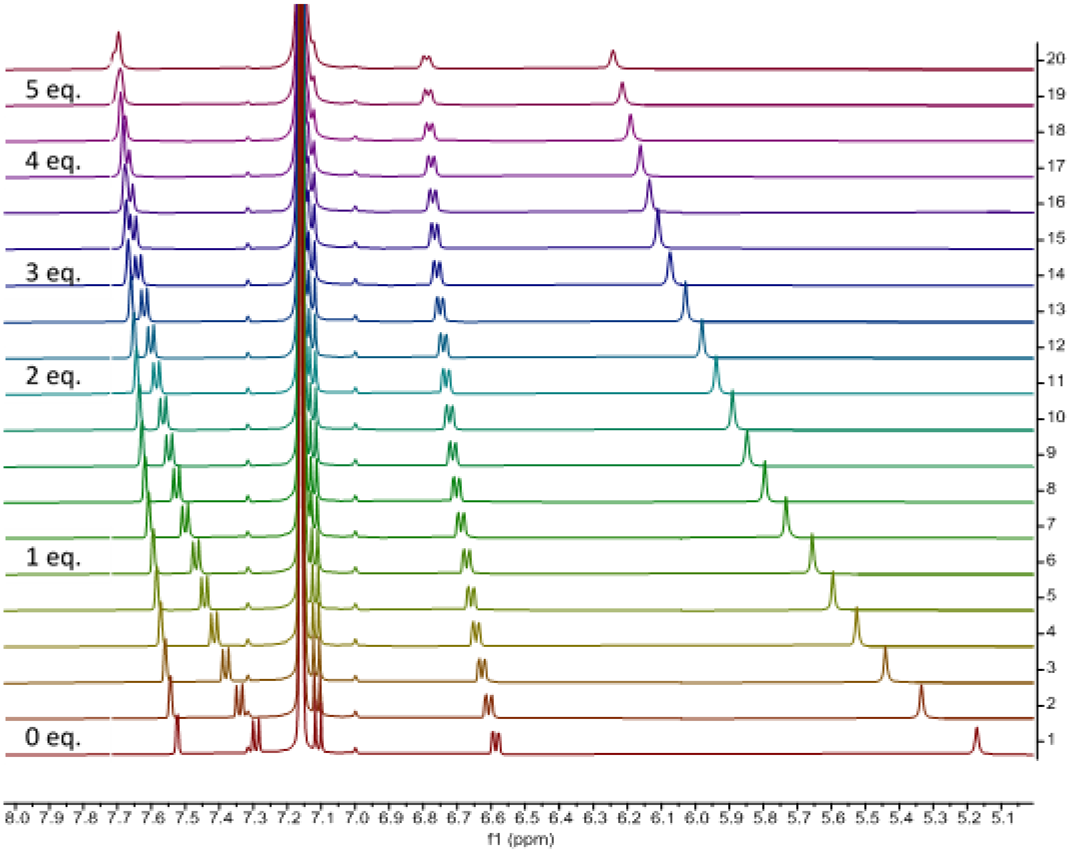

Figure S83-2. ^{1}H NMR spectra of **F-G3XB** upon addition of increasing equivalents of THA·I (C_6D_6, 298 K, 500 MHz).

Assay 3 K_a= 241.1 M^{-1} http://app.supramolecular.org/bindfit/view/ed534960-85cd-4a81-bb80-9e35b275c9d0

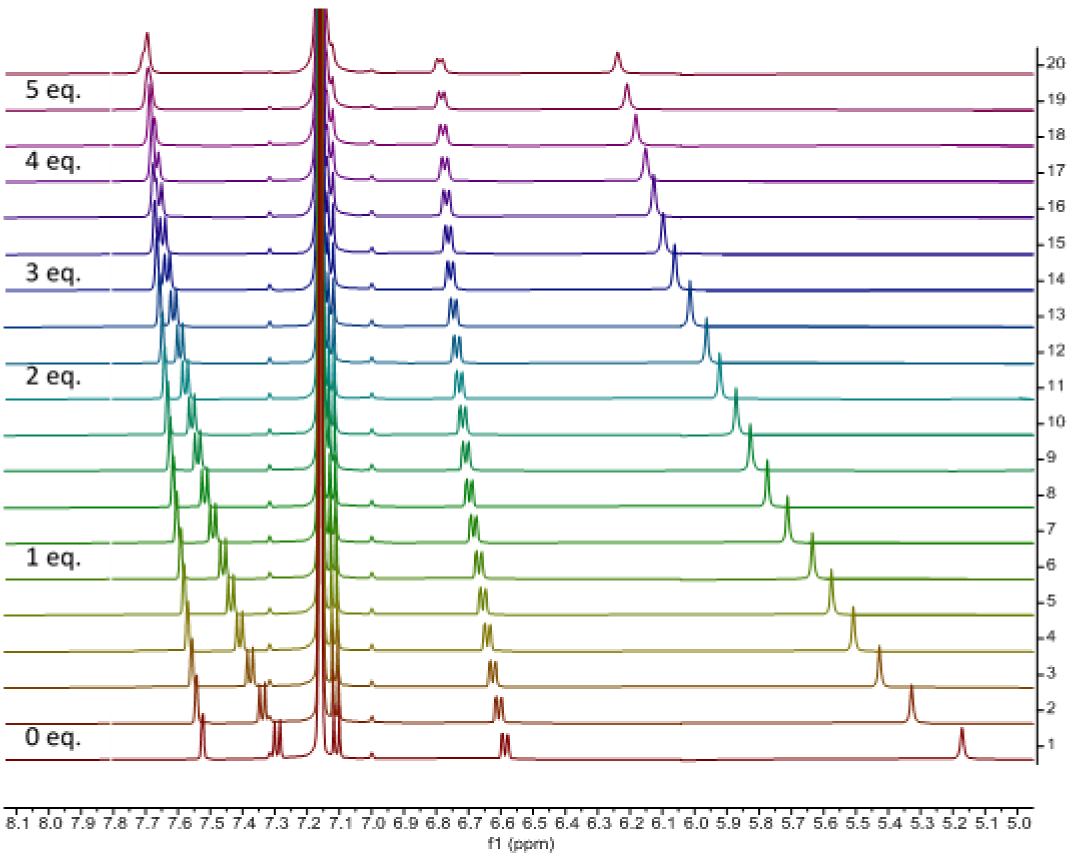

Figure S83-3. ^{1}H NMR spectra of **F-G3XB** upon addition of increasing equivalents of THA·I (C_6D_6, 298 K, 500 MHz).

H-G3XB with THA iodide

Assay 1 K_a= 194.6 M^{-1} http://app.supramolecular.org/bindfit/view/17befb01-d3f6-4d27-bf34-4c9c67c9ca8c

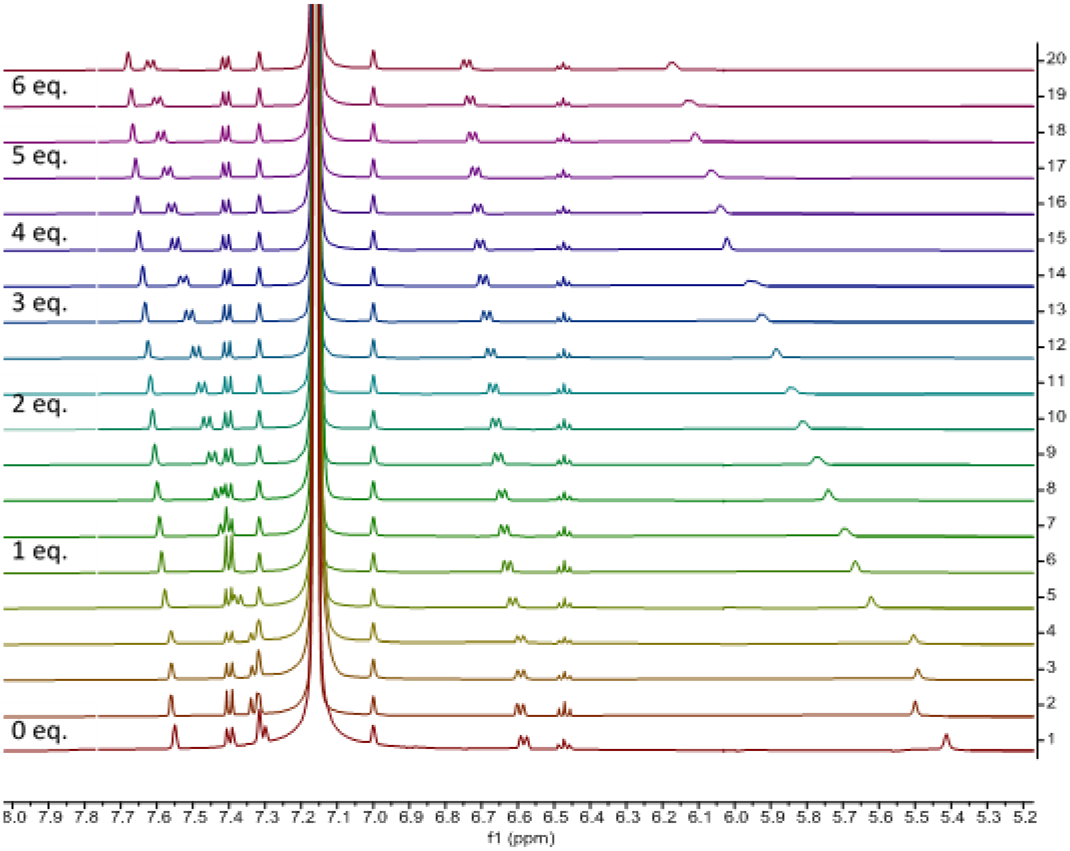

Figure S84-1. ^{1}H NMR spectra of **H-G3XB** upon addition of increasing equivalents of THA·I (C_6D_6, 298 K, 500 MHz).

Assay 2 K_a= 199.1 M^{-1} http://app.supramolecular.org/bindfit/view/8c81c119-d5b1-408a-a314-c9c82af2eac8

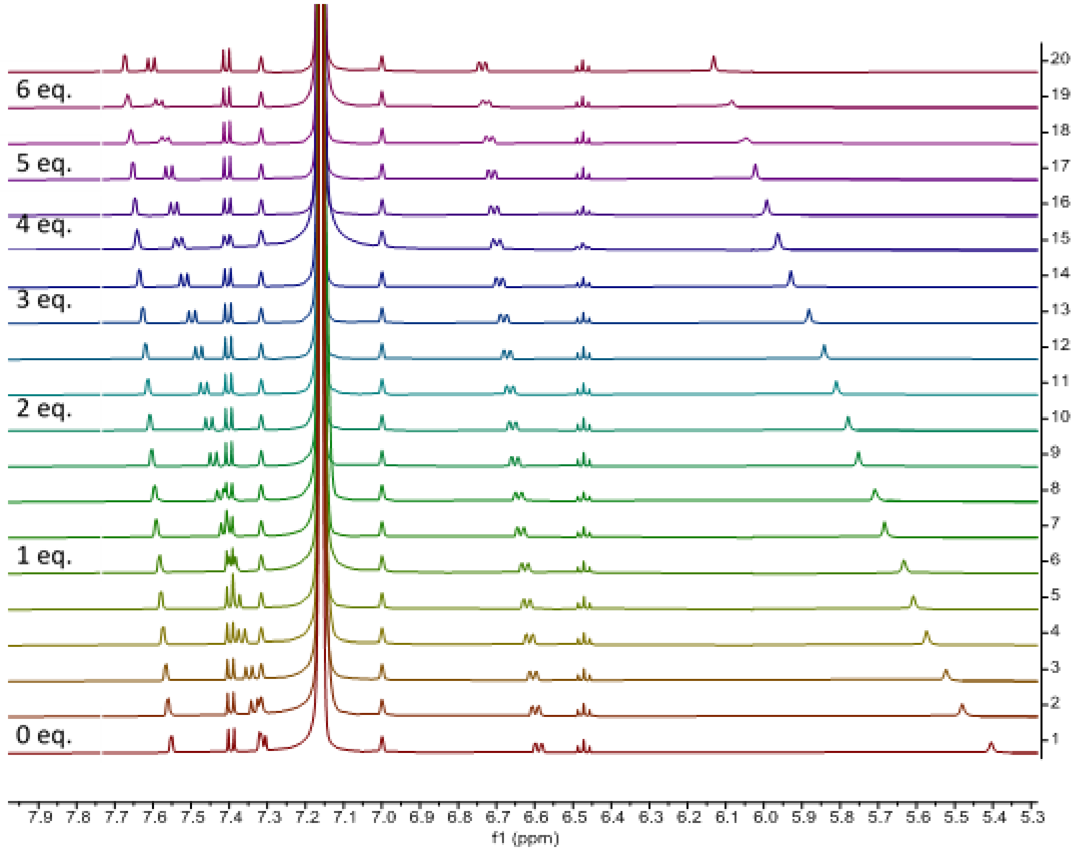

Figure S84-2. ^{1}H NMR spectra of **H-G3XB** upon addition of increasing equivalents of THA·I (C_6D_6, 298 K, 500 MHz).

Assay 3 K_a= 188.8 M^{-1} http://app.supramolecular.org/bindfit/view/f9dda397-b6ad-4f30-9b80-56fea76e0b2c

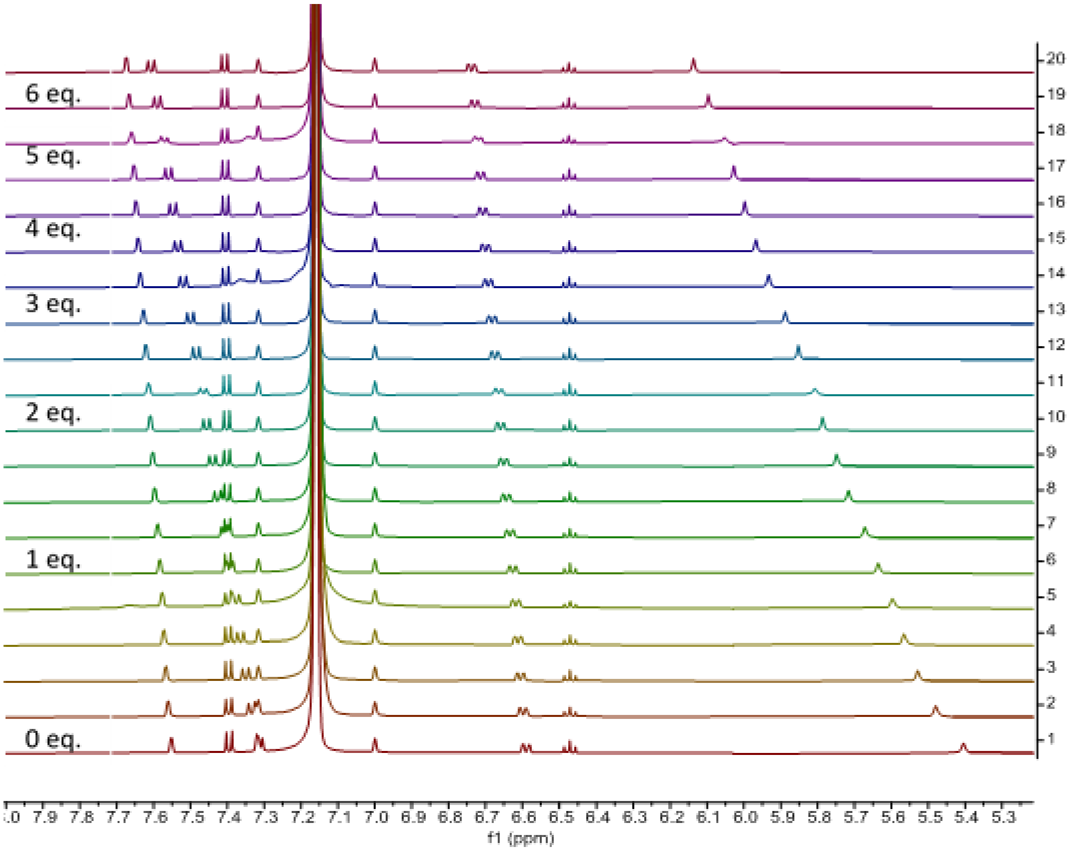

Figure S84-3. ^{1}H NMR spectra of **H-G3XB** upon addition of increasing equivalents of THA·I (C_6D_6, 298 K, 500 MHz).

Me-G3XB with THA iodide

Assay 1 K_a= 182.0 M^{-1} http://app.supramolecular.org/bindfit/view/c2346362-48e8-41e3-90c1-53aae0eb5eeb

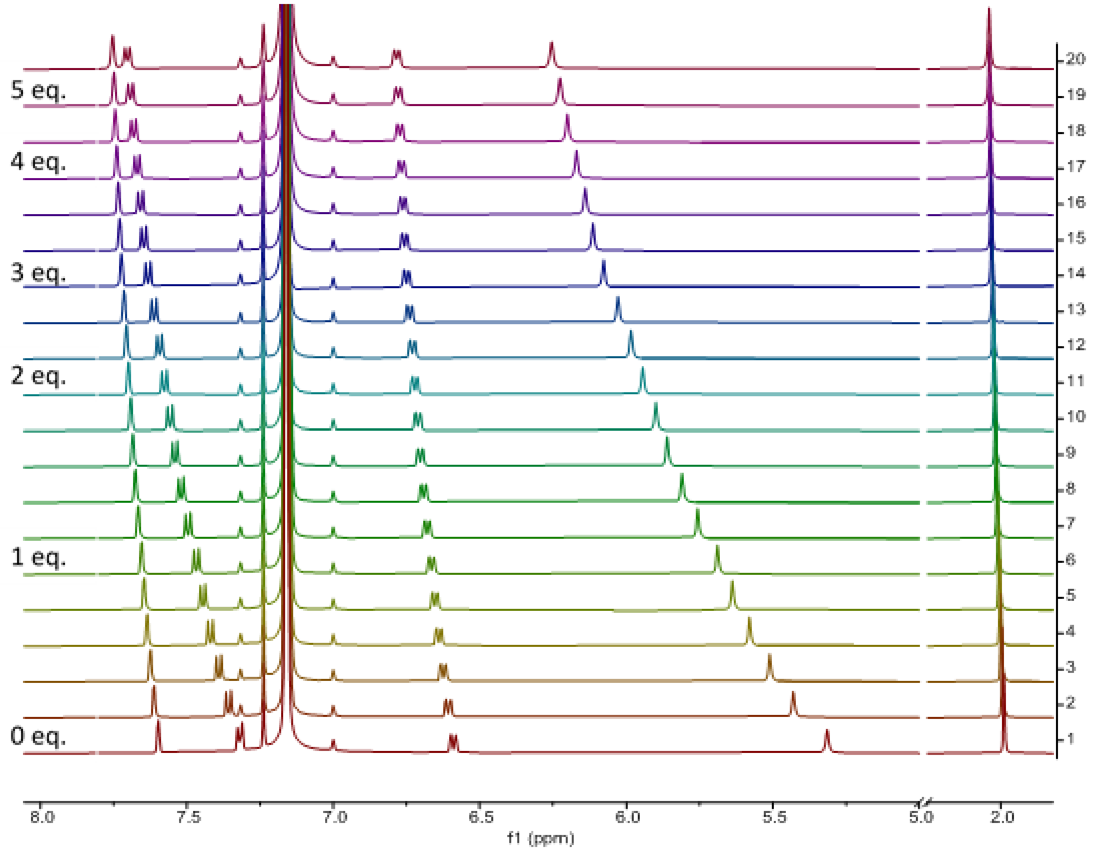

Figure S85-1. ^{1}H NMR spectra of **Me-G3XB** upon addition of increasing equivalents of THA·I (C_6D_6, 298 K, 500 MHz).

Assay 2 K_a= 171.7 M^{-1} http://app.supramolecular.org/bindfit/view/d3632f46-0eea-411e-9b0f-4a90ccbbbed8

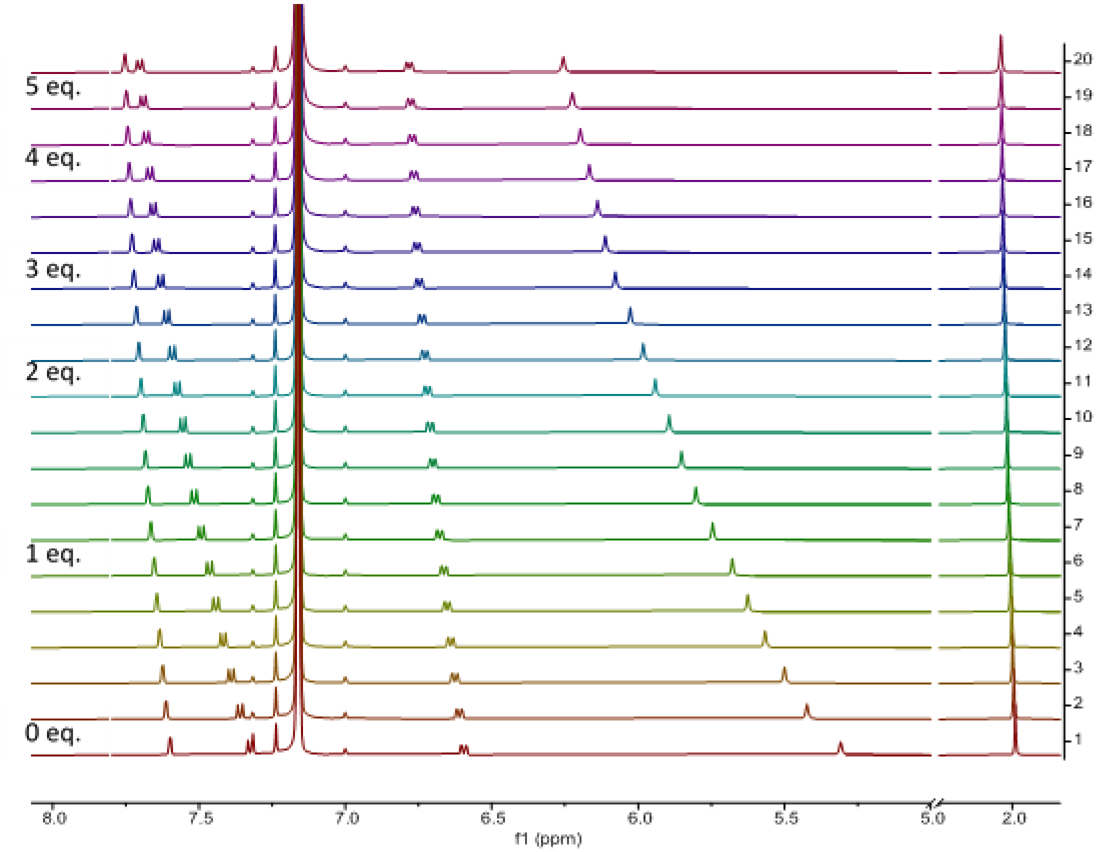

Figure S85-2. ^{1}H NMR spectra of **Me-G3XB** upon addition of increasing equivalents of THA·I (C_6D_6, 298 K, 500 MHz).

Assay 3 K_a= 170.2 M^{-1} http://app.supramolecular.org/bindfit/view/74be5d95-2df8-47cf-905a-4cda87ec7d65

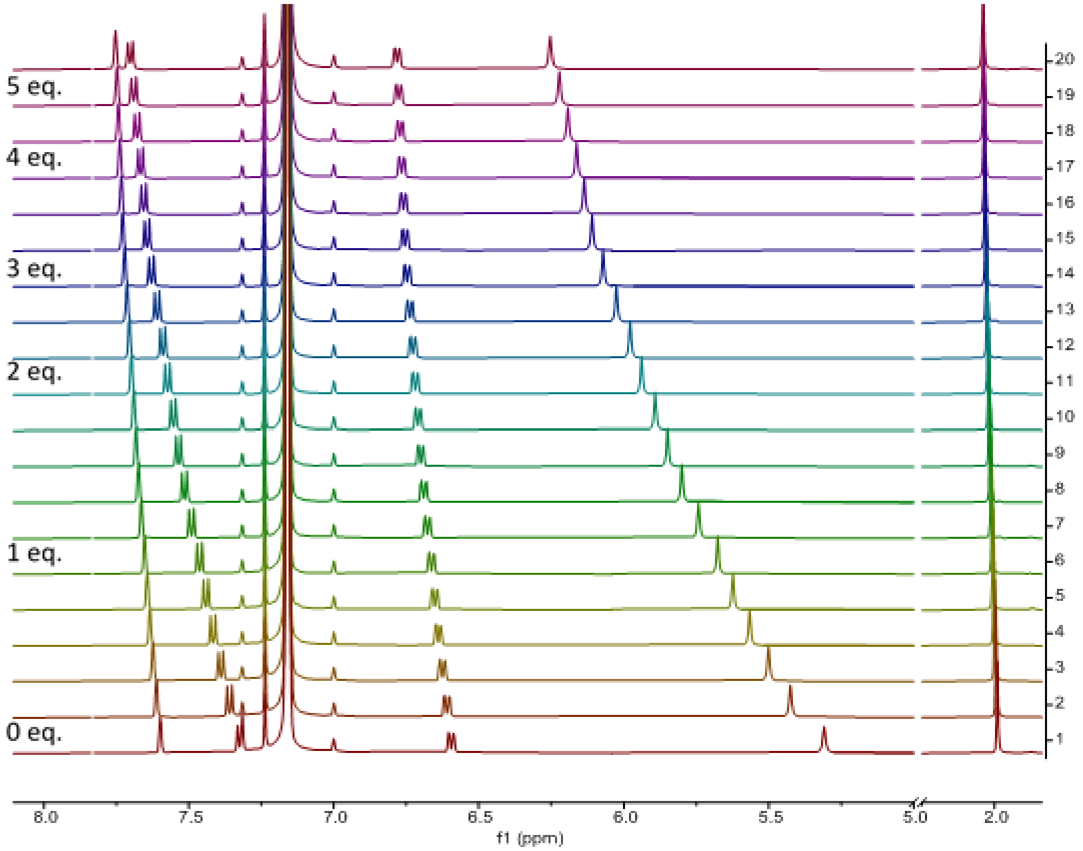

Figure S85-3. ^{1}H NMR spectra of **Me-G3XB** upon addition of increasing equivalents of THA·I (C_6D_6, 298 K, 500 MHz).

nHBeXB with THA iodide

Assay 1 K_a= 12.4 M^{-1} http://app.supramolecular.org/bindfit/view/7e08d321-604f-45b4-869d-2dd0451a9fea

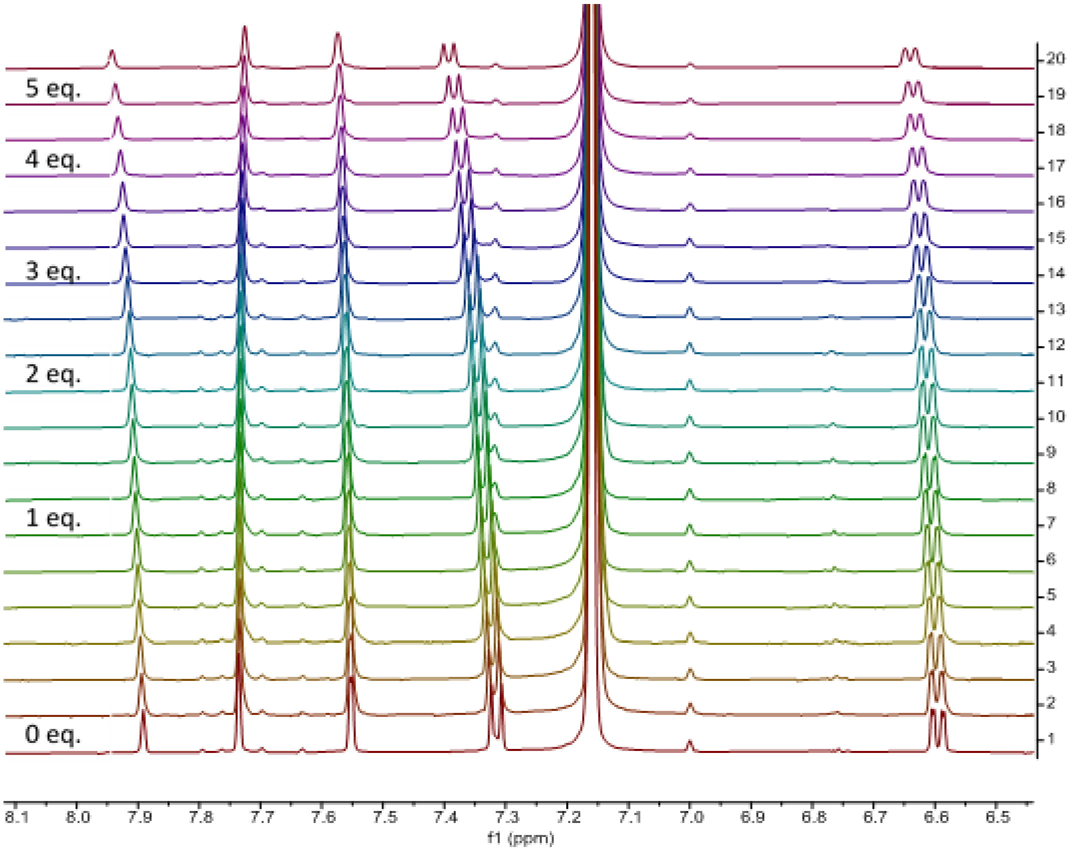

Figure S86-1. ^{1}H NMR spectra of **nHBeXB** upon addition of increasing equivalents of THA·I (C_6D_6, 298 K, 500 MHz).

Assay 2 K_a= 4.4 M^{-1} http://app.supramolecular.org/bindfit/view/29d125a6-7c77-4292-83bb-9e8b03dbcd1b

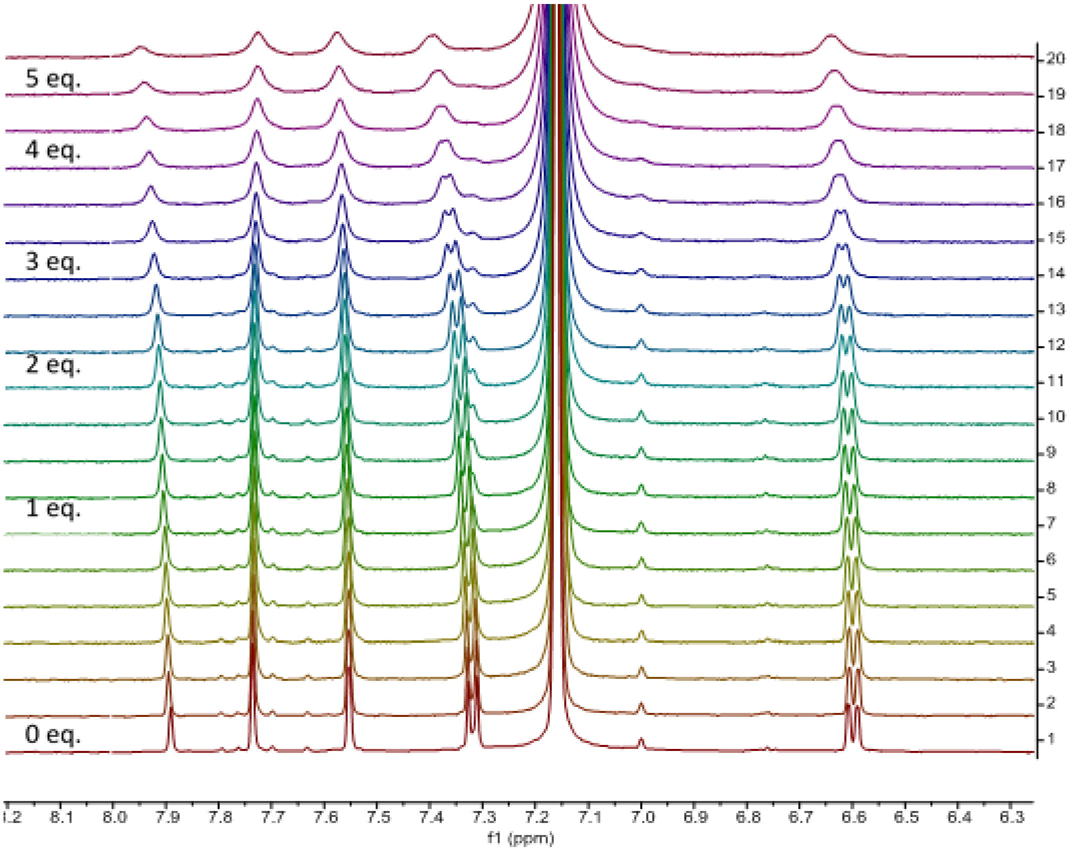

Figure S86-2. ^{1}H NMR spectra of **nHBeXB** upon addition of increasing equivalents of THA·I (C_6D_6, 298 K, 500 MHz).

Assay 3 K_a= 8.0 M^{-1} http://app.supramolecular.org/bindfit/view/da8fec06-3b2f-4e26-8bd9-0b9467705ec2

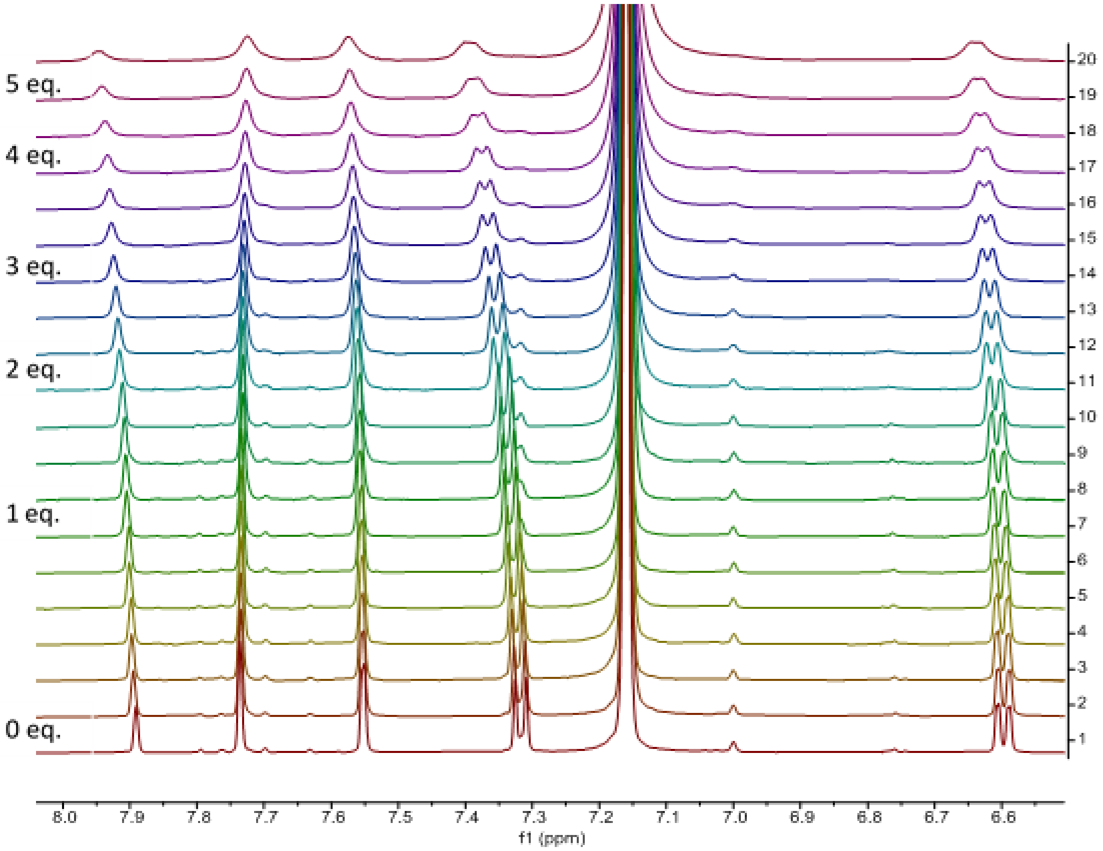

Figure S86-3. ^{1}H NMR spectra of **nHBeXB** upon addition of increasing equivalents of THA·I (C_6D_6, 298 K, 500 MHz).

G3HB with THA iodide

Assay 1 K_a= 36.8 M^{-1} http://app.supramolecular.org/bindfit/view/6847ebbe-5e75-417c-a18f-3b9e06f6c3fe

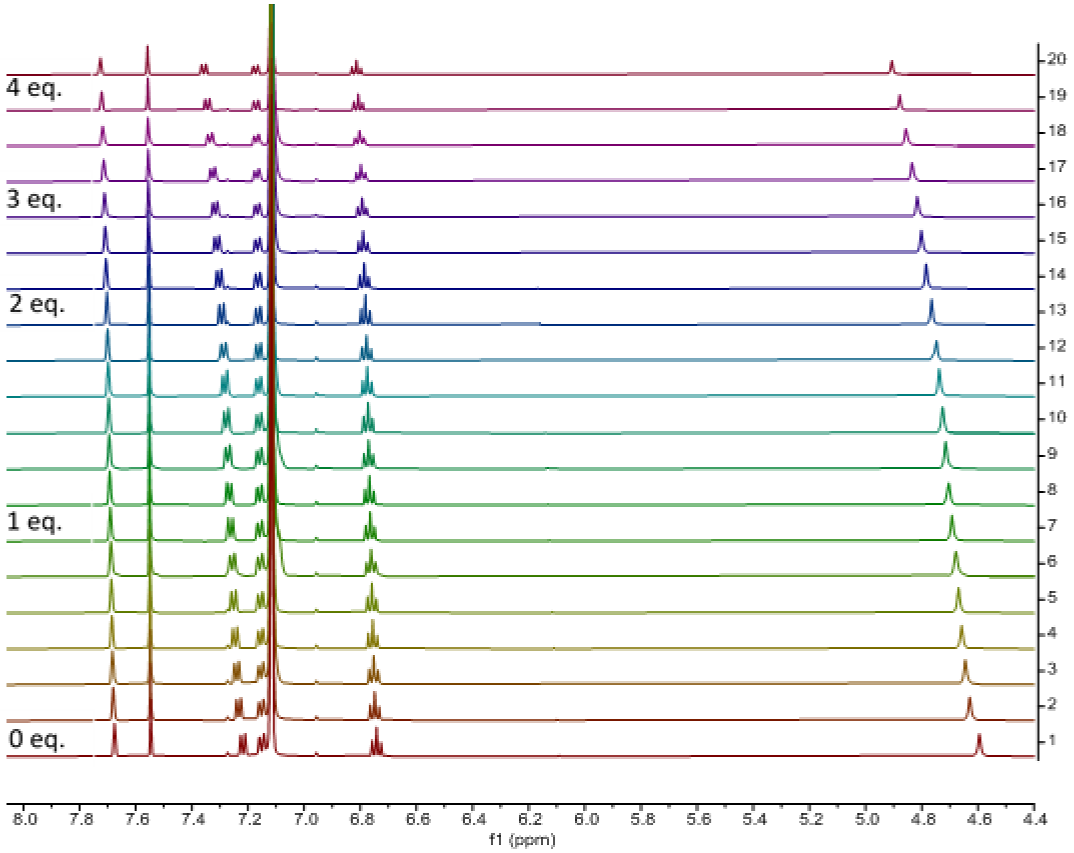

Figure S87-1. ^1H NMR spectra of **G3HB** upon addition of increasing equivalents of THA·I (C_6D_6, 298 K, 500 MHz).

Assay 2 K_a= 22.5 M^{-1} http://app.supramolecular.org/bindfit/view/29553f62-9021-445b-85b1-32cd17529328

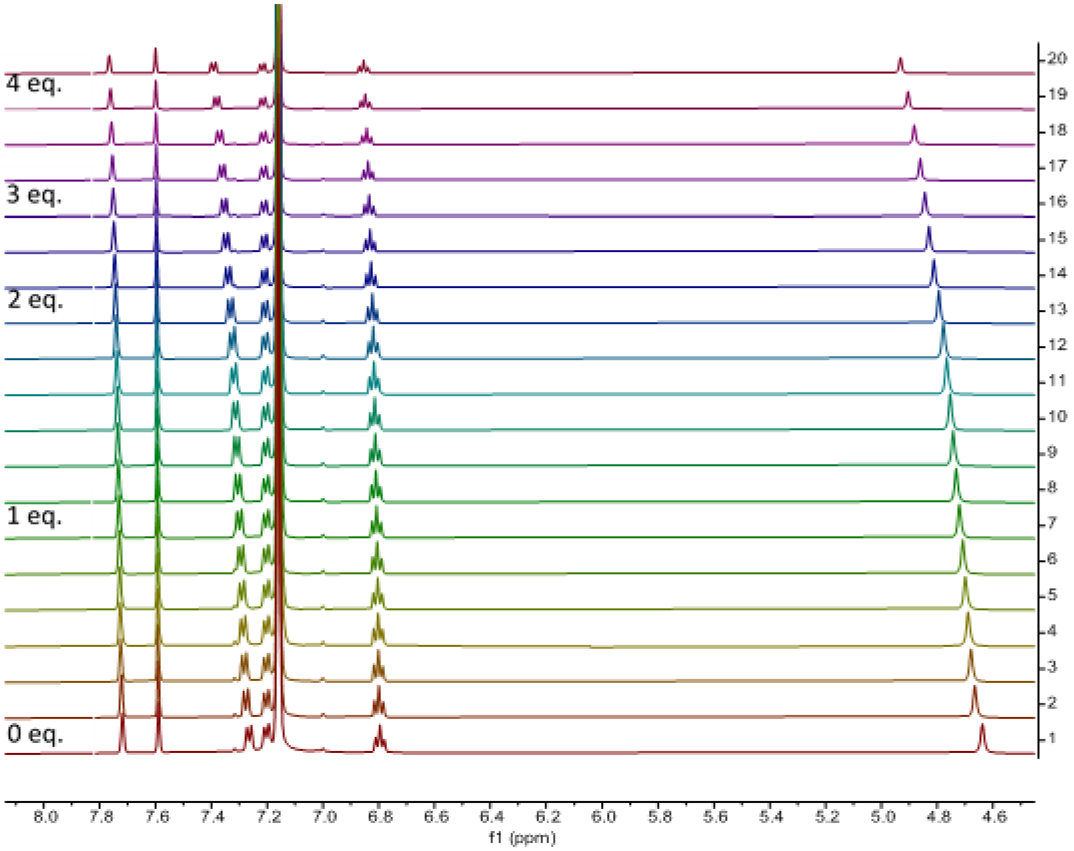

Figure S87-2. ^{1}H NMR spectra of **G3HB** upon addition of increasing equivalents of THA·I (C_6D_6, 298 K, 500 MHz).

Assay 3 K_a= 23.2 M^{-1} http://app.supramolecular.org/bindfit/view/ff9ef97c-9dea-47c3-865d-9a8fab9e4b50

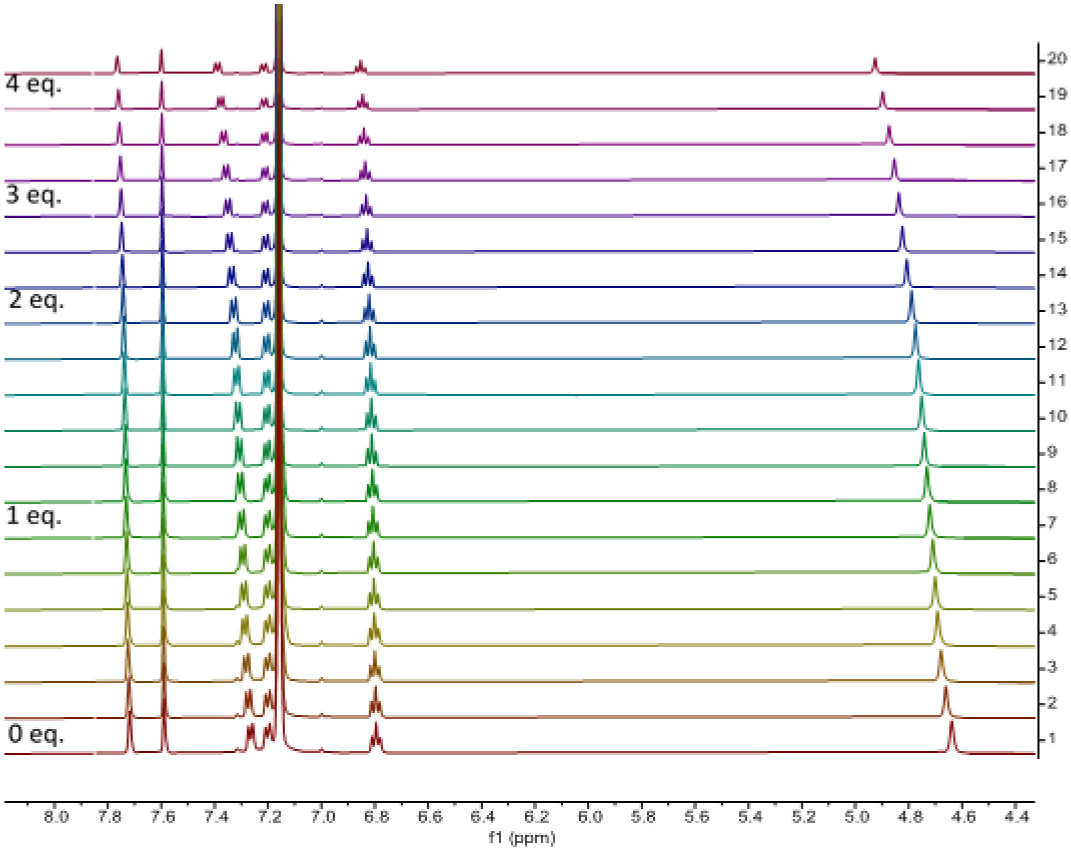

Figure S87-3. ^{1}H NMR spectra of **G3HB** upon addition of increasing equivalents of THA·I (C_6D_6, 298 K, 500 MHz).

Linear free energy relationships

Besides Hammett parameters, we also plotted the association constants obtained from NMR titrations with other parameters including Taft's σ_I, σ_R[6] and Dobrowolski's sEDA, pEDA[7].

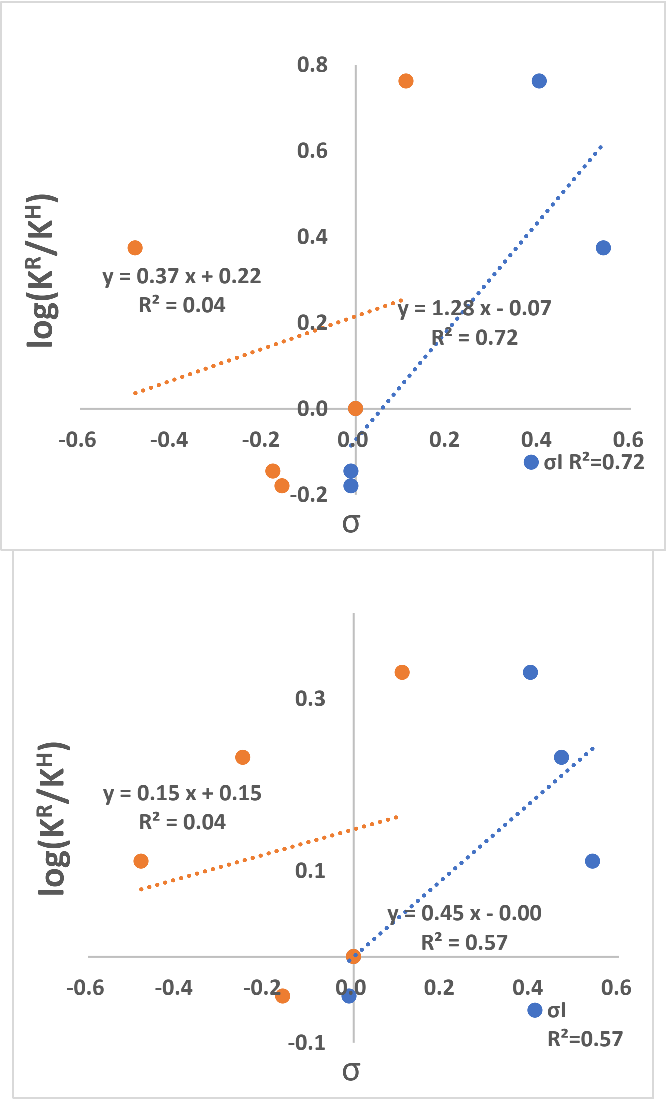

Figure S88. Normalized Hammett plots of K values of **2R₂-G3XB** (Left) and **R₁-G3XB** (Right) with σI (Blue) and σR (Orange).

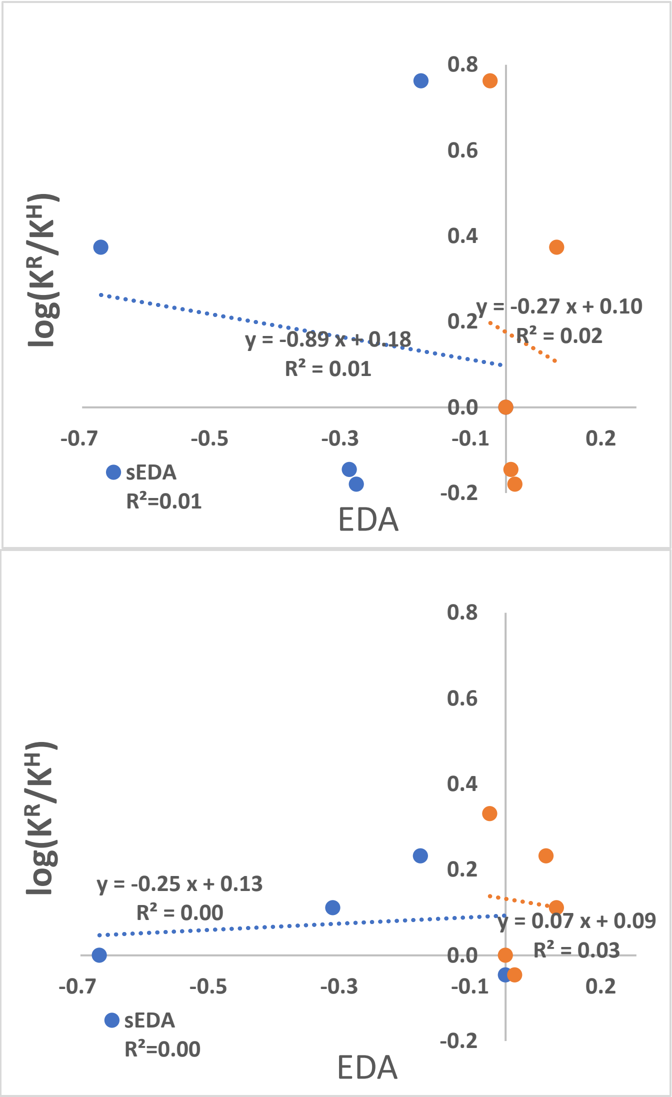

Figure S89. Normalized Hammett plots of K values of **$2R_2$-G3XB** (Left) and **R_1-G3XB** (Right) with sEDA (Blue) and pEDA (Orange).

Computaions

Calculations were carried out with the Gaussian 16 suite of programs using M062X functional employing the def2TZVPP basis set for all atoms except iodine. For iodine LANL2DZdp and effective core potential (ECP) were used. The LANL2DZdp ECP basis set was downloaded from the EMSL Basis Set Exchange (https://bse.pnl.gov/bse/portal).

Table S3-1. **Computation results summary**

	$V_{s,max}$ (kcal/mol)	$V_{s,min}$ (kcal/mol)	SPE (kcal/mol)		$V_{s,max}$(HBed) (kcal/mol)	$V_{s,max}$(non-HBed) (kcal/mol)	SPE (kcal/mol)	$\Delta V_{s,max}$ (kcal/mol)		$V_{s,max}$ (kcal/mol)	SPE (kcal/mol)
**G3XB**	32.05	-1.11	-1573352.23	**G3XB-S**	31.45	24.46	-1573350.70	6.99	**G3XB-W**	24.64	-1573349.12
**2F-G3XB**	28.40	-4.14	-1274864.51	**2F-G3XB-S**	28.52	20.94	-1274862.74	7.58			
**2H-G3XB**	24.98	-7.22	-1150303.91	**2H-G3XB-S**	26.05	17.95	-1150302.08	8.10			
**2Me-G3XB**	23.03	-9.43	-1199640.14	**2Me-G3XB-S**	24.69	16.94	-1199638.23	7.75			
**2MeO-G3XB**	22.96	-8.87	-1294035.26	**2MeO-G3XB-S**	23.25	15.73	-1294032.74	7.52			
**2*t*Bu-G3XB**	22.41	-11.14	-1347636.51	**2*t*Bu-G3XB-S**	24.37	16.15	-1347634.36	8.18			
**Cl-G3XB**	30.84		-1650226.19	**Cl-G3XB-S**	29.85	24.16	-1650224.76	5.69			
**F-G3XB**	30.12		-1424105.19	**F-G3XB-S**	29.33	24.61	-1424103.85	4.72			
**H-G3XB**	28.72		-1361826.55	**H-G3XB-S**	28.08	24.07	-1361825.11	4.01			
**Me-G3XB**	28.11		-1386493.72	**Me-G3XB-S**	27.36	24.04	-1386492.36	3.32			
**G3HB**											
**nHBeXB**	28.03		-1538603.60	**nHBeXB-S**	26.64	25.39	-1538603.61				

Table S3-2. **Computation results summary continued**

Dihedral Freeze	$V_{s,max}$(HBed) (kcal/mol)	$V_{s,max}$(non-HBed) (kcal/mol)	$\Delta V_{s,max}$ (kcal/mol)
**G3XB-S**	31.62	24.98	6.65
**2F-G3XB-S**	28.67	20.85	7.82
**2H-G3XB-S**	26.17	18.28	7.89
**2Me-G3XB-S**	24.99	16.46	8.53
**2MeO-G3XB-S**	23.31	15.73	7.58
**2*t*Bu-G3XB-S**	24.67	16.06	8.61

	Vs,max	Vs,min	SPE		Vs,max(Hbed)	Vs,max(non-HBed)	SPE		
G3XB	32.05	-1.11	-1573352.23	G3XB-S	31.45	24.46	-1573350.70	G3XB-W	24.64
2F-G3XB	28.40	-4.14	-1274864.51	2F-G3XB-S	28.52	20.94	-1274862.74		

2H-G3XB	24.98	-7.22	-1150303.91	2H-G3XB-S	26.05	17.95	-1150302.08			
2Me-G3XB	23.03	-9.43	-1199640.14	2Me-G3XB-S	24.69	16.94	-1199638.23			
2MeO-G3XB	22.96	-8.87	-1294035.26	2MeO-G3XB-S	23.25	15.73	-1294032.74			
2tBu-G3XB	22.41	-11.14	-1347636.51	2tBu-G3XB-S	24.37	16.15	-1347634.36			
Cl-G3XB	30.84		-1650226.19	Cl-G3XB-S	29.85	24.16	-1650224.76			
F-G3XB	30.12		-1424105.19	F-G3XB-S	29.33	24.61	-1424103.85			
H-G3XB	28.72		-1361826.55	H-G3XB-S	28.08	24.07	-1361825.11			
Me-G3XB	28.11		-1386493.72	Me-G3XB-S	27.36	24.04	-1386492.36			
G3HB			-1200577.22							
nHBeXB	28.03		-1538603.60	nHBeXB-S	26.64	25.39	-1538603.61			

Dihedral freeze			DVs,max
G3XB-S	31.62	24.98	6.65
2F-G3XB-S	28.67	20.85	7.82
2H-G3XB-S	26.17	18.28	7.89
2Me-G3XB-S	24.99	16.46	8.53
2MeO-G3XB-S	23.31	15.73	7.58
2tBu-G3XB-S	24.67	16.06	8.61

G3XB bidentate

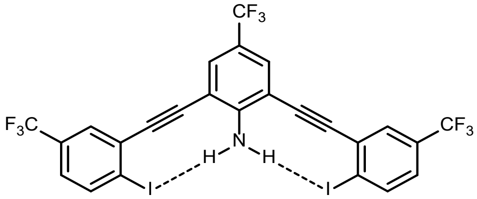

Coordinates for **G3XB bidentate**:

0 1

I	2.9778167	-2.7396896	-0.3462496
C	0.0111348	5.4783418	0.0113884
N	-0.0015477	-0.1739476	0.0079975
H	0.8673365	-0.6824503	-0.0002460
H	-0.8707141	-0.6820348	0.0141493
C	7.1194457	-1.5729951	-0.0511001
C	4.6677710	-0.2193214	-0.0446361
C	4.7227050	-1.6123268	-0.1631284
C	3.4442640	0.5082846	-0.0371995
C	1.2154129	1.8974180	-0.0178372
C	2.4390423	1.1733689	-0.0288475
C	5.9388684	-2.2829825	-0.1661045
H	5.9607287	-3.3588905	-0.2585033
C	-0.0013717	1.1717394	-0.0009224
C	-2.4408280	1.1761420	0.0156468
C	1.1989788	3.2861319	-0.0272539
H	2.1385803	3.8209328	-0.0446999
C	-1.2164467	1.8986306	0.0030879
C	-3.4466567	0.5119105	0.0269671
C	-0.0003083	3.9813980	-0.0247885
C	5.8756692	0.4830954	0.0708923
H	5.8469550	1.5588241	0.1636559
C	7.0796411	-0.1883958	0.0670550
C	-1.1993217	3.2885593	-0.0086554
H	-2.1383377	3.8237698	-0.0105614
C	-7.1234836	-1.5665715	0.0470892
C	-4.6706862	-0.2146754	0.0368664
C	-7.0823230	-0.1828566	-0.0807222
C	-5.8778614	0.4877231	-0.0864855
C	-4.7270483	-1.6067718	0.1652057

C	-5.9436998	-2.2765372	0.1699948
H	-5.9665497	-3.3517460	0.2700410
I	-2.9834987	-2.7341139	0.3608386
H	-5.8481493	1.5627551	-0.1867250
F	-1.1200125	6.0004797	-0.4758799
F	1.0265030	5.9863056	-0.6986026
F	0.1470914	5.9512746	1.2605097
C	8.3797316	0.5576216	0.1893799
F	9.0773888	0.1473314	1.2558848
F	8.2008776	1.8736697	0.3106732
F	9.1590296	0.3556154	-0.8804797
C	-8.3815398	0.5632183	-0.2115550
F	-9.1653657	0.3660315	0.8558880
F	-9.0750252	0.1488289	-1.2792251
F	-8.2015954	1.8786786	-0.3376366
H	-8.0729711	-2.0848137	0.0502089
H	8.0685407	-2.0919666	-0.0527297

G3XB S conformation

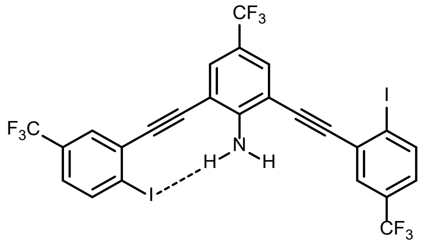

Coordinates for **G3XB-S**:

0 1

I	5.8844208	2.0037635	-0.5392262
C	-0.0025468	5.1285307	0.3759551
N	-0.2235464	-0.5121546	0.0530508
H	0.6267284	-1.0358890	-0.0541402
H	-1.1073377	-0.9827150	-0.0545641
C	6.8446987	-2.1653467	0.0392177
C	4.4999818	-0.6388215	0.0831043
C	5.7405074	-0.0419894	-0.1748174
C	3.2878308	0.1085954	0.1105293
C	1.0678897	1.5134913	0.1633647
C	2.2728584	0.7575554	0.1357951
C	6.9022619	-0.8037983	-0.1948461
H	7.8513904	-0.3291387	-0.3961245
C	-0.1732938	0.8345333	0.1141673
C	-2.6096540	0.9244354	0.0797011
C	1.1013429	2.8999388	0.2324677
H	2.0613276	3.3975793	0.2625916
C	-1.3610290	1.6019768	0.1394670

C	-3.6366992	0.2958869	0.0243188
C	-0.0729893	3.6372025	0.2567611
C	4.4611658	-2.0186659	0.3211809
H	3.5110193	-2.4890095	0.5280224
C	5.6187313	-2.7675189	0.2968443
C	-1.2953604	2.9894890	0.2128494
H	-2.2147987	3.5571948	0.2266763
C	-7.3803795	-1.6514901	-0.1501949
C	-4.8841405	-0.3871513	-0.0373253
C	-7.2956429	-0.2724157	0.0037775
C	-6.0695248	0.3552330	0.0599181
C	-4.9842454	-1.7741656	-0.1918396
C	-6.2226152	-2.4005317	-0.2478627
H	-6.2796082	-3.4725296	-0.3675508
I	-3.2755106	-2.9595048	-0.3452188
H	-6.0058239	1.4267480	0.1799622
F	-1.1350405	5.7184721	-0.0214132
F	0.9949368	5.6395134	-0.3574748
F	0.2164197	5.5225369	1.6402318
C	5.5846423	-4.2480977	0.5545520
F	6.1107013	-4.9340413	-0.4677333
F	4.3430003	-4.7020505	0.7419466
F	6.2961695	-4.5720520	1.6412130
C	-8.5719170	0.5161626	0.1092875
F	-9.3032817	0.1192128	1.1581073
F	-9.3368225	0.3513998	-0.9771048
F	-8.3509605	1.8240859	0.2480959
H	-8.3466211	-2.1359236	-0.1932057
H	7.7489691	-2.7588730	0.0215081

G3XB W conformation

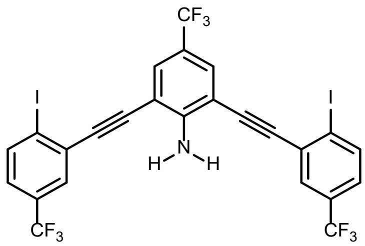

Coordinates for **G3XB-W**:

0 1

I	6.0338196	-1.9098178	-0.3151856
C	-0.0000747	-4.7563945	0.5001288
N	-0.0000173	0.8796073	-0.0021362
H	0.8626034	1.3512258	-0.2112487
H	-0.8626316	1.3512086	-0.2113135
C	7.1234483	2.2619556	-0.1139124
C	4.7237799	0.8261936	-0.0152305
C	5.9499326	0.1657121	-0.1626960

C	3.4847547	0.1259633	0.0388682
C	1.2142877	-1.1919612	0.1538317
C	2.4461322	-0.4826213	0.0909674
C	7.1390129	0.8827485	-0.2102806
H	8.0766035	0.3587948	-0.3237636
C	-0.0000015	-0.4720600	0.0921002
C	-2.4461363	-0.4826292	0.0909844
C	1.1993217	-2.5773791	0.2697405
H	2.1407764	-3.1077903	0.3089121
C	-1.2142948	-1.1919793	0.1538481
C	-3.4847510	0.1259659	0.0388738
C	0.0000185	-3.2683616	0.3257243
C	4.7273031	2.2234881	0.0838454
H	3.7883215	2.7437158	0.2042417
C	5.9119160	2.9272739	0.0331116
C	-1.1993062	-2.5773802	0.2697573
H	-2.1407546	-3.1078109	0.3089402
C	-7.1233777	2.2620633	-0.1139382
C	-4.7237637	0.8262240	-0.0152312
C	-5.9118321	2.9273404	0.0331504
C	-4.7272425	2.2235122	0.0839026
C	-5.9499306	0.1657778	-0.1627585
C	-7.1389833	0.8828591	-0.2103556
H	-8.0765872	0.3589414	-0.3238974
I	-6.0338764	-1.9097454	-0.3152982
H	-3.7882464	2.7437017	0.2043530
F	-1.0784994	-5.3239887	-0.0527250
F	-0.0013405	-5.1107000	1.7945725
F	1.0794411	-5.3239090	-0.0506414
C	5.9234656	4.4267691	0.1392399
F	6.6260139	4.8361829	1.2025530
F	4.6950073	4.9369365	0.2528794
F	6.4913166	4.9876123	-0.9357204
C	-5.9233239	4.4268293	0.1394079
F	-6.6256816	4.8361926	1.2028662
F	-6.4913363	4.9877758	-0.9354082
F	-4.6948296	4.9369516	0.2528886
H	-8.0491313	2.8204041	-0.1527745
H	8.0492237	2.8202630	-0.1527130

2F-G3XB bidentate

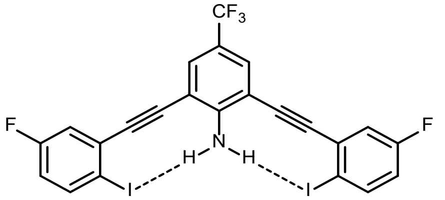

Coordinates for **2F-G3XB bidentate**:

0 1

I	2.9677784	-2.6096644	-0.0150579
C	-0.0147241	5.6193306	0.0098570
N	0.0020129	-0.0321476	0.0043696
H	0.8715768	-0.5401546	0.0075899
H	-0.8667414	-0.5415180	0.0056261
C	7.1244145	-1.4338020	0.0010375
C	4.6699090	-0.0741043	-0.0031239
C	4.7182853	-1.4743409	-0.0064263
C	3.4466610	0.6547417	-0.0051574
C	1.2158940	2.0407521	-0.0086576
C	2.4407665	1.3188937	-0.0071285
C	5.9337938	-2.1423149	-0.0043189
H	5.9542695	-3.2223508	-0.0068891
C	0.0010596	1.3129797	-0.0034724
C	-2.4397475	1.3133005	-0.0110250
C	1.1977193	3.4305876	-0.0186170
H	2.1364724	3.9661488	-0.0268314
C	-1.2165082	2.0381743	-0.0107773
C	-3.4442405	0.6471380	-0.0103856
C	-0.0017449	4.1228952	-0.0267773
C	5.8768192	0.6351977	0.0023162
H	5.8753414	1.7152623	0.0050325
C	7.0686742	-0.0526774	0.0042329
C	-1.2007024	3.4266915	-0.0213990
H	-2.1407883	3.9608306	-0.0320697
C	-7.1179839	-1.4482646	-0.0128230
C	-4.6661360	-0.0840712	-0.0106436
C	-7.0648298	-0.0671212	-0.0277227
C	-5.8742437	0.6229661	-0.0269558
C	-4.7118292	-1.4842945	0.0045026
C	-5.9261052	-2.1545094	0.0033706
H	-5.9446414	-3.2345131	0.0151471
I	-2.9592432	-2.6161293	0.0294302
H	-5.8746975	1.7029679	-0.0389505
F	-1.0292758	6.1273713	-0.7019700
F	1.1167248	6.1437852	-0.4748658
F	-0.1540425	6.0929048	1.2587817
F	8.2115926	0.6409132	0.0094714
F	-8.2089796	0.6242227	-0.0435438
H	-8.0740805	-1.9512497	-0.0140364
H	8.0814508	-1.9349903	0.0027674

2F-G3XB S conformation

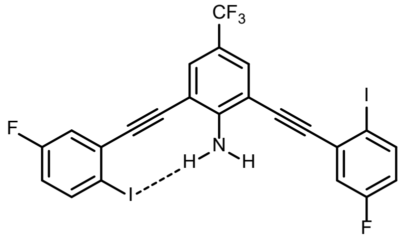

Coordinates for **2F-G3XB-S**:

0 1

I	5.8126004	0.6646000	-0.3983000
C	0.6187000	4.7595003	0.2964000
N	-0.6557000	-0.7445001	0.0725000
H	0.0816000	-1.4201001	-0.0214000
H	-1.6112001	-1.0415001	-0.0426000
C	6.0593004	-3.6182003	0.1490000
C	3.9917003	-1.7247001	0.1415000
C	5.3222004	-1.3347001	-0.0686000
C	2.9212002	-0.7848001	0.1447000
C	0.9948001	1.0024001	0.1536000
C	2.0378001	0.0347000	0.1499000
C	6.3401005	-2.2775002	-0.0620000
H	7.3613005	-1.9645001	-0.2234000
C	-0.3516000	0.5697000	0.1056000
C	-2.7271002	1.1188001	0.0389000
C	1.2903001	2.3590002	0.1984000
H	2.3271002	2.6653002	0.2292000
C	-1.3730001	1.5484001	0.1021000
C	-3.8546003	0.6969001	-0.0203000
C	0.2769000	3.3050002	0.1965000
C	3.7147003	-3.0792002	0.3563000
H	2.7017002	-3.4126002	0.5278000
C	4.7450003	-3.9917003	0.3542000
C	-1.0460001	2.8991002	0.1498000
H	-1.8414001	3.6308003	0.1418000
C	-7.9019006	-0.5111000	-0.2342000
C	-5.2086004	0.2616000	-0.0915000
C	-7.5368006	0.8220001	-0.2301000
C	-6.2226005	1.2243001	-0.1611000
C	-5.5708004	-1.0918001	-0.0954000
C	-6.9035005	-1.4695001	-0.1660000
H	-7.1670005	-2.5171002	-0.1676000
I	-4.1227003	-2.5903002	0.0109000
H	-5.9777005	2.2762002	-0.1606000
F	-0.3439000	5.5370004	-0.2134000
F	1.7543001	5.0515004	-0.3498000
F	0.7940001	5.1501004	1.5690001
F	4.4611003	-5.2828004	0.5602000
F	-8.4923006	1.7546001	-0.2960000

H	-8.9457006	-0.7842001	-0.2898000
H	6.8395005	-4.3655003	0.1564000

2H-G3XB bidentate

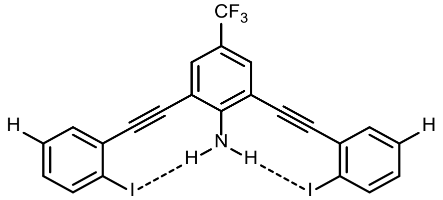

Coordinates for **2H-G3XB bidentate**:

0 1

I	2.9426820	-2.5931106	0.0094086
C	0.0537267	5.6561503	0.0038087
N	-0.0054434	0.0063102	-0.0011298
H	0.8619398	-0.5053422	-0.0006533
H	-0.8768550	-0.4985523	0.0003256
C	7.1026303	-1.4359426	-0.0081033
C	4.6607494	-0.0691552	-0.0060041
C	4.7025736	-1.4686389	-0.0005650
C	3.4415281	0.6672594	-0.0052509
C	1.2215016	2.0716491	-0.0042247
C	2.4414556	1.3402750	-0.0049052
C	5.9120313	-2.1464922	-0.0016011
H	5.9230406	-3.2268269	0.0026466
C	-0.0004917	1.3517605	-0.0023412
C	-2.4391833	1.3650370	-0.0024905
C	1.2100301	3.4587429	-0.0065101
H	2.1524162	3.9901014	-0.0094792
C	-1.2118200	2.0831568	-0.0029889
C	-3.4463038	0.7023031	-0.0018485
C	0.0124647	4.1605091	-0.0072319
C	5.8775993	0.6260023	-0.0125307
H	5.8497631	1.7066081	-0.0167827
C	7.0838541	-0.0476061	-0.0135682
C	-1.1888030	3.4742246	-0.0048484
H	-2.1254253	4.0126222	-0.0062681
C	-7.1272979	-1.3664638	0.0003494
C	-4.6724313	-0.0222894	-0.0011295
C	-7.0954051	0.0216743	0.0007117
C	-5.8829462	0.6840371	-0.0000124
C	-4.7276720	-1.4213718	-0.0015008
C	-5.9433516	-2.0880292	-0.0007567
H	-5.9643320	-3.1682461	-0.0010486
I	-2.9782384	-2.5622915	-0.0032899
H	-5.8451085	1.7643482	0.0002785
F	-1.1637956	6.1986178	-0.0993220
F	0.7916855	6.1438931	-1.0053999

F	0.6028271	6.1355054	1.1314933
H	8.0099384	0.5100541	-0.0187005
H	-8.0162228	0.5880377	0.0015772
H	-8.0718953	-1.8926147	0.0009286
H	8.0422436	-1.9709514	-0.0088971

2H-G3XB S conformation

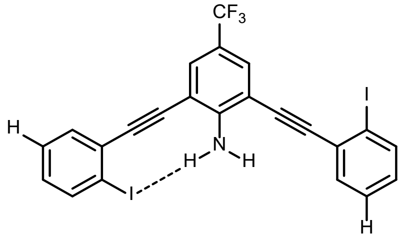

Coordinates for **2H-G3XB-S**:

0 1

I	5.7577752	0.1164299	-0.2900239
C	0.8348525	4.6384819	0.1563167
N	-0.8212901	-0.7660395	0.0963505
H	-0.1329341	-1.4926216	0.0108011
H	-1.7951003	-0.9962522	-0.0187599
C	5.5973291	-4.1674755	0.2638444
C	3.7166931	-2.0995743	0.1945547
C	5.0818538	-1.8327834	0.0227992
C	2.7335314	-1.0685560	0.1654104
C	0.9493517	0.8607832	0.1212577
C	1.9182475	-0.1812681	0.1448006
C	6.0125579	-2.8603577	0.0581235
H	7.0612629	-2.6376597	-0.0755699
C	-0.4244874	0.5243828	0.0896781
C	-2.7568786	1.2362211	0.0219528
C	1.3402424	2.1939585	0.1256194
H	2.3960014	2.4274420	0.1441448
C	-1.3751882	1.5715642	0.0629372
C	-3.9116800	0.8921236	-0.0132446
C	0.3958978	3.2084139	0.0991379
C	3.3217610	-3.4269955	0.4028819
H	2.2699396	-3.6357201	0.5418272
C	4.2493238	-4.4507049	0.4366729
C	-0.9532178	2.8960150	0.0699043
H	-1.6948937	3.6818611	0.0437797
C	-8.0325750	-0.0194733	-0.1352774
C	-5.2947147	0.5542360	-0.0543816
C	-7.5996349	1.2995766	-0.1159863
C	-6.2476746	1.5816040	-0.0760893
C	-5.7532289	-0.7684578	-0.0744427
C	-7.1093439	-1.0538144	-0.1146393
H	-7.4428006	-2.0813310	-0.1296276

I	-4.4095310	-2.3675363	-0.0443393
H	-5.8980626	2.6043495	-0.0607076
F	-0.0487017	5.4582224	-0.4271377
F	2.0142747	4.8255347	-0.4485516
F	0.9814303	5.0721710	1.4190234
H	3.9218406	-5.4680729	0.5991456
H	-8.3162749	2.1087877	-0.1320334
H	-9.0887356	-0.2489382	-0.1665894
H	6.3307090	-4.9616801	0.2893967

2Me-G3XB bidentate

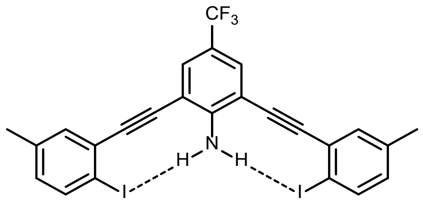

Coordinates for **2Me-G3XB bidentate**:

0 1

I	2.9646706	-2.6111428	0.2748366
C	-0.0155659	5.6095114	0.0091426
N	0.0020403	-0.0402137	0.0039999
H	0.8717356	-0.5479056	-0.0030146
H	-0.8668330	-0.5492489	0.0117484
C	7.1065439	-1.4399278	-0.0780195
C	4.6646825	-0.0943323	-0.0458859
C	4.7117459	-1.4863778	0.0690729
C	3.4443037	0.6403480	-0.0351364
C	1.2163168	2.0320002	-0.0208486
C	2.4407327	1.3084176	-0.0294344
C	5.9280851	-2.1534363	0.0526407
H	5.9527768	-3.2299736	0.1423342
C	0.0010311	1.3051223	-0.0043221
C	-2.4399649	1.3027857	0.0099320
C	1.1977753	3.4217009	-0.0318000
H	2.1364356	3.9570894	-0.0492461
C	-1.2172152	2.0294290	-0.0002776
C	-3.4420193	0.6325938	0.0190759
C	-0.0020021	4.1138914	-0.0277889
C	5.8759268	0.6004649	-0.1766051
H	5.8357362	1.6781813	-0.2654624
C	7.0974553	-0.0501808	-0.1945672
C	-1.2011943	3.4177109	-0.0099859
H	-2.1413904	3.9517579	-0.0111707
C	-7.1000867	-1.4545932	0.0707880
C	-4.6609456	-0.1045283	0.0332803
C	-7.0940902	-0.0639182	0.1757394
C	-5.8737973	0.5889812	0.1552572

C	-4.7048496	-1.4975709	-0.0700570
C	-5.9199157	-2.1668587	-0.0509850
H	-5.9422959	-3.2441444	-0.1317258
I	-2.9550177	-2.6204575	-0.2622128
H	-5.8359316	1.6674811	0.2351322
F	-1.0308044	6.1189763	-0.7019032
F	1.1152121	6.1363366	-0.4759412
F	-0.1548202	6.0848282	1.2581794
C	8.3850132	0.7117449	-0.3370439
C	-8.3834831	0.6965527	0.3090428
H	-8.0421659	-1.9886893	0.0844441
H	8.0496542	-1.9722427	-0.0895318
H	8.2053320	1.7827826	-0.4022574
H	8.9206363	0.4011645	-1.2347435
H	9.0400353	0.5272024	0.5149550
H	-8.2062456	1.7685861	0.3636898
H	-8.9194084	0.3938342	1.2092520
H	-9.0371437	0.5019806	-0.5417642

2Me-G3XB S conformation

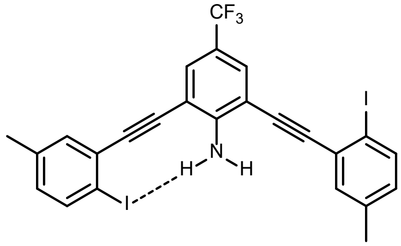

Coordinates for **2Me-G3XB-S**:

0 1

I	5.8345231	0.6955690	-0.4249696
C	0.5767275	4.7861550	0.3105710
N	-0.6309208	-0.7315336	0.0804033
H	0.1158566	-1.3941142	-0.0318829
H	-1.5816062	-1.0374442	-0.0514047
C	6.0441560	-3.5811449	0.1669379
C	4.0072774	-1.6811573	0.1494180
C	5.3342308	-1.2986090	-0.0730139
C	2.9377601	-0.7393765	0.1514153
C	0.9986276	1.0347786	0.1620502
C	2.0507825	0.0764866	0.1573423
C	6.3448491	-2.2495899	-0.0614307
H	7.3680179	-1.9463347	-0.2310427
C	-0.3428269	0.5873410	0.1130972
C	-2.7254430	1.1057136	0.0471676
C	1.2778709	2.3945874	0.2089635
H	2.3109366	2.7132100	0.2402373
C	-1.3765582	1.5526140	0.1111258
C	-3.8471296	0.6679899	-0.0125924

C	0.2527485	3.3283351	0.2083265
C	3.7337286	-3.0363007	0.3801934
H	2.7064128	-3.3266973	0.5593796
C	4.7317470	-3.9954329	0.3908632
C	-1.0654475	2.9069623	0.1611412
H	-1.8695678	3.6290499	0.1542743
C	-7.8683475	-0.5669017	-0.2219119
C	-5.1958724	0.2152892	-0.0831246
C	-7.5607714	0.7933577	-0.2047174
C	-6.2286212	1.1625997	-0.1359642
C	-5.5410819	-1.1387258	-0.1021581
C	-6.8716261	-1.5255184	-0.1713859
H	-7.1274478	-2.5753208	-0.1852736
I	-4.0767430	-2.6248140	-0.0215110
H	-5.9576061	2.2101156	-0.1211871
F	-0.3958500	5.5540572	-0.1963879
F	1.7078124	5.0948122	-0.3362573
F	0.7489060	5.1780148	1.5837449
C	4.4193384	-5.4454892	0.6328456
H	4.6618725	-6.0446848	-0.2456690
H	3.3652494	-5.5902651	0.8608066
H	5.0050520	-5.8347696	1.4658932
C	-8.6539194	1.8231585	-0.2598087
H	-9.3339176	1.7104601	0.5851582
H	-9.2430970	1.7132192	-1.1707764
H	-8.2478589	2.8322323	-0.2372509
H	-8.9033382	-0.8813269	-0.2755849
H	6.8441292	-4.3113012	0.1729672

2*t*Bu-G3XB bidentate

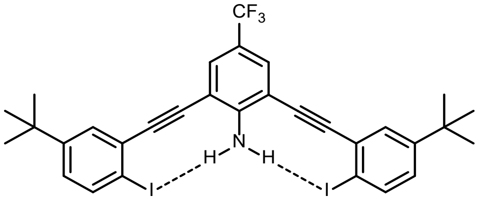

Coordinates for **2*t*BuG3XB bidentate**:

0 1

I	2.9590790	-2.7471867	0.3474641
C	-0.0117364	5.4754927	0.0109845
N	0.0017177	-0.1741229	0.0049849
H	0.8711138	-0.6821653	0.0182635
H	-0.8674337	-0.6826595	-0.0009859
C	7.1032327	-1.5688572	0.0509412
C	4.6643910	-0.2317019	0.0420982
C	4.7073447	-1.6212303	0.1636017
C	3.4443828	0.5041539	0.0311886
C	1.2173905	1.8974380	0.0039260

C	2.4413872	1.1730661	0.0184128
C	5.9258016	-2.2843465	0.1676250
H	5.9533306	-3.3604582	0.2620500
C	0.0014986	1.1713165	-0.0025240
C	-2.4393781	1.1700854	-0.0334309
C	1.1997417	3.2870691	-0.0075549
H	2.1388438	3.8220140	-0.0073003
C	-1.2162114	1.8961391	-0.0212773
C	-3.4418492	0.5004139	-0.0422280
C	0.0003798	3.9799558	-0.0262690
C	5.8772629	0.4664167	-0.0743548
H	5.8154914	1.5403520	-0.1674658
C	7.1045465	-0.1762623	-0.0727046
C	-1.1992744	3.2844956	-0.0308226
H	-2.1389844	3.8190021	-0.0498165
C	-7.0989417	-1.5755657	-0.0528319
C	-4.6612748	-0.2365422	-0.0495904
C	-7.1015944	-0.1821307	0.0608539
C	-5.8748027	0.4615000	0.0598767
C	-4.7028290	-1.6269355	-0.1610928
C	-5.9207789	-2.2909985	-0.1623637
H	-5.9473456	-3.3677804	-0.2490491
I	-2.9532959	-2.7526196	-0.3339462
H	-5.8139921	1.5361228	0.1453278
F	-1.0222941	5.9865853	-0.7054682
F	1.1224834	6.0011496	-0.4676540
F	-0.1568615	5.9509297	1.2594062
C	8.4293470	0.5717438	-0.2003671
C	-8.4271864	0.5657191	0.1809900
H	-8.0339675	-2.1202993	-0.0562001
H	8.0386876	-2.1128352	0.0566464
C	8.2293995	2.0816655	-0.3241367
C	-8.2285679	2.0765867	0.2950205
H	9.2009987	2.5684543	-0.4123214
H	7.7284439	2.4940827	0.5527754
H	7.6463860	2.3386732	-1.2095869
H	-9.2006741	2.5632170	0.3783159
H	-7.7264354	2.4835577	-0.5837598
H	-7.6472667	2.3398964	1.1797463
C	-9.2819543	0.2808325	-1.0618022
C	-9.1695710	0.0779045	1.4329107
H	-8.5791272	0.2665304	2.3303003
H	-9.3804632	-0.9903028	1.3846944
H	-10.1206630	0.6040112	1.5299606
C	9.2867081	0.2958561	1.0426648
C	9.1696958	0.0760811	-1.4504140
H	8.7788933	0.6364115	1.9458117
H	10.2381134	0.8242918	0.9628725

H	9.5016360	-0.7666764	1.1553416
H	8.5773602	0.2582231	-2.3478974
H	9.3814663	-0.9916257	-1.3954250
H	10.1202017	0.6022332	-1.5528373
H	-8.7726222	0.6156039	-1.9662561
H	-10.2338532	0.8091623	-0.9874064
H	-9.4959739	-0.7825883	-1.1676745

2*t*Bu-G3XB S conformation

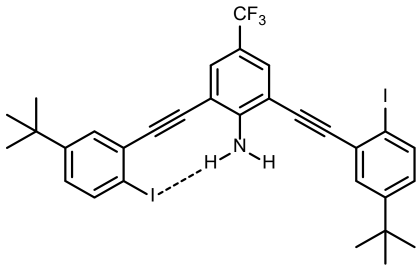

Coordinates for **2*t*Bu-G3XB-S**:

0 1

I	5.8759896	2.0307369	-0.6482970
C	-0.0257677	5.1286818	0.4635703
N	-0.1990384	-0.5058303	0.0399261
H	0.6576898	-1.0069796	-0.1161618
H	-1.0757009	-0.9781869	-0.1127251
C	6.8399465	-2.1334158	0.0347702
C	4.5141474	-0.6047665	0.0821772
C	5.7445564	-0.0072930	-0.2247456
C	3.2983503	0.1393872	0.1178569
C	1.0699166	1.5312695	0.1931960
C	2.2812228	0.7846030	0.1530378
C	6.8948156	-0.7739150	-0.2465707
H	7.8428832	-0.3130226	-0.4844519
C	-0.1653366	0.8418934	0.1262005
C	-2.6024720	0.9031064	0.0943354
C	1.0881406	2.9147954	0.2947365
H	2.0430208	3.4218857	0.3406577
C	-1.3609941	1.5935821	0.1691325
C	-3.6222142	0.2638814	0.0246133
C	-0.0955996	3.6389277	0.3364080
C	4.4907780	-1.9726706	0.3633136
H	3.5329787	-2.4135283	0.6070349
C	5.6371149	-2.7606717	0.3471436
C	-1.3103272	2.9805511	0.2755856
H	-2.2357906	3.5370058	0.3053947
C	-7.3371625	-1.7077051	-0.1879428
C	-4.8609613	-0.4363369	-0.0520901
C	-7.3041877	-0.3277187	-0.0060763
C	-6.0551379	0.2811086	0.0581345

C	-4.9376469	-1.8229665	-0.2346955
C	-6.1678626	-2.4490326	-0.3014534
H	-6.2218618	-3.5191159	-0.4424286
I	-3.2122314	-2.9852093	-0.4130537
F	-1.2306512	5.7023495	0.3757535
F	0.7441374	5.6763096	-0.4885545
F	0.5044260	5.5082811	1.6368344
C	5.5330078	-4.2496851	0.6712711
C	-8.5644273	0.5255278	0.1227306
H	-8.2797192	-2.2319922	-0.2449313
H	7.7614160	-2.6962920	0.0066486
C	6.8909020	-4.9475144	0.6008458
C	-9.8372216	-0.3154906	0.0308714
H	-10.7070715	0.3345210	0.1284735
H	-9.8858960	-1.0590590	0.8276951
H	-9.9112780	-0.8292020	-0.9287043
H	6.7666254	-6.0042555	0.8388816
H	7.5969706	-4.5254431	1.3173191
H	7.3264237	-4.8793415	-0.3970457
C	-8.5542568	1.2433526	1.4794907
H	-8.5334075	0.5229522	2.2981286
H	-9.4515136	1.8557116	1.5828289
H	-7.6883169	1.8969520	1.5821168
C	-8.5883996	1.5668326	-1.0047808

2MeO-G3XB bidentate

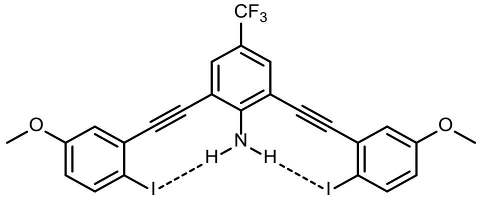

Coordinates for **2MeO-G3XB bidentate**:

0 1

I	2.9505845	-2.7223297	0.0140013
C	-0.0130552	5.5124485	0.0125191
N	0.0022144	-0.1372898	0.0058355
H	0.8717900	-0.6456915	0.0066149
H	-0.8667688	-0.6466719	0.0065441
C	7.1055209	-1.5471748	-0.0050216
C	4.6642964	-0.1942129	-0.0055345
C	4.7020424	-1.5885701	0.0023808
C	3.4443698	0.5420916	-0.0062896
C	1.2171430	1.9346256	-0.0081017
C	2.4414562	1.2110668	-0.0074879
C	5.9226193	-2.2549464	0.0025869
H	5.9449465	-3.3352170	0.0087211
C	0.0015541	1.2074791	-0.0020983

C	-2.4394399	1.2065928	-0.0079615
C	1.1988502	3.3241880	-0.0179647
H	2.1376776	3.8595235	-0.0268314
C	-1.2164387	1.9325986	-0.0083826
C	-3.4413406	0.5362029	-0.0067369
C	-0.0006602	4.0169443	-0.0251291
C	5.8737762	0.5193043	-0.0132695
H	5.8242049	1.5969514	-0.0193406
C	7.0861093	-0.1508996	-0.0130222
C	-1.1999177	3.3208618	-0.0187028
H	-2.1398498	3.8552166	-0.0284983
C	-7.1001553	-1.5569650	-0.0041618
C	-4.6604679	-0.2014937	-0.0057221
C	-7.0822868	-0.1606582	-0.0071783
C	-5.8706456	0.5108221	-0.0079475
C	-4.6966097	-1.5958990	-0.0027139
C	-5.9164986	-2.2635383	-0.0019523
H	-5.9377097	-3.3438309	0.0003736
I	-2.9439152	-2.7277489	0.0005196
H	-5.8222246	1.5885346	-0.0102327
F	-1.0294265	6.0231083	-0.6959916
F	1.1172233	6.0385064	-0.4749420
F	-0.1486409	5.9876282	1.2620135
O	8.2919767	0.4589672	-0.0201468
O	-8.2887858	0.4479369	-0.0091545
H	-8.0542253	-2.0648703	-0.0035895
H	8.0601335	-2.0540574	-0.0049576
C	8.3174975	1.8702218	-0.0287750
C	-8.3158317	1.8591982	-0.0121224
H	9.3651783	2.1543211	-0.0336675
H	7.8337955	2.2778259	0.8617383
H	7.8283974	2.2669972	-0.9212254
H	-9.3638266	2.1421883	-0.0133047
H	-7.8293710	2.2600504	-0.9041844
H	-7.8303727	2.2637728	0.8788158

2MeO-G3XB S conformation

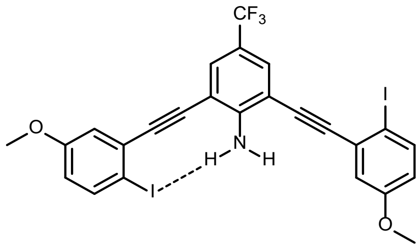

Coordinates for **2MeO-G3XB-S**:

0 1

I	5.8855740	1.2594806	-0.2342502
C	0.3458913	4.9379282	0.1711974

N	-0.4852575	-0.6515994	0.0514540
H	0.3039796	-1.2686033	-0.0205578
H	-1.4160340	-1.0287447	-0.0240516
C	6.3905553	-3.0332719	0.0544009
C	4.2083992	-1.2811686	0.0605672
C	5.5181012	-0.7858852	-0.0593281
C	3.0748526	-0.4166420	0.0669589
C	1.0205659	1.2220578	0.0800895
C	2.1356071	0.3380800	0.0732302
C	6.5866704	-1.6619617	-0.0608278
H	7.5929509	-1.2789718	-0.1532059
C	-0.2879156	0.6826873	0.0624731
C	-2.7018128	1.0379452	0.0363787
C	1.2065830	2.5982753	0.0994386
H	2.2158349	2.9871564	0.1072555
C	-1.3851796	1.5755416	0.0636184
C	-3.7941852	0.5293014	0.0110331
C	0.1200831	3.4599382	0.1007837
C	4.0194426	-2.6554677	0.1769670
H	3.0219976	-3.0596034	0.2748801
C	5.0979950	-3.5326143	0.1743349
C	-1.1668018	2.9484943	0.0847067
H	-2.0186603	3.6136616	0.0806109
C	-7.7349446	-0.9935512	-0.0761072
C	-5.1114190	-0.0142148	-0.0183358
C	-7.5034169	0.3779626	-0.0421868
C	-6.1982082	0.8564900	-0.0138344
C	-5.3584316	-1.3962271	-0.0523524
C	-6.6558404	-1.8691547	-0.0808567
H	-6.8395613	-2.9336291	-0.1071846
I	-3.7908790	-2.7740702	-0.0598207
H	-6.0335379	1.9240099	0.0121993
F	-0.6885336	5.6288668	-0.3233757
F	1.4375918	5.3109106	-0.5089021
F	0.5220179	5.3632856	1.4330768
O	4.7924460	-4.8453001	0.2936510
O	-8.4761534	1.3165579	-0.0344174
H	-8.7367052	-1.3943495	-0.0986169
H	7.2479754	-3.6887514	0.0494280
C	5.8582137	-5.7696466	0.3010762
C	-9.8164448	0.8764701	-0.0612986
H	-10.4297803	1.7720756	-0.0493298
H	-10.0481475	0.2655453	0.8141629
H	-10.0254496	0.3050041	-0.9685260
H	5.4075117	-6.7516411	0.4066215
H	6.5323235	-5.5870162	1.1410839
H	6.4230865	-5.7275210	-0.6330135

Cl-G3XB bidentate

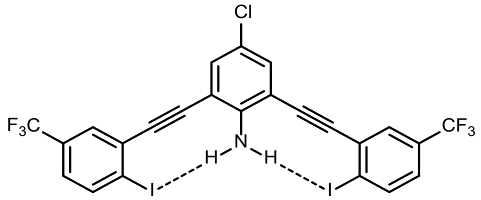

Coordinates for **Cl-G3XB bidentate**:

0 1

I	3.0044819	-2.4755100	-0.0549749
Cl	0.0000345	5.9789710	-0.1222959
N	-0.0000139	0.0873892	-0.0417882
H	0.8671318	-0.4144874	0.0548511
H	-0.8671607	-0.4144883	0.0548365
C	7.1409464	-1.2592923	0.0659764
C	4.6771222	0.0724899	0.0111693
C	4.7426461	-1.3252391	0.0028969
C	3.4482801	0.7897829	-0.0148094
C	1.2120720	2.1662339	-0.0547692
C	2.4385806	1.4481646	-0.0356368
C	5.9647389	-1.9848127	0.0303863
H	5.9944558	-3.0645325	0.0235876
C	-0.0000078	1.4386190	-0.0450889
C	-2.4385876	1.4481946	-0.0356610
C	1.1994054	3.5588488	-0.0787814
H	2.1355505	4.0982115	-0.0862587
C	-1.2120738	2.1662529	-0.0547833
C	-3.4482868	0.7898114	-0.0148469
C	0.0000146	4.2467277	-0.0919868
C	5.8809511	0.7905711	0.0473660
H	5.8442388	1.8700271	0.0540583
C	7.0907218	0.1300995	0.0739892
C	-1.1993825	3.5588700	-0.0787947
H	-2.1355223	4.0982427	-0.0862829
C	-7.1409422	-1.2592892	0.0658828
C	-4.6771243	0.0725140	0.0111152
C	-7.0907207	0.1301081	0.0740933
C	-5.8809598	0.7905857	0.0474892
C	-4.7426451	-1.3252084	0.0026526
C	-5.9647377	-1.9847961	0.0301196
H	-5.9944420	-3.0645151	0.0231683
I	-3.0044897	-2.4754821	-0.0554770
H	-5.8442470	1.8700411	0.0543338
C	8.3856977	0.8933359	0.1134548
F	9.0958393	0.5912504	1.2077749
F	8.1978365	2.2139457	0.1114674
F	9.1580769	0.5967110	-0.9393402
C	-8.3857012	0.8933259	0.1137475

F	-9.0957953	0.5910385	1.2080414
F	-9.1581202	0.5968814	-0.9390695
F	-8.1978514	2.2139386	0.1119867
H	-8.0944556	-1.7697103	0.0871304
H	8.0944585	-1.7697149	0.0872413

Cl-G3XB S conformation

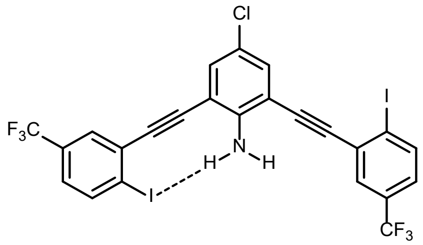

Coordinates for **Cl-G3XB-S**:

0 1

I	-5.8875688	2.2829669	0.5330351
Cl	0.0251806	5.6145805	-0.5743805
N	0.2108299	-0.2577320	-0.0851129
H	-0.6357400	-0.7520566	0.1362036
H	1.0880358	-0.7064947	0.1245467
C	-6.8456652	-1.8905321	-0.0161655
C	-4.5052239	-0.3580744	-0.0986216
C	-5.7437700	0.2360744	0.1739884
C	-3.2947436	0.3906438	-0.1466916
C	-1.0667300	1.7798960	-0.2469099
C	-2.2771735	1.0344391	-0.1937884
C	-6.9034166	-0.5281609	0.2129281
H	-7.8508023	-0.0548528	0.4252386
C	0.1662405	1.0957232	-0.1750790
C	2.6024520	1.1810071	-0.1458678
C	-1.0988226	3.1671544	-0.3674427
H	-2.0530597	3.6716254	-0.4199502
C	1.3542643	1.8569944	-0.2288618
C	3.6308613	0.5572272	-0.0661419
C	0.0789270	3.8899119	-0.4216046
C	-4.4664347	-1.7389866	-0.3317759
H	-3.5177782	-2.2069611	-0.5507524
C	-5.6216814	-2.4905696	-0.2880773
C	1.2990358	3.2437340	-0.3533924
H	2.2179052	3.8105319	-0.3934106
C	7.3776227	-1.3765699	0.1826384
C	4.8790973	-0.1212290	0.0212513
C	7.2908689	-0.0016264	-0.0039767
C	6.0637093	0.6213775	-0.0840908
C	4.9811749	-1.5041557	0.2089775

C	6.2207181	-2.1257545	0.2890480
H	6.2791265	-3.1945299	0.4342284
I	3.2737279	-2.6890541	0.3752127
H	5.9984374	1.6896720	-0.2292480
C	-5.5877014	-3.9718101	-0.5416778
F	-6.1106933	-4.6551325	0.4841495
F	-4.3467385	-4.4260812	-0.7315907
F	-6.3025796	-4.2992999	-1.6252847
C	8.5657564	0.7873001	-0.1203746
F	9.2947663	0.3822498	-1.1679339
F	9.3345126	0.6334563	0.9649970
F	8.3430502	2.0938835	-0.2704057
H	8.3445574	-1.8575552	0.2445357
H	-7.7480470	-2.4862697	0.0166023

F-G3XB bidentate

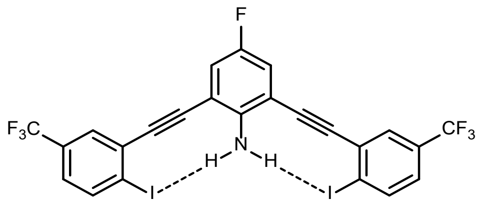

Coordinates for **F-G3XB bidentate**:

0 1

I	-2.9988322	-2.3109140	-0.1456544
F	0.0010717	5.7267431	-0.3540432
N	0.0001995	0.2426987	-0.0613461
H	-0.8627843	-0.2397408	0.1296771
H	0.8628595	-0.2399958	0.1306220
C	-7.1310854	-1.1084790	0.1461332
C	-4.6739969	0.2303227	0.0177584
C	-4.7365414	-1.1675114	-0.0065678
C	-3.4475924	0.9500164	-0.0437706
C	-1.2118090	2.3241595	-0.1474108
C	-2.4379427	1.6067568	-0.0953698
C	-5.9554730	-1.8303328	0.0574398
H	-5.9829556	-2.9099419	0.0381050
C	0.0003627	1.5992847	-0.1080204
C	2.4386155	1.6058458	-0.0960375
C	-1.2016101	3.7160337	-0.2302779
H	-2.1297845	4.2680636	-0.2612098
C	1.2127782	2.3237165	-0.1478915
C	3.4480189	0.9487348	-0.0442359
C	0.0008572	4.3862331	-0.2726935
C	-5.8770966	0.9447371	0.1079967
H	-5.8424044	2.0240621	0.1290924
C	-7.0836529	0.2807615	0.1708998
C	1.2030831	3.7155961	-0.2309740

H	2.1314536	4.2672683	-0.2623827
C	7.1318295	-1.1091934	0.1468215
C	4.6743972	0.2291568	0.0182673
C	7.0856337	0.2802919	0.1445485
C	5.8789098	0.9440178	0.0820025
C	4.7357633	-1.1689411	0.0203314
C	5.9548234	-1.8315010	0.0844542
H	5.9813869	-2.9113085	0.0847429
I	2.9963527	-2.3138953	-0.0786417
H	5.8454449	2.0236046	0.0804295
C	-8.3782436	1.0403010	0.2612149
F	-9.1521197	0.8130444	-0.8078677
F	-8.1898351	2.3583224	0.3436770
F	-9.0872549	0.6683576	1.3343011
C	8.3804047	1.0406239	0.2252115
F	9.2216010	0.6760866	-0.7503271
F	9.0109832	0.8065047	1.3832872
F	8.2025788	2.3591644	0.1282625
H	8.0829096	-1.6221548	0.1958292
H	-8.0819331	-1.6216540	0.1976198

F-G3XB S conformation

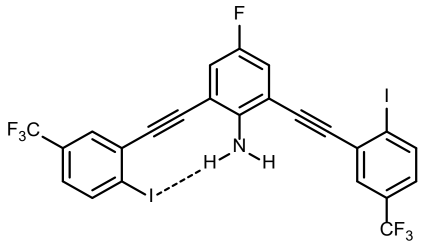

Coordinates for **F-G3XB-S**:

0 1

I	5.8868596	-2.4347896	0.5038344
F	-0.0425510	-5.3493153	-0.6871336
N	-0.2072426	0.1134535	-0.1137311
H	0.6348264	0.5860607	0.1667350
H	-1.0810458	0.5441350	0.1443116
C	6.8393775	1.7466239	0.0080451
C	4.5032670	0.2099318	-0.1094972
C	5.7411495	-0.3844060	0.1654304
C	3.2947603	-0.5404456	-0.1758205
C	1.0671115	-1.9268020	-0.3133905
C	2.2776315	-1.1833120	-0.2402270
C	6.8987658	0.3817786	0.2215383
H	7.8456900	-0.0918642	0.4350793
C	-0.1650101	-1.2440997	-0.2276385
C	-2.6011771	-1.3239457	-0.1991191

C	1.0999998	-3.3122299	-0.4661709
H	2.0462346	-3.8301839	-0.5303683
C	-1.3544486	-2.0002066	-0.2996269
C	-3.6291561	-0.7020059	-0.1016037
C	-0.0823364	-4.0156719	-0.5374411
C	4.4627870	1.5934478	-0.3266692
H	3.5144235	2.0613884	-0.5471371
C	5.6159369	2.3470718	-0.2654595
C	-1.3039416	-3.3853609	-0.4568116
H	-2.2154660	-3.9626061	-0.5118924
C	-7.3744836	1.2261504	0.2052259
C	-4.8768287	-0.0253414	0.0054589
C	-7.2887451	-0.1457187	-0.0032454
C	-6.0619673	-0.7667552	-0.1023206
C	-4.9777496	1.3545499	0.2153642
C	-6.2170169	1.9740708	0.3144370
H	-6.2746679	3.0404354	0.4766349
I	-3.2692413	2.5368406	0.3860503
H	-5.9974411	-1.8326750	-0.2642242
C	5.5804125	3.8310975	-0.5020766
F	6.1009517	4.5031753	0.5324325
F	4.3392935	4.2859369	-0.6888183
F	6.2967018	4.1719904	-1.5806626
C	-8.5640183	-0.9333734	-0.1231853
F	-9.2952740	-0.5201334	-1.1660308
F	-9.3305862	-0.7880688	0.9648914
F	-8.3419342	-2.2388678	-0.2840831
H	-8.3410519	1.7057115	0.2821085
H	7.7400918	2.3439764	0.0544424

H-G3XB bidentate

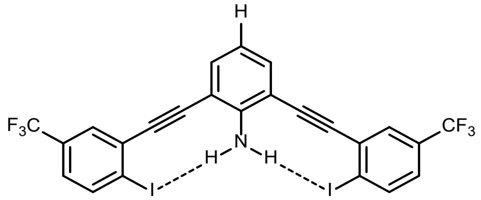

Coordinates for **H-G3XB bidentate**:

0 1

I	-2.9972002	-2.1570002	0.3511000
N	0.0000000	0.3969000	-0.0016000
H	-0.8685001	-0.1107000	0.0063000
H	0.8687001	-0.1107000	-0.0042000
C	-7.1335005	-0.9727001	0.0492000
C	-4.6734003	0.3703000	0.0398000
C	-4.7366003	-1.0223001	0.1629000
C	-3.4471002	1.0920001	0.0306000
C	-1.2134001	2.4740002	0.0088000

C	-2.4381002	1.7526001	0.0204000
C	-5.9560004	-1.6872001	0.1673000
H	-5.9825004	-2.7627002	0.2633000
C	0.0000000	1.7470001	-0.0008000
C	2.4381002	1.7527001	-0.0214000
C	-1.1943001	3.8673003	0.0073000
H	-2.1393002	4.3929003	0.0141000
C	1.2134001	2.4740002	-0.0102000
C	3.4471002	1.0921001	-0.0312000
C	0.0000000	4.5687003	-0.0006000
C	-5.8790004	1.0769001	-0.0788000
H	-5.8450004	2.1521002	-0.1752000
C	-7.0863005	0.4114000	-0.0735000
C	1.1942001	3.8673003	-0.0085000
H	2.1392002	4.3930003	-0.0150000
C	7.1335005	-0.9727001	-0.0482000
C	4.6734003	0.3704000	-0.0398000
C	7.0863005	0.4114000	0.0743000
C	5.8790004	1.0769001	0.0791000
C	4.7366003	-1.0222001	-0.1628000
C	5.9560004	-1.6872001	-0.1666000
H	5.9825004	-2.7627002	-0.2625000
I	2.9972002	-2.1569002	-0.3516000
H	5.8451004	2.1521002	0.1754000
C	-8.3821006	1.1634001	-0.1991000
F	-8.1974006	2.4789002	-0.3230000
F	-9.1648007	0.9683001	0.8699001
F	-9.0813007	0.7551001	-1.2658001
C	8.3820006	1.1633001	0.2004000
F	9.1650006	0.9685001	-0.8685001
F	9.0811006	0.7548001	1.2671001
F	8.1974006	2.4788002	0.3246000
H	8.0850006	-1.4872001	-0.0505000
H	-8.0850006	-1.4871001	0.0520000
H	-0.0001000	5.6485004	-0.0005000

H-G3XB S conformation

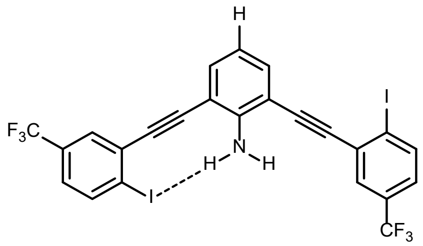

Coordinates for **H-G3XB-S**:
0 1

I	5.8856302	-2.5753849	0.4409753
H	-0.0492608	-5.2372218	-0.7767792
N	-0.2092416	-0.0251502	-0.1157810
H	0.6378872	0.4584852	0.1258917
H	-1.0860889	0.4151574	0.1120381
C	6.8417493	1.6131372	0.0186682
C	4.5026911	0.0800328	-0.1248143
C	5.7409925	-0.5190687	0.1382203
C	3.2938507	-0.6680473	-0.2040016
C	1.0671669	-2.0544309	-0.3656379
C	2.2767577	-1.3106386	-0.2794109
C	6.8995858	0.2445323	0.2069028
H	7.8461232	-0.2341849	0.4105881
C	-0.1661368	-1.3735008	-0.2652604
C	-2.6022971	-1.4538426	-0.2396925
C	1.0914697	-3.4360217	-0.5462406
H	2.0533913	-3.9253105	-0.6178395
C	-1.3562488	-2.1293684	-0.3524164
C	-3.6305788	-0.8332380	-0.1320592
C	-0.0816744	-4.1672321	-0.6346458
C	4.4644555	1.4676282	-0.3162983
H	3.5165036	1.9407053	-0.5272143
C	5.6182864	2.2191005	-0.2422243
C	-1.2963544	-3.5099343	-0.5371968
H	-2.2251772	-4.0600031	-0.6003874
C	-7.3778544	1.0885174	0.2056969
C	-4.8782382	-0.1591264	-0.0142590
C	-7.2908889	-0.2799351	-0.0238209
C	-6.0637867	-0.8986420	-0.1330058
C	-4.9811018	1.2172974	0.2169799
C	-6.2204960	1.8348819	0.3259128
H	-6.2783438	2.8985989	0.5046536
I	-3.2731196	2.3979568	0.4070787
H	-5.9986977	-1.9618975	-0.3111755
C	5.5834793	3.7071513	-0.4509774
F	6.1041924	4.3599341	0.5959286
F	4.3427129	4.1665075	-0.6295277
F	6.3001903	4.0683315	-1.5228969
C	-8.5653469	-1.0664939	-0.1563721
F	-9.2924207	-0.6446193	-1.1989634
F	-9.3373681	-0.9311560	0.9293151
F	-8.3428668	-2.3707900	-0.3278549
H	-8.3446068	1.5662954	0.2905146
H	7.7430484	2.2086967	0.0750242

Me-G3XB bidentate

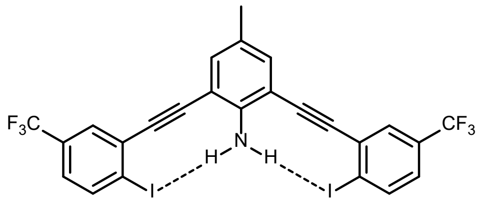

Coordinates for **Me-G3XB bidentate**:

0 1

I	-3.0128835	-2.3252616	-0.1553788
C	0.0013383	5.9118906	-0.4032012
N	0.0000786	0.2192494	-0.0610367
H	-0.8639668	-0.2658666	0.1172965
H	0.8637942	-0.2662246	0.1180285
C	-7.1403483	-1.1100450	0.1525313
C	-4.6774740	0.2215368	0.0218470
C	-4.7462850	-1.1761141	-0.0072159
C	-3.4490637	0.9368319	-0.0408726
C	-1.2080826	2.3032873	-0.1494467
C	-2.4365595	1.5899360	-0.0938517
C	-5.9672433	-1.8351456	0.0578893
H	-5.9981525	-2.9146109	0.0346921
C	0.0003150	1.5739539	-0.1119804
C	2.4371915	1.5888132	-0.0947814
C	-1.1860971	3.6947303	-0.2311459
H	-2.1334645	4.2185652	-0.2552940
C	1.2090471	2.3027188	-0.1500982
C	3.4494689	0.9353631	-0.0416401
C	0.0009545	4.4128823	-0.2787879
C	-5.8786823	0.9388867	0.1180831
H	-5.8400969	2.0179649	0.1429673
C	-7.0874032	0.2789624	0.1820823
C	1.1876956	3.6941523	-0.2320389
H	2.1353033	4.2175273	-0.2568556
C	7.1411609	-1.1107767	0.1530031
C	4.6778871	0.2202399	0.0220829
C	7.0894616	0.2785751	0.1555135
C	5.8805442	0.9381963	0.0918757
C	4.7455135	-1.1777732	0.0193976
C	5.9666233	-1.8364881	0.0846498
H	5.9966022	-2.9162260	0.0810663
I	3.0103090	-2.3286430	-0.0887905
H	5.8432171	2.0176235	0.0940635
H	0.8824052	6.3444208	0.0689138
H	-0.8811267	6.3446163	0.0660938
H	0.0030671	6.2190716	-1.4505657
C	-8.3786615	1.0428470	0.2784755
F	-9.1563051	0.8237292	-0.7899005
F	-8.1857552	2.3601514	0.3663535

F	-9.0875423	0.6693453	1.3513837
C	8.3808652	1.0433299	0.2426404
F	9.2273292	0.6851554	-0.7310412
F	9.0091735	0.8087955	1.4021443
F	8.1991271	2.3618507	0.1488387
H	8.0938714	-1.6205603	0.2028625
H	-8.0927917	-1.6201019	0.2048950

Me-G3XB S conformation

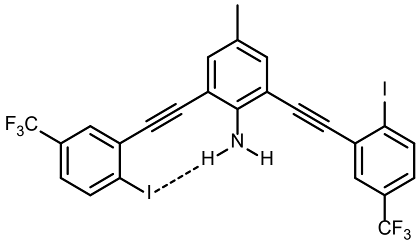

Coordinates for **Me-G3XB-S**:

0 1

I	5.8875365	-2.4305623	0.5031895
C	-0.0360083	-5.5357503	-0.7164516
N	-0.2063570	0.1367434	-0.1190666
H	0.6374890	0.6148334	0.1456681
H	-1.0810333	0.5731583	0.1253353
C	6.8487751	1.7493698	0.0138411
C	4.5072950	0.2172846	-0.1029610
C	5.7450883	-0.3789615	0.1685873
C	3.2970532	-0.5295988	-0.1687559
C	1.0636570	-1.9079034	-0.3070430
C	2.2771916	-1.1690310	-0.2330491
C	6.9048278	0.3839655	0.2243267
H	7.8508110	-0.0926865	0.4353681
C	-0.1648316	-1.2192839	-0.2283515
C	-2.5991894	-1.3062846	-0.1952500
C	1.0839337	-3.2939469	-0.4503677
H	2.0479018	-3.7849765	-0.5023012
C	-1.3506431	-1.9793925	-0.2950881
C	-3.6298222	-0.6875282	-0.0994159
C	-0.0822083	-4.0444557	-0.5260250
C	4.4709619	1.6017453	-0.3170379
H	3.5233803	2.0724533	-0.5348512
C	5.6259208	2.3526104	-0.2562537
C	-1.2891630	-3.3650874	-0.4429742
H	-2.2210007	-3.9146427	-0.4876023
C	-7.3827375	1.2290591	0.2052852
C	-4.8793918	-0.0151389	0.0064553
C	-7.2917608	-0.1442528	0.0083317
C	-6.0629046	-0.7612225	-0.0901236
C	-4.9863209	1.3662328	0.2045579

C	-6.2275525	1.9819453	0.3031981
H	-6.2883804	3.0495219	0.4561551
I	-3.2817765	2.5570366	0.3570557
H	-5.9947404	-1.8281984	-0.2431312
C	5.5932740	3.8370309	-0.4896961
F	6.1161986	4.5063448	0.5456817
F	4.3532091	4.2955367	-0.6746666
F	6.3096330	4.1791227	-1.5681885
C	-8.5640091	-0.9380064	-0.0992824
F	-9.3052284	-0.5327727	-1.1385189
F	-9.3241187	-0.7922462	0.9935864
F	-8.3378450	-2.2435266	-0.2565053
H	-8.3508649	1.7054758	0.2817154
H	7.7508885	2.3445735	0.0599401
H	-0.9308226	-6.0098534	-0.3158931
H	0.8296763	-5.9682281	-0.2164465
H	0.0306663	-5.7942913	-1.7747287

nHBeXB bidentate

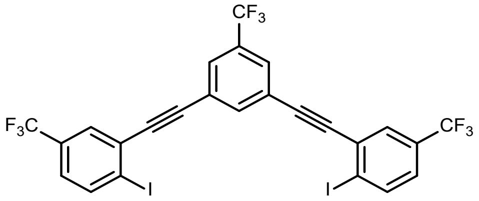

Coordinates for **nHBeXB bidentate**:

0 1

I	-2.9050544	-2.6853110	0.3614407
C	-0.0129098	5.4799344	-0.0057602
C	-7.0794848	-1.6307811	0.0529380
C	-4.6667739	-0.2176083	0.0440308
C	-4.6810610	-1.6117128	0.1702748
C	-3.4568216	0.5337664	0.0347393
C	-1.2090578	1.8989397	0.0180800
C	-2.4386620	1.1741947	0.0257591
C	-5.8815459	-2.3106993	0.1739383
H	-5.8769319	-3.3862454	0.2720601
C	0.0014586	1.2067987	0.0047972
C	2.4406745	1.1761761	-0.0094684
C	-1.2053855	3.2941913	0.0284566
H	-2.1406911	3.8356239	0.0447996
C	1.2102676	1.8994335	0.0028167
C	3.4597423	0.5372366	-0.0233615
C	0.0011059	3.9752432	0.0276180
C	-5.8894641	0.4550551	-0.0771059
H	-5.8872931	1.5307517	-0.1749154
C	-7.0766626	-0.2463947	-0.0721687
C	1.2068012	3.2963454	0.0140478

H	2.1418699	3.8372987	0.0176644
C	7.0846895	-1.6233295	-0.0585965
C	4.6704738	-0.2127457	-0.0384604
C	7.0805675	-0.2396877	0.0744555
C	5.8926391	0.4604754	0.0849449
C	4.6860835	-1.6061075	-0.1726733
C	5.8872966	-2.3037856	-0.1819511
H	5.8836733	-3.3787531	-0.2862945
I	2.9109997	-2.6805740	-0.3675284
H	5.8894866	1.5356028	0.1888196
F	1.1265560	6.0015688	0.4552051
F	-1.0115438	5.9808659	0.7281263
F	-0.1794049	5.9379472	-1.2532665
C	-8.3949163	0.4651719	-0.1999361
F	-9.0793143	0.0355395	-1.2675281
F	-8.2494658	1.7854797	-0.3236268
F	-9.1724206	0.2460969	0.8680214
C	8.3981945	0.4725111	0.2051181
F	9.1769719	0.2562536	-0.8624630
F	9.0817847	0.0410929	1.2725254
F	8.2517056	1.7924604	0.3315378
H	0.0016980	0.1257466	-0.0013476
H	-8.0150382	-2.1740345	0.0558051
H	8.0208115	-2.1655586	-0.0659254

nHBeXB S conformation

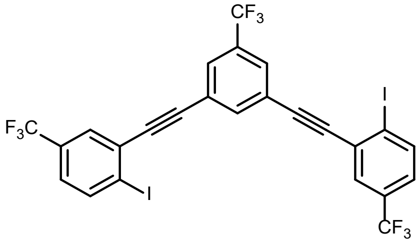

Coordinates for **nHBeXB-S**:
0 1

I	-5.9082569	2.0431822	0.0788376
C	0.0162489	5.0831801	-0.0627421
C	-6.8703310	-2.1639413	0.0013421
C	-4.5105461	-0.6641656	-0.0099292
C	-5.7603749	-0.0342647	0.0281485
C	-3.2890882	0.0684531	-0.0167376
C	-1.0484802	1.4453545	-0.0281618
C	-2.2541751	0.6816007	-0.0223746
C	-6.9306107	-0.7829169	0.0336917
H	-7.8876669	-0.2832661	0.0631667
C	0.1913150	0.8136766	-0.0238649

C	2.6312049	0.9009191	-0.0198380
C	-1.1143705	2.8429525	-0.0367216
H	-2.0768261	3.3334956	-0.0384502
C	1.3679388	1.5652520	-0.0275476
C	3.6785466	0.3093585	-0.0115429
C	0.0550517	3.5788640	-0.0404535
C	-4.4679124	-2.0637975	-0.0423085
H	-3.5076068	-2.5572788	-0.0728235
C	-5.6344966	-2.7991056	-0.0369276
C	1.2949679	2.9568368	-0.0362791
H	2.2026376	3.5446816	-0.0374335
C	7.3995860	-1.6812679	0.0233770
C	4.9228279	-0.3838194	0.0000895
C	7.3339176	-0.2929444	0.0061911
C	6.1148021	0.3516690	-0.0049971
C	5.0002905	-1.7816167	0.0169342
C	6.2325752	-2.4228774	0.0284809
H	6.2768149	-3.5020137	0.0408356
I	3.2735629	-2.9477679	0.0242326
H	6.0639457	1.4305352	-0.0192238
F	0.7340194	5.6047943	0.9391348
F	-1.2253948	5.5591963	0.0443120
F	0.5328853	5.5669594	-1.1992621
C	-5.5999150	-4.3018274	-0.0637940
F	-6.1392956	-4.8221565	1.0460248
F	-4.3579692	-4.7794757	-0.1644451
F	-6.3011139	-4.7877323	-1.0955260
C	8.6192420	0.4871144	0.0126693
F	9.4198950	0.1164977	-0.9941007
F	9.3064245	0.2847541	1.1441751
F	8.4162488	1.8008868	-0.1001384
H	8.3598068	-2.1795395	0.0309704
H	-7.7811934	-2.7476417	0.0047008
H	0.2482856	-0.2655621	-0.0162549

G3HB bidentate

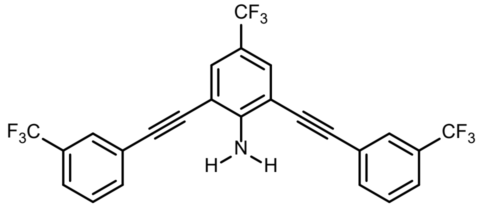

Coordinates for **G3HB bidentate**:
0 1

H	3.8211248	-3.0153264	-1.1781052
C	0.0111564	4.4211812	-0.2321958
N	-0.0028983	-1.2371315	-0.3090547
H	0.8581874	-1.7159598	-0.1073313

H	-0.8641657	-1.7157593	-0.1075718
C	7.0760756	-2.6971833	-0.3088310
C	4.6842477	-1.2615903	-0.2998216
C	4.7265440	-2.5670085	-0.7930016
C	3.4601091	-0.5252114	-0.2925763
C	1.2132361	0.8406200	-0.2838152
C	2.4413588	0.1186766	-0.2874817
C	5.9162103	-3.2778106	-0.7949257
H	5.9381755	-4.2883111	-1.1782063
C	-0.0028651	0.1205861	-0.2822737
C	-2.4458732	0.1209860	-0.2865454
C	1.1978373	2.2299460	-0.2814591
H	2.1376493	2.7645197	-0.2863635
C	-1.2170550	0.8415387	-0.2833336
C	-3.4651021	-0.5222438	-0.2913698
C	-0.0019231	2.9238266	-0.2815358
C	5.8557349	-0.6765136	0.1895434
H	5.8311244	0.3319153	0.5758339
C	7.0350839	-1.3969542	0.1805169
C	-1.2012088	2.2323524	-0.2805305
H	-2.1403743	2.7670015	-0.2843677
C	-7.0820993	-2.6926696	-0.3065565
C	-4.6895849	-1.2579955	-0.2982914
C	-7.0408607	-1.3916465	0.1806316
C	-5.8612092	-0.6716767	0.1892758
C	-4.7321710	-2.5642394	-0.7893357
C	-5.9221161	-3.2745560	-0.7908819
H	-5.9442233	-4.2856929	-1.1724810
H	-3.8266868	-3.0135909	-1.1730820
H	-5.8364662	0.3373745	0.5739258
F	-1.1249129	4.9488809	-0.7005473
F	1.0182553	4.9343152	-0.9499093
F	0.1636239	4.8808036	1.0197713
C	8.3119129	-0.7763597	0.6770681
F	8.9691047	-1.6005728	1.5022003
F	8.0994481	0.3642757	1.3367108
F	9.1463571	-0.4998111	-0.3341397
C	-8.3177437	-0.7696121	0.6752059
F	-8.9764198	-1.5924436	1.5005402
F	-9.1509695	-0.4934983	-0.3371465
F	-8.1051515	0.3715874	1.3338643
H	-8.0144321	-3.2409187	-0.3016378
H	8.0081860	-3.2458201	-0.3042037

G3XB S conformation dihedral freeze

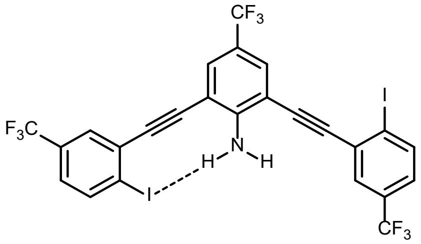

Coordinates for **G3XB-S-dhf**:

0 1

I	-5.9080738	2.0521259	0.0306599
C	-0.0142076	5.1148793	-0.0353816
N	0.2354904	-0.5332595	-0.0200982
H	-0.6123821	-1.0706734	0.0018482
H	1.1236114	-1.0071396	-0.0008309
C	-6.8692945	-2.1561894	0.0060159
C	-4.5072034	-0.6551999	0.0040620
C	-5.7584542	-0.0255032	0.0150666
C	-3.2880497	0.0806676	0.0014693
C	-1.0683595	1.4867714	-0.0053965
C	-2.2705841	0.7263583	-0.0015279
C	-6.9283533	-0.7747504	0.0161921
H	-7.8854151	-0.2742433	0.0245626
C	0.1766432	0.8134351	-0.0115903
C	2.6132429	0.9168292	-0.0072846
C	-1.1100279	2.8751879	0.0023097
H	-2.0730976	3.3673863	0.0125514
C	1.3602015	1.5886573	-0.0084396
C	3.6450722	0.2936965	-0.0058428
C	0.0594759	3.6195360	0.0048920
C	-4.4667772	-2.0553118	-0.0063954
H	-3.5082315	-2.5530653	-0.0167088
C	-5.6329272	-2.7910904	-0.0056764
C	1.2860016	2.9772250	-0.0020416
H	2.2022385	3.5504462	0.0046178
C	7.4055199	-1.6288292	0.0076891
C	4.8983356	-0.3812879	-0.0019319
C	7.3088058	-0.2420347	0.0114348
C	6.0771550	0.3774124	0.0070996
C	5.0104265	-1.7759984	-0.0059774
C	6.2541314	-2.3940427	-0.0012487
H	6.3204773	-3.4721716	-0.0051804
I	3.3114184	-2.9852374	-0.0200732
H	6.0041359	1.4550657	0.0090940
F	1.0947375	5.6848849	0.4491953
F	-1.0489753	5.5822266	0.6742308
F	-0.1708015	5.5775738	-1.2857653
C	-5.5961016	-4.2938196	-0.0087527
F	-6.1444266	-4.7983626	1.1037030

F	-4.3515257	-4.7709661	-0.0897277
F	-6.2854125	-4.7976574	-1.0395182
C	8.5773313	0.5655398	0.0348848
F	9.3947930	0.2175137	-0.9662181
F	9.2570714	0.3704256	1.1720131
F	8.3473476	1.8749785	-0.0720855
H	8.3759428	-2.1066735	0.0097844
H	-7.7802649	-2.7395872	0.0056331

2F-G3XB S conformation dihedral freeze

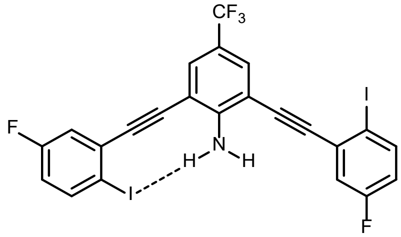

Coordinates for **2F-G3XB-S-dhf**:

0 1

I	5.8511034	0.6698532	-0.0188525
C	0.6289491	4.7631124	0.0226575
N	-0.6618586	-0.7403989	0.0079139
H	0.0721927	-1.4254366	0.0041807
H	-1.6226708	-1.0416940	0.0026787
C	6.0298416	-3.6509700	0.0052489
C	3.9777834	-1.7400724	0.0010200
C	5.3238820	-1.3471248	-0.0057661
C	2.9138597	-0.7928893	-0.0007831
C	0.9945737	1.0017980	-0.0035004
C	2.0344348	0.0309399	-0.0022856
C	6.3335860	-2.2986829	-0.0035839
H	7.3668420	-1.9834802	-0.0087213
C	-0.3538219	0.5718514	0.0003713
C	-2.7291238	1.1253664	-0.0089729
C	1.2938562	2.3583306	-0.0118805
H	2.3317449	2.6624071	-0.0194103
C	-1.3731142	1.5533097	-0.0066699
C	-3.8583727	0.7039051	-0.0102404
C	0.2829393	3.3068608	-0.0189136
C	3.6774854	-3.1066710	0.0099489
H	2.6514903	-3.4443023	0.0154935
C	4.7004273	-4.0272426	0.0118484
C	-1.0418189	2.9035491	-0.0149145
H	-1.8355479	3.6371236	-0.0247772
C	-7.9131114	-0.4984393	-0.0163747
C	-5.2147300	0.2702045	-0.0121696
C	-7.5462150	0.8342321	-0.0246928
C	-6.2295685	1.2343645	-0.0227882

C	-5.5787858	-1.0826364	-0.0035637
C	-6.9139537	-1.4582485	-0.0056973
H	-7.1787256	-2.5055756	0.0011200
I	-4.1306980	-2.5844831	0.0138977
H	-5.9834517	2.2859836	-0.0295545
F	-0.3398124	5.5237869	-0.5008145
F	1.7552557	5.0288808	-0.6502640
F	0.8232512	5.1994868	1.2775631
F	4.3937903	-5.3294957	0.0204499
F	-8.5024075	1.7683387	-0.0349896
H	-8.9587468	-0.7700230	-0.0181973
H	6.8036879	-4.4048816	0.0071025

2H-G3XB S conformation dihedral freeze

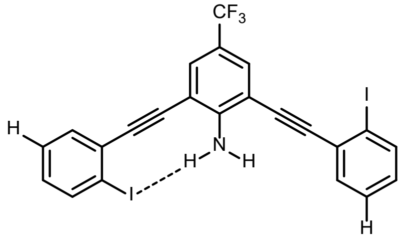

Coordinates for **2H-G3XB-S-dhf**:
0 1

I	5.7651500	0.1406116	-0.0155438
C	0.8569622	4.6138160	0.0175916
N	-0.8315873	-0.7798549	0.0073546
H	-0.1491979	-1.5163654	-0.0037167
H	-1.8120227	-1.0088086	-0.0055894
C	5.6160807	-4.1785427	0.0146770
C	3.7190429	-2.1240517	0.0047434
C	5.0904277	-1.8337565	-0.0006726
C	2.7293709	-1.0990229	0.0000796
C	0.9492882	0.8345410	-0.0065077
C	1.9134554	-0.2119911	-0.0035176
C	6.0288873	-2.8546903	0.0042660
H	7.0819219	-2.6134386	0.0001004
C	-0.4270839	0.5065234	-0.0028416
C	-2.7560168	1.2337087	-0.0128162
C	1.3484216	2.1653927	-0.0158867
H	2.4057893	2.3919480	-0.0234114
C	-1.3716917	1.5601872	-0.0110694
C	-3.9136914	0.8974847	-0.0129524
C	0.4103998	3.1857021	-0.0240998
C	3.3265702	-3.4683383	0.0153621
H	2.2697193	-3.6968043	0.0199520
C	4.2621869	-4.4852990	0.0203033
C	-0.9411483	2.8815566	-0.0199438
H	-1.6782643	3.6721381	-0.0307185

C	-8.0429002	0.0158057	-0.0136422
C	-5.2997094	0.5696003	-0.0131060
C	-7.5999515	1.3316372	-0.0233566
C	-6.2454050	1.6038377	-0.0231436
C	-5.7683100	-0.7496623	-0.0038889
C	-7.1270114	-1.0252311	-0.0040167
H	-7.4682671	-2.0502646	0.0033384
I	-4.4357824	-2.3584483	0.0098211
H	-5.8880422	2.6239910	-0.0305626
F	-0.0219736	5.4315407	-0.5760491
F	2.0378896	4.7882805	-0.5877149
F	1.0034239	5.0612857	1.2756153
H	3.9362068	-5.5159956	0.0285797
H	-8.3108317	2.1460377	-0.0310670
H	-9.1011586	-0.2059626	-0.0136324
H	6.3556766	-4.9673548	0.0185212

2Me-G3XB S conformation dihedral freeze

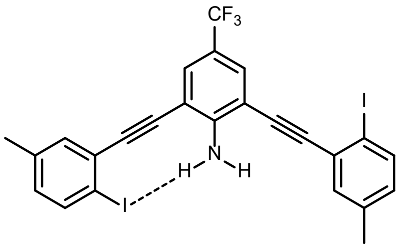

Coordinates for **2Me-G3XB-S-dhf**:

0 1

I	5.8749014	0.7024849	-0.0205846
C	0.5912602	4.7883561	0.0244101
N	-0.6393423	-0.7274005	0.0110345
H	0.1035159	-1.4028172	-0.0033907
H	-1.5967990	-1.0388845	-0.0031151
C	6.0132989	-3.6174651	0.0051613
C	3.9918615	-1.7003311	0.0003902
C	5.3357384	-1.3124212	-0.0068199
C	2.9288127	-0.7514920	-0.0014015
C	0.9982693	1.0319441	-0.0030941
C	2.0462344	0.0693039	-0.0025321
C	6.3382077	-2.2721714	-0.0043872
H	7.3741997	-1.9650105	-0.0098271
C	-0.3458214	0.5887476	0.0014610
C	-2.7275519	1.1142187	-0.0077843
C	1.2829884	2.3914489	-0.0115635
H	2.3175527	2.7065311	-0.0196053
C	-1.3762688	1.5581881	-0.0052697
C	-3.8512066	0.6773962	-0.0096135
C	0.2614660	3.3289840	-0.0179821
C	3.6938573	-3.0700027	0.0101093

H	2.6527974	-3.3665210	0.0161233
C	4.6837231	-4.0373875	0.0126103
C	-1.0591097	2.9117688	-0.0133876
H	-1.8606446	3.6367580	-0.0228687
C	-7.8796758	-0.5521179	-0.0179821
C	-5.2023366	0.2263557	-0.0122593
C	-7.5696086	0.8076600	-0.0262755
C	-6.2351925	1.1749934	-0.0232981
C	-5.5502174	-1.1270820	-0.0040267
C	-6.8830649	-1.5120264	-0.0069488
H	-7.1407382	-2.5614474	-0.0004198
I	-4.0869937	-2.6161763	0.0143622
H	-5.9623086	2.2221049	-0.0295762
F	-0.3853364	5.5398013	-0.4995228
F	1.7151770	5.0684660	-0.6471869
F	0.7795618	5.2280890	1.2796773
C	4.3457185	-5.5017770	0.0230061
H	4.7555835	-5.9997974	-0.8562665
H	3.2690826	-5.6592744	0.0313669
H	4.7678745	-5.9901736	0.9018396
C	-8.6627115	1.8388927	-0.0379525
H	-9.3014401	1.7349142	0.8397067
H	-9.2951028	1.7218365	-0.9185423
H	-8.2546619	2.8473922	-0.0439583
H	-8.9164897	-0.8650654	-0.0200885
H	6.8072243	-4.3542073	0.0070433

2*t*Bu-G3XB S conformation dihedral freeze

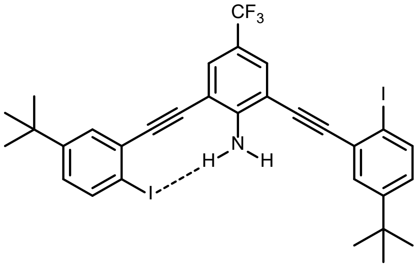

Coordinates for **2*t*Bu-G3XB-S-dhf**:
0 1

I	5.9143222	2.0948356	-0.0311834
C	-0.0126361	5.1210064	0.0367022
N	-0.2156995	-0.5269675	0.0233032
H	0.6380126	-1.0530850	-0.0293484
H	-1.0993485	-1.0076437	-0.0248304
C	6.8661697	-2.1270159	-0.0077946
C	4.5214839	-0.6254910	-0.0062726
C	5.7692027	0.0140856	-0.0163388
C	3.2979427	0.1059133	-0.0041634
C	1.0716457	1.5030572	0.0037203

C	2.2785865	0.7488702	-0.0008529
C	6.9283380	-0.7390809	-0.0170475
H	7.8893769	-0.2450725	-0.0249110
C	-0.1685883	0.8211888	0.0098461
C	-2.6062552	0.9010054	0.0039115
C	1.1022353	2.8915163	-0.0028107
H	2.0615282	3.3910894	-0.0126746
C	-1.3590074	1.5850703	0.0067952
C	-3.6310350	0.2660638	0.0006931
C	-0.0740427	3.6260872	-0.0050541
C	4.4910951	-2.0218160	0.0032010
H	3.5194470	-2.4980979	0.0120061
C	5.6461166	-2.7971106	0.0025376
C	-1.2956054	2.9740761	0.0010939
H	-2.2164257	3.5397436	-0.0056251
C	-7.3626802	-1.6849760	-0.0133389
C	-4.8757430	-0.4277234	-0.0035845
C	-7.3179770	-0.2933933	-0.0183845
C	-6.0636775	0.3080463	-0.0132532
C	-4.9642321	-1.8256042	0.0013986
C	-6.1997381	-2.4446952	-0.0035325
H	-6.2629097	-3.5235292	0.0002676
I	-3.2483964	-3.0155375	0.0169931
H	-5.9765682	1.3862958	-0.0169074
F	-1.1302744	5.6842347	-0.4386988
F	1.0123216	5.6005042	-0.6796904
F	0.1489542	5.5859567	1.2865194
H	-8.3097397	-2.2041828	-0.0170353
H	7.7951804	-2.6780183	-0.0087423
C	-8.5709625	0.5798354	-0.0293594
C	-8.5780413	1.4716265	1.2199682
C	-8.5626538	1.4621271	-1.2854183
C	-9.8510739	-0.2551165	-0.0340700
H	-8.5801839	0.8663176	2.1272647
H	-7.7069975	2.1257595	1.2519185
H	-9.4702319	2.0999094	1.2233773
H	-8.5531880	0.8499526	-2.1880553
H	-9.4549462	2.0899707	-1.3048124
H	-7.6915181	2.1164019	-1.3114025
H	-10.7152279	0.4096241	-0.0418950
H	-9.9126029	-0.8917719	-0.9178531
H	-9.9234937	-0.8850374	0.8537002
C	5.5319976	-4.3202625	0.0130922
C	4.7747357	-4.7641071	1.2722846
C	4.7626892	-4.7804426	-1.2328773
C	6.9022711	-4.9971800	0.0108586
H	5.3012067	-4.4453547	2.1726789
H	3.7684292	-4.3467428	1.3041524

H	4.6881314	-5.8517983	1.2909371
H	5.2803967	-4.4733525	-2.1423755
H	4.6760305	-5.8682806	-1.2366305
H	3.7560477	-4.3635496	-1.2603186
H	6.7704981	-6.0795024	0.0182087
H	7.4771298	-4.7373493	-0.8791199
H	7.4854177	-4.7263444	0.8921164

2MeO-G3XB S conformation dihedral freeze

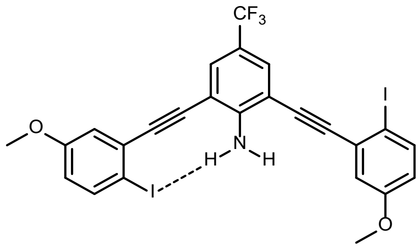

Coordinates for **2MeO-G3XB-S-dhf**:

0 1

I	5.8845788	1.2724940	-0.0293463
C	0.3546521	4.9278865	0.0313147
N	-0.4901411	-0.6604340	0.0153164
H	0.2974790	-1.2828766	-0.0100163
H	-1.4233076	-1.0381010	-0.0083632
C	6.4053185	-3.0277214	0.0043669
C	4.2126796	-1.2884571	-0.0015796
C	5.5233941	-0.7811981	-0.0100342
C	3.0749023	-0.4296410	-0.0026104
C	1.0205667	1.2090611	0.0001427
C	2.1342597	0.3234171	-0.0017550
C	6.5970580	-1.6509885	-0.0070416
H	7.6039639	-1.2586411	-0.0136432
C	-0.2892782	0.6727502	0.0052833
C	-2.7025689	1.0340470	-0.0023364
C	1.2099894	2.5850085	-0.0076635
H	2.2202360	2.9711984	-0.0165934
C	-1.3844264	1.5685087	0.0003114
C	-3.7966037	0.5283697	-0.0048941
C	0.1257810	3.4493601	-0.0118266
C	4.0281064	-2.6681940	0.0097939
H	3.0299456	-3.0822257	0.0170238
C	5.1118283	-3.5389420	0.0126675
C	-1.1624855	2.9409036	-0.0063381
H	-2.0127635	3.6081037	-0.0141263
C	-7.7429891	-0.9822963	-0.0164341
C	-5.1157517	-0.0111595	-0.0085071
C	-7.5065452	0.3887793	-0.0225240
C	-6.1995575	0.8632160	-0.0185498
C	-5.3677506	-1.3926973	-0.0023988

C	-6.6669082	-1.8615994	-0.0064233
H	-6.8544171	-2.9257170	-0.0016323
I	-3.8047782	-2.7756818	0.0136308
H	-6.0311212	1.9304511	-0.0234232
F	-0.6772554	5.6115889	-0.4785329
F	1.4487203	5.2860137	-0.6527652
F	0.5281515	5.3763325	1.2856051
O	4.8099997	-4.8578808	0.0240625
O	-8.4759996	1.3307747	-0.0322799
H	-8.7462523	-1.3799312	-0.0192647
H	7.2665967	-3.6781280	0.0065776
C	5.8806193	-5.7766408	0.0249127
C	-9.8179728	0.8950352	-0.0369884
H	-10.4280850	1.7928754	-0.0450215
H	-10.0451732	0.3092618	0.8566320
H	-10.0357891	0.2996444	-0.9265846
H	5.4320708	-6.7652532	0.0323902
H	6.5045241	-5.6539890	0.9131952
H	6.4971935	-5.6640923	-0.8697946

X-ray Diffraction Data

Single Crystal X-ray Diffraction Methods and Refinement

X-ray diffraction data were collected at 100 K (unless noted below) on a Bruker D8 Venture using MoKα-radiation (λ=0.71073 Å). Data have been corrected for absorption using SADABS[8] area detector absorption correction program. Using Olex2[9], the structure was solved with the SHELXT[10] structure solution program using Direct Methods and refined with the SHELXL[11] refinement package using least squares minimization. All non-hydrogen atoms were refined with anisotropic thermal parameters. Hydrogen atoms of the investigated structure were located from difference Fourier maps but finally their positions were placed in geometrically calculated positions and refined using a riding model. Isotropic thermal parameters of the placed hydrogen atoms were fixed to 1.2 times the *U* value of the atoms they are linked to (1.5 for methyl groups). Hydrogen atoms connected to heteroatoms were located from difference maps placed and refined. Calculations and refinement of structures were carried out using APEX3[12], SHELXTL[13], and Olex2 software. Specific and/or unique individual structure refinement details and crystal growth conditions are presented below.

Table S4-1. **Crystallographic Data**

Identification code	UMT_OB112	UMT_OB218	UMT_OB162	UMT_OB144	UMT_OB220
Manu Reference	G3XB	nHBeXB	G3HB-triclinic	G3HB-orthorhombic	2H-G3XB
CCDC Number	2023091	2238833	2023092	2023093	2238834
Empirical formula	$C_{25}H_{10}F_9I_2N$	$C_{25}H_9F_9I_2$	$C_{25}H_{12}F_9N$	$C_{25}H_{12}F_9N$	$C_{23}H_{12}F_3I_2N$

Formula weight	749.14	734.12	497.36	497.36	613.14
Temperature/K	105	100	100	100	100
Crystal system	monoclinic	monoclinic	triclinic	orthorhombic	monoclinic
Space group	$P2_1/c$	$P2_1/c$	P-1	Fdd2	$P2_1$
a/Å	17.0483(14)	18.7496(16)	7.9820(4)	37.842(3)	8.4061(3)
b/Å	4.7023(4)	4.7821(4)	7.9889(4)	50.545(4)	20.5959(7)
c/Å	30.687(3)	25.949(2)	17.3963(9)	8.9837(6)	12.3043(4)
α/°	90	90	84.337(2)	90	90
β/°	103.370(2)	94.215(2)	89.492(2)	90	108.6440(10)
γ/°	90	90	70.559(2)	90	90
Volume/$Å^3$	2393.4(3)	2320.4(3)	1040.62(9)	17183(2)	2018.47(12)
Z	4	4	2	32	4
$\rho_{calc}g/cm^3$	2.079	2.101	1.587	1.538	2.018
μ/mm^{-1}	2.714	2.796	0.150	0.145	3.152
F(000)	1416.0	1384.0	500.0	8000.0	1160.0
Crystal size/mm^3	0.46 × 0.02 × 0.01	0.46 × 0.02 × 0.01	0.39 × 0.07 × 0.02	0.55 × 0.32 × 0.09	0.12 × 0.07 × 0.01
Radiation	MoKα (λ = 0.71073)	MoKα (λ = 0.71073)	MoKα (λ = 0.71073)	MoKα (λ = 0.71073)	MoKα (λ = 0.71073)
2Θ range for data collection/°	6.146 to 55.072	5.184 to 50.118	5.414 to 55.106	5.25 to 52.936	5.114 to 55.074
Index ranges	-21 ≤ h ≤ 22, -6 ≤ k ≤ 6, -39 ≤ l ≤ 39	-22 ≤ h ≤ 22, -5 ≤ k ≤ 5, -28 ≤ l ≤ 30	-10 ≤ h ≤ 10, -10 ≤ k ≤ 10, -22 ≤ l ≤ 22	-47 ≤ h ≤ 47, -62 ≤ k ≤ 63, -11 ≤ l ≤ 11	-10 ≤ h ≤ 10, -26 ≤ k ≤ 26, -15 ≤ l ≤ 15
Reflections collected	51830	15943	47574	130424	75201
Independent reflections	5489 [R_{int} = 0.0667, R_{sigma} = 0.0419]	4089 [R_{int} = 0.0771, R_{sigma} = 0.0770]	4794 [R_{int} = 0.0459, R_{sigma} = 0.0252]	8847 [R_{int} = 0.0464, R_{sigma} = 0.0232]	9280 [R_{int} = 0.0447, R_{sigma} = 0.0250]
Data/restraints/parameters	5489/2/342	4089/21/353	4794/15/352	8847/37/703	9280/5/540
Goodness-of-fit on F^2	1.060	1.022	1.017	1.054	1.086
Final R indexes [I>=2σ (I)]	R_1 = 0.0364, wR_2 = 0.0734	R_1 = 0.0441, wR_2 = 0.0690	R_1 = 0.0390, wR_2 = 0.0892	R_1 = 0.0564, wR_2 = 0.1408	R_1 = 0.0254, wR_2 = 0.0559
Final R indexes [all data]	R_1 = 0.0598, wR_2 = 0.0805	R_1 = 0.0806, wR_2 = 0.0776	R_1 = 0.0600, wR_2 = 0.0991	R_1 = 0.0660, wR_2 = 0.1475	R_1 = 0.0312, wR_2 = 0.0579
Largest diff. peak/hole / e $Å^{-3}$	1.08/-0.81	0.66/-0.63	0.46/-0.31	0.64/-0.38	1.71/-0.36

Table S4-2. **Crystallographic Data Continued**

Identification code	UMT_OB229	UMT_OB227	UMT_OB129	UMT_OB148	UMT_OB196
Manu Reference	Me-G3XB	F-G3XB	G3XB-Cl^-	2H-G3XB-Cl^-	2Me-G3XB-Cl^-
CCDC number	2238835	2238836	2238837	2238838	2238839
Empirical formula	$C_{25}H_{13}F_6I_2N$	$C_{24}H_{10}F_7I_2N$	$C_{41}H_{46}ClF_9I_2N_2$	$C_{50}H_{67.69}Cl_{1.25}F_3I_2N_{2.25}$	$C_{41}H_{52}ClF_3I_2N_2$
Formula weight	695.16	699.13	1027.05	1055.37	919.09
Temperature/K	100	100	105	100	100
Crystal system	monoclinic	monoclinic	triclinic	tetragonal	triclinic
Space group	$P2_1/n$	C2/c	P-1	I-4	P-1
a/Å	18.182(4)	7.3702(7)	8.0763(5)	36.9115(18)	12.2702(5)
b/Å	4.5894(10)	18.1535(18)	15.6429(11)	36.9115(18)	13.0397(5)
c/Å	29.337(6)	34.027(3)	17.8668(12)	8.3724(4)	14.4541(6)
α/°	90	90	73.593(2)	90	107.965(2)
β/°	106.945(4)	94.009(2)	87.048(2)	90	108.3600(10)
γ/°	90	90	88.270(2)	90	93.925(2)
Volume/$Å^3$	2341.8(9)	4541.5(8)	2162.2(3)	11407.0(12)	2052.49(15)
Z	4	8	2	8	2
$\rho_{calc}g/cm^3$	1.972	2.045	1.578	1.229	1.487
μ/mm^{-1}	2.747	2.840	1.586	1.201	1.641
F(000)	1320.0	2640.0	1020.0	4302.0	924.0
Crystal size/mm^3	0.41 × 0.12 × 0.01	0.19 × 0.05 × 0.01	0.38 × 0.31 × 0.09	0.66 × 0.06 × 0.04	0.81 × 0.21 × 0.11
Radiation	MoKα (λ = 0.71073)	MoKα (λ = 0.71073)	MoKα (λ = 0.71073)	MoKα (λ = 0.71073)	MoKα (λ = 0.71073)
2Θ range for data collection/°	5.514 to 50.156	5.09 to 50.198	6.036 to 54.968	4.936 to 50.094	5.308 to 54.966
Index ranges	-20 ≤ h ≤ 21, -5 ≤ k ≤ 5, -34 ≤ l ≤ 34	-8 ≤ h ≤ 8, -21 ≤ k ≤ 21, -40 ≤ l ≤ 40	-10 ≤ h ≤ 10, -20 ≤ k ≤ 20, -23 ≤ l ≤ 23	-43 ≤ h ≤ 43, -43 ≤ k ≤ 43, -9 ≤ l ≤ 8	-15 ≤ h ≤ 15, -16 ≤ k ≤ 16, -18 ≤ l ≤ 18
Reflections collected	19640	47545	134813	86060	119487
Independent reflections	4155 [R_{int} = 0.0532, R_{sigma} = 0.0463]	4043 [R_{int} = 0.0621, R_{sigma} = 0.0319]	9902 [R_{int} = 0.0633, R_{sigma} = 0.0268]	10071 [R_{int} = 0.0551, R_{sigma} = 0.0288]	9379 [R_{int} = 0.0233, R_{sigma} = 0.0093]

Data/restraints /parameters	4155/2/316	4043/2/315	9902/2/508	10071/92/578	9379/97/524
Goodness-of-fit on F^2	1.179	1.183	1.146	1.052	1.152
Final R indexes [I>=2σ (I)]	R_1 = 0.0413, wR_2 = 0.0775	R_1 = 0.0404, wR_2 = 0.0634	R_1 = 0.0507, wR_2 = 0.1202	R_1 = 0.0334, wR_2 = 0.0697	R_1 = 0.0232, wR_2 = 0.0532
Final R indexes [all data]	R_1 = 0.0587, wR_2 = 0.0817	R_1 = 0.0556, wR_2 = 0.0667	R_1 = 0.0654, wR_2 = 0.1324	R_1 = 0.0408, wR_2 = 0.0736	R_1 = 0.0287, wR_2 = 0.0590
Largest diff. peak/hole / e $Å^{-3}$	1.38/-0.97	0.72/-0.64	2.37/-0.99	0.74/-0.38	1.37/-0.51

Table S4-3. **Crystallographic Data Continued**

Identification code	UMT_OB219
Manu Reference	H-G3XB-Cl^-
CCDC number	2238840
Empirical formula	$C_{48}H_{63}ClF_6I_2N_2$
Formula weight	1071.25
Temperature/K	100
Crystal system	monoclinic
Space group	$P2_1/c$
a/Å	8.2189(5)
b/Å	22.0601(14)
c/Å	26.8587(18)
α/°	90
β/°	93.235(2)
γ/°	90
Volume/$Å^3$	4862.0(5)
Z	4
$\rho_{calc}g/cm^3$	1.463
μ/mm^{-1}	1.405
F(000)	2168.0
Crystal size/mm^3	0.26 × 0.21 × 0.2
Radiation	MoKα (λ = 0.71073)
2Θ range for data collection/°	5.296 to 61.15

Index ranges	$-11 \le h \le 11$, $-31 \le k \le 31$, $-38 \le l \le 38$
Reflections collected	181343
Independent reflections	14933 [R_{int} = 0.0493, R_{sigma} = 0.0237]
Data/restraints /parameters	14933/0/544
Goodness-of-fit on F^2	1.080
Final R indexes [I>=2σ (I)]	R_1 = 0.0293, wR_2 = 0.0665
Final R indexes [all data]	R_1 = 0.0405, wR_2 = 0.0707
Largest diff. peak/hole / e Å^{-3}	1.25/-0.72

Crystal Growth Conditions and Additional Refinement Details

G3XB
Diffraction quality crystals of **G3XB** were grown by vapor diffusion of diethyl ether into an ethyl acetate solution of **G3XB** resulting in yellow needles.

Diffraction data were collected at 105 K. The hydrogen atom locations of the aniline amine were found from the difference maps, placed, and refined with isotropic thermal parameters. Upon refinement the N—H bond distances reduced to an unreasonable length necessitating the use of bond distance restraints (DFIX 0.87 0.02).

nHBeXB
Colorless needles were obtained by slow evaporation of a methanol solution of the parent compound.

After initial refinement, it was apparent the trifluoromethyl (CF_3) group on the central benzene ring was disordered. The disorder was modeled over two positions using a PART instruction and tied to an individual free variable. Refinement of the free variable showed an approximate 70:30 disorder for the CF_3 group. The disorder model incorporated both bond length and thermal ellipsoid similarity restraints (SADI 0.02 and SIMU 0.08).

G3HB-triclinic
Diffraction quality crystals of **G3HB** were grown by slow evaporation of a benzene solution of **G3HB** resulting in colorless rods.

During anisotropic refinement, the fluorine atoms of one CF_3 group exhibited elongated ellipsoids suggesting disorder. The fluorine atoms of the CF_3 group were subsequently modeled over two positions using a PART instruction and tied to an individual free variable. Refinement of the free variable shows an approximate 57:43 disorder for the CF_3 group. In modeling the disorder, additional bond length (SADI 0.02) and thermal ellipsoid (SIMU 0.04 0.08) similarity restraints were utilized.

G3HB-orthorhombic

Diffraction quality crystals of **G3HB** were grown by vapor diffusion of diethyl ether into an ethyl acetate solution of **G3HB** resulting in colorless plates.

Several CF_3 groups displayed disorder. Two of the groups were modeled over two positions using a PART instruction and tied to an individual free variable. Refinement of the free variables shows an approximate 55:45 and 57:43 disorder for the CF_3 groups. The disorder of these CF_3 groups is likely dynamic in nature, and there was no improvement in the model by modeling this disorder of this group over 3 or more sites. One fluorine atom in the model presented has an elongated ellipsoid causing Level B Alert in the checkCIF suggesting further disorder. Attempts at producing suitable disorder model were unsuccessful. Contributing to the difficulty of finding was suitable model was the lack of observable secondary sites for the other fluorine atoms in the CF_3 group containing fluorine F8A. As such a model of simplicity was chosen for as a more complex disorder model would not significantly enhance the results and would necessitate several geometrical and thermal constraints.

2H-G3XB

Light yellow plates were grown from by slow evaporation of a acetone solution of **2H-G3XB.**

The hydrogen atoms of the amine required a N—H bond distance restraint (DFIX 0.87 0.02) due to unreasonable lengthening of the N—H bond upon initial refinement.

Me-G3XB

Yellow plank-like crystals of **Me-G3XB** were grown by slow evaporation of an diethyl ether and methanol solution of **Me-G3XB** and tetraphenylphosphonium chloride.

The hydrogen atoms of the amine required a N—H bond distance restraint (DFIX 0.87 0.02) due to unreasonable shortening of the N—H bond upon refinement.

F-G3XB

Yellow plates were grown by slow evaporation of an ethyl acetate and hexanes solution of **F-G3XB** and tetraphenylphosphonium iodide.

The hydrogen atoms of the amine required a N—H bond distance restraint (DFIX 0.87 0.02) due to unreasonable shortening of the N—H bond upon refinement.

G3XB-Cl^-

Colorless plates of **G3XB-Cl**$^{-}$ were grown by vapor diffusion of hexanes into a benzene solution of **G3XB** and tetra-*n*-butyl ammonium chloride.

Diffraction data were collected at 105 K. The hydrogen atoms of the amine required a N—H bond distance restraint (DFIX 0.87 0.02) due to unreasonable shortening of the N—H bond upon refinement.

2H-G3XB-Cl^{-}

Colorless needles of **2H-G3XB-Cl**$^{-}$ were grown by vapor diffusion of hexanes into a benzene solution of **2H-G3XB** and tetra-*n*-hexyl ammonium chloride.

Upon initial solution of the data, it was evident that one of the hexyl groups of the tetra-*n*-hexylammonium (THA) molecules was severely disordered. Subsequent modeling and refinement indicated that proximal to the hexyl disordered region a benzene molecule had partial occupancy. After several hours of considering disorder models we conceded. The various models we came up with to describe this region were not completely satisfactory, as the benzene molecule and the hexyl groups were always too close to each other, meaning the models were not chemically reasonable. All this despite evidence from the difference map the presence of a partially occupied benzene is present*, and the existence of THA hexyl group is undeniable. Our best overall disorder model described three locations of the disordered alkyl portion of the THA that were modeled and refined using free variables tied to a SUMP command to ensure full occupancy. To ensure a stable refinement here, various bond length and angle (1,3 distances) similarity restraints as well as thermal ellipsoids restraints and constraints were employed. The benzene molecule was geometrically constrained to a hexagonal shape, treated with a PART -1 command, and refined freely using a free variable. After treating the data in this manner there remained some diffuse electron density located in a channel that can be viewed by viewing a packing diagram down the crystallographic *c* axis. The disordered solvent in this channel was most likely hexane, considering the crystals were grown from a benzene/hexanes vapor diffusion. The SQUEEZE[14] routine within PLATON[15] was utilized to account for the residual, diffuse electron density and the model is refined against these data. A total of 78 electrons per unit cell were corrected for. Collectively this data led to an R_1 statistic ($I > 2\sigma(I)$) of 3.46%.

Considering the extensive use of restraints and constraints to model the disorder in a manner not fully chemically reasonable and the fact that no additional information is gained by modeling this region we ultimately decided to treat the disorder of hexyl chain and the benzene molecule using the SQUEEZE program. The program accounted for 412 electrons per unit cell (this included the diffuse solvent molecules found in the channels that propagate along the crystallographic *c* axis). Treating the data in this manner led to a minor improvement in the R_1 statistic to 3.35 % ($I > 2\sigma(I)$) over the model described in the previous paragraph.

*We considered that the benzene might not be located here and that potentially it could be intertwined hexyl disorder and the Q-peaks are just coincidentally in a roughly hexagonal shape. However, we feel this is unlikely. First, the crystals were grown using benzene, so the

presence of this species would be understandable. Second, for the hexyl group to reach several of the residual electron density peaks (of the benzene) the cation would need an additional carbon atom on the alkyl chain or the entire cation would need to be translated which the data do not support.

2Me-G3XB-Cl$^-$

Colorless prisms of **2Me-G3XB-Cl$^-$** were grown by vapor diffusion of hexanes into a benzene solution of **2Me-G3XB** and tetra-*n*-butyl ammonium chloride.

H-G3XB-Cl$^-$

Yellow Rods of **H-G3XB-Cl$^-$** were obtained by vapor diffusion of hexanes into a benzene solution of **H-G3XB** and tetra-*n*-butyl ammonium chloride.

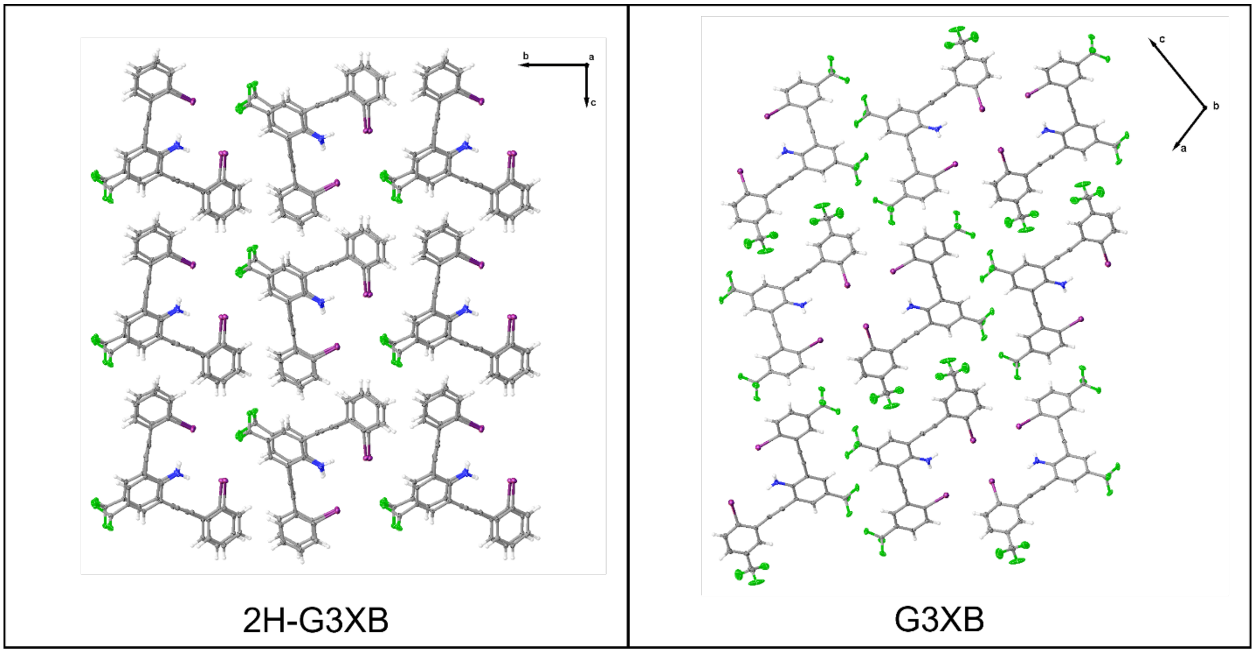

Figure S90. Crystal packing diagram showing one example of the long-range packing differences between **G3XB** and **2H-G3XB**. Thermal ellipsoids are drawn at the 50% probability level.

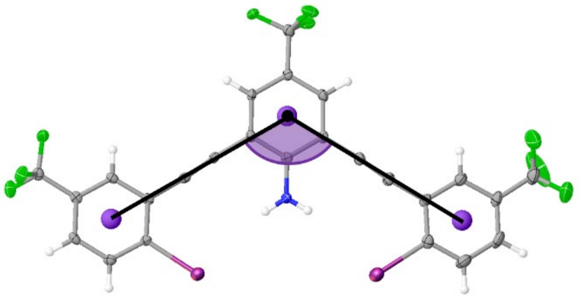

Figure S91. Image of **G3XB** showing the angle formed by the centroids of the three rings (i.e. flanking-core-flanking angle). Thermal ellipsoids are drawn at the 50% probability level.

Table S5. **Intramolecular HBing N-H…I distances of G3XB derivatives**

	Space group	N-H···I_1 (Å)	N-H···I_2 (Å)	Average (Å)
**G3XB**	$P2_1/c$	3.12	3.20	3.16
**2H-G3XB**	$P2_1$	3.16 / 3.16	3.00 / 2.99	3.08/3.08
**Me-G3XB**	$P2_1/n$	3.16	3.48	3.32
**F-G3XB (S conformation)**	C2/c	3.13		

Methoxy derivative

We also evaluated the **2R$_2$-G3XB** receptor containing methoxy substituent. The methoxy group at the *para* position is considered an electron withdrawing substituent by inductive/field effect, but electron-releasing by resonance effect. We initially assumed the *para* methoxy substituent would decrease the XB donor ability the most according to its Hammet parameter (σ_{para} = -0.268). However, noticed that the **2MeO-G3XB** derivative deviated from the expected electronic trend several times. For example, the difference between $\Delta V_{s,max}$ of **G3XB** and $\Delta V_{s,max}$ of **2MeO-G3XB** when ruling out the differences in planarity should tell us how **R$_2$** affect the iodine $V_{s,max}$ via intramolecular HBing. However, this value was not the highest as expected. Additionally, the **2MeO-G3XB** did not have the lowest association constant (K_a = 62 M^{-1}) among **2R$_2$-G3XB** derivatives. With this derivative included in the LFER analysis, a poor correlation is obtained for both σ_{meta} (R^2=0.88) and σ_{para} (R^2=0.89). This deviation from the expected trend potentially indicates a subtle interplay between preorganization and maximizing the XB donor strength and makes us question the utility of using the methoxy Hammett parameters for interpreting molecular recognition.

References

1. Riel, Asia Marie S., et al., *Chem. Sci.,* 2018, **9(26)**, 5828-5836.
2. Pollock J B, et al., *Journal of the American Chemical Society*, 2012, **134(25)**: 10607-10620.
3. Nair, Reji N., et al., *Topics in Catalysis* 2010, **53**, 1045-1047.
4. Asmus S, Beckendorf S, et al., *Chemistry–An Asian Journal*, 2014, **9(8)**: 2178-2186.
5. http://supramolecular.org/
6. M. Charton, *Progress in physical organic chemistry*, 1981, 119-251.
7. W. P. Ozimiński and J. C. Dobrowolski, *Journal of Physical Organic Chemistry*, 2009, **22**, 769-778.
8. Sheldrick, G. M. (1996). SADABS: Area Detector Absorption Correction; University of Göttingen, Germany.
9. Dolomanov, O.V.; Bourhis, L.J.; Gildea, R.J.; Howard, J.A.K.; Puschmann, H., (2009). J. Appl. Cryst., **42**, 339-341.
10. Sheldrick, G. M. (2015). *Acta Cryst.* **A71**, 3-8.
11. Sheldrick, G. M. (2015). *Acta Cryst.* **C71**, 3-8.
12. Bruker (2016). APEX3. Bruker AXS Inc., Madison, Wisconsin, USA.
13. Sheldrick, G.M. (2008). Acta Cryst. **A64**, 112-122.
14. Spek, A. L. (2015). Acta Cryst. **C71**, 9-18.
15. Spek, A. L. (2009). Acta Cryst. **D65**, 148-155.
